# An elite *RNA-DEPENDENT RNA POLYMERASE3* allele enhances preharvest sprouting resistance in rice

**DOI:** 10.1093/plphys/kiaf282

**Published:** 2025-08-22

**Authors:** Huazhong Guan, Zhiwei Chen, Shuoxun Wang, Bo Jiang, Jinxiang Zhao, Yijin Gao, Suming Zhu, Shiying Li, Damei Mao, Lu Lin, Weishu Fan, Qiang Liu, Wenzhen Song, Likun Huang, Xiaofang Xie, Chuanlong Wan, Yafeng Ye, Shujing Cheng, Jinfang Chu, Xiangdong Fu, Weiren Wu, Kun Wu

**Affiliations:** Fujian Provincial Key Laboratory of Crop Breeding by Design, Fujian Agriculture and Forestry University, Fuzhou, Fujian 350002, China; Key Laboratory of Genetics, Breeding and Multiple Utilization of Crops, Ministry of Education, Fujian Agriculture and Forestry University, Fuzhou, Fujian 350002, China; Fujian Provincial Key Laboratory of Crop Breeding by Design, Fujian Agriculture and Forestry University, Fuzhou, Fujian 350002, China; Key Laboratory of Genetics, Breeding and Multiple Utilization of Crops, Ministry of Education, Fujian Agriculture and Forestry University, Fuzhou, Fujian 350002, China; State Key Laboratory of Seed Innovation, Institute of Genetics and Developmental Biology, Chinese Academy of Sciences, Beijing 100101, China; Fujian Provincial Key Laboratory of Crop Breeding by Design, Fujian Agriculture and Forestry University, Fuzhou, Fujian 350002, China; Key Laboratory of Genetics, Breeding and Multiple Utilization of Crops, Ministry of Education, Fujian Agriculture and Forestry University, Fuzhou, Fujian 350002, China; Fujian Provincial Key Laboratory of Crop Breeding by Design, Fujian Agriculture and Forestry University, Fuzhou, Fujian 350002, China; Key Laboratory of Genetics, Breeding and Multiple Utilization of Crops, Ministry of Education, Fujian Agriculture and Forestry University, Fuzhou, Fujian 350002, China; Fujian Provincial Key Laboratory of Crop Breeding by Design, Fujian Agriculture and Forestry University, Fuzhou, Fujian 350002, China; Key Laboratory of Genetics, Breeding and Multiple Utilization of Crops, Ministry of Education, Fujian Agriculture and Forestry University, Fuzhou, Fujian 350002, China; Fujian Provincial Key Laboratory of Crop Breeding by Design, Fujian Agriculture and Forestry University, Fuzhou, Fujian 350002, China; Key Laboratory of Genetics, Breeding and Multiple Utilization of Crops, Ministry of Education, Fujian Agriculture and Forestry University, Fuzhou, Fujian 350002, China; Fujian Provincial Key Laboratory of Crop Breeding by Design, Fujian Agriculture and Forestry University, Fuzhou, Fujian 350002, China; Key Laboratory of Genetics, Breeding and Multiple Utilization of Crops, Ministry of Education, Fujian Agriculture and Forestry University, Fuzhou, Fujian 350002, China; Fujian Provincial Key Laboratory of Crop Breeding by Design, Fujian Agriculture and Forestry University, Fuzhou, Fujian 350002, China; Key Laboratory of Genetics, Breeding and Multiple Utilization of Crops, Ministry of Education, Fujian Agriculture and Forestry University, Fuzhou, Fujian 350002, China; Fujian Provincial Key Laboratory of Crop Breeding by Design, Fujian Agriculture and Forestry University, Fuzhou, Fujian 350002, China; Key Laboratory of Genetics, Breeding and Multiple Utilization of Crops, Ministry of Education, Fujian Agriculture and Forestry University, Fuzhou, Fujian 350002, China; State Key Laboratory of Seed Innovation, Institute of Genetics and Developmental Biology, Chinese Academy of Sciences, Beijing 100101, China; State Key Laboratory of Seed Innovation, Institute of Genetics and Developmental Biology, Chinese Academy of Sciences, Beijing 100101, China; State Key Laboratory of Seed Innovation, Institute of Genetics and Developmental Biology, Chinese Academy of Sciences, Beijing 100101, China; Fujian Provincial Key Laboratory of Crop Breeding by Design, Fujian Agriculture and Forestry University, Fuzhou, Fujian 350002, China; Key Laboratory of Genetics, Breeding and Multiple Utilization of Crops, Ministry of Education, Fujian Agriculture and Forestry University, Fuzhou, Fujian 350002, China; Fujian Provincial Key Laboratory of Crop Breeding by Design, Fujian Agriculture and Forestry University, Fuzhou, Fujian 350002, China; Key Laboratory of Genetics, Breeding and Multiple Utilization of Crops, Ministry of Education, Fujian Agriculture and Forestry University, Fuzhou, Fujian 350002, China; Fujian Provincial Key Laboratory of Crop Breeding by Design, Fujian Agriculture and Forestry University, Fuzhou, Fujian 350002, China; Key Laboratory of Genetics, Breeding and Multiple Utilization of Crops, Ministry of Education, Fujian Agriculture and Forestry University, Fuzhou, Fujian 350002, China; Key Laboratory of High Magnetic Field and Ion Beam Physical Biology, Hefei Institutes of Physical Science, Chinese Academy of Sciences, Hefei 230031, China; State Key Laboratory of Seed Innovation, Institute of Genetics and Developmental Biology, Chinese Academy of Sciences, Beijing 100101, China; State Key Laboratory of Seed Innovation, Institute of Genetics and Developmental Biology, Chinese Academy of Sciences, Beijing 100101, China; College of Life Sciences, University of Chinese Academy of Sciences, Beijing 100049, China; State Key Laboratory of Seed Innovation, Institute of Genetics and Developmental Biology, Chinese Academy of Sciences, Beijing 100101, China; College of Life Sciences, University of Chinese Academy of Sciences, Beijing 100049, China; New Cornerstone Science Laboratory, Institute of Genetics and Developmental Biology, Chinese Academy of Sciences, Beijing 100101, China; Fujian Provincial Key Laboratory of Crop Breeding by Design, Fujian Agriculture and Forestry University, Fuzhou, Fujian 350002, China; Key Laboratory of Genetics, Breeding and Multiple Utilization of Crops, Ministry of Education, Fujian Agriculture and Forestry University, Fuzhou, Fujian 350002, China; State Key Laboratory of Seed Innovation, Institute of Genetics and Developmental Biology, Chinese Academy of Sciences, Beijing 100101, China

## Abstract

A new elite allele conferring enhanced preharvest sprouting resistance via hormonal pathways is a valuable target for molecular design breeding to improve seed quality and production stability.

Dear Editor,

Preharvest sprouting (PHS) substantially threatens agricultural production by reducing both grain yield and quality, especially in regions with high rainfall or typhoon activity (Xu et al. 2022). Rice (*Oryza sativa* L.) varieties exhibit variations in PHS resistance. For instance, *japonica* rice variety Nipponbare (NIP) displays significantly higher PHS resistance compared to *indica* rice variety Jiafuzhan (JFZ; Fig. 1A and B). To explore the genetic basis of PHS resistance, a set of random crossing-recombinant inbred lines (RC-RILs) were developed from the cross between NIP and JianfuzhanS (JFZS), a dominant nuclear male sterile line with JFZ background (Supplementary Fig. S1). The RC-RILs displayed transgressive inheritance with significant continuous variation in germination rate (Fig. 1C). Bulked segregant analysis by deep sequencing (BSA-seq) revealed 4 major QTLs conferring PHS resistance, with the resistant alleles of *qPHS1a* and *qPHS4a* deriving from NIP, while those of *qPHS1b and qPHS7a* from JFZ (Fig. 1D; Supplementary Table S1). The confidence intervals of *qPHS1b* and *qPHS7a* covered *Gibberellin* (*GA*) *2-oxidases3* (*GA2ox3*) and *Seed Dormancy4* (*Sdr4*), respectively, which were previously reported to regulate PHS resistance in rice (Magwa et al. 2016; Zhao et al. 2022; Fig. 1D). To isolate the gene responsible for *qPHS1a*, a residual heterozygous line (RHL23) carrying a heterozygous region containing *qPHS1a* was selected from the RC-RIL population. We observed that plants with the homozygous *qPHS1a^JFZ^* and *qPHS1a^NIP^* genotypes in RHL23 exhibited the highest and lowest germination rates, respectively, while those with the heterozygous genotype displayed moderate germination rate close to the mid-point (Fig. 1E). These results suggest that this QTL mainly has additive effect with the *qPHS1a^NIP^* allele enhancing PHS resistance.

**Figure 1.**
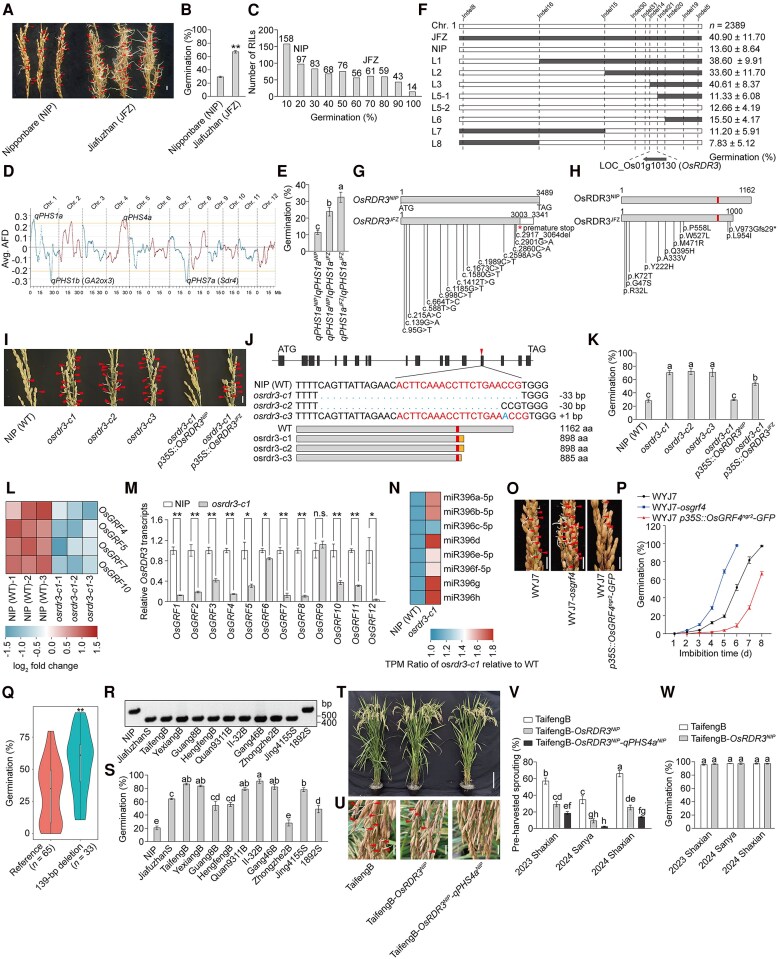
Elite *OsRDR3* allele enhances PHS resistance in rice. **A, B)** Germination performance **(A)** and germination rate **(B)** of seeds in freshly harvested panicles of NIP and JFZ (Shaxian). Scale bar, 1 cm. Data are mean ± s.e.m. (*n* = 10). Significant differences were calculated using two-tailed Student's *t*-tests. **P < 0.01. **C)** Frequency distribution of germination rate in RC-RILs. **D)** QTL analysis (*qPHS1a*, PVE: 3.59%; *qPHS1b*, PVE: 5.81%; *qPHS4a*, PVE: 4.04%; *qPHS7a*, PVE: 3.75%). **E)** Germination rates of individual plants from the RHL23, categorized by genotypes: *qPHS1a^NIP^*/*qPHS1a^NIP^*, *qPHS1a^NIP^*/*qPHS1a^JFZ^* and *qPHS1a^JFZ^*/*qPHS1a^JFZ^* (Sanya). Data are mean ± s.e.m. (*n* = 10). Different letters denote significant differences (*P* < 0.05) from a Duncan's multiple range test. **F)** Fine mapping of *qPHS1a*. Chromosomal segments homozygous for *qPHS1a^JFZ^* and *qPHS1a^NIP^* alleles are represented by filled and open bars, respectively (Sanya). Data are mean ± s.e.m (*n* = 6). **G)** The allelic variations in the coding region of *OsRDR3* between NIP and JFZ. **H)** Amino acid substitution and deletion of OsRDR3 protein from JFZ. In the diagram, the small box in the middle indicates the catalytic domain. **I)** Germination performance of seeds in freshly harvested mature panicles of wild type, 3 *osrdr3* mutant lines generated by CRISPR/Cas9 and transgenic *osrdr3-c1* plants carrying the *p35S::OsRDR3^NIP^* or *p35S::OsRDR3^JFZ^* constructs. Scale bar, 1 cm. **J)***OsRDR3* mutations generated via CRISPR/Cas9-based genome editing. The *osrdr3-c1* and *osrdr3-c2* possess a 33-bp deletion or a 30-bp deletion in the splice site of the intron 14/exon 15 junction, respectively. And both *osrdr3-c1* and *osrdr3-c2* produce the same alternatively spliced proteins, in which a frameshift causes a premature stop codon resulting in a truncated protein with 898 amino acids. The *osrdr3-c3* contains an insertion of a single adenine resulting in a frameshift mutation causing a premature protein with 885 amino acids. In the diagram, the small box in the middle indicates the catalytic domain. **K)** Germination rate of plants in I (Shaxian). Data are mean ± s.e.m. (*n* = 10). Different letters denote significant differences (*P* < 0.05) from a Duncan's multiple range test. **L)** Heatmap showing the log2 fold-change of relative expression levels of *OsGRF* genes in *osrdr3-c1* relative to the WT. Data used in heatmap were derived from 3 independent RNA-seq biological replicates. **M)** Relative transcript abundances of *OsGRF* genes. Data are mean ± s.e.m. (*n* = 3). Significant differences were calculated using two-tailed Student's *t*-tests. **P < 0.01, *P < 0.05, and n.s. is not significant. **N)** Heatmap shows the TPM (transcripts per million) ratios of miR396 in the *osrdr3-c1* relative to the NIP (WT), with the TPM values in WT set as 1. **O)** Germination performance of seeds in freshly harvested mature panicles of WYJ7, WYJ7-*osgrf4*, and transgenic WYJ7 plants carrying the *p35S::OsGRF4^ngr2^*-*GFP* constructs. Scale bar, 1 cm. **P)** Time-course germination percentage of seeds in freshly harvested mature panicles of WYJ7, WYJ7-*osgrf4*, and WYJ7 *p35S::OsGRF4^ngr2^-GFP*. **Q)** Distribution of germination rate among 98 rice varieties for the 2 *OsRDR3* haplotypes defined by a 139-bp deletion. The center line represents the average value. The upper and lower box limits indicate the medians of the data greater than or less than the average value, respectively. The ends of the whiskers represent the absolute maximum and minimum values. Significant differences were calculated using two-tailed Student's *t*-tests. **P < 0.01. **R)** Genotyping analysis of *OsRDR3*. **S)** Germination rate of seeds in freshly harvested panicles of plants showed in **R** (Shaxian). Data are mean ± s.e.m. (*n* = 10). Different letters denote significant differences (*P* < 0.05) from a Duncan's multiple range test. **T)** Plants of TaifengB, TaifengB-*OsRDR3^NIP^*, and TaifengB-*OsRDR3^NIP^*-*qPHS4a^NIP^*. Scale bar, 20 cm. **U)** The PHS phenotype of TaifengB, TaifengB-*OsRDR3^NIP^*, and TaifengB-*OsRDR3^NIP^*-*qPHS4a^NIP^* (observed in field condition after 3 consecutive days of rain in Shaxian, 2024). Scale bar, 1 cm. **V)** Germination rate of seeds in freshly harvested mature panicles of TaifengB, TaifengB-*OsRDR3^NIP^*, and TaifengB-*OsRDR3^NIP^*-*qPHS4a^NIP^* (Sanya, 2024, Shaxian, 2023 and 2024). Data are mean ± s.e.m. (*n* = 10). Different letters denote significant differences (*P* < 0.05) from a Duncan's multiple range test. **W)** The germination rate of seeds stored for 6 months after being harvested. Data are mean ± s.e.m. (*n* = 10). Different letters denote significant differences (*P* < 0.05) from a Duncan's multiple range test. Germination tests in **(A, B, I, K, O,** and **P)** were repeated with 10 panicle replicates. Photographs in (**A**, **I,** and **O**) were taken after 6-d imbibition. The arrowheads in **(A, I, O,** and **U)** indicated the sprouted seeds.

Fine mapping using 2,389 progenies from RHL23 narrowed down *qPHS1a* to a ∼20-kb region harboring a single gene, *RNA-DEPENDENT RNA POLYMERASE3* (*OsRDR3*; Fig. 1F). The RDR family is divided into α, β, and γ subtypes, with 5 RDRs identified in rice. OsRDR1 (Wang et al. 2016), OsRDR2 (Wang et al. 2022), and OsRDR6 (Wagh et al. 2016; Liu et al. 2020) belong to the α subtype; OsRDR3 and OsRDR4 belong to the γ-clade (Jha et al. 2021). Although the roles of RDRs in viral defense as well as rice development and growth have been investigated, their specific function in regulating PHS remains unclear. Sequence comparison revealed that the *OsRDR3^JFZ^* harbored multiple nucleotide substitutions and a 139-bp deletion spanning an exon-intron junction (Supplementary Fig. S2), leading to exon skipping and a C-terminal truncated protein with a premature stop codon (Fig. 1G and H). To validate whether *OsRDR3* is the causal gene at *qPHS1a*, we first examined the expression levels of *OsRDR3* in 2 RHL23 progeny plants: one harboring *qPHS1a^JFZ^* and the other *qPHS1a^NIP^*. Our analysis revealed no significant difference in *OsRDR3* expression level between the 2 plants (Supplementary Fig. S3). We next generated 3 *ososrdr3* mutants using CRISPR/Cas9 (Fig. 1I), which yielded C-terminally truncated OsRDR3 proteins (Fig. 1J). Although previously reported RNAi-mediated knockdown of *OsRDR3* severely affects rice plant growth and development (Jha et al. 2021), our CRISPR/Cas9-generated *osrdr3* mutations primarily accelerated the germination of freshly harvested seeds (Fig. 1K), with minimal impact on other aspects of rice plant growth and development (Supplementary Fig. S4A to F). This discrepancy may stem from the CRISPR target site lying downstream of the catalytic-domain-encoding region of *OsRDR3* (Fig. 1J), thus preserving most catalytic function, whereas RNAi-mediated knockdown reduces *OsRDR3* function more globally. Moreover, we found that the accelerated germination in *ososrdr3-c1* mutant was restored to the wild-type (NIP) level by introducing *OsRDR3^NIP^*, but not by *OsRDR3^JFZ^* (Fig. 1I and K). These results demonstrate that *OsRDR3* plays a previously uncharacterized role in seed germination, and that the elite *OsRDR3^NIP^* allele confers enhanced PHS resistance.

Although RDRs synthesize double-stranded RNAs, which are processed into small RNAs and mediate gene silencing, the absence of RDRs can result in complex downstream effects, including both upregulation and downregulation of target genes (Liu et al. 2020). To understand how *OsRDR3* regulates PHS, RNA-seq assays were performed using freshly harvested seeds of NIP and *osrdr3-c1* after 24 h of imbibition. The knockout of *OsRDR3* resulted in the upregulation of 1,622 genes and the downregulation of 1,562 genes (log2 fold-change > 0.5, *P* < 0.05; Supplementary Fig. S5A and Table S2). Among these differentially expressed genes, several were associated with ABA biosynthesis and signaling. For instance, the expression level of *β-OsLCY*, a gene involved in ABA biosynthesis, was reduced in *osrdr3-c1*, and this reduction was reported to an increased PHS (Supplementary Fig. S5B; Fang et al. 2008). Similarly, *OsPP2C51*, a positive regulator of seed germination acting downstream of the receptor in ABA signaling, showed increased expression in *osrdr3-c1* (Supplementary Fig. S5B; Bhatnagar et al. 2017). In addition, we identified several genes associated with seed germination from our RNA-seq data (Supplementary Fig. S5C). For example, *OsZDS*, which inhibits seed germination, was downregulated in *osrdr3-c1* (Supplementary Fig. S5C; Fang et al. 2008), while *SD6*, which promotes seed germination, was upregulated (Supplementary Fig. S5C; Xu et al. 2022). These genes are also linked to ABA biosynthesis and signaling. Reverse transcription quantitative polymerase chain reaction (RT-qPCR) assays further confirmed that these seed germination-related genes are regulated by *OsRDR3* (Supplementary Fig. S5D to K). Moreover, the endogenous ABA content was lower in *osrdr3-c1* grains compared to NIP (Supplementary Fig. S5L). These results indicated that *OsRDR3* regulates PHS via modulating the ABA biosynthesis and signaling pathway.

Notably, several *OsGRF* genes, including *OsGRF4*, *OsGRF5*, *OsGRF7*, and *OsGRF10*, were downregulated in the *osrdr3-c1* mutant (Fig. 1L). Growth-regulating factors (GRFs) are plant-specific transcription factors that play essential roles in controlling various developmental processes and responses to both biotic and abiotic stimuli (Piya et al. 2020). Many *GRF* genes contain a target site for microRNA miR396, which can suppress *GRF* expression in mature tissues (Debernardi et al. 2012). RT-qPCR assays of all the *OsGRF* genes showed that the mRNA abundances were reduced in the *osrdr3-c1* mutant, except for *OsGRF9* (Fig. 1M). Correspondingly, miRNA-seq assays showed that the abundance of miR396 was increased in the *osrdr3-c1* mutant (Fig. 1N; Supplementary Table S3). Our previous research results showed that increasing the expression of *OsGRF4* enhances nitrogen use efficiency (NUE) and grain yield (Li et al. 2018). However, the role of *OsGRF4* in PHS regulation remains unknown. Using wild-type plants WYJ7 (Wuyunjing 7, a *japonica* rice variety with the *OsRDR3^NIP^* allele), *osgrf4* mutant (WYJ7-*osgrf4*), and *OsGRF4* overexpression transgenic plant (WYJ7 *35S*::*OsGRF4^ngr2^*-*GFP*; Li et al. 2018), we observed that the *osgrf4* mutant showed faster germination in freshly collected panicles compared to WYJ7, whereas the overexpression of *OsGRF4* delayed germination (Fig. 1O and P). RT-qPCR assays revealed that seed germination-related genes regulated by *OsRDR3* were also influenced by *OsGRF4* (Supplementary Figs. S5D to K and S6A to H). These results indicate that *OsGRF4* is involved in *OsRDR3*-mediated PHS resistant regulation, and increased OsGRF4 activity simultaneously enhances both NUE and PHS resistance in rice.

In hybrid rice seed production, PHS can result in average yield losses of 10% to 20%, with losses reaching up to 50% in certain years (Hu et al. 2003). Genotyping analysis of 98 rice varieties revealed that the 139-bp deletion characteristic of *OsRDR3^JFZ^* is predominantly observed in *indica* varieties and is associated with reduced PHS resistance (Fig. 1Q; Supplementary Tables S4 and S5). Examination of *OsRDR3* genotypes in the maintainer lines of several prominent *indica* sterile lines crucial for hybrid rice breeding in China showed that nearly all carried the *OsRDR3^JFZ^* allele (Fig. 1R) and exhibited relatively lower PHS resistance (Fig. 1S). TaifengA and YexiangA are 2 dominant sterile lines in high-quality hybrid rice breeding in China. We introduced the *OsRDR3^NIP^* allele into the maintainer lines (TaifengB and YexiangB) through backcrossing (Fig. 1T; Supplementary Fig. S7A). Both TaifengB-*OsRDR3^NIP^* and YexiangB-*OsRDR3^NIP^* exhibited markedly enhanced resistance to PHS compared to TaifengB and YexiangB (Fig. 1U and V; Supplementary Fig. S7B and C). Importantly, the introduction of *OsRDR3^NIP^* improved PHS resistance without affecting the germination of stored seeds (Fig. 1W; Supplementary Fig. S7D) or other agronomic traits related to yield and grain shape (Supplementary Figs. S7E to K and S8A to H). As mentioned above, the *qPHS4a^NIP^* allele from NIP also contributes to improving resistance to PHS (Fig. 1D). By introducing *qPHS4a^NIP^* into TaifengB-*OsRDR3^NIP^*, the PHS resistance of TaifengB was further enhanced (Fig. 1T to W).

In summary, our work suggests that *OsRDR3* regulates PHS resistance partly by modulating ABA biosynthesis and signaling pathway. Additionally, *OsGRF4*, a positive regulator of NUE and grain yield (Li et al. 2018), is identified as a target of *OsRDR3* that also represses PHS. We also propose a breeding strategy utilizing the elite *OsRDR3^NIP^* allele in *indica* hybrid rice parents to reduce PHS risk, thereby enhancing the safety of hybrid rice seed production.

## Supplementary Material

kiaf282_Supplementary_Data

## Data Availability

The data underlying this article are available in the article and in its online supplementary material.

## Supplementary data

The following materials are available in the online version of this article.

**Supplementary Figure S1.** Flowchart of building the RC_4_F_6_ population for QTL analysis.

**Supplementary Figure S2.** Sequence variations between JFZ and NIP at *OsRDR3.*

**Supplementary Figure S3.** The relative expression levels of *OsRDR3*.

**Supplementary Figure S4.** Characterization of NIP and *osrdr3-c1*.

**Supplementary Figure S5.**  *OsRDR3* regulates PHS via modulating the ABA-related pathway.

**Supplementary Figure S6.** Seed-germination-related genes were regulated by *OsGRF4*.

**Supplementary Figure S7.** The *OsRDR3^NIP^* allele enhances PHS resistance in YexiangB.

**Supplementary Figure S8.** Characterization of TaifengB and TaifengB-*OsRDR3^NIP^*.

**Supplementary Table S1.** Linkage analysis between INDEL markers and candidate QTLs of PHS.

**Supplementary Table S2.** RNA-Seq gene expression levels and differential expression analysis.

**Supplementary Table S3.** miRNA expression profile of NIP and *rdr3-c1*.

**Supplementary Table S4.** Germination rates of 98 varieties selected from 3,000 Rice Genomes Project.

**Supplementary Table S5.** The primer sequences used for map-based cloning, genotyping, transgene constructs and RT-qPCR assays.

## Dive Curated Terms

The following phenotypic, genotypic, and functional terms are of significance to the work described in this paper:

OsRDR1 Gramene: Os02g0736200
OsRDR1 Araport: Os02g0736200

OsRDR2 Gramene: Os04g0465700
OsRDR2 Araport: Os04g0465700

OsRDR6 Gramene: Os01g0527600
OsRDR6 Araport: Os01g0527600

OsRDR3 Gramene: Os01g0197900
OsRDR3 Araport: Os01g0197900

OsRDR4 Gramene: Os01g0198000
OsRDR4 Araport: Os01g0198000
