# Supplementary material for: An elite RNA-DEPENDENT RNA POLYMERASE3 allele enhances preharvest sprouting resistance in rice
Source: Plant Physiol. 2025 Aug 22;198(4):kiaf282. doi: 10.1093/plphys/kiaf282 (PMC12371833; doi:10.1093/plphys/kiaf282)
Supplement: kiaf282_Supplementary_Data [file kiaf282_supplementary_data.pdf]

An elite *RNA-DEPENDENT RNA POLYMERASE3* allele enhances pre-harvest sprouting resistance in rice

Huazhong Guan[1, 2, †], Zhiwei Chen[1, 2, †], Shuoxun Wang[3, †], Bo Jiang[1, 2], Jinxiang Zhao[1, 2], Yijin Gao[1, 2], Suming Zhu[1, 2], Shiying Li[1, 2], Damei Mao[1, 2], Lu Lin[1, 2], Weishu Fan[3], Qiang Liu[3], Wenzhen Song[3], Likun Huang[1, 2], Xiaofang Xie[1, 2], Chuanlong Wan[1, 2], Yafeng Ye[6], Shujing Cheng[3], Jinfang Chu[3, 5], Xiangdong Fu[3, 4, 5, *], Weiren Wu[1, 2, *], Kun Wu[3, *]

[1]Fujian Provincial Key Laboratory of Crop Breeding by Design, Fujian Agriculture and Forestry University, Fuzhou 350002, Fujian, China

[2]Key Laboratory of Genetics, Breeding and Multiple Utilization of Crops, Ministry of Education, Fujian Agriculture and Forestry University, Fuzhou 350002, Fujian, China

[3]State Key Laboratory of Seed Innovation, Institute of Genetics and Developmental Biology, Chinese Academy of Sciences, Beijing 100101, China

[4]New Cornerstone Science Laboratory, Institute of Genetics and Developmental Biology, Chinese Academy of Sciences, Beijing 100101, China

[5]College of Life Sciences, University of Chinese Academy of Sciences, Beijing 100049, China

[6]Key Laboratory of High Magnetic Field and Ion Beam Physical Biology, Hefei Institutes of Physical Science, Chinese Academy of Sciences, Hefei 230031, China

[*]Correspondence: (Tel 86-010-64806557; email kunwu@genetics.ac.cn (K.W.); Tel 0591-83789176 email wuwr@fafu.edu.cn (W.W.); Tel 86-010-64806558; email xdfu@genetics.ac.cn (X.F.))

[†] These authors contributed equally to this work.

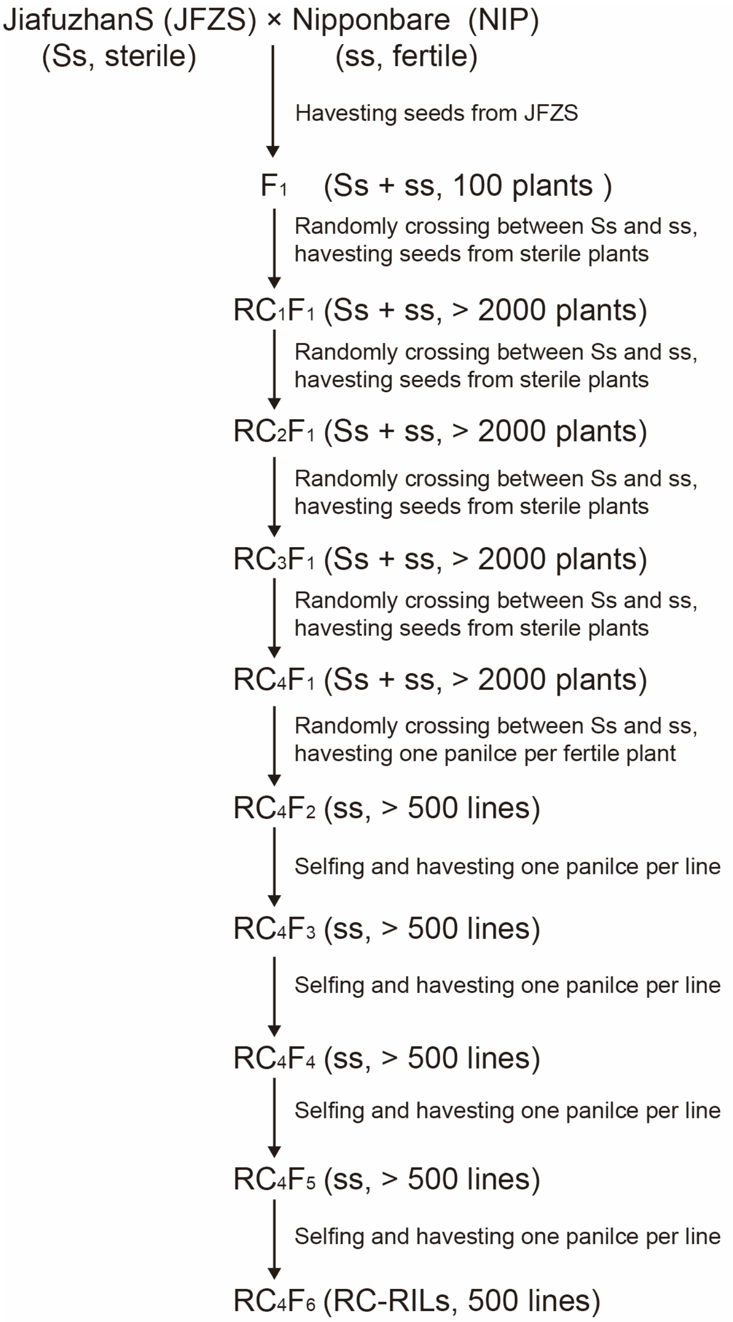

Supplementary Figure S1. Flowchart of building RC_4F_6 population for QTL analysis.

JiafuzhanS (JFZS), the dominant nuclear male sterility line with the genetic background of Jiafuzhan (JFZ), which carries a dominant nuclear sterility gene in a heterozygous genotype at this locus (Ss), was crossed with the Nipponbare (NIP) to obtain F_1 seeds. These F_1 seeds were grown under isolated conditions, resulting in a population consisting of approximately 50% fertile plants and 50% sterile plants. Fertile plants pollinated the sterile plants, and seeds were collected from the sterile plants. Over four generations, more than 2,000 plants were grown in each generation. Starting from the fourth generation, panicles from fertile plants were collected and grown as individual lines. After four generations of selfing, a population of 500 random crossing-recombinant inbred lines (RC-RILs) was established.

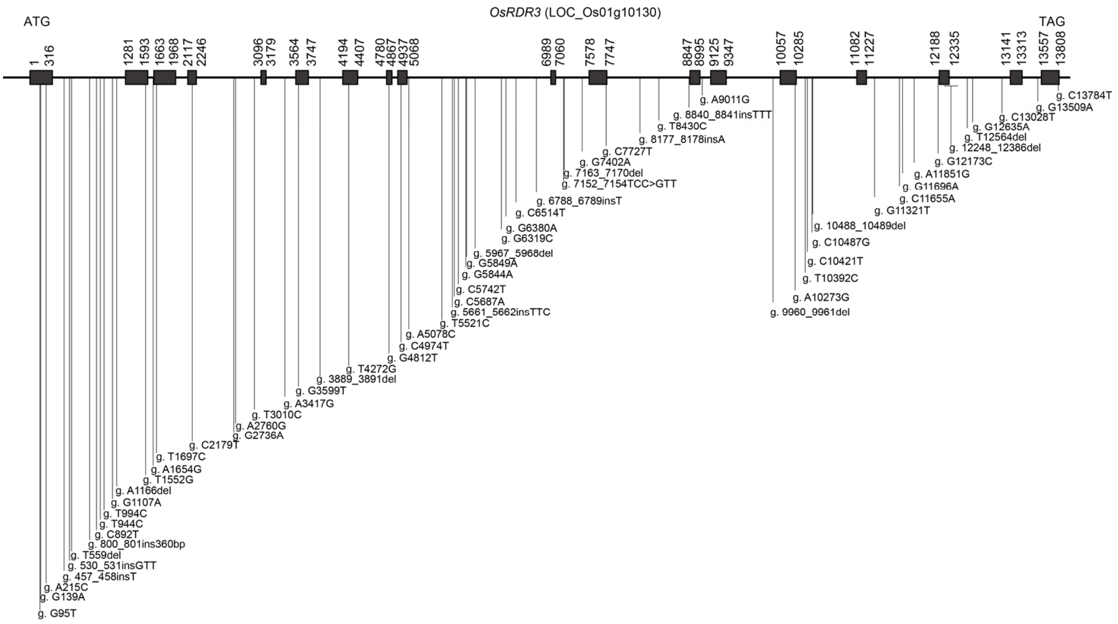

Supplementary Figure S2. Sequence variations between JFZ and NIP at *OsRDR3*.

The sequence comparison at the *OsRDR3* locus shows the substitutions, insertions and deletions between the Jiafuzhan (JFZ) and Nipponbare (NIP) genomes. The DNA sequence of NIP was set as the reference. Black boxes indicate the exons of *OsRDR3*.

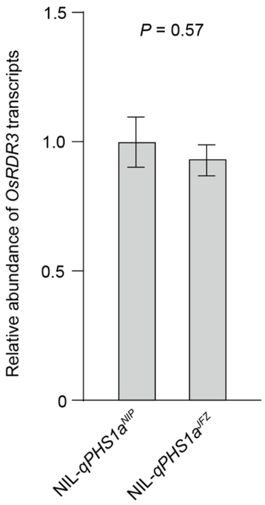

Supplementary Figure S3. The relative expression levels of *OsRDR3*.

The relative expression levels of *OsRDR3* in two near-isogenic lines (NILs) from the residual heterozygous line 23 (RHL23) progeny, with one harboring $qPHS1a^{JFZ}$ and the other $qPHS1a^{NIP}$. Data are mean ± s.e.m. (n = 3). Significant difference was calculated using two-tailed Student's t-tests.

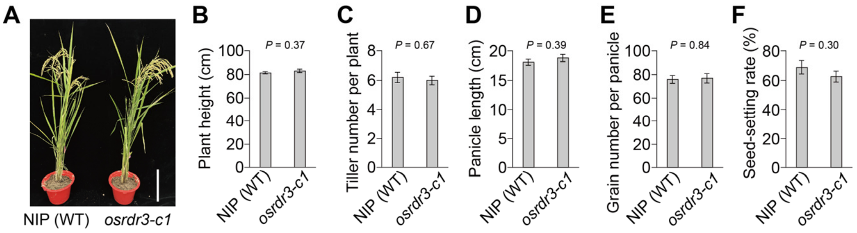

Supplementary Figure S4. Characterization of NIP and *osrdr3-c1*.

A, Plants of wild type NIP and *osrdr3-c1*. Scale bar, 20 cm. **B**, Plant height. **C**, Tiller number per plant. **D**, Panicle length. **E**, Grain number per panicle. **F**, Seed-setting rate. **B-F**, Data are mean ± s.e.m. (n = 10). Significant differences were calculated using two-tailed Student's t-tests. n.s. is not significant.

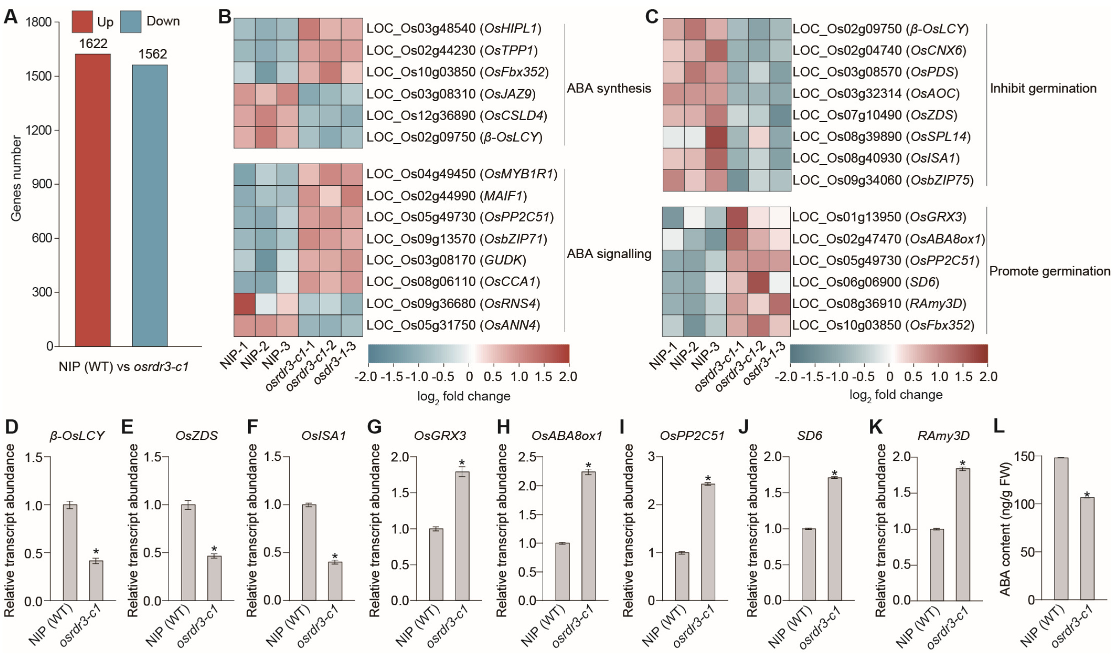

Supplementary Figure S5. *OsRDR3* regulates PHS via modulating the ABA-related pathway.

A, Up-and downregulated genes in *osrdr3-c1* relative to the NIP (WT) using 24-hour imbibed seeds of NIP and *osrdr3-c1*. **B**, Relative gene expression of ABA-related genes. **C**, Relative gene expression of seed-germination-related genes. The heatmaps in **B** and **C** show the normalized changes in gene expression levels of ABA-related and seed-germination-related genes in NIP and *osrdr3-c1* on a row-normalized scale. Gene clusters are generated using hierarchical clustering. **D-K**, Transcript abundances of genes that inhibit germination (**D-F**) and promote germination (**G-K**). Data are mean ± s.e.m. (n = 3). **L**, ABA contents in fresh seeds of WT and *osrdr3*. Values are means ± s.e.m. (n = 3). Significant differences were calculated using two-tailed Student's t-tests. *$P < 0.05$.

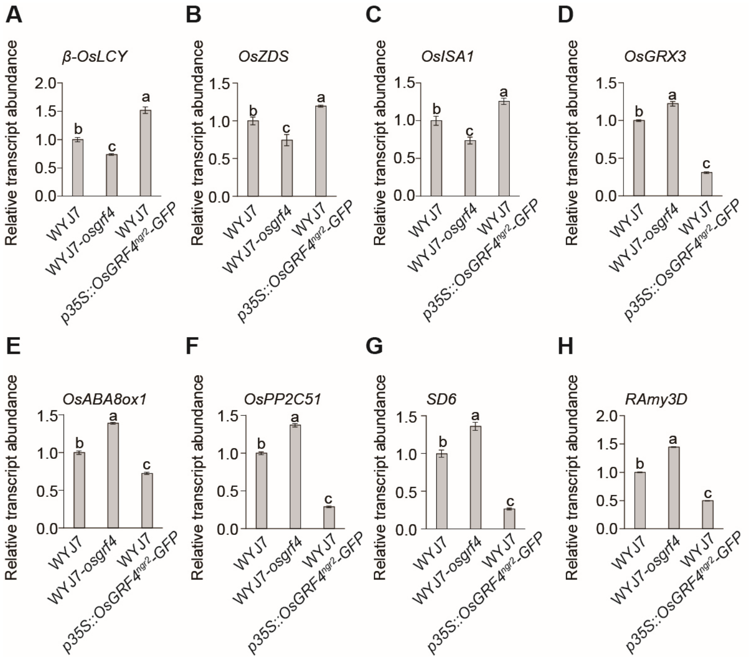

Supplementary Figure S6. Seed-germination-related genes were regulated by *OsGRF4*.

A-H, Transcript abundances of genes that inhibit germination (**A-C**) and promote germination (**D-H**) in WYJ7, WYJ7-*osgrf4*, and WYJ7 *p35S::OsGRF4*^{ngr2}*-GFP*. Data are mean ± s.e.m. (n = 3). Different letters denote significant differences ($P < 0.05$) from a Duncan's multiple range test.

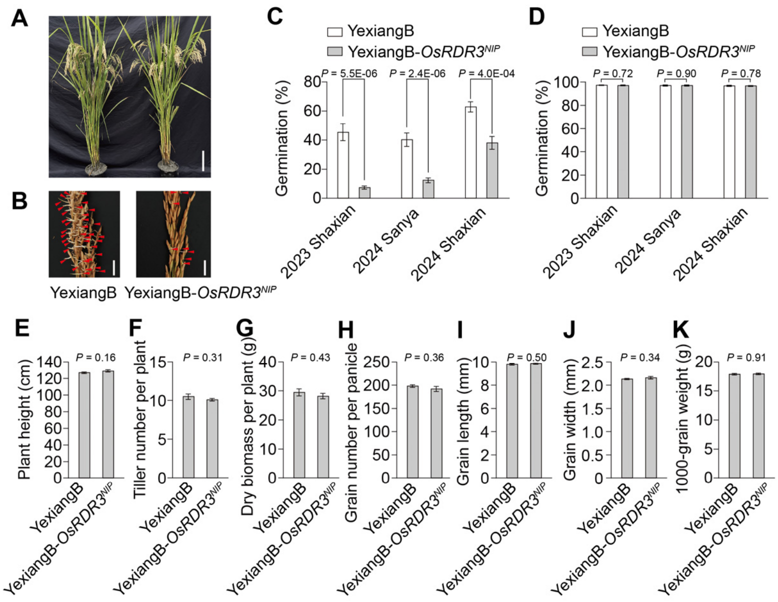

Supplementary Figure S7. The *OsRDR3*^{NIP} allele enhances PHS resistance in YexiangB.

A, Plants of YexiangB and YexiangB-*OsRDR3*^{NIP}. Scale bar, 20 cm. **B**, Germination performance of seeds in freshly harvested mature panicles of YexiangB and YexiangB-*OsRDR3*^{NIP} (harvested in Shaxian, 2024). Scale bar, 2 cm. The red arrowheads indicated the sprouted seeds. **C**, Germination rate of seeds in freshly harvested mature panicles of YexiangB and YexiangB-*OsRDR3*^{NIP} (harvested in Sanya, 2024 and in Shaxian, 2023 and 2024). **D**, The germination rate of seeds stored for 6 months after being harvested. **E**, Plant height. **F**, Tiller number per plant. **G**, Dry biomass per plant. **H**, Grain number per panicle. **I**, Grain length. **J**, Grain width. **K**, 1000-grain weight. **C-K**, Data are mean ± s.e.m. (n = 10). Significant differences were calculated using two-tailed Student's t-tests. n.s. is not significant.

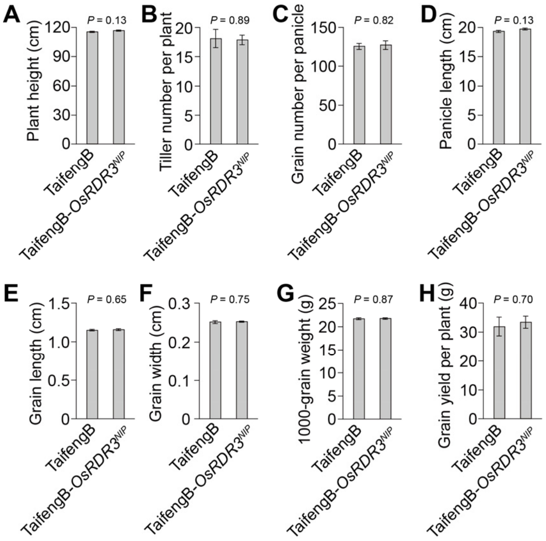

Supplementary Figure S8. Characterization of TaifengB and TaifengB-*OsRDR3*^{NIP}.

A, Plant height. **B**, Tiller number per plant. **C**, Grain number per panicle. **D**, Panicle length. **E**, Grain length. **F**, Grain width. **G**, 1000-grain weight. **H**, Grain yield per plant. **A-H**, Data are mean ± s.e.m. (n = 10). Significant differences were calculated using two-tailed Student's t-tests.

Supplementary Table S1. Linkage analysis between INDEL markers and Candidate QTLs of PHS.

Marker	Chromesome	Locus	Position	*p* value	Type
Indel1a8	1	*qPHS1a*	4814665	4.95E-03	positive
Indel1a5	1	*qPHS1a*	5413105	6.08E-05	positive
Ind1a10	1	*qPHS1a*	5642823	2.47E-02	positive
Indel1b5	1	*qPHS1b*	31792808	2.72E-05	positive
Indel1b7	1	*qPHS1b*	31799047	2.35E-06	positive
Indel2a4	2	*qPHS2a*	18946385	8.58E-01	false positive
Indel2a11	2	*qPHS2a*	19072458	5.10E-01	false positive
Indel2a13	2	*qPHS2a*	19284338	1.30E-01	false positive
Ind4a7	4	*qPHS4a*	22290945	1.10E-03	positive
Ind4a9	4	*qPHS4a*	23197669	1.34E-05	positive
Ind4a15	4	*qPHS4a*	23545096	1.23E-03	positive
Ind7a1	7	*qPHS7a*	23746285	1.42E-04	positive
Ind7a6	7	*qPHS7a*	23971735	4.02E-04	positive

Supplementary Table S2. RNA-Seq gene expression levels and differential expression analysis.

Gene ID	NIP-1 (FKPM)	NIP-2 (FKPM)	NIP-3 (FKPM)	*rdr3-c1-1* (FKPM)	*rdr3-c1-2* (FKPM)	*rdr3-c1-3* (FKPM)	log2 fold-Change	*p* value
LOC_Os10g20480	31.798	32.542	32.952	0	0	0	-14.70785384	3.95E-29
MSTRG.4037	0	0	0	24.368	24.699	24.186	13.17212213	6.06E-24
LOC_Os10g16440	23.219	22.177	22.396	0	0	0.022	-11.13205364	5.91E-22
MSTRG.5492	0	0	0	9.735	8.951	8.321	10.499636	1.26E-15
LOC_Os06g16040	37.247	36.74	34.838	0.027	0.027	0.075	-9.503593364	2.16E-49
LOC_Os10g22394	0	0	0	1.714	1.795	1.787	9.28312923	2.09E-12
MSTRG.3949	7.249	5.968	6.709	0	0	0.026	-8.989516051	5.41E-15
LOC_Os11g09460	0	0	0	4.617	3.353	3.956	8.946165191	1.53E-11
LOC_Os11g01690	0	0	0	5.517	4.149	3.91	8.916841018	1.84E-11
LOC_Os03g02470	0.252	0.298	0.241	93.495	94.905	81.538	8.512393426	4.47E-141
LOC_Os11g09979	0.304	0.501	0.218	109.203	109.853	114.866	8.461833857	7.96E-255
LOC_Os06g38150	0	0	0	6.206	3.932	5.802	8.222253291	7.91E-10
LOC_Os11g14810	0	0	0	1.423	2.774	1.228	8.139578111	1.57E-09
LOC_Os11g09720	0	0	0	0.721	0.422	0.493	7.198732712	0.00000011
LOC_Os07g01960	0	0	0	2.924	2.496	2.733	6.998319857	0.000000238
LOC_Os07g46130	0.242	0.42	0.454	0	0	0	-6.674813401	0.00000104
LOC_Os02g26870	0	0	0	0.768	0.633	0.673	6.583530131	0.00000127
LOC_Os02g44080	0.902	0.473	0.827	0	0	0	-6.371326713	0.00000341
LOC_Os11g09200	0.055	0	0	2.479	1.39	1.923	6.279805188	0.000000047
LOC_Os02g08330	5.298	5.408	4.636	0.026	0.052	0.097	-6.228971205	1.5E-28
LOC_Os12g11710	0.099	0.098	0	5.803	4.147	5.425	6.099256874	1.58E-09
LOC_Os11g09260	0	0	0	0.328	0.333	0.307	6.000759106	0.0000115
LOC_Os07g01970	0.07	0	0	1.615	2.592	1.636	5.897135395	0.00000029
MSTRG.14929	0	0.059	0.11	2.773	3.981	3.672	5.759149744	1.08E-11
LOC_Os01g51180	0	0	0	0.155	0.031	0.133	5.73172463	0.0000396
LOC_Os02g53240	0	0	0	1.233	1.25	1.153	5.552660011	0.000049
LOC_Os11g45190	0	0	0.013	0.351	0.308	0.243	5.425563139	0.00000226
LOC_Os11g09510	0	0	0	0.483	0.178	0.451	5.424085231	0.0000826
LOC_Os11g01700	0.047	0.075	0.003	2.146	2.045	1.737	5.383873864	9.75E-26
LOC_Os11g09230	0	0	0	0.238	0.345	0.223	5.271832278	0.000113037
LOC_Os11g45180	0.019	0.093	0.104	2.44	2.574	2.846	5.200993037	8.93E-31
LOC_Os03g18779	0.771	0.808	0.748	28.205	26.815	25.428	5.199741233	4.12E-124

LOC_Os11g09590	11.791	11.759	12.678	0.259	0.526	0.139	-5.170843012	1.96E-60
LOC_Os11g07480	0.271	0.269	0.28	0	0	0	-5.164844667	0.000143164
LOC_Os04g26330	0	0	0	0.342	2.265	0.482	4.850148389	0.00040939
LOC_Os04g32610	0	0	0	0.517	0.455	0.375	4.798762209	0.000360912
LOC_Os05g39540	0	0	0.121	0.859	1.114	1.575	4.773043243	3.89E-08
LOC_Os07g08940	0	0	0	0.255	0.712	0.298	4.764070476	0.000428804
LOC_Os07g13390	0.013	0	0	0.329	0.217	0.338	4.763109623	0.00000309
LOC_Os06g30090	0.132	0	0.121	2.1	2.322	2.796	4.667678463	1.07E-09
LOC_Os03g07950	0	0	0.058	0.855	1.115	0.628	4.665738984	0.0000436
MSTRG.8798	0	0	0	0.931	0.708	0.544	4.654512813	0.000499114
LOC_Os11g09190	0	0	0	0.248	0.335	0.193	4.653375679	0.000500124
LOC_Os11g45050	0.029	0.149	0.11	2.175	3	2.185	4.610431059	1.36E-30
LOC_Os06g43150	0	0	0	0.112	0.043	0.105	4.602815898	0.000588227
LOC_Os09g08510	0	0	0	0.203	0.271	0.2	4.488358102	0.000689381
LOC_Os11g45090	0	0	0	0.1	0.183	0.075	4.477243469	0.000738472
MSTRG.6602	0	0.365	0.118	5.023	2.427	3.847	4.454246655	0.000000012
LOC_Os07g11440	1.301	1.002	0.486	0.046	0	0.043	-4.269540343	0.0000304
LOC_Os07g31140	0	0.047	0	0.411	0.648	0.384	4.075227985	0.000266868
LOC_Os11g44960	0.054	0	0.033	0.503	0.669	0.421	4.074407885	1.14E-08
MSTRG.6104	0.254	0.353	0.047	3.928	3.633	3.259	4.069399907	2.6E-18
LOC_Os05g12630	1.603	1.332	1.896	0.05	0.152	0.047	-3.918320183	3.32E-08
LOC_Os11g20330	11.352	12.386	14.993	0.782	0.991	0.609	-3.883204238	6.36E-41
LOC_Os04g18770	0	0.05	0.023	0.436	0.467	0.26	3.842968527	0.000000012
LOC_Os02g41961	0	0	0.587	3.913	4.279	5.487	3.836112416	0.00019199
MSTRG.12933	0	0	0.077	0.488	0.447	1.292	3.807997096	0.000613724
LOC_Os10g29400	0.025	0	0.069	0.55	0.574	0.437	3.786031315	0.00000151
LOC_Os06g40240	0.23	0.104	0.173	2.009	2.364	2.124	3.751251101	4.95E-49
LOC_Os02g07654	0	0	0.067	0.779	0.574	0.464	3.638220368	0.00084091
LOC_Os05g34490	0.102	0.034	0	0.628	0.456	0.855	3.612391776	0.00000515
LOC_Os11g09830	0	0.165	0	0.764	0.733	0.902	3.590974763	0.00000602
LOC_Os03g54804	0.149	0.247	0.105	0	0.017	0	-3.528495222	0.000418413
LOC_Os07g02430	0.449	0.405	0.337	4.451	4.671	4.05	3.51551613	7.59E-27
LOC_Os04g24340	0.536	0.531	0.344	0.052	0	0	-3.499555506	0.001052759
LOC_Os06g37290	0	0.014	0.064	0.376	0.286	0.339	3.444784285	0.000000495
LOC_Os04g18780	0	0.027	0	0.265	0.042	0.289	3.410953927	0.001491788

LOC_Os05g01010	0.227	0.601	0.174	3.945	3.179	2.847	3.39394141	1.02E-20
LOC_Os09g34950	1.987	2.019	2.149	0.049	0.149	0.276	-3.385928836	5.05E-10
LOC_Os03g25500	0.064	0	0	0.248	0.627	0.579	3.381092292	0.001447722
LOC_Os02g35650	2.057	1.614	1.965	0.248	0	0.155	-3.329771474	0.00000397
LOC_Os11g12010	2.098	1.786	2.254	19.101	17.982	18.064	3.2580258	2.01E-120
LOC_Os04g03830	0.053	0.026	0.024	0.28	0.309	0.666	3.249581391	0.0000464
MSTRG.12815	3.704	4.47	3.025	32.221	34.963	30.366	3.218085381	1.33E-99
LOC_Os10g11370	0.489	0.485	0.299	0.052	0	0	-3.199395956	0.00192576
MSTRG.1145	1.648	1.633	1.395	12.231	15.806	12.292	3.182644925	1.53E-45
LOC_Os04g06900	0.027	0	0	0.155	0.157	0.242	3.171156185	0.002093789
LOC_Os09g37350	0	0	0.474	1.326	4.697	2.571	3.164512442	0.001184079
LOC_Os08g07900	0.021	0	0	0.101	0.123	0.207	3.148386257	0.002206769
LOC_Os08g10244	0.043	0.068	0.031	0.399	0.499	0.486	3.125838862	1.84E-12
LOC_Os08g11200	0.011	0	0	0.105	0.092	0.108	3.118419101	0.001302117
LOC_Os01g24560	0.084	0	0	0.486	0.287	0.414	3.107243188	0.00136654
LOC_Os06g15750	0.018	0.053	0.049	0.361	0.488	0.321	3.071840641	0.00000188
LOC_Os08g05690	0	0.015	0.029	0.271	0.153	0.127	3.063501737	0.000414713
LOC_Os01g07040	1.344	1.617	0.176	0.093	0.094	0	-3.043473457	0.001817482
LOC_Os07g09760	0.126	0.169	0.111	1.201	1.158	0.975	3.023618305	9.06E-22
LOC_Os12g18360	0.065	0.016	0.112	0.62	0.563	0.476	2.988649087	5.8E-09
LOC_Os10g14170	0.972	1.489	2.121	11.802	11.329	12.477	2.980850488	7.95E-33
LOC_Os07g06834	0.14	0.139	0.172	1.264	1.281	1.266	2.944958597	0.00000013
LOC_Os01g35330	0.441	0.273	0.809	3.407	4.64	3.683	2.937395779	1.04E-15
LOC_Os12g12600	3.747	3.452	2.652	0.19	0.514	0.415	-2.886687703	5.94E-11
LOC_Os10g10620	1.241	1.085	0.87	0.211	0	0.066	-2.870528555	0.000216939
LOC_Os06g23860	0	0	0.025	0.077	0.092	0.166	2.842921392	0.002729416
LOC_Os11g40340	0.056	0.093	0.12	0.723	0.623	0.626	2.78699028	6.51E-09
LOC_Os11g15060	0.311	0.103	0.142	0.899	1.164	2.101	2.754806367	0.00000113
LOC_Os01g50032	0.73	0.93	0.815	0.129	0	0.102	-2.750877639	0.00000789
LOC_Os11g44990	0.128	0	0.03	0.478	0.273	0.589	2.749195126	0.00000795
LOC_Os01g50020	0	0.062	0.057	0.451	0.335	0.338	2.732458494	0.000422003
MSTRG.2032	0	0.172	0	0.838	1.019	1.254	2.724962859	0.004129078
LOC_Os10g38730	0.958	0.61	1.192	0.066	0.067	0.123	-2.724145065	0.000399255
LOC_Os11g47390	0.315	0.338	0.168	1.898	1.745	1.609	2.698665268	1.18E-14
LOC_Os02g21320	0.025	0.148	0.068	0.527	0.656	0.538	2.653801919	0.00000355

LOC_Os06g49350	0.923	0.994	1.065	0.114	0.202	0.117	-2.600890266	1.06E-20
LOC_Os10g31930	1.083	2.569	1.876	0.405	0.171	0.13	-2.586208311	1.62E-09
LOC_Os08g26880	3.333	3.97	4.361	22.363	19.847	22.73	2.56790019	1.46E-92
LOC_Os03g50420	0.725	1.015	0.822	0.082	0.083	0.154	-2.520250129	0.0000353
LOC_Os12g24050	0.408	0.404	0.385	2.015	2.336	2.155	2.51320222	2.96E-34
LOC_Os11g43990	0.247	0.398	0.312	1.76	1.603	1.896	2.458329482	5.67E-12
LOC_Os05g50390	0.398	0.46	0.912	3.328	3.049	3.53	2.448187868	1.93E-10
LOC_Os10g03360	0.138	0.183	0.042	0.578	1.127	0.707	2.446900241	0.0000802
LOC_Os01g27140	0.076	0	0.139	0.587	0.743	0.653	2.437171058	0.002470339
LOC_Os11g15030	0.05	0.05	0	0.532	0.245	0.317	2.427980843	0.005277691
LOC_Os07g48680	0.785	0.389	0.42	0.063	0.064	0	-2.423048604	0.005205011
LOC_Os04g20090	0.145	0.168	0	0.704	0.655	0.472	2.422613266	0.00000607
LOC_Os04g07890	0	0.119	0.028	0.29	0.529	0.298	2.419701053	0.000730049
LOC_Os10g10030	0.019	0.038	0	0.26	0.169	0.087	2.375980301	0.00290523
LOC_Os12g36840	2.203	2.046	1.767	0.399	0.337	0.186	-2.366630068	0.00000189
LOC_Os05g12640	65.321	68.23	58.956	9.955	11.607	13.022	-2.361632325	6.53E-151
LOC_Os04g50930	0	0.085	0	0.248	0.195	0.309	2.339719614	0.00316017
LOC_Os06g04410	0.056	0.167	0.052	0.868	0.495	0.609	2.336637677	0.000869136
LOC_Os01g39850	0.169	0.067	0.062	0.588	0.695	0.519	2.328489544	0.0000773
LOC_Os01g07030	1.438	1.781	1.78	0.069	0.281	0.389	-2.321773438	0.0000236
LOC_Os10g17660	2.113	1.57	0.969	0.127	0.323	0.238	-2.320107851	0.0000286
LOC_Os03g37290	0.491	0.486	0.365	0.059	0.06	0.055	-2.319456365	0.000415979
LOC_Os05g15850	0.254	0.302	0.233	0.98	1.093	2.154	2.278876251	0.00000448
LOC_Os01g07050	2.302	2.281	1.535	0.202	0.512	0.189	-2.278456265	0.0000958
LOC_Os01g06990	5.828	6.908	3.817	1.205	0.305	1.314	-2.27595915	0.000000013
LOC_Os01g72140	0.797	0.743	0.902	0.136	0.092	0.127	-2.255893296	0.000170476
LOC_Os07g35700	0.783	0.659	0.807	0.191	0	0.179	-2.238281957	0.00000597
LOC_Os10g29390	0.228	0.226	0.084	0.924	1.026	0.946	2.237160788	0.0000261
LOC_Os08g28880	2.009	1.99	1.332	0.371	0.251	0.308	-2.236777041	7.55E-08
LOC_Os12g30420	0.329	0.958	0.136	3.25	2.792	2.417	2.217275645	0.00000683
LOC_Os05g05680	6.306	7.102	6.957	1.336	1.35	1.267	-2.205291367	4.05E-25
LOC_Os07g46930	0.928	0.726	0.851	0.141	0.191	0.044	-2.202249029	0.000243952
LOC_Os11g09350	1.79	2.122	1.407	7.804	8.16	6.921	2.185438915	3.13E-35
LOC_Os07g19410	0.755	0.457	0.346	0.081	0	0.113	-2.180009922	0.0015281
LOC_Os07g37730	4.104	4.066	2.508	0.646	0.569	0.866	-2.178245074	3.76E-16

LOC_Os08g11190	0.028	0.055	0.051	0.296	0.354	0.251	2.174751627	0.001448535
LOC_Os12g41600	0.578	0.394	0.763	0.076	0	0.163	-2.169881424	0.000630088
LOC_Os11g30020	0.013	0.014	0.059	0.186	0.255	0.185	2.147849415	0.000162639
LOC_Os02g14600	1.919	2.489	2.24	10.1	9.145	8.592	2.145479489	4.78E-39
LOC_Os01g12570	21.951	21.465	18.381	3.743	4.532	4.601	-2.141996522	1.23E-67
LOC_Os07g36740	0.415	0.498	0.702	2.405	2.245	2.268	2.135387428	2.96E-17
LOC_Os04g47320	0.473	0.552	0.712	0.098	0.066	0.152	-2.129673607	0.00000162
LOC_Os11g34610	0.697	0.978	0.746	3.643	3.692	3.196	2.123286573	4.66E-11
LOC_Os05g13380	0	0.115	0.097	0.474	0.212	0.696	2.107185793	0.000726969
LOC_Os03g25490	0.568	0.462	0.428	1.934	1.994	2.317	2.106135482	3.08E-11
LOC_Os01g18860	6.695	6.213	7.081	1.504	1.42	1.374	-2.077311286	1.23E-26
MSTRG.12524	0.522	0.172	0.878	1.973	2.552	2.315	2.067384423	2.16E-08
LOC_Os10g32630	0.024	0	0.087	0.046	0.603	0.192	2.066719636	0.003303828
LOC_Os10g14180	8.793	8.35	9.416	30.403	31.835	31.607	2.05659294	0
LOC_Os11g13980	0.401	0.398	0.368	2.064	1.569	1.689	2.053097281	0.00000787
MSTRG.19922	0.75	0.55	0.636	2.223	2.47	3.105	2.046436256	1.37E-14
LOC_Os09g27350	0.118	0.06	0.073	0.374	0.536	0.44	2.04407991	0.0000214
LOC_Os08g23790	0.924	0.722	0.462	2.486	3.095	2.83	2.040626006	4.8E-15
LOC_Os01g28744	0.523	0.734	0.543	2.287	2.785	2.873	2.024478194	0.000000418
MSTRG.2770	0.325	0.322	0.298	1.253	1.778	1.347	2.023374965	0.0000367
LOC_Os02g42430	0.423	0.419	0.31	1.714	1.324	2.366	2.021941841	0.0000655
LOC_Os11g34624	0.261	0.355	0.478	1.415	1.594	1.529	2.010251766	1.89E-08
LOC_Os11g20160	4.972	4.31	5.162	0.776	1.18	1.253	-2.008608501	7.78E-18
LOC_Os10g39160	6.649	8.462	6.376	1.648	1.312	1.816	-1.996993686	6.43E-16
LOC_Os03g08490	14.425	18.801	16.188	3.819	3.515	4.063	-1.987187127	1.54E-40
LOC_Os05g03400	0.25	0.183	0.209	1.059	0.745	0.89	1.986496633	1.65E-08
LOC_Os05g41990	3.804	2.964	2.743	1.031	0.669	0.424	-1.955767994	4.11E-10
LOC_Os03g25330	0.634	0.982	0.981	0.191	0.233	0.071	-1.944092638	0.000146375
LOC_Os04g36610	2.175	1.847	1.211	0.187	0.646	0.28	-1.943920794	0.00000121
LOC_Os09g10340	0.519	0.545	0.812	0.206	0.03	0.11	-1.931814316	0.000268678
LOC_Os03g08290	0.578	0.562	0.734	0.176	0	0.112	-1.916730141	0.000799387
LOC_Os08g29980	0.255	0.241	0.276	0.871	0.815	1.159	1.906717876	4.01E-12
LOC_Os01g50680	0.321	0.637	0.36	0.034	0.07	0.097	-1.90623221	0.002212436
LOC_Os08g40740	0.137	0.068	0	0.463	0.469	0.618	1.904691645	0.00647702
LOC_Os11g10490	0.078	0.207	0.262	1.027	0.538	0.672	1.893630907	0.000022

LOC_Os05g15920	10.438	7.323	8.975	34.448	26.468	32.34	1.892721432	6.58E-51
LOC_Os05g45870	0.591	0.53	0.439	0.109	0.055	0.127	-1.891313331	0.000374309
LOC_Os07g30090	10.492	9.996	8.387	2.141	2.433	2.426	-1.886908955	2.88E-18
LOC_Os04g22090	0.125	0.096	0.203	0.467	0.636	0.549	1.886393245	0.000000627
MSTRG.24594	4.326	6.514	1.964	13.707	12.465	22.418	1.881765811	2.18E-09
LOC_Os09g36740	5.924	4.876	5.188	0.796	1.729	1.382	-1.881326867	4.28E-19
LOC_Os11g19880	0.516	0.511	0.384	0.031	0.095	0.116	-1.876593624	0.001174734
LOC_Os11g28104	0.707	0.501	0.486	2.047	2.248	1.8	1.872118374	6.09E-12
LOC_Os12g14699	2.077	2.035	1.773	7.256	6.491	6.547	1.86987021	8.9E-36
LOC_Os04g39670	2.181	2.826	2.615	0.378	0.601	0.757	-1.862129977	0.000000376
LOC_Os03g13200	1.662	1.085	1.004	0.196	0.396	0.219	-1.85362015	0.0000235
LOC_Os05g15880	28.94	29.668	33.846	110.999	91.761	111.053	1.853007066	7.52E-52
LOC_Os04g33680	2.604	2.348	2.02	0.547	0.326	0.783	-1.844314976	2.83E-09
LOC_Os12g36490	0.251	0.228	0.288	0.849	0.758	1.228	1.836999425	0.000000249
LOC_Os03g59690	1.412	0.973	1.463	0.296	0.42	0.055	-1.836576056	0.000331433
LOC_Os04g45290	5.829	6.31	4.384	1.515	1.279	1.395	-1.836344374	1.12E-23
LOC_Os07g36560	0.502	1.209	0.526	2.25	2.737	2.945	1.830502487	1.36E-09
LOC_Os09g27750	4.286	5.248	4.953	1.072	1.343	1.213	-1.824663912	2.09E-15
LOC_Os07g30860	0.044	0.174	0	0.212	0.687	0.436	1.824201934	0.004253944
MSTRG.14506	0.972	0.401	0.742	3.359	2.296	2.41	1.823417597	0.0000071
LOC_Os03g59450	2.725	2.729	2.499	8.773	8.191	9.392	1.814198052	4.75E-37
LOC_Os07g34280	1.585	1.856	1.717	0.278	0.528	0.422	-1.813148303	0.000000262
LOC_Os05g05670	3.302	3.994	4.265	1.111	0.869	0.898	-1.812288628	1.57E-11
LOC_Os05g08910	2.73	4.101	4.037	12.148	12.227	12.31	1.809517539	3.87E-18
LOC_Os11g26390	1.243	1.527	1.322	0.432	0.146	0.314	-1.808557735	0.0000547
LOC_Os09g17540	3.201	2.379	1.782	0.588	0.484	0.722	-1.797518823	2.45E-08
LOC_Os01g65240	0.807	0.6	0.894	2.628	2.532	2.73	1.796488796	2.31E-11
LOC_Os06g16160	0.171	0.084	0.195	0.452	0.542	0.75	1.791447322	0.0000406
LOC_Os06g46799	23.064	23.589	25.038	5.953	6.633	6.513	-1.790042753	1.71E-57
LOC_Os01g67490	0.357	0.255	0.248	0.796	1.054	1.402	1.785098099	0.0000273
LOC_Os05g26660	5.668	4.139	4.684	1.181	1.275	1.344	-1.781804411	7.45E-20
LOC_Os11g40780	0.037	0.037	0.101	0.338	0.307	0.166	1.762431142	0.001297931
MSTRG.11643	0.502	0.497	0.184	2.323	1.275	1.448	1.76117845	0.000706063
LOC_Os04g24328	31.41	34.622	22.258	8.903	7.344	6.989	-1.76106851	1.81E-15
LOC_Os04g24319	21.314	23.622	15.989	5.689	5.19	5.395	-1.760968729	7.14E-23

LOC_Os01g58960	0.764	0.724	0.487	0.192	0.065	0.15	-1.757710784	0.000467358
LOC_Os12g02160	0.077	0.152	0	0.517	0.3	0.898	1.751992861	0.007847199
LOC_Os08g41880	40.457	39.882	36.202	10.128	10.04	11.801	-1.751943787	1.18E-110
LOC_Os07g48030	0.508	0.88	0.737	0	0.29	0.114	-1.749165913	0.00140093
LOC_Os06g18010	9.43	9.807	8.349	2.431	2.534	2.532	-1.744596159	7.41E-28
LOC_Os01g67110	6.782	9.761	8.35	1.356	1.4	3.239	-1.724907333	0.00000575
LOC_Os11g41780	0.136	0.077	0.071	0.281	0.323	0.525	1.720460741	0.000416751
LOC_Os02g26140	1.912	1.94	1.667	0.45	0.593	0.336	-1.713036126	0.00000421
LOC_Os02g50040	1.589	1.004	1.055	0.423	0.268	0.198	-1.710134528	0.00000331
LOC_Os06g04510	0.899	0.411	0.824	0.167	0.169	0.125	-1.709177705	0.000506227
LOC_Os06g48180	11.526	11.97	10.849	34.495	34.581	35.742	1.703859642	5.22E-72
LOC_Os04g19960	11.567	11.4	13.117	40.465	34.467	35.304	1.701600757	2.49E-55
LOC_Os08g14660	2.446	2.255	2.331	7.522	6.938	7.098	1.69924095	8.02E-34
LOC_Os02g15169	64.294	65.731	79.413	231.462	212.814	194.676	1.696880196	1.03E-42
LOC_Os07g44920	3.965	5.523	3.214	0.942	1.068	1.347	-1.695982968	1.16E-08
LOC_Os02g56690	2.193	3.205	3.367	9.52	7.344	10.285	1.692405557	6.62E-17
LOC_Os05g27304	3.931	3.685	3.039	0.723	0.914	1.257	-1.686925724	7.42E-14
MSTRG.6375	0.878	2.61	0.805	5.082	5.866	4.091	1.686370154	0.0000273
LOC_Os08g28600	0.302	0.259	0.351	0.019	0.138	0.018	-1.683913585	0.002121517
LOC_Os10g25870	6.623	6.427	4.257	0.988	2.137	1.54	-1.68319461	3.88E-09
LOC_Os02g42820	4.816	3.464	3.992	1.4	1.161	0.714	-1.678864094	7.67E-08
LOC_Os11g47510	87.29	78.908	87.771	260.424	240.452	256.529	1.678645113	1.28E-231
LOC_Os10g11100	0.408	0.898	0.707	1.793	2.305	2.535	1.675679883	0.00000054
LOC_Os10g14020	0.477	0.649	0.601	2.184	1.689	2.042	1.675609431	0.0000116
LOC_Os05g11570	4.761	3.939	3.56	1.026	1.085	1.251	-1.672982263	1.38E-10
LOC_Os09g37400	0.349	0.604	1.198	2.69	2.897	2.122	1.672174503	0.0000576
LOC_Os06g03710	5.42	5.986	4.918	1.253	1.638	1.782	-1.668052077	1.14E-26
LOC_Os04g41750	0.438	0.833	0.569	0.07	0.107	0.198	-1.66773311	0.001316101
LOC_Os06g07420	0.022	0.22	0.102	0.386	0.391	0.562	1.657063971	0.000625019
LOC_Os01g16770	2.341	2.755	2.348	0.565	0.286	0.99	-1.654801826	0.0000389
LOC_Os02g50000	0.278	0.229	0.254	1.25	0.679	0.918	1.652088143	0.000365493
LOC_Os07g06830	4.746	6.117	4.968	1.337	1.561	1.591	-1.651478608	8.57E-14
LOC_Os09g33490	1.973	1.768	1.593	0.453	0.413	0.508	-1.650741731	0.00000855
LOC_Os11g34570	1.629	1.529	1.493	4.797	4.527	5.335	1.647307356	3.33E-08
LOC_Os06g51110	5.554	5.596	5.493	1.475	2.105	1.239	-1.643581285	1.57E-17

LOC_Os09g38450	4.157	3.312	3.312	1.318	0.882	0.863	-1.64340047	1.12E-13
LOC_Os04g15800	5.525	6.406	5.137	1.739	1.341	1.802	-1.643278411	2.77E-15
LOC_Os03g11400	4.797	4.847	3.833	1.201	1.06	1.569	-1.639991341	6.05E-12
LOC_Os03g05610	0.648	0.706	0.475	0.187	0.158	0.058	-1.63950365	0.00085805
LOC_Os01g33040	6.443	6.459	4.787	1.767	1.538	1.873	-1.638724243	1.18E-29
LOC_Os06g40870	3.414	4.93	4.456	1.061	1.188	1.305	-1.638639445	6.56E-09
LOC_Os04g43650	17.609	17.187	19.996	54.54	52.885	51.349	1.631685373	7.06E-108
LOC_Os04g41980	4.143	5.682	5.007	1.812	1.105	1.104	-1.630165135	0.000000701
LOC_Os04g49260	1.046	0.471	1.482	3.761	2.789	3.516	1.629547369	0.0000199
LOC_Os12g02710	2.988	2.951	2.569	0.692	0.938	0.823	-1.627010516	7.04E-14
LOC_Os06g03500	1.062	1.09	1.115	0.186	0.396	0.313	-1.626914348	0.00000027
LOC_Os11g04800	0.069	0.046	0.063	0.289	0.315	0.208	1.626034953	0.002955872
LOC_Os02g10020	3.997	4.57	3.277	1.112	1.09	1.179	-1.625800021	2.02E-11
LOC_Os06g51000	1.885	1.334	0.803	0.39	0.198	0.304	-1.625396919	0.000843526
LOC_Os03g63900	1.388	2.007	2.442	0.398	0.257	0.88	-1.624070868	0.00000642
LOC_Os03g61500	2.056	1.901	2.262	7.007	8.576	4.326	1.619439922	0.00000154
LOC_Os06g21950	0.499	0.727	0.323	1.501	1.779	1.535	1.614592976	0.000000253
LOC_Os02g17880	9.697	12.075	9.312	3.034	3.011	3.073	-1.613932422	4.19E-16
LOC_Os08g40720	48.802	50.709	42.472	12.634	14.87	15.437	-1.610535033	9.02E-81
LOC_Os02g01220	0.828	0.504	0.467	0.097	0.15	0.045	-1.605815304	0.002454694
LOC_Os05g08530	0.832	0.584	0.826	0.268	0.136	0.125	-1.605567906	0.000523586
LOC_Os03g49300	7.095	5.92	6.163	20.055	17.65	18.188	1.604151934	5.43E-17
LOC_Os03g18630	7.385	7.015	5.792	1.618	2.173	2.259	-1.602341377	1.91E-25
LOC_Os02g44090	0.222	0.22	0.61	1.64	0.939	1.466	1.600913974	0.00091722
LOC_Os03g08880	3.022	4.145	3.587	0.934	0.985	1.188	-1.599978847	2.48E-10
LOC_Os12g16210	3.721	4.046	2.441	0.934	1.006	1.037	-1.592342843	5.92E-11
LOC_Os10g35580	12.115	10.958	10.605	3.217	3.385	3.579	-1.590887736	1.08E-25
LOC_Os03g64340	3.276	2.18	2.914	8.444	8.08	7.629	1.589456091	1.92E-17
LOC_Os04g56430	359.825	346.419	439.967	1062.28	1027.143	1149.047	1.586103822	4.53E-39
LOC_Os05g41390	7.777	6.837	6.854	2.166	2.731	1.63	-1.583306604	1.17E-21
LOC_Os06g09980	1.457	2.085	1.83	5.414	5.487	4.818	1.581398556	1.43E-11
LOC_Os05g43940	4.201	5.398	3.826	1.573	1.231	1.082	-1.577705152	1.59E-10
LOC_Os07g37230	3.964	3.854	3.702	1.429	1.014	0.969	-1.577691139	2.76E-11
LOC_Os06g09870	13.026	13.586	15.86	40.041	41.977	36.958	1.576952686	2.17E-60
LOC_Os06g18670	90.645	87.46	77.444	26.514	26.683	26.43	-1.57170805	1.5E-132

LOC_Os10g42660	4.258	4.725	5.31	1.356	1.499	1.421	-1.570834376	4.21E-12
LOC_Os08g04230	68.185	61.774	70.865	190.967	178.519	185.352	1.568026128	8.33E-192
LOC_Os02g15810	24.379	24.277	20.83	6.317	7.966	7.32	-1.567748801	4.98E-53
LOC_Os02g03640	0.079	0.081	0.14	0.458	0.375	0.436	1.565654504	0.001087219
LOC_Os03g52650	21.875	19.729	17.185	5.817	6.033	6.366	-1.562591713	1.02E-33
LOC_Os09g27135	0.481	0.45	0.508	1.086	1.953	1.606	1.561528158	0.0000511
LOC_Os12g42220	0.753	1.099	0.763	0.153	0.271	0.214	-1.559468013	0.000541678
LOC_Os03g62060	145.288	144.648	131.18	39.34	40.737	51.023	-1.557474775	2.68E-34
LOC_Os02g21750	0.507	0.23	0.213	0.999	0.951	0.953	1.555977828	0.00000395
LOC_Os03g07810	5.51	6.316	4.986	1.564	2.113	1.462	-1.555806238	9.57E-15
LOC_Os01g24030	26.316	26.91	28.199	74.831	69.584	75.5	1.55400541	1.75E-156
LOC_Os11g03840	0.815	0.6	0.249	2.117	1.43	1.376	1.553708506	0.000000207
LOC_Os02g57770	0.999	1.179	1.091	0.321	0.326	0.172	-1.55161981	0.000431411
LOC_Os05g40080	1.715	1.348	1.573	0.342	0.636	0.213	-1.549333323	0.000281228
LOC_Os08g31120	0.339	0.179	0.36	0.802	1.599	1.033	1.547233877	0.00191093
LOC_Os04g40580	0.858	1.233	0.865	3.559	2.936	2.167	1.542099159	7.41E-08
LOC_Os01g74340	2.646	2.796	2.911	0.936	0.517	0.795	-1.538620347	0.0000761
LOC_Os11g03820	0.303	0.243	0.423	0.708	1.156	0.989	1.537795222	0.000000118
LOC_Os04g30570	1.571	1.124	1.72	0.505	0.256	0.433	-1.537350983	0.0000673
LOC_Os01g03630	17.428	16.531	14.825	44.064	41.976	45.866	1.535687489	1.31E-100
LOC_Os03g03630	6.297	5.875	5.381	1.887	1.315	2.095	-1.535574164	3.9E-10
LOC_Os05g38740	10.878	11.786	9.133	3.414	4.054	2.054	-1.532383519	2.25E-10
LOC_Os12g13445	25.253	28.572	25.046	8.278	7.859	8.914	-1.530551063	1.37E-39
LOC_Os08g40690	174.46	162.328	175.648	482.277	446.682	445.019	1.525482181	5.79E-243
LOC_Os10g20350	0.953	1.708	1.304	0.292	0.444	0.382	-1.525335705	0.0000144
LOC_Os07g08160	2.104	2.556	2.49	0.72	0.531	0.735	-1.520193484	0.0000399
LOC_Os03g48780	27.086	26.845	27.591	8.619	8.253	9.258	-1.518822083	9.16E-43
LOC_Os07g25690	28.84	32.945	37.611	95.288	79.539	91.797	1.517445683	5.94E-62
LOC_Os03g03334	5.286	5.703	7.001	18.132	16.77	14.832	1.516668667	1.57E-14
LOC_Os09g17810	0.322	0.399	0.418	1.579	0.708	1.161	1.513112607	0.0000157
LOC_Os03g16260	20.337	19.908	19.179	56.027	49.462	52.737	1.51308076	4.19E-108
LOC_Os02g58360	0.874	0.791	1.069	2.445	2.602	2.4	1.509954107	2.55E-19
LOC_Os11g37960	51.172	53.449	60.936	151.236	142.602	146.674	1.509789367	4.41E-108
LOC_Os09g34020	3.171	4.116	4.411	12.973	7.804	12.03	1.509562934	8.74E-10
LOC_Os09g28280	1.108	1.198	1.201	0.292	0.296	0.318	-1.509446877	0.000440269

LOC_Os07g46920	5.763	5.942	5.069	1.898	1.695	1.648	-1.509340067	3.14E-12
LOC_Os02g33380	1.291	1.279	0.623	0.525	0.066	0.061	-1.503238107	0.003480319
LOC_Os03g28090	1.274	1.906	0.906	0.226	0.534	0.422	-1.498300932	0.0000124
LOC_Os11g46120	0.285	0.06	0.28	0.608	0.696	0.66	1.496923026	0.000112841
LOC_Os05g24580	0.052	0.103	0.048	0.25	0.609	0.374	1.494959188	0.0082568
LOC_Os09g11460	3.476	5.33	4.155	1.168	1.593	1.318	-1.493583606	6.49E-10
LOC_Os09g16790	0.665	1.089	1.036	2.921	2.507	2.335	1.493555168	5.37E-11
LOC_Os01g42070	3.847	3.125	3.692	0.82	1.312	1.271	-1.48888125	7.92E-14
LOC_Os09g15550	0.574	0.645	0.772	0.111	0.225	0.138	-1.487865641	0.001689127
LOC_Os07g17010	1.169	1.195	1.105	0.211	0.429	0.329	-1.48431887	0.000107649
LOC_Os02g56540	4.954	4.518	4.38	1.308	1.576	1.613	-1.48289121	9.56E-21
LOC_Os09g34250	5.93	6.438	6.42	2.13	2.005	1.963	-1.481326681	1.6E-18
LOC_Os01g43774	6.88	7.495	5.956	2.115	1.796	2.67	-1.480124941	4.93E-19
LOC_Os12g08930	2.538	3.039	4.364	0.306	1.448	0.954	-1.479330002	0.000268694
LOC_Os08g08130	0.823	0.992	0.743	2.221	3.025	1.988	1.477469958	0.000000135
LOC_Os02g26480	0.916	1.622	1.081	3.11	3.534	3.08	1.475941187	8.56E-10
LOC_Os06g49750	1.169	1.459	1.469	0.501	0.381	0.273	-1.471359486	0.000123581
LOC_Os06g15430	1.047	1.297	0.96	2.625	3.021	3.589	1.469559922	0.000000324
LOC_Os02g56680	23.725	23.412	24.705	65.323	56.97	63.454	1.469048431	1.21E-89
LOC_Os03g10970	2.865	1.419	2.627	0.346	0.7	0.646	-1.466103041	0.001484082
LOC_Os10g06000	1.417	1.053	0.812	0.342	0.346	0.106	-1.465746357	0.001709128
LOC_Os09g38350	2.542	2.432	1.768	0.479	0.629	0.975	-1.464540087	0.000000167
LOC_Os10g31670	3.92	3.607	3.081	10.738	7.256	10.291	1.464484323	3E-12
LOC_Os07g01904	7.42	9.039	9.99	19.513	18.628	17.174	1.46447081	1.42E-72
LOC_Os08g27840	9.249	9.782	8.646	3.022	2.87	3.343	-1.460879138	5.09E-42
LOC_Os01g13260	7.433	6.418	6.573	2.228	2.381	2.106	-1.460839544	2.85E-21
LOC_Os08g06620	2.738	4.551	3.158	0.852	1.555	0.637	-1.455754745	0.0000656
LOC_Os09g06950	0.073	0.072	0.268	0.352	0.785	0.987	1.454553402	0.006790701
LOC_Os02g37480	243.628	237.576	294.77	705.88	627.959	666.637	1.454412823	1.03E-36
LOC_Os05g02300	32.011	30.773	22.499	8.551	10.673	9.183	-1.452605354	8.98E-24
LOC_Os10g10290	1.006	0.345	0.177	2.165	1.513	2.094	1.451209827	0.002447882
LOC_Os02g47130	28.296	27.714	23.537	7.956	9.064	9.869	-1.447328273	8.46E-58
LOC_Os02g31867	4.694	3.779	3.564	10.437	9.503	11.047	1.444905068	1.58E-23
LOC_Os01g09580	6.407	6.978	5.874	1.719	2.074	2.526	-1.444832712	2.12E-13
LOC_Os03g46920	5.953	4.094	3.789	1.401	1.78	1.421	-1.436001691	8.19E-15

LOC_Os12g36890	4.814	5.807	4.593	1.858	1.76	1.523	-1.435878095	8.85E-26
LOC_Os07g44130	0.492	0.152	0.028	0.861	0.842	0.555	1.434880222	0.001271588
LOC_Os03g12500	1.675	1.688	1.615	0.446	0.649	0.469	-1.433618304	0.00000115
LOC_Os09g26760	1.147	1.196	0.996	0.369	0.335	0.345	-1.425959758	0.00000171
LOC_Os06g35560	22.655	21.455	23.413	55.62	54.122	59.301	1.423549865	1.78E-109
LOC_Os06g18140	4.651	4.393	3.667	1.286	1.576	1.37	-1.422342689	2.6E-12
LOC_Os10g02500	0.473	0.41	0.475	0.171	0.116	0.094	-1.421256233	0.000182886
LOC_Os08g43980	1.515	0.869	0.585	0	0.468	0.072	-1.419959713	0.006246625
LOC_Os10g10300	0.589	0.399	0.25	1.137	1.206	0.959	1.418046166	0.000000139
LOC_Os02g11970	10.003	11.006	11.693	30.336	25.165	26.856	1.415404892	2.81E-30
LOC_Os01g49200	11.297	10.196	8.722	2.669	3.488	4.187	-1.411550181	4.67E-24
LOC_Os11g42390	0.504	0.208	0.771	0.122	0.041	0.114	-1.410544896	0.006241704
LOC_Os01g08150	7.461	7.413	7.222	2.426	2.622	2.587	-1.406104894	7.98E-29
LOC_Os05g12210	1.256	1.116	0.675	0.334	0.423	0.039	-1.405651612	0.001424423
LOC_Os07g01570	0	0.504	0.35	1.35	0.991	1.584	1.402431268	0.006409745
LOC_Os03g13050	1.533	1.119	1.85	3.425	4.103	4.185	1.40110795	1.55E-09
LOC_Os12g06080	35.071	40.782	40.242	16.073	10.747	13.448	-1.400488478	5.13E-30
LOC_Os01g70360	9.826	9.496	9.315	24.957	23.22	22.593	1.400419468	2.4E-57
LOC_Os05g31140	15.305	16.27	16.921	38.76	39.756	41.022	1.400347033	5.6E-79
LOC_Os11g37000	0.482	0.539	0.613	0.165	0.228	0.098	-1.400329522	0.000119789
LOC_Os04g52280	16.204	16.43	16.307	39.755	40.025	40.667	1.400003208	4.9E-146
LOC_Os01g17160	0.951	0.907	0.888	0.19	0.386	0.291	-1.39946803	0.0000072
LOC_Os04g41900	18	17.421	15.212	5.084	6.734	5.741	-1.396192609	1.45E-23
LOC_Os03g18990	0.066	0.197	0.121	0.639	0.388	0.836	1.393187858	0.007189579
LOC_Os02g56460	60.554	58.446	63.284	151.039	143.405	151.711	1.3928195	2.75E-165
LOC_Os01g09900	9.294	8.119	8.765	2.196	3.116	3.695	-1.390944253	2.04E-16
LOC_Os03g25370	1.145	1.588	0.945	0.276	0.168	0.516	-1.389316427	0.00143525
LOC_Os01g73940	0.401	1.193	1.288	1.742	3.531	3.438	1.385139867	0.000452225
LOC_Os02g14840	0.075	0.049	0	0.264	0.292	0.112	1.383821415	0.007962269
LOC_Os01g62110	2.577	4.125	2.817	0.956	1.163	0.715	-1.380587027	0.000229938
LOC_Os01g70560	6.678	7.225	5.785	2.213	2.123	2.439	-1.380383875	1.53E-13
LOC_Os10g38340	21.161	19.133	20.457	6.467	6.31	8.52	-1.380271848	6.87E-27
LOC_Os07g29190	3.807	4.063	3.625	1.036	1.241	1.541	-1.379890148	6.85E-08
LOC_Os04g27980	2.173	2.679	1.417	0.792	0.755	0.392	-1.376716343	0.0000687
LOC_Os04g20100	1.027	0.819	0.628	2.017	2.212	2.044	1.376645181	1.3E-10

LOC_Os11g40820	0.079	0.049	0.036	0.162	0.195	0.17	1.374868687	0.001647721
LOC_Os06g17490	5.56	5.983	4.993	1.759	2.05	1.994	-1.371048942	6.16E-19
LOC_Os10g31640	0.537	0.665	0.554	1.942	1.575	1.634	1.368854717	0.000378979
LOC_Os06g10870	7.012	8.161	7.858	19.168	17.197	19.777	1.368290245	1.77E-26
LOC_Os06g11610	8.139	9.055	7.295	3.202	2.757	2.583	-1.368161867	1.81E-14
LOC_Os11g10510	134.543	129.165	133.108	329.954	312.413	310.858	1.367351807	5.41E-245
LOC_Os01g66530	1.227	1.472	0.947	0.249	0.063	0.582	-1.366941649	0.002687997
LOC_Os06g48100	0.076	0.07	0.031	0.17	0.365	0.171	1.366827854	0.00467981
LOC_Os02g53620	1.41	1.847	1.801	0.632	0.591	0.318	-1.366316229	0.000183082
LOC_Os09g23560	0.797	0.888	0.794	0.256	0.227	0.239	-1.36497685	0.000588194
LOC_Os02g54140	4.222	3.333	6.956	14.383	10.643	13.506	1.361733071	0.00000605
LOC_Os08g37470	9.435	9.349	9.683	24.256	23.747	20.791	1.358490644	2.25E-32
LOC_Os02g09400	2.568	2.678	2.509	6.877	6.145	5.942	1.356742604	1.73E-16
LOC_Os02g06340	8.506	10.059	7.938	2.823	3.637	2.97	-1.355178824	6.53E-20
LOC_Os01g45110	18.108	19.051	17.602	6.339	7.432	5.954	-1.355143503	1.86E-39
LOC_Os10g38660	99.675	88.049	113.88	243.833	249.171	232.098	1.353423099	2.59E-35
LOC_Os02g18880	2.455	2.883	2.751	1.053	0.889	0.492	-1.35106657	0.000352539
LOC_Os10g20840	29.992	35.727	32.327	10.27	13.096	11.666	-1.350651738	8.59E-21
LOC_Os09g29210	8.401	7.698	7.888	2.687	3.34	2.535	-1.350138551	2.57E-19
LOC_Os02g57010	1.222	0.835	0.618	0.325	0.247	0.152	-1.346230835	0.0016848
LOC_Os02g52930	0.146	0.507	0.201	1.41	0.822	0.461	1.344929852	0.001509816
LOC_Os02g56700	200.073	203.267	207.095	502.286	473.984	467.104	1.34456998	2.49E-255
LOC_Os12g02210	9.303	9.472	11.191	22.068	26.624	23.479	1.341924023	8.59E-22
LOC_Os02g36470	0.034	0.033	0.041	0.079	0.211	0.138	1.341097484	0.004470027
LOC_Os02g02780	16.918	20.31	18.295	45.458	42.727	43.106	1.340347958	9.12E-84
LOC_Os03g60620	39.209	40.992	37.933	14.344	13.76	15.094	-1.339143604	6.46E-93
LOC_Os11g37100	5.474	5.925	5.041	1.815	2.334	1.762	-1.33542727	8E-17
LOC_Os01g68050	1.684	1.686	2.97	0.704	0.546	0.756	-1.331406099	0.00000927
LOC_Os11g17954	1.547	1.184	1.676	3.086	4.193	3.614	1.331265794	1.91E-09
LOC_Os12g16890	88.611	91.125	106.982	239.792	237.185	202.393	1.330843802	8.12E-32
LOC_Os09g17530	2.126	1.998	1.05	0.263	0.64	0.639	-1.330398954	0.000515345
LOC_Os08g17500	0.452	0.896	0.942	0.278	0.241	0.074	-1.329937688	0.002849159
LOC_Os11g33000	21.113	19.191	26.091	54.994	55.292	47.19	1.329567108	4.41E-32
LOC_Os05g01444	65.97	63.965	76.279	169.476	159.181	154.588	1.327980741	8.59E-109
LOC_Os01g51030	4.049	6.251	4.403	11.355	13.259	11.21	1.327570468	7.04E-11

LOC_Os08g43210	4.043	3.358	4.034	11.241	8.022	8.418	1.326101276	3.57E-12
LOC_Os02g02400	237.947	235.761	223.14	82.728	87.384	87.962	-1.325972389	9.66E-228
LOC_Os04g24469	6.424	7.749	10.442	1.414	3.157	3.669	-1.325229593	0.00000124
LOC_Os12g41630	0.443	0.11	0.71	1.068	1.515	1.298	1.324328414	0.001278149
LOC_Os01g19820	30.532	30.566	31.271	79.092	68.437	68.202	1.322988213	1.12E-67
LOC_Os03g63310	9.091	6.96	8.227	2.904	2.828	3.088	-1.322168998	7.26E-19
LOC_Os05g17604	1.434	1.319	1.462	3.148	3.292	3.116	1.32113696	8.02E-19
LOC_Os02g45940	40.711	37.861	37.239	14.413	13.422	14.731	-1.320661939	1.61E-37
LOC_Os12g16200	1.195	1.651	1.096	0.245	0.602	0.392	-1.318516355	0.000213527
LOC_Os07g08150	1.922	2.556	1.809	0.439	0.445	1.095	-1.318058018	0.000146208
LOC_Os05g43140	8.456	9.182	6.107	2.961	2.605	2.873	-1.317345002	2.33E-10
LOC_Os01g12600	0.222	0.233	0.285	0.05	0.034	0.094	-1.317308597	0.004453787
LOC_Os07g44460	1.997	1.64	1.517	0.606	0.67	0.309	-1.316720275	0.000425346
LOC_Os09g26620	18.514	19.033	17.172	5.776	5.786	8.27	-1.313487688	1.72E-23
LOC_Os01g73580	0.24	0.37	0.294	0.928	0.705	0.843	1.312431276	0.00022089
LOC_Os01g07060	10.678	11.866	10.614	4.236	3.123	4.41	-1.311282082	1.66E-09
LOC_Os10g03400	9.802	11.101	9.416	3.8	3.851	3.526	-1.310344067	5.13E-24
LOC_Os10g39600	0.65	1.427	0.639	2.778	2.634	1.676	1.308397549	0.0000385
LOC_Os11g12260	0.38	0.264	0.314	0.66	0.818	1.303	1.307800684	0.000882654
LOC_Os03g64130	1.029	1.744	1.492	0.416	0.617	0.359	-1.307570844	0.0000854
LOC_Os07g37210	3.156	3.908	3.054	1.226	1.2	1.107	-1.307487471	0.00000025
LOC_Os01g52690	39.136	36.799	32.183	12.904	13.293	14.165	-1.306689871	8.36E-63
LOC_Os01g16650	15.318	13.81	12.839	3.488	5.529	6.226	-1.305656976	1.77E-13
LOC_Os09g08100	4.865	6.628	9.061	16.574	19.178	14.398	1.304589384	6.23E-09
LOC_Os03g61590	11.462	11.675	11.688	3.927	3.247	5.217	-1.302680697	4.01E-09
LOC_Os03g39020	12.133	11.444	9.386	3.988	4.266	4.022	-1.300745406	2.83E-36
LOC_Os01g52750	0.304	0.301	0.438	0.084	0.085	0.098	-1.300708447	0.003564425
LOC_Os04g12720	7.218	7.359	7.021	2.687	2.67	2.614	-1.299346411	8.94E-17
LOC_Os08g40910	0.561	0.618	0.515	2.046	1.281	1.463	1.299096668	0.000629066
LOC_Os12g41720	3.794	3.488	3.291	1.187	1.37	1.233	-1.298089204	3.7E-09
LOC_Os05g09500	46.164	45.801	49.395	110.994	105.389	107.691	1.298018321	1.43E-144
LOC_Os09g29600	5.307	6.123	5.45	2.185	1.938	2.106	-1.297866063	1.32E-17
LOC_Os01g72150	7.393	7.256	5.131	1.866	3.581	1.495	-1.296008081	0.000000777
LOC_Os07g06850	2.331	1.529	1.535	4.371	4.463	3.998	1.295444123	9.84E-11
LOC_Os01g59120	9.346	10.447	7.91	2.861	3.691	3.701	-1.29522115	8.94E-19

LOC_Os03g55590	12.762	12.301	14.37	29.934	28.013	32.445	1.295136542	2.14E-59
LOC_Os08g14880	20.96	18.075	19.781	47.883	47.633	46.245	1.294990114	4.48E-114
LOC_Os02g12350	0.657	0.412	0.414	1.643	1.176	1.076	1.294748743	0.0000352
MSTRG.19369	1.571	0.715	1.012	2.785	3.446	2.068	1.294002541	0.00000574
LOC_Os10g11730	15.725	14.554	13.727	4.996	6.537	4.586	-1.292633024	5.54E-12
LOC_Os01g32670	37.557	40.896	36.566	12.287	15.181	15.511	-1.292340161	1.61E-26
LOC_Os06g50040	2.983	2.704	4.189	7.408	6.639	9.615	1.291341186	1.66E-09
LOC_Os08g40170	19.813	19.849	16.955	6.201	8.008	6.989	-1.290319381	4.65E-26
LOC_Os01g70850	14.337	13.301	12.23	3.336	5.634	5.332	-1.289039742	8.38E-16
LOC_Os12g42160	2.184	2.607	1.942	0.734	0.841	0.881	-1.28421523	1.48E-10
LOC_Os10g25220	2.19	1.824	1.794	1.011	0.319	0.693	-1.27935006	0.00000321
LOC_Os07g44280	1.49	1.284	1.129	0.313	0.444	0.351	-1.279273796	0.002031376
LOC_Os02g03870	1.601	2.328	1.823	0.697	0.783	0.559	-1.27773434	0.00000104
LOC_Os05g35930	4.537	3.021	4.637	9.399	8.799	10.531	1.277085534	2.02E-10
LOC_Os04g28260	5.052	4.99	4.397	1.718	2.055	1.664	-1.276699551	3.33E-20
LOC_Os02g28850	3.32	3.174	3.348	0.993	1.363	1.317	-1.274110262	1.84E-14
LOC_Os08g32600	5.026	5.831	4.392	1.388	1.911	2.363	-1.274037829	1.59E-13
LOC_Os11g47530	132.611	129.802	141.729	313.98	285.779	309.727	1.27155147	2.03E-141
LOC_Os03g52410	4.224	3.999	3.873	1.449	1.285	1.354	-1.271151967	0.000056
LOC_Os03g29150	3.1	3.221	3.19	0.995	1.434	0.995	-1.271145871	0.00000196
LOC_Os01g43740	3.836	3.714	3.597	1.149	1.023	1.887	-1.270222754	3.49E-09
LOC_Os10g42130	2.062	2.102	2.246	5.621	4.85	4.392	1.269669336	3.85E-13
LOC_Os04g44640	1.36	1.3	0.921	0.399	0.439	0.459	-1.269136199	3.83E-08
LOC_Os03g15960	55.222	55.05	83.059	161.134	134.913	159.183	1.268366922	2.97E-09
LOC_Os04g44500	3.728	2.393	2.214	6.179	6.982	6.345	1.266990554	9.32E-10
LOC_Os11g47500	264.022	269.169	277.643	608.302	597.45	607.835	1.264297827	3.68E-251
LOC_Os11g05730	27.436	24.923	23.174	9.055	10.394	9.142	-1.264145928	1.83E-18
LOC_Os04g50960	5.848	4.765	4.335	1.809	1.694	2.114	-1.264134191	2.7E-15
LOC_Os12g01700	3.064	3.983	2.948	1.154	1.232	1.313	-1.261445236	3.77E-11
LOC_Os03g49430	1.016	1.187	1.49	2.487	2.635	2.942	1.259857396	3.89E-10
LOC_Os06g41640	1.048	0.849	0.812	2.08	2.137	2.024	1.257670493	8.58E-12
LOC_Os02g37000	7.174	6.542	5.386	3.109	2.288	1.641	-1.256190099	1.48E-08
LOC_Os02g15090	10.787	9.635	11.381	24.879	24.016	22.421	1.256107877	1.36E-42
LOC_Os02g27510	1.927	1.878	1.981	4.511	4.353	4.292	1.254428878	2.04E-18
LOC_Os07g03580	1.129	2.157	1.553	0.389	0.473	0.509	-1.250701049	0.00253461

LOC_Os09g00999	3.093	3.107	4.168	8.087	6.303	9.092	1.250080313	3.79E-17
MSTRG.20115	0.948	0.731	0.628	1.576	2.653	1.378	1.249903834	0.0000056
LOC_Os07g35860	16.366	20.303	15.699	6.899	7.06	6.016	-1.249606579	3.85E-16
LOC_Os01g52900	24.532	24.034	21.608	54.726	49.972	50.839	1.248341889	6.22E-81
LOC_Os07g46830	6.555	6.802	5.614	2.148	2.721	2.175	-1.243865436	1.82E-08
LOC_Os11g47570	148.24	143.369	167.663	341.632	322.514	350.346	1.243855813	7.51E-116
LOC_Os07g11330	42.289	38.897	44.119	103.119	92.434	82.522	1.243453716	2.62E-48
LOC_Os03g02780	37.199	37.4	32.621	13.513	12.476	15.508	-1.240232655	6.53E-26
LOC_Os02g42560	5.27	5.492	4.456	2.002	2.003	1.798	-1.240059722	3.03E-13
LOC_Os01g04360	0.814	0.88	0.82	2.284	2.171	2.269	1.239721636	0.000328591
LOC_Os09g29480	5.456	4.718	6.178	12.1	11.49	12.595	1.238935809	1.38E-18
LOC_Os03g55980	6.523	5.817	6.28	12.822	14.272	15.002	1.238537003	1.2E-13
LOC_Os02g54060	8.243	7.647	6.691	2.735	2.824	2.983	-1.237409515	6.46E-11
LOC_Os07g08240	0.724	0.882	1.531	2.309	2.231	3.313	1.235818875	0.0000716
LOC_Os01g59260	0.094	0.284	0.081	0.329	0.435	0.57	1.2349219	0.001480122
LOC_Os09g25440	0.339	0.504	0.404	0	0.199	0.061	-1.233563202	0.007491322
LOC_Os08g39300	0.927	0.899	0.998	2.191	2.092	2.52	1.233453094	0.00000242
LOC_Os05g12770	0.334	0.281	0.337	0.789	0.816	0.723	1.232384756	0.0000262
LOC_Os08g02440	3.944	4.776	4.107	1.057	1.785	2.02	-1.232008751	1.43E-10
LOC_Os02g36190	4.68	6.754	5.231	2.337	2.22	1.756	-1.231859887	2.25E-11
LOC_Os03g49310	3.971	6.296	3.095	9.579	11.068	11.641	1.231033995	0.000018
LOC_Os05g16054	2.32	1.429	1.862	5.082	4.543	3.225	1.230408254	0.000000119
LOC_Os11g34590	0.04	0.08	0.073	0.23	0.33	0.197	1.230212785	0.005853316
LOC_Os05g43510	1.105	1.125	0.986	0.404	0.438	0.243	-1.229955434	0.000271767
LOC_Os07g32390	7.433	7.853	6.128	2.929	2.449	2.934	-1.229676086	3.41E-20
LOC_Os07g01420	15.317	14.181	12.317	6.601	4.542	4.926	-1.229251158	1.43E-13
LOC_Os01g58860	9.934	10.007	10.019	22.267	21.015	22.519	1.228809899	1.42E-42
LOC_Os03g02290	5.712	5.684	5.089	1.673	2.661	2.021	-1.225964432	3.83E-13
LOC_Os09g37890	1.632	1.518	1.589	3.416	4.027	3.261	1.225303642	2.06E-11
LOC_Os02g44320	3.664	2.459	2.546	0.912	1.329	0.799	-1.225025308	0.0000678
LOC_Os07g32570	35.35	35.171	36.424	12.961	14.01	15.264	-1.224653271	3.37E-58
LOC_Os02g21640	8.216	11.166	7.199	3.717	3.412	2.885	-1.223882356	1.66E-08
LOC_Os10g14194	2.116	3.273	3.463	6.55	6.628	7.113	1.222722649	8.27E-09
LOC_Os07g44910	19.89	20.276	18.482	7.234	8.642	7.248	-1.220821722	2.69E-29
LOC_Os05g36280	79.391	74.035	70.468	26.126	31.563	30.928	-1.22033407	2.81E-46

LOC_Os05g31160	3.391	2.864	3.138	6.953	6.213	7.818	1.220183148	1.88E-13
LOC_Os03g09830	1.317	0.822	0.581	2.164	2.146	2.331	1.218143934	0.000069
LOC_Os06g25910	0.314	0.131	0.491	0.791	0.815	0.749	1.217705259	0.0000522
LOC_Os03g31750	185.729	189.373	221.329	466.469	414.553	416.827	1.217286014	2.08E-34
LOC_Os02g06290	0.995	2.032	1.732	3.638	4.446	3.104	1.216265032	0.000000662
LOC_Os01g12420	1.824	1.742	2.095	0.551	0.752	0.813	-1.21518358	0.000000348
LOC_Os07g44830	2.344	2.079	1.874	4.523	5.01	4.474	1.215028183	3.92E-13
LOC_Os04g25380	9.363	7.882	8.735	2.601	3.71	3.786	-1.213183109	6.26E-15
LOC_Os02g05270	0.191	0.189	0.35	1.38	0.84	0.774	1.212016264	0.0078121
LOC_Os06g29340	1.064	1.439	1.182	0.335	0.56	0.443	-1.211439931	0.0000143
LOC_Os05g08450	4.732	4.723	4.306	1.846	1.191	2.197	-1.21089085	5.18E-09
LOC_Os04g08350	98.009	102.969	95.868	39.856	38.5	40.305	-1.210685388	6.03E-108
LOC_Os02g44230	23.495	21.624	24.676	49.273	52.211	49.442	1.210637383	1.08E-75
LOC_Os03g59440	1.955	1.069	1.854	3.707	4.812	3.162	1.210435817	0.0000177
LOC_Os04g47780	1.705	1.793	3.126	1.041	0.714	0.628	-1.210370503	0.0000352
LOC_Os10g33240	0.6	0.42	0.291	1.056	0.897	1.146	1.210058947	0.0000141
LOC_Os03g18230	4.575	4.419	5.045	12.181	9.628	9.402	1.209731944	1.2E-12
LOC_Os01g39970	0.866	0.776	1.134	0.398	0.262	0.316	-1.208093971	0.000108241
LOC_Os01g50200	5.251	5.605	4.298	2.171	1.834	1.86	-1.207941151	4E-11
LOC_Os09g27930	9.4	6.039	7.272	3.051	3.231	2.252	-1.205517221	0.000000192
LOC_Os11g47560	174.023	160.221	210.787	407.678	358.062	427.675	1.20484697	1.57E-17
LOC_Os01g11750	2.845	3.625	1.631	1.127	1.292	0.367	-1.204459114	0.000277803
LOC_Os12g01570	0.968	0.484	0.929	2.317	1.448	2.008	1.203585411	0.0000257
LOC_Os01g49720	1.819	2.5	1.506	0.113	0.516	1.164	-1.202516528	0.001662829
LOC_Os02g46260	18.666	18.197	15.526	6.771	7.094	7.105	-1.202213738	2.71E-32
LOC_Os02g43660	0.733	0.792	0.489	0.193	0.13	0.06	-1.200693429	0.008822993
LOC_Os12g23700	6.526	7.054	7.942	2.576	2.727	2.997	-1.200648741	3.02E-09
LOC_Os05g37780	10.557	10.145	10.321	24.296	21.517	21.452	1.198916778	2.99E-23
LOC_Os03g63330	55.359	51.212	52.052	118.666	114.395	106.785	1.198570815	1.06E-117
LOC_Os01g56570	3.239	3.067	2.706	1.146	1.021	1.234	-1.198430037	0.000000431
LOC_Os08g38300	56.719	55.948	51.536	21.399	21.525	23.076	-1.198164832	4.08E-50
LOC_Os03g18130	51.97	52.808	47.196	19.951	19.436	22.142	-1.195616309	5.8E-77
LOC_Os03g15370	4.864	2.666	2.509	0.656	1.442	0.677	-1.195283368	0.000405636
LOC_Os05g39320	41.726	42.425	45.896	97.494	89.481	90.989	1.195260382	4.63E-99
LOC_Os05g49760	2.902	3.159	2.375	1.356	0.972	0.881	-1.194958633	8.66E-08

LOC_Os03g19080	6.087	5.46	4.835	2.132	1.867	2.478	-1.194448047	1.86E-17
MSTRG.20081	2.005	2.287	2.005	4.396	3.802	6.191	1.192437864	0.00000134
LOC_Os03g57490	11.053	10.346	9.567	22.208	20.838	23.223	1.188248493	2.22E-40
LOC_Os04g37970	0.934	0.814	0.685	0.252	0.292	0.202	-1.187869806	0.00228761
LOC_Os03g06670	36.024	37.928	34.063	14.4	13.234	15.856	-1.18760343	1.18E-29
LOC_Os09g23540	2.372	2.278	2.508	0.769	1.072	0.804	-1.187479613	0.00000591
LOC_Os01g21310	1.938	1.383	1.99	0.224	0.455	0.909	-1.186054504	0.002661344
LOC_Os03g19840	30.559	29.544	32.618	69.027	63.777	64.326	1.184222009	3.53E-58
LOC_Os05g48700	0.855	1.219	0.878	2.671	2.34	1.788	1.182647359	0.00000586
LOC_Os06g07914	94.268	96.955	80.524	33.549	37.19	40.039	-1.180446856	6.84E-60
LOC_Os10g31460	402.583	432.493	452.741	889.441	983.458	824.646	1.179739591	2.14E-45
LOC_Os08g04630	23.565	24.972	23.495	9.145	10.785	9.468	-1.179406752	6.69E-52
LOC_Os05g38770	3.718	4.467	3.866	1.497	1.718	1.532	-1.178553691	5.95E-10
LOC_Os01g23970	0.046	0.091	0.377	0.529	0.671	0.66	1.177899132	0.006719804
LOC_Os09g37200	2.105	1.183	1.498	0.394	0.645	0.68	-1.17768667	0.00011063
LOC_Os02g08310	2.684	2.972	2.461	0.876	1.158	1.032	-1.176734014	0.00000446
LOC_Os03g11140	1.816	1.53	1.646	0.671	0.767	0.49	-1.17633318	0.000000173
LOC_Os01g04380	4.464	3.918	4.53	10.002	9.299	9.085	1.174612726	1.05E-10
LOC_Os04g44130	8.489	8.264	4.916	3.161	1.893	2.686	-1.174209657	0.000141738
LOC_Os11g32890	10.628	9.547	12.472	28.503	20.23	21.471	1.174123024	8.24E-16
LOC_Os10g20510	3.026	2.108	2.939	6.482	5.475	5.459	1.173026929	2.39E-12
MSTRG.24964	2.609	1.352	2.157	4.701	4.827	4.638	1.172352322	1.39E-09
LOC_Os05g44340	9.211	8.826	11.48	22.035	19.519	20.874	1.17221411	2.67E-40
LOC_Os07g03180	17.125	20.31	19.686	41.303	44.021	35.863	1.172188924	6.64E-31
LOC_Os05g47540	16.493	17.059	17.015	6.877	7.456	6.487	-1.17176534	5.84E-34
LOC_Os05g18550	0.323	0.375	0.37	0.798	1.01	0.689	1.171569735	0.0000245
LOC_Os02g11930	30.528	29.049	29.544	66.868	62.033	58.239	1.171198716	1.02E-109
LOC_Os03g14180	4.459	5.329	6.491	11.22	11.372	12.396	1.171101773	1.45E-15
LOC_Os08g08960	1.617	2.403	2.012	3.343	5.421	4.896	1.17096673	0.00000214
LOC_Os06g09560	5.337	3.792	6.228	11.181	10.015	12.057	1.170784457	3.5E-12
LOC_Os09g32440	0.208	0.309	0.286	0.1	0.068	0.047	-1.169852125	0.005446781
LOC_Os01g53350	2.604	1.942	1.825	0.768	0.869	0.774	-1.169631743	0.00000648
LOC_Os04g53320	2.051	1.524	2.149	0.565	0.573	0.727	-1.168436193	0.001434895
LOC_Os02g47620	2.702	3.965	4.854	1.427	1.4	1.619	-1.16784105	0.000000512
LOC_Os03g17100	159.385	150.707	137.463	60.413	62.884	61.34	-1.165652988	3.53E-75

LOC_Os08g38880	28.66	26.888	31.169	63.358	58.585	59.608	1.164738537	1.26E-73
LOC_Os05g49430	4.615	4.121	5.139	9.495	9.942	9.607	1.163084256	8.46E-20
LOC_Os07g32620	12.336	13.359	10.96	4.928	4.248	5.704	-1.162470583	2.94E-18
LOC_Os10g24690	1.34	1.54	1.376	0.155	0.472	0.725	-1.162329452	0.001938383
LOC_Os06g49340	27.336	28.259	27.879	59.725	57.176	57.426	1.162284293	1.82E-90
LOC_Os03g25930	2.911	3.102	2.77	1.007	1.074	1.189	-1.161795237	0.0000346
LOC_Os08g03600	1.414	1.401	0.648	2.327	3.212	2.4	1.161217988	0.0000225
LOC_Os11g31715	0.679	0.547	0.467	1.352	1.163	1.647	1.160573952	0.000462746
LOC_Os11g13710	8.563	7.492	7.851	4.077	2.338	3.059	-1.160552379	4.32E-09
LOC_Os09g37490	1.198	1.088	2.196	3.755	3.025	4.23	1.1601561	0.000367573
LOC_Os02g35230	1.583	1.887	1.066	0.549	0.653	0.491	-1.160147046	0.0000347
LOC_Os05g12280	1.48	1.291	0.977	0.114	0.463	0.534	-1.159378677	0.003460385
LOC_Os02g36020	0.144	0.262	0.184	0.311	0.545	0.575	1.158677986	0.000304551
LOC_Os06g39390	5.237	4.419	3.996	9.061	9.25	10.543	1.157430009	3.35E-17
LOC_Os10g08250	0.374	0.625	0.471	1.064	1.18	1.119	1.155414926	0.00000645
LOC_Os05g06140	3.566	4.335	2.885	1.243	1.612	1.406	-1.15468433	4.66E-08
LOC_Os02g07180	2.921	2.451	2.268	1.227	0.773	0.93	-1.154423704	0.00000502
LOC_Os05g42960	0.137	0.092	0.186	0.509	0.228	0.302	1.153046708	0.000485403
LOC_Os09g02360	6.968	6.336	6.768	2.149	2.8	3.185	-1.152089171	1.09E-12
LOC_Os04g34240	8.723	7.51	6.818	3.449	3.391	2.515	-1.152064246	2.53E-12
LOC_Os04g09570	21.731	19.638	21.701	42.547	37.27	51.947	1.151616341	1.1E-28
LOC_Os09g37620	1.172	1.311	1.185	0.407	0.443	0.435	-1.151050584	0.000178164
LOC_Os06g09120	1.463	1.39	1.095	3.023	2.539	3.042	1.150928346	9.79E-08
LOC_Os01g10140	3.395	3.623	3.544	1.319	1.7	1.354	-1.150106079	2.95E-09
LOC_Os02g52650	0.353	0.299	0.647	0.875	1.182	1.454	1.149741672	0.002116896
LOC_Os10g26700	73.639	71.639	71.344	155.555	141.396	150.791	1.148927753	1.23E-93
LOC_Os01g03530	34.265	34.182	35.022	69.417	68.503	75.44	1.145689127	9.82E-98
LOC_Os05g40600	0.088	0.24	0.6	0.653	0.981	0.932	1.144595997	0.002247766
LOC_Os01g21420	0.844	0.586	0.697	1.792	2.064	1.218	1.144090337	0.000244949
LOC_Os10g02040	3.569	3.952	2.987	1.013	1.75	1.358	-1.143772918	0.00000122
LOC_Os09g23530	28.509	31.661	25.581	11.542	11.662	12.498	-1.142769967	4.94E-33
LOC_Os09g36280	3.729	4.334	4.855	1.199	1.891	1.993	-1.140661442	0.000000871
LOC_Os03g51040	4.1	4.658	3.458	1.898	1.657	1.183	-1.14057512	0.00000624
LOC_Os09g28660	2.206	1.506	0.944	0.757	0.24	0.531	-1.14035073	0.001690147
LOC_Os03g49270	10.157	9.611	10.068	18.274	22.012	22.366	1.139654899	6.93E-15

LOC_Os09g27060	2.085	2.516	1.896	0.695	0.874	0.992	-1.13882238	6.03E-08
LOC_Os09g16780	10.295	9.763	11.835	22.76	22.088	21.463	1.138486733	4.29E-25
LOC_Os09g27510	1.221	1.21	1.177	0.362	0.52	0.423	-1.138328357	0.000315416
LOC_Os09g23550	9.089	7.759	7.725	3.475	2.834	3.729	-1.137965876	2.2E-14
LOC_Os04g31050	5.819	6.262	6.077	2.305	2.977	2.12	-1.137160898	2.48E-10
LOC_Os05g26460	52.776	57.635	69.783	135.314	134.931	108.452	1.136657889	5.73E-14
LOC_Os06g05000	134.321	129.295	126.802	276.035	264.157	259.615	1.136298841	4E-120
LOC_Os04g05360	0.026	0.101	0.164	0.246	0.35	0.415	1.134853773	0.007133869
LOC_Os05g49850	0.449	0.629	0.61	1.135	1.151	1.41	1.133104707	0.0000013
LOC_Os07g03970	0.26	0.687	0.318	0.056	0.198	0.052	-1.130305846	0.007443223
LOC_Os08g44470	9.451	8.169	9.218	3.686	3.277	3.929	-1.130181795	2.93E-09
LOC_Os03g04550	6.978	6.004	5.087	2.759	2.43	2.211	-1.128210582	1.65E-10
LOC_Os01g12430	2.496	2.989	3.522	6.595	5.56	6.596	1.127914549	1.53E-15
LOC_Os03g53580	1.237	1.8	1.17	0.448	0.416	0.628	-1.127903116	0.000602246
LOC_Os03g19275	2.114	1.128	1.044	0.471	0.265	0.636	-1.127164983	0.002469927
LOC_Os05g26620	12.201	10.972	14.666	27.242	27.419	24.189	1.125784826	1.89E-16
LOC_Os07g34570	112.467	119.905	108.954	46.436	46.421	51.902	-1.124762937	1.64E-82
LOC_Os03g16030	2.979	3.896	3.894	7.471	6.596	8.762	1.123413499	7.12E-09
LOC_Os11g01620	3.739	3.745	2.881	1.128	1.677	1.376	-1.123138642	2.1E-09
LOC_Os09g25910	7.216	8.579	7.779	15.947	15.48	17.039	1.123128144	8.46E-22
LOC_Os07g26550	28.064	21.25	26.864	53.306	52.324	50.293	1.122523987	1.85E-31
LOC_Os07g10190	1.365	1.758	1.721	0.757	0.601	0.462	-1.122054815	0.000149535
LOC_Os02g30080	3.922	3.556	3.562	1.499	1.339	1.602	-1.121949969	0.000000172
LOC_Os08g04240	1276.82	1251.772	1491.148	2955.633	2638.073	2653.29	1.121506477	1.44E-28
LOC_Os07g32630	13.033	13.326	12.195	4.88	5.557	5.797	-1.120401874	2.67E-23
LOC_Os06g05860	4.058	4.587	3.747	1.599	1.76	1.727	-1.118859761	1.12E-09
LOC_Os06g43384	2.839	2.072	2.123	0.757	0.767	1.179	-1.118790335	0.0000351
LOC_Os09g29660	16.874	15.37	15.363	7.109	6.315	6.736	-1.117647035	1E-32
LOC_Os08g39290	1.969	2.048	2.076	4.179	4.14	5.505	1.117286516	0.0000889
LOC_Os01g01890	2.754	3.021	1.962	1.044	1.178	0.887	-1.117216472	0.00000054
LOC_Os12g22620	2.584	2.089	2.743	0.984	0.831	1.104	-1.116589493	0.0000074
LOC_Os12g34500	13.826	15.377	14.071	5.902	6.625	5.767	-1.116541642	1.29E-22
LOC_Os04g39600	3.805	4.465	4.132	2.029	1.469	1.445	-1.116266534	0.00000226
LOC_Os11g31060	1.697	1.717	1.854	3.588	3.742	3.842	1.114898393	3.48E-08
LOC_Os03g45390	2.927	2.932	3.244	7.137	6.354	5.425	1.114107066	3.15E-12

LOC_Os06g43900	5.498	6.611	6.287	2.563	1.812	3.009	-1.113495775	0.000000607
LOC_Os04g30720	4.412	5.015	3.567	1.707	1.942	1.737	-1.113100018	5.1E-11
LOC_Os09g23300	56.815	54.27	56.052	23.948	24.274	23.245	-1.112965989	9.23E-56
LOC_Os09g37330	4.883	5.048	3.958	11.264	9.203	8.616	1.111327272	3.17E-09
LOC_Os05g09440	61.814	61.039	63.47	128.471	122.335	124.069	1.110511102	3.18E-140
LOC_Os07g11910	45.366	46.057	62.592	115.893	119.922	88.123	1.109365666	8.91E-09
LOC_Os05g33410	73.158	70.25	69.413	146.282	138.661	142.862	1.109360373	4.68E-123
LOC_Os12g02310	25.008	29.116	26.843	11.756	11.133	11.499	-1.109320467	1.2E-26
LOC_Os05g42250	1.363	1.885	1.702	0.657	0.62	0.678	-1.108931966	0.0000102
MSTRG.10847	0.423	1.047	0.485	1.428	1.654	1.462	1.108760428	0.000321582
LOC_Os07g10230	0.891	0.475	0.942	0.331	0	0.124	-1.107426446	0.00906128
LOC_Os11g47580	415.721	403.624	503.961	954.965	806.391	933.583	1.106882394	1.4E-17
LOC_Os01g15830	7.623	6.705	6.99	2.589	3.157	3.157	-1.106198921	2.11E-11
LOC_Os01g04340	0.626	0.497	1.149	1.329	2.021	2.316	1.106172555	0.001331855
LOC_Os02g56120	12.54	13.946	11.955	27.27	24.195	26.48	1.105380195	1.66E-24
LOC_Os08g44420	14.66	13.142	12.348	5.053	6.028	6.091	-1.105287261	2.99E-33
LOC_Os04g53606	3.701	3.785	3.777	1.9	1.167	1.346	-1.104627285	0.0000266
LOC_Os04g45590	2.297	1.909	2.378	4.646	4.274	5.412	1.103960421	0.0000103
MSTRG.23415	1.388	1.052	1.048	0.236	0.319	0.368	-1.103773773	0.006990337
LOC_Os03g53590	36.553	37.163	36.929	74.941	74.892	71.706	1.102947538	4.75E-127
LOC_Os02g22260	7.517	7.008	6.92	2.571	3.536	2.863	-1.101479121	2.84E-10
LOC_Os11g09710	40.671	38.041	40.274	17.802	17.726	15.679	-1.101063007	3.08E-39
LOC_Os03g56070	4.43	4.857	4.84	1.758	2.12	2.012	-1.10077559	6.82E-10
LOC_Os11g47550	511.552	490.794	571.63	1077.192	1012.837	1066.579	1.09994744	3.28E-48
LOC_Os01g60360	1.567	2.459	3.473	6.048	5.875	5.301	1.098856337	0.000221704
ChrSy.fgenesh.gene.69	0.029	0.149	0.197	0.502	0.357	0.252	1.098413759	0.004552864
LOC_Os06g06510	49.321	46.824	42.545	17.469	21.158	20.862	-1.096853624	1.34E-27
MSTRG.9468	0.857	0.5	0.393	0.948	1.356	1.637	1.094156397	0.000182994
LOC_Os08g37290	1.914	2.055	2.998	5.155	3.587	6.618	1.093310381	0.0000494
LOC_Os11g18366	1.704	1.566	1.332	0.533	0.72	0.561	-1.091982396	0.0000205
LOC_Os12g44350	8.478	7.236	6.234	2.875	2.914	3.407	-1.09149263	4.46E-10
LOC_Os06g04950	11.703	11.619	9.749	21.502	22.2	23.38	1.091189856	2.19E-16
LOC_Os10g07114	15.717	14.657	17.235	31.544	32.776	31.287	1.090994289	6.75E-28
LOC_Os12g26960	27.206	32.711	29.205	61.739	58.129	58.417	1.090804081	8.63E-31
LOC_Os05g12400	1.311	1.082	1.322	0.253	0.641	0.394	-1.090524015	0.001810778

LOC_Os05g49860	94.282	91.985	80.725	38.573	37.681	39.399	-1.089807377	5.52E-47
LOC_Os06g04699	10.786	9.398	8.812	3.604	3.817	4.816	-1.089713897	2.63E-12
LOC_Os06g05010	100.982	91.699	106.097	197.154	199.308	197.398	1.089631756	2.07E-78
LOC_Os10g14920	3.768	3.744	4.277	1.298	1.511	2.007	-1.088968388	0.000000011
LOC_Os04g31410	1.033	0.658	1.15	0.214	0.144	0.333	-1.088898805	0.008304991
LOC_Os07g34720	2.354	1.684	2.098	5.046	4.603	3.597	1.088492254	0.0000125
LOC_Os10g19190	4.491	4.742	2.492	1.596	1.445	1.546	-1.087759983	0.0000608
LOC_Os09g02270	6.93	7.059	8.239	2.89	3.367	3.1	-1.087378797	1.31E-09
LOC_Os09g23590	5.011	3.806	5.243	11.793	9.584	10.072	1.087009103	0.000052
LOC_Os07g11410	34.95	31.834	43.9	83.311	77.777	65.093	1.086210836	7.15E-11
LOC_Os10g01570	10.91	12.147	11.04	4.224	4.995	5.397	-1.085554668	1.63E-16
LOC_Os05g26386	345.665	335.181	425.579	802.046	784.542	648.963	1.085446887	2.16E-14
LOC_Os07g22580	3.288	3.784	3.075	1.538	1.331	1.318	-1.08536546	0.000000329
LOC_Os10g20830	105.963	115.367	97.08	46.374	49.306	42.987	-1.084610094	1.75E-44
LOC_Os05g08920	8.033	6.725	9.525	3.274	3.251	3.56	-1.084284838	0.00000013
LOC_Os04g27400	0.881	1.025	0.597	0.296	0.15	0.345	-1.083656573	0.003863222
LOC_Os08g36310	4.336	4.77	5.509	9.321	10.082	10.038	1.083536656	1.29E-16
LOC_Os12g28590	1.983	1.84	1.501	0.668	0.831	0.596	-1.082904062	0.0000691
LOC_Os06g09420	3.065	1.776	1.427	0.682	0.922	0.723	-1.082693821	0.000562746
LOC_Os07g35350	104.157	103.688	100.999	202.384	199.867	206.846	1.082599227	6.31E-173
LOC_Os02g48360	24.798	27.759	24.495	10.733	11.294	11.53	-1.082589971	3.51E-42
LOC_Os09g37910	31.633	31.827	26.988	13.374	12.6	13.163	-1.082000335	1.58E-23
LOC_Os04g47580	6.558	7.167	6.691	2.357	3.332	3.015	-1.081570904	5.43E-12
LOC_Os03g41120	0.696	0.965	0.935	0.134	0.272	0.376	-1.081390398	0.005109324
LOC_Os02g32840	61.442	59.437	75.065	135.674	120.609	135.948	1.081282274	4.54E-19
LOC_Os10g31850	196.578	200.608	248.986	434.453	410.81	446.615	1.081271679	4.03E-20
LOC_Os11g18194	1.899	1.501	1.493	0.48	0.73	0.755	-1.081090551	0.000016
LOC_Os02g58520	1.156	1.285	0.646	0.408	0.303	0.407	-1.077576333	0.000983911
LOC_Os05g26720	5.56	7.197	7.071	13.15	15.055	12.288	1.077371217	9.01E-10
MSTRG.23765	2.244	2.427	2.26	4.078	5.301	4.555	1.076639559	7.66E-16
LOC_Os03g08470	85.038	92.309	83.727	36.428	38.133	40.321	-1.07608752	1.05E-75
LOC_Os02g50910	4.995	5.793	4.876	1.984	2.069	2.597	-1.075094714	2.6E-10
LOC_Os12g10220	16.129	20.491	17.328	8.305	7.934	6.783	-1.073508914	1.92E-10
LOC_Os02g03940	4.025	4.299	3.664	1.46	2.233	1.365	-1.07278685	3.81E-08
LOC_Os01g16200	0.597	0.455	0.084	0.841	1.122	1.076	1.071800525	0.004045958

LOC_Os09g36470	0.513	0.746	0.705	0.217	0.298	0.231	-1.071675746	0.000459903
LOC_Os01g73700	1.718	3.175	2.555	1.031	0.908	0.963	-1.071305275	0.000171854
LOC_Os06g30179	1.883	2.223	2.209	1.069	0.65	0.849	-1.070741919	0.0000111
LOC_Os03g19240	6.964	5.907	7.042	2.793	3.139	2.662	-1.069996558	7.84E-13
LOC_Os07g11360	39.733	39.669	49.829	91.349	85.032	78.397	1.069577375	5.92E-34
LOC_Os04g52950	2.586	2.837	3.642	4.456	8.039	6.998	1.069330265	0.0000271
LOC_Os03g02900	7.927	8.858	8.033	3.824	3.322	3.602	-1.069094187	3.17E-15
MSTRG.3248	1.194	1.105	0.939	2.025	3.758	2.362	1.067068293	0.002139758
LOC_Os02g13600	3.936	3.09	4.312	1.381	2	1.245	-1.065069622	0.0000201
LOC_Os01g07420	0.461	1.104	0.951	0.334	0.225	0.243	-1.064965092	0.004363269
LOC_Os08g10510	13.889	14.574	12.523	5.683	5.79	6.458	-1.064937653	2.81E-21
LOC_Os09g25070	3.789	3.501	4.225	9.069	6.143	8.34	1.064497248	6.71E-10
LOC_Os07g01440	33.058	32.165	32.25	13.331	13.965	15.444	-1.064352919	1.45E-24
LOC_Os03g17960	3.23	3.14	2.207	1.264	1.073	1.209	-1.063673174	0.00000256
LOC_Os01g03680	77.021	77.533	65.62	28.84	30.664	37.376	-1.063652375	1.03E-32
LOC_Os03g12820	28.776	26.384	29.169	58.571	51.387	54.757	1.063652097	7.08E-57
LOC_Os11g02300	2.934	3.108	3.333	6.346	6.241	5.475	1.06330346	7.12E-21
LOC_Os02g36210	29.104	33.274	29.24	13.167	15.132	12.223	-1.06329311	5.24E-44
LOC_Os07g47590	2.918	3.103	3.87	6.777	5.766	6.807	1.061001018	6.35E-11
LOC_Os09g02410	1.017	0.576	0.999	1.961	2.343	1.899	1.060272984	0.001459688
LOC_Os01g55240	16.337	17.244	18.128	35.221	32.016	33.739	1.060210095	1.37E-43
LOC_Os04g12970	27.743	25.848	23.517	10.872	11.349	11.841	-1.05859925	3.3E-33
LOC_Os01g46570	29.591	27.673	30.297	54.881	57.704	58.013	1.058486149	9.68E-84
LOC_Os02g04510	0.772	0.765	1.04	0.21	0.401	0.348	-1.057242723	0.000979367
LOC_Os07g35740	0.827	0.898	0.905	2.084	1.513	1.924	1.055995822	0.0000126
LOC_Os11g02100	5.473	4.126	4.091	7.896	10.752	8.85	1.05511341	3E-10
LOC_Os07g44400	4.954	4.487	3.698	1.946	2.249	1.372	-1.05490798	0.000000291
LOC_Os11g41365	0.741	0.795	0.424	1.191	1.75	1.155	1.054142771	0.0000101
LOC_Os12g10880	13.07	10.484	17.348	26.9	31.161	23.913	1.052208612	2.25E-10
LOC_Os11g06300	1.065	0.69	0.902	1.858	2.203	1.737	1.051586061	0.0001682
LOC_Os12g03130	2.111	2.431	1.925	1.22	0.623	0.83	-1.05151336	0.00000761
LOC_Os12g21734	0.997	1.288	1.2	2.206	2.384	2.559	1.050872476	0.000000705
LOC_Os10g17780	0.811	1.395	0.743	0.206	0.334	0.423	-1.050627836	0.004042433
LOC_Os10g23310	5.742	4.847	6.37	11.046	10.225	12.085	1.050008288	9.24E-16
LOC_Os11g40830	0.266	0.211	0.31	0.548	0.66	0.593	1.049816025	0.000757615

LOC_Os05g25770	4.012	4.316	4.169	1.585	1.831	1.895	-1.049699944	0.000000201
LOC_Os03g60210	16.641	15.045	14.876	32.375	28.679	29.704	1.048882665	2.84E-22
LOC_Os11g38240	6.784	6.28	7.448	14.037	13.879	13.043	1.048311691	5E-10
LOC_Os12g03730	6.156	7.001	6.267	12.805	12.706	12.268	1.046456119	1.08E-23
LOC_Os07g12610	0.351	0.696	0.778	0.226	0.172	0.185	-1.046431375	0.004885311
LOC_Os03g03910	6.673	6.394	8.183	2.888	3.061	3.368	-1.046290201	8.47E-13
LOC_Os02g43370	3.686	2.926	2.909	5.4	7.19	6.42	1.046088448	1.74E-15
LOC_Os03g61150	137.43	127.816	147.373	263.71	254.179	276.381	1.045300217	4.34E-86
LOC_Os07g09630	17.529	15.798	15.618	30.86	28.665	35.326	1.044775053	3.68E-26
LOC_Os01g16140	8.803	11.018	9.559	22.126	18.349	19.641	1.04450053	0.00000163
LOC_Os03g02750	47.011	48.371	49.441	94.632	88.653	94.878	1.043078843	2.35E-107
LOC_Os01g71130	5.431	4.29	5.148	8.815	8.683	11.749	1.042804696	2.14E-12
LOC_Os01g70370	66.038	64.735	69.929	136.353	126.141	123.131	1.042197523	2.92E-87
LOC_Os04g29960	0.345	0.41	0.232	0.044	0.135	0.083	-1.041295807	0.008159376
LOC_Os10g19910	11.84	14.04	12.819	5.893	6.466	4.601	-1.041070397	8.51E-11
LOC_Os07g22730	2.496	4.283	2.735	1.703	0.655	1.318	-1.041018429	0.000488595
LOC_Os09g11380	0.036	0.126	0.05	0.298	0.124	0.295	1.040436126	0.008495691
LOC_Os04g43230	5.528	4.964	4.366	1.579	2.625	2.235	-1.040092029	4.28E-08
LOC_Os11g47610	145.879	142.696	153.449	287.511	278.258	280.13	1.038988961	3.28E-129
LOC_Os06g22919	8.374	7.692	6.884	3.178	2.823	2.614	-1.038633348	2.47E-12
LOC_Os05g49060	5.199	6.068	5.817	2.21	2.585	2.583	-1.038128951	3.61E-08
LOC_Os04g51150	13.951	14.813	14.236	6.303	5.938	6.898	-1.036602472	1.04E-18
LOC_Os08g28560	2.127	2.959	2.438	4.894	5.161	4.981	1.036456211	3.52E-08
LOC_Os01g57854	40.014	45.217	50.12	89.771	83.966	85.616	1.035234485	1.12E-55
LOC_Os09g35850	2.487	3.238	2.951	0.941	1.622	0.968	-1.035144888	0.000113638
LOC_Os02g33550	12.856	11.799	12.223	4.931	5.358	5.988	-1.034760013	1.04E-12
LOC_Os12g34124	0.409	0.224	0.216	0.528	0.838	0.98	1.034698219	0.003974668
LOC_Os04g41710	3.073	2.929	2.496	0.901	0.647	1.859	-1.03432248	0.000138466
LOC_Os06g01250	10.723	10.939	9.346	4.444	4.583	4.8	-1.034044122	1.03E-18
LOC_Os09g23595	4.387	3.448	3.027	7.166	6.699	7.493	1.034007673	2.59E-10
LOC_Os03g55660	1.153	0.971	1.533	2.865	1.776	2.912	1.033446883	0.0000129
LOC_Os04g54280	3.701	4.566	2.932	8.238	7.777	5.911	1.032131884	8.64E-12
LOC_Os10g38670	8.009	9.283	10.576	3.207	4.778	4.225	-1.031406002	2.46E-09
LOC_Os07g28610	1.792	2.286	1.923	0.781	0.584	0.897	-1.030607249	0.001509163
LOC_Os09g31482	39.971	37.864	46.032	79.757	73.273	81.121	1.029658435	4.06E-49

LOC_Os04g25970	5.354	4.354	5.386	2.511	1.642	2.341	-1.028634939	0.000000205
LOC_Os03g02960	6.269	7.058	6.627	3.003	2.387	3.362	-1.027467115	3.26E-11
LOC_Os01g43720	3.676	4.452	3.611	1.801	1.911	1.395	-1.027279465	0.000000071
LOC_Os02g34120	2.673	1.713	2.195	4.485	4.746	4.112	1.026588784	0.000000683
LOC_Os05g26770	14.686	13.743	17.621	31.834	29.192	27.887	1.025313617	4.2E-16
LOC_Os07g23120	23.852	21.724	18.384	7.838	9.319	11.367	-1.025078691	8.82E-15
LOC_Os05g04210	3.066	3.72	2.639	6.037	6.975	5.869	1.024905416	0.000000622
LOC_Os01g45720	47.847	51.255	48.24	22.938	22.041	21.868	-1.023481095	3.74E-36
LOC_Os01g42690	1.751	1.884	2.104	4.199	3.424	4.061	1.023140578	0.00000607
LOC_Os03g12260	1.791	1.173	1.377	0.669	0.678	0.391	-1.022324985	0.000348598
LOC_Os09g38210	0.779	0.695	0.393	0.301	0.114	0.105	-1.021063625	0.007831669
LOC_Os03g07590	16.047	19.226	21.703	35.714	42.302	34.195	1.020904329	2.32E-09
LOC_Os05g28740	16.764	12.344	17.823	28.067	26.726	40.299	1.019601295	0.00000573
LOC_Os09g27990	0.379	0.205	0.316	0.499	0.978	0.809	1.018682669	0.004041447
LOC_Os08g33820	2.277	3.158	2.839	1.318	1.158	0.945	-1.018403667	0.0000935
LOC_Os05g35690	10.567	9.355	11.754	20.557	21.005	19.687	1.018068903	3.52E-13
LOC_Os03g52860	1745.673	1730.713	1575.257	738.37	777.655	798.941	-1.017893097	3.62E-137
LOC_Os04g46240	4.88	5.44	5.146	11.182	8.65	10.344	1.017828407	1.17E-09
LOC_Os08g38560	2.867	2.904	2.496	1.088	1.197	1.365	-1.017720817	1.39E-09
LOC_Os07g26100	3.015	1.992	2.164	5.101	4.23	4.926	1.017470167	0.000000568
LOC_Os07g48830	4.765	5.614	7.391	11.094	10.337	12.898	1.016574576	5E-12
LOC_Os06g46990	4.181	3.782	4.028	1.666	2.044	1.557	-1.016423612	5.21E-08
LOC_Os01g51320	0.714	0.708	0.655	0.379	0.07	0.193	-1.01587385	0.00625358
LOC_Os11g37040	1.46	1.709	1.367	0.614	0.592	0.743	-1.015591728	0.0000021
LOC_Os02g40260	2.243	1.538	1.424	0.732	0.574	0.778	-1.015418816	0.000332969
LOC_Os03g58400	1.35	1.296	1.044	0.488	0.454	0.418	-1.015012056	0.002035777
LOC_Os12g36100	1.338	1.669	1.087	0.464	0.691	0.585	-1.014323103	0.00000733
LOC_Os12g03770	0.225	0.142	0.169	0.375	0.58	0.388	1.013281432	0.003883865
LOC_Os11g04860	0.343	0.272	0.408	0.76	0.905	0.803	1.012195377	0.002871845
LOC_Os07g41460	18.003	16.971	17.709	7.131	8.705	8.101	-1.012149705	2.07E-20
LOC_Os05g36060	0.323	0.217	0.38	0.685	0.484	0.785	1.012040189	0.000228181
LOC_Os09g06770	10.029	10.664	9.046	18.171	18.577	20.297	1.011274392	4.6E-13
LOC_Os02g56830	1.501	1.569	1.809	3.222	3.581	2.872	1.010834482	6.07E-08
LOC_Os03g08310	2.521	2.165	2.723	0.811	0.986	1.162	-1.009492264	0.000470814
LOC_Os05g26350	72.344	69.456	85.638	153.76	145.041	130.581	1.009114906	3.35E-38

LOC_Os02g47180	32.825	34.101	30.502	15.306	14.818	14.708	-1.008099082	8.36E-51
LOC_Os02g33590	3.883	3.538	3.401	7.057	6.238	7.504	1.007481735	2.8E-11
LOC_Os01g07910	30.238	31.141	31.115	13.714	13.619	15.044	-1.007035766	3.6E-32
LOC_Os07g01400	1.334	1.983	1.325	0.59	0.598	0.501	-1.006541418	0.002628535
LOC_Os06g06460	35.716	39.152	32.589	16.404	15.53	17.137	-1.006410368	7.1E-25
LOC_Os09g34214	2.427	2.69	2.782	0.955	1.374	1.066	-1.005937339	0.0000101
LOC_Os07g25900	0.301	0.251	0.334	0.642	0.542	0.729	1.0043193	0.000525781
LOC_Os04g53050	0.09	0.053	0.247	0.502	0.334	0.247	1.002707436	0.005989327
LOC_Os03g62780	1.315	0.977	1.482	0.404	0.357	0.659	-1.002675555	0.001601205
LOC_Os06g32160	222.729	210.429	183.311	375.482	384.392	392.248	1.002565888	1.56E-73
LOC_Os03g53970	2.814	1.568	1.559	1.018	0.172	0.899	-1.002440773	0.003378804
LOC_Os02g48200	1.286	1.998	1.24	0.706	0.585	0.58	-1.002172955	0.0000891
LOC_Os09g21510	3.851	3.702	4.329	1.459	1.995	1.861	-1.002082587	2.88E-09
LOC_Os07g08960	17.449	14.954	13.746	7.68	6.945	6.443	-1.001908073	2.03E-19
LOC_Os12g39980	2.603	2.378	2.07	0.998	1.084	1.122	-1.001687822	1.83E-15
LOC_Os02g28900	5.867	6.158	5.263	2.815	2.233	2.688	-1.000867913	9.95E-10
LOC_Os03g19270	75.805	71.481	74.987	133.388	137.078	143.113	1.000863585	5.27E-79
LOC_Os09g27820	1.428	0.73	1.128	0.238	0.61	0.265	-0.999955622	0.003547758
LOC_Os08g16359	2.78	2.629	3.708	0.975	1.236	1.482	-0.999730718	0.000408888
LOC_Os11g04409	260.37	253.827	265.382	482.107	511.622	457.794	0.999240757	8.72E-141
LOC_Os04g44420	0.985	0.732	1.694	1.901	2.89	3.777	0.998853903	0.004303366
LOC_Os02g13370	7.65	6.783	8.92	13.797	14.061	16.32	0.998737528	1.14E-17
LOC_Os01g02530	0.93	0.903	0.975	1.552	1.86	1.969	0.996567149	3.36E-10
LOC_Os01g42350	0.513	0.56	0.374	0.203	0.206	0.154	-0.996456846	0.001192849
LOC_Os01g53820	1.836	0.91	3.03	3.896	5.565	4.636	0.995584105	0.00353082
LOC_Os04g54220	29.989	32.763	32.514	63.032	59.206	56.562	0.995481652	2.64E-24
LOC_Os02g03120	1.312	1.999	1.418	2.66	3.913	2.851	0.995253991	0.0000069
LOC_Os07g05850	0.407	0.446	0.314	1.053	0.796	0.676	0.995131837	0.000741099
LOC_Os07g34260	4.101	5.124	3.793	2.133	1.534	2.059	-0.993322158	0.000000751
LOC_Os02g51590	13.949	12.088	11.795	5.565	6.358	5.423	-0.993054371	4.77E-16
LOC_Os04g11390	7.042	6.446	6.565	14.228	11.941	12.371	0.992873821	6.54E-10
LOC_Os03g08500	125.983	127.292	123.122	56.196	54.203	64.507	-0.992841174	1.52E-61
LOC_Os04g58200	6.175	6.598	6.671	3.251	2.729	2.789	-0.992748498	1.58E-09
LOC_Os05g38264	7.522	8.12	6.176	13.593	12.954	14.733	0.991616376	1.72E-12
LOC_Os11g14330	0.086	0.114	0.254	0.442	0.301	0.405	0.991367029	0.004182296

LOC_Os06g34090	0.022	0.111	0.117	0.093	0.282	0.381	0.989934921	0.007485283
LOC_Os11g02240	60.422	61.488	59.501	111.291	114.672	109.748	0.989562036	1.61E-117
LOC_Os09g31019	7.83	12.48	8.349	4.269	4.827	3.454	-0.989033751	0.00000476
LOC_Os04g09800	1.907	1.821	2.142	0.895	0.689	1.007	-0.988745588	0.000000919
LOC_Os03g19000	14.926	13.792	14.898	28.033	29.501	24.107	0.988426588	8.52E-22
LOC_Os06g39480	5.596	5.385	5.101	2.287	2.634	2.473	-0.988292945	5.65E-16
LOC_Os02g57280	6.192	6.112	4.85	9.954	9.616	12.801	0.988048242	3.45E-12
LOC_Os04g48870	1.271	1.723	1.104	0.258	0.719	0.362	-0.987355881	0.006050046
MSTRG.2782	5.089	7.124	8.38	13.597	11.908	14.88	0.986991133	3.22E-08
LOC_Os08g28010	0.416	0.126	0.28	0.815	0.631	0.538	0.986948083	0.003635715
LOC_Os10g32810	3.773	3.738	3.174	6.888	5.72	7.516	0.98654802	8.08E-13
LOC_Os07g47090	2.497	2.114	1.707	1.095	0.488	0.951	-0.986083201	0.000731351
LOC_Os03g19250	296.704	287.321	324.842	589.8	522.425	577.921	0.985934125	1.41E-34
LOC_Os11g42370	2.248	2.374	2.197	0.889	1.045	0.964	-0.985889029	0.0000802
LOC_Os04g44510	41.6	45.361	45.81	82.099	81.515	82.091	0.985617387	3.39E-56
LOC_Os06g10950	0.855	0.651	0.452	0.19	0.321	0.119	-0.982454159	0.006511018
LOC_Os06g45140	42.987	39.474	42.186	74.688	75.863	79.019	0.982163011	1.27E-69
LOC_Os05g33030	6.617	5.86	6.049	2.9	2.939	2.711	-0.981787267	4.08E-15
LOC_Os08g43334	2.816	2.306	2.96	5.505	4.369	5.655	0.981698441	6.65E-08
LOC_Os01g53779	0.2	0.254	0.34	0.578	0.474	0.901	0.981254256	0.004410612
LOC_Os09g13575	52.414	55.168	70.457	113.267	112.231	105.583	0.981221429	1.09E-25
LOC_Os10g37340	25.982	27.238	25.995	13.003	11.848	12.172	-0.980566551	1.76E-36
LOC_Os12g09640	4.406	3.763	3.1	7.329	6.277	7.742	0.980455135	1.5E-09
LOC_Os06g05910	30.468	27.432	38.952	63.52	59.508	60.049	0.979939848	9.2E-11
LOC_Os07g34640	32.594	34.251	31.919	63.069	58.163	63.503	0.97896404	2.33E-60
LOC_Os01g15900	1.887	2.285	2.427	4.297	4.023	4.23	0.977998814	2.97E-09
LOC_Os03g40210	3.92	5.687	5.905	8.914	13.142	9.091	0.9774972	0.0000419
LOC_Os07g46020	0.108	0.051	0.038	0.155	0.271	0.233	0.977441582	0.009314331
LOC_Os08g04210	1640.014	1618.029	1866.713	3286.799	3005.947	3173.835	0.977000858	8.92E-34
LOC_Os04g28120	18.759	16.74	17.971	35.15	31.807	31.994	0.976164418	5.04E-24
LOC_Os10g32658	0.372	0.108	0.33	0.892	0.508	1.065	0.975884558	0.007651764
LOC_Os11g06210	1.773	1.522	1.409	2.926	4.16	2.309	0.975312698	0.000038
LOC_Os10g27980	0.717	1.158	1.141	0.509	0.148	0.408	-0.975085337	0.004298219
LOC_Os04g40650	9.285	9.99	10.402	4.72	4.102	4.779	-0.974603672	1.75E-09
LOC_Os10g20450	27.535	27.152	25.967	11.732	13.43	12.683	-0.973942568	2.47E-32

LOC_Os06g39270	9.669	9.813	9.788	3.757	4.038	5.662	-0.973299425	8.64E-12
LOC_Os05g33600	23.12	25.813	23.722	46.454	43.426	43.457	0.973249904	3.45E-49
LOC_Os08g40680	103.057	100.162	108.03	199.407	187.416	183.211	0.973062353	4.77E-81
LOC_Os06g32240	27.721	28.719	26.274	50.225	50.598	51.185	0.972898549	2.59E-37
LOC_Os12g34510	65.896	65.773	62.46	31.484	28.101	31.567	-0.972660797	2.3E-34
LOC_Os08g14109	3.027	3.202	3.528	1.584	1.455	1.157	-0.971993591	0.0000935
LOC_Os10g39920	13.753	13.293	17.448	27.89	24.537	30.175	0.971847179	3.73E-17
LOC_Os09g25720	8.111	7.891	7.637	16.354	13.575	14.432	0.971685094	6.7E-11
LOC_Os04g48350	2.883	3.186	2.999	1.123	1.464	1.25	-0.971453765	0.00020283
LOC_Os08g14890	1.739	2.154	1.595	4.09	3.933	3.726	0.970576123	0.00096743
LOC_Os10g31420	233.024	239.367	276.293	473.369	477.479	427.49	0.970322218	1.57E-26
LOC_Os03g55310	10.38	10.358	10.868	5.398	4.247	4.714	-0.969243778	4.78E-08
LOC_Os01g24780	0.517	0.544	1.007	1.496	1.706	1.195	0.969166353	0.000675201
LOC_Os02g07490	55.864	51.679	52.791	23.136	28.27	24.341	-0.968787289	1.85E-42
LOC_Os12g15530	0.684	1.332	0.722	1.799	2.006	1.607	0.968664821	0.0000292
LOC_Os09g26960	45.078	48.243	40.092	21.362	22.284	19.308	-0.968421854	1.06E-37
LOC_Os04g33240	8.663	6.889	9.663	4.152	3.777	3.533	-0.968421852	8.38E-08
LOC_Os05g27100	4.401	5.027	3.867	2.477	1.674	1.599	-0.968377138	0.0000651
LOC_Os05g26368	12.577	13.621	16.432	27.607	29.234	22.816	0.968130403	1.79E-12
LOC_Os01g01290	0.225	0.186	0	0.362	0.587	0.474	0.967723916	0.009532083
LOC_Os02g55890	374.606	370.395	364.94	694.164	664.06	662.125	0.967428111	1.7E-181
LOC_Os04g36750	2.043	2.392	2.498	4.479	3.693	5.528	0.967416277	0.0000336
LOC_Os09g37180	11.908	12.134	14.359	5.608	5.96	6.413	-0.966146874	8.83E-18
LOC_Os08g39840	2.385	2.034	1.465	0.853	0.72	1.008	-0.966060961	0.00000762
LOC_Os01g56940	2.375	2.038	1.663	0.92	0.987	0.81	-0.965160865	0.00000287
LOC_Os12g07980	0.739	0.976	0.903	1.782	2.89	2.111	0.964922839	0.006249802
LOC_Os05g51620	0.796	0.847	0.865	0.255	0.205	0.482	-0.964029293	0.000646004
LOC_Os12g31810	4.333	4.683	4.256	2.144	2.036	1.903	-0.963907362	0.000000011
LOC_Os04g31210	14.139	12.558	10.834	4.923	5.968	6.61	-0.963677177	2.42E-14
LOC_Os02g52730	0.686	0.549	1.064	1.426	1.756	1.43	0.963111671	0.00018393
LOC_Os07g43260	5.373	3.099	3.162	1.934	1.725	1.085	-0.962912805	0.000876478
LOC_Os11g08380	28.839	29.928	26.478	13.855	12.364	13.973	-0.962888302	1.04E-26
LOC_Os01g43750	15.919	16.899	13.762	7.052	7.307	7.501	-0.96273154	7.83E-19
LOC_Os01g21590	24.787	22.765	23.117	44.434	39.065	45.352	0.962584779	2.46E-37
LOC_Os03g12940	4.506	5.036	4.992	1.799	1.674	2.784	-0.962131379	0.000000068

LOC_Os06g05420	5.798	4.801	6.19	13.103	9.165	9.743	0.961810565	0.000000351
LOC_Os01g65650	18.984	19.395	19.775	37.832	34.669	33.228	0.960744983	4.43E-61
LOC_Os01g62010	2.125	1.551	1.692	4.1	4.156	2.522	0.957958819	0.000137765
LOC_Os02g45780	22.317	20.659	22.478	41.821	38.805	38.561	0.957757276	8.71E-34
LOC_Os06g48680	1.471	2.027	1.466	3.455	2.939	3.633	0.957610578	0.000229772
LOC_Os06g45890	7.944	8.405	9.546	16.926	14.253	16.38	0.956988034	5.02E-17
LOC_Os08g01950	4.066	4.469	4.66	1.747	2.143	2.149	-0.956597098	0.000000105
LOC_Os01g73630	3.165	2.605	3.036	1.55	1	1.23	-0.956375624	0.000175355
LOC_Os12g10870	6.302	6.585	6.375	12.621	11.369	11.125	0.955844655	2.56E-25
LOC_Os12g36430	0.303	0.221	0.19	0.108	0.018	0.072	-0.955112711	0.00936265
LOC_Os04g50700	6.935	7.742	6.198	3.218	3.09	2.93	-0.955060419	0.0000241
LOC_Os03g01590	1.398	1.148	0.855	0.445	0.235	0.65	-0.954920261	0.002507368
LOC_Os07g47620	13.123	12.631	14.111	24.524	24.093	23.899	0.954744852	8.57E-34
LOC_Os12g03180	3.096	3.132	2.339	1.393	1.271	1.184	-0.954429342	0.00000168
LOC_Os10g11500	17.933	18.081	16.877	6.326	9.27	8.978	-0.954319833	3.92E-10
LOC_Os04g54260	0.264	0.189	0.149	0.527	0.507	0.25	0.953747514	0.001152225
LOC_Os12g04204	157.935	154.127	170.201	284.376	297.801	280.408	0.953524374	7.09E-120
LOC_Os12g38720	2.282	3.339	2.093	4.83	5.871	4.463	0.953350188	0.0000681
LOC_Os10g25450	4.415	4.883	3.653	1.981	1.747	2.26	-0.953057856	2.21E-09
LOC_Os02g11790	11.202	11.516	14.793	22.315	21.571	24.689	0.953054581	1.5E-19
LOC_Os06g05440	20.289	19.13	22.263	42.052	32.96	37.602	0.952800926	1.29E-19
LOC_Os12g07830	5.151	4.973	4.239	1.784	2.368	2.343	-0.951046413	0.00000235
LOC_Os02g55570	3.048	2.785	2.984	1.484	1.273	1.228	-0.950450142	0.00000364
LOC_Os04g20400	4.031	3.089	2.829	1.185	1.416	1.846	-0.950192228	0.00000929
LOC_Os11g18044	11.056	10.226	9.511	4.941	4.417	5.187	-0.948620563	4.13E-16
LOC_Os11g14240	1.404	1.257	0.817	0.625	0.369	0.414	-0.948544904	0.001248651
LOC_Os01g12860	3.809	4.293	3.733	1.83	1.794	1.933	-0.947432112	4.51E-12
LOC_Os05g39310	181.136	184.213	184.174	335.98	321.528	328.795	0.946831751	1.75E-147
LOC_Os04g14680	1.505	1.594	1.427	2.954	3.197	2.808	0.946229794	0.00011802
LOC_Os02g18430	4.377	3.523	3.762	1.734	2.063	1.48	-0.945638685	0.00000623
LOC_Os09g32944	2.386	1.957	2.037	1.072	0.523	1.076	-0.945429678	0.000573944
LOC_Os11g42989	1.653	1.351	1.728	2.574	3.12	3.296	0.94469009	0.00000214
LOC_Os05g15150	3.154	2.94	2.892	1.555	1.142	1.517	-0.944104587	3.81E-11
LOC_Os11g07120	18.878	17.913	17.559	33.323	32.891	31.419	0.943964988	4.5E-60
LOC_Os11g39260	0.484	0.8	0.592	1.558	1.737	1.384	0.943863144	0.006053118

LOC_Os03g36560	7.878	6.955	6.794	3.047	3.813	3.236	-0.943269273	3.42E-09
LOC_Os02g52150	6.656	6.794	7.69	14.224	11.847	12.428	0.943253971	4.56E-16
LOC_Os12g25090	189.429	190.306	217.135	345.112	390.395	337.296	0.942742737	1E-53
LOC_Os11g02080	339.283	348.317	347.207	622.522	615.785	613.585	0.942461114	1.05E-173
LOC_Os02g18650	21.237	20.612	21.863	39.747	34.252	40.581	0.942277628	1.83E-36
LOC_Os12g06660	6.89	6.645	6.216	2.758	2.974	3.471	-0.941883808	4.49E-09
LOC_Os03g58580	9.342	8.661	8.129	3.938	4.335	4.134	-0.941641166	3.18E-15
LOC_Os06g44034	60.375	56.441	69.613	114.038	108.833	111.564	0.940748889	2.13E-53
LOC_Os07g24000	20.302	19.282	22.818	41.564	33.941	37.128	0.940568579	3.55E-24
LOC_Os03g51530	15.914	15.856	17.296	26.997	30.073	32.173	0.940340263	3.06E-15
LOC_Os09g31040	144.388	140.795	146.015	268.487	260.306	242.86	0.940117825	3.04E-85
LOC_Os08g35440	2.229	1.948	1.683	0.97	0.769	0.709	-0.940105275	0.000783614
LOC_Os07g11150	0.568	0.631	0.575	1.122	1.18	1.124	0.939553534	0.000119113
LOC_Os04g20080	1.073	1.37	0.942	2.132	2.626	1.668	0.939232434	0.00000301
LOC_Os06g03520	267.859	261.159	287.407	497.807	480.719	481.497	0.938405326	5.6E-107
LOC_Os08g18974	8.5	7.778	8.588	14.122	12.722	12.867	0.936386648	1.17E-15
LOC_Os01g69200	2.652	2.697	1.696	0.976	1.16	0.944	-0.934374183	0.000174125
LOC_Os04g40940	9.067	10.599	11.39	5.023	5.323	4.473	-0.934309382	1.32E-09
LOC_Os09g13570	105.989	98.593	113.612	199.516	188.354	179.545	0.933829074	2.03E-68
LOC_Os02g51760	0.576	0.714	0.713	1.056	1.578	1.429	0.933457494	0.000514527
LOC_Os04g01690	279.187	269.829	258.8	124.315	131.083	137.124	-0.93301569	6.19E-106
MSTRG.24598	163.044	170.01	227.282	372.002	269.886	433.164	0.93277009	0.0000314
LOC_Os06g43220	1.013	0.803	0.929	0.195	0.481	0.392	-0.932416432	0.002984488
LOC_Os07g02880	81.942	77.939	94.067	155.371	149.754	148.278	0.932328339	2.58E-46
LOC_Os03g39110	2.014	1.885	2.463	3.421	3.327	5.353	0.932299919	0.0000106
LOC_Os08g10250	0.236	0.359	0.216	0.637	0.523	0.593	0.931943255	0.001754276
LOC_Os02g10830	0.651	0.774	0.477	1.381	1.464	1.585	0.931548777	0.004662121
LOC_Os06g02580	4.387	3.05	4.152	1.946	1.419	1.915	-0.931176497	0.00000771
LOC_Os06g05300	0.755	0.433	0.364	1.533	1.01	0.931	0.930569473	0.003410256
LOC_Os09g28730	4.679	6.53	5.148	2.473	2.904	2.202	-0.92978948	0.000000943
LOC_Os02g44990	13.048	14.805	14.491	26.029	21.794	27.827	0.929485025	2.32E-27
LOC_Os07g44930	4.146	6.162	3.679	1.871	2.223	2.051	-0.92925714	0.000170088
LOC_Os03g55670	55.44	52.36	59	96.039	103.095	98.287	0.929042231	3.07E-40
LOC_Os03g04020	27.533	25.622	28.949	51.139	45.153	49.939	0.929030513	5.01E-38
LOC_Os04g33190	42.861	45.245	45.724	80.465	75.937	81.179	0.928929205	2.16E-82

LOC_Os08g34330	0.449	0.405	0.187	1.143	0.679	0.737	0.92706053	0.006346265
LOC_Os01g13040	9.093	8.422	7.189	4.068	2.899	4.398	-0.926915359	0.00000201
LOC_Os02g12690	8.808	9.09	8.552	3.894	3.917	4.799	-0.926863859	1.57E-12
LOC_Os11g03430	2.194	2.33	2.245	0.852	0.948	1.268	-0.926612951	0.0000133
LOC_Os09g30190	3.692	3.924	3.009	1.553	1.605	1.864	-0.926230349	1.02E-09
LOC_Os04g28420	5.027	4.72	5.237	9.066	8.674	9.211	0.926208545	6.42E-16
LOC_Os07g11920	85.452	79.994	112.675	192.905	174.947	147.471	0.92580224	0.000000289
LOC_Os05g10940	12.086	13.379	13.126	22.883	23.194	22.671	0.925374714	1.46E-30
LOC_Os01g73200	22.561	22.087	22.581	12.01	10.02	10.458	-0.923563728	3.29E-19
LOC_Os01g33080	3.246	3.376	2.765	1.31	1.823	1.246	-0.92199378	0.000000804
LOC_Os04g49370	11.091	11.915	12.367	21.557	21.174	20.373	0.921633488	5.14E-22
LOC_Os09g11440	1.178	1.935	1.023	0.658	0.394	0.643	-0.921528547	0.001569212
LOC_Os11g09160	5.828	6.112	5.937	10.344	10.06	11.148	0.920840257	4.28E-14
LOC_Os10g39640	9.501	8.503	9.164	17.104	15.368	16.022	0.920646089	3.05E-19
LOC_Os05g33554	3.025	3.554	2.838	4.75	5.64	7.486	0.920268358	0.0000325
LOC_Os02g15150	64.893	61.592	73.25	125.717	124.112	106.66	0.9201024	4.25E-19
LOC_Os05g07000	2.862	2.117	2.939	1.141	1.492	0.895	-0.91877957	0.000172802
LOC_Os03g63300	7.006	5.627	5.683	3.115	2.876	2.73	-0.917797194	1.74E-09
LOC_Os06g28970	11.465	10.384	10.448	4.682	4.677	5.836	-0.917646651	8.85E-08
LOC_Os03g45760	5.45	6.798	5.86	2.459	2.914	3.147	-0.917489076	0.000000126
LOC_Os02g14720	11.246	10.58	10.946	20.244	20.472	17.388	0.916408125	8.01E-27
LOC_Os07g47670	33.55	40.143	35.706	64.841	67.674	61.243	0.915489204	5.49E-27
LOC_Os08g07010	1.223	1.368	0.85	0.514	0.694	0.267	-0.91457192	0.000815758
LOC_Os05g48270	4.771	5.397	3.617	1.921	2.02	2.44	-0.91457023	0.00000387
LOC_Os07g41310	4.787	6.634	6.758	11.361	10.855	10.356	0.914295451	1.4E-13
LOC_Os04g45890	0.355	0.44	0.448	0.986	0.739	1.202	0.914061781	0.005566784
LOC_Os03g45280	62.673	62.847	59.199	106.129	111.582	107.13	0.9135125	1.66E-56
LOC_Os01g42030	6.954	7.412	7.394	12.826	13.115	12.702	0.913415892	1.28E-19
LOC_Os07g01840	1.236	1.571	1.176	0.675	0.479	0.589	-0.912990781	0.000370088
LOC_Os04g42540	1.858	2.038	2.251	0.832	0.843	0.778	-0.912584769	0.002886952
LOC_Os11g08569	1.79	2.853	1.92	3.864	4.622	3.525	0.912165202	0.000000087
LOC_Os06g47130	2.56	1.865	2.326	4.462	3.78	4.052	0.911996726	5.47E-08
LOC_Os01g16714	1.983	2.8	1.856	0.814	0.949	1.103	-0.910615367	0.000621764
LOC_Os10g24004	5.096	4.255	5.606	8.076	10.25	9.191	0.909924828	0.000000621
LOC_Os04g25440	72.586	75.126	71.797	34.38	34.738	38.987	-0.909730562	2.84E-58

LOC_Os03g04310	3.571	3.469	4.228	1.706	1.932	1.626	-0.909234617	0.00000419
LOC_Os03g05070	3.158	3.284	2.064	1.237	1.223	1.41	-0.908840619	0.000045
LOC_Os03g18350	2.763	2.527	2.162	1.044	1.342	1.115	-0.908664024	0.00000115
LOC_Os09g25620	82.218	80.565	77.773	38.268	41.999	38.554	-0.908545439	2.1E-70
LOC_Os07g42910	5.696	4.553	5.4	9.803	9.493	9.165	0.908494331	0.000000056
LOC_Os08g15444	6.795	6.271	7.096	12.131	11.04	12.189	0.908327653	1.63E-19
LOC_Os01g59510	3.64	3.473	3.338	1.587	1.74	1.605	-0.908296218	0.000000471
LOC_Os09g20400	8.63	8.109	9.249	3.909	4.12	4.458	-0.908176754	5.93E-10
LOC_Os01g69870	22.01	21.69	26.443	40.542	40.543	43.135	0.90817413	9.21E-20
LOC_Os01g18080	2.305	1.394	1.976	3.495	3.132	3.861	0.907889179	0.00000334
LOC_Os04g46630	22.159	21.909	19.449	38.233	39.077	34.505	0.907874708	4.87E-27
LOC_Os09g31025	24.46	22.707	25.511	43.611	40.914	43.615	0.907274879	7.89E-24
LOC_Os04g55850	12.171	15.422	12.76	6.455	6.911	6.123	-0.90714465	2.84E-11
LOC_Os02g13780	1.043	1.292	0.973	1.797	1.967	2.335	0.907123428	0.0000032
LOC_Os04g46660	6.502	5.406	6.503	2.72	3.157	2.83	-0.906998119	0.000000483
LOC_Os07g11650	20.488	17.099	21.95	42.295	32.888	30.731	0.905137507	2.01E-13
LOC_Os01g49614	5.818	4.887	5.386	10.829	9.677	9.962	0.90442729	5.27E-13
LOC_Os01g18970	5.174	6.297	5.312	3.093	2.805	1.877	-0.904336335	0.0000199
LOC_Os03g15230	1.138	1.458	1.578	2.571	2.742	2.454	0.903499973	0.00000906
LOC_Os10g18340	1.636	1.102	1.219	0.758	0.32	0.236	-0.903332429	0.008136368
LOC_Os01g37832	38.451	37.413	47.873	68.487	68.596	65.401	0.902823778	3.71E-33
LOC_Os05g32860	19.022	14.678	21.861	10.233	6.514	9.645	-0.90240894	0.000000215
LOC_Os05g44922	203.33	205.499	209.462	377.231	355.959	342.914	0.902025677	5.15E-109
LOC_Os01g17330	25.394	24.413	22.038	11.497	11.998	11.746	-0.90067536	3.6E-18
LOC_Os02g15930	4.804	4.36	5.312	9.269	7.889	8.665	0.899750856	1.05E-10
LOC_Os05g41220	29.241	29.047	35.024	55.88	54.686	52.769	0.899526105	1.04E-36
LOC_Os07g41340	91.586	85.736	92.242	153.475	166.9	149.916	0.899426927	1.47E-46
LOC_Os08g32140	0.735	0.605	0.247	1.122	1.272	1.02	0.899005052	0.002543112
LOC_Os09g20180	1.096	1.969	1.759	2.71	2.479	4.573	0.898654076	0.001256558
LOC_Os12g39210	3.876	5.674	5.955	2.459	2.049	2.803	-0.898557355	0.00000212
LOC_Os02g02140	1.747	1.412	1.135	0.851	0.464	0.627	-0.898537045	0.000126354
LOC_Os08g04360	5.317	3.056	4.29	2.257	1.456	1.535	-0.898535091	0.002074835
MSTRG.2029	1.294	1.485	1.686	3.023	2.798	3.072	0.898448432	0.000789718
LOC_Os11g24060	3.828	3.534	3.51	1.455	1.418	2.223	-0.898260888	0.00000369
LOC_Os02g11760	68.088	64.954	63.714	114.284	114.733	112.451	0.897825374	4.98E-128

LOC_Os10g05250	0.577	0.477	0.507	0.882	1.317	0.933	0.896910908	0.00098227
LOC_Os10g38600	3.823	2.706	3.672	2.166	1.068	1.204	-0.896206376	0.00079467
LOC_Os10g42190	34.194	31.032	33.292	56.869	56.189	58.056	0.895638175	2.43E-58
LOC_Os10g37830	50.162	47.598	51.071	88.6	87.923	84.449	0.894523429	2.28E-59
LOC_Os01g67430	1.007	0.959	1.1	0.224	0.492	0.454	-0.89415446	0.00503904
LOC_Os03g53920	7.527	8.035	7.321	3.483	4.101	3.72	-0.894082767	5.46E-20
LOC_Os08g09950	17.885	16.734	16.25	8.564	7.444	8.98	-0.893936713	8.43E-20
LOC_Os04g06590	1.583	1.326	1.348	2.782	2.525	2.38	0.893873768	0.000000243
LOC_Os08g03420	29.349	27.712	28.426	51.249	47.198	49.789	0.893424555	3.76E-49
LOC_Os06g24070	3.413	3.295	3.534	1.93	1.64	1.274	-0.893124541	0.0000043
LOC_Os03g07990	14.375	16.266	15.199	26.461	27.839	25.641	0.89240978	3.93E-25
LOC_Os04g53230	22.443	21.523	22.558	9.765	11.714	11.454	-0.891641659	8.89E-21
LOC_Os11g32500	5.759	7.217	5.229	2.615	2.761	3.157	-0.89146747	0.00000983
LOC_Os06g01210	9.877	11.529	9.49	5.286	4.101	5.369	-0.891369266	0.000000322
LOC_Os05g33890	2.884	4.032	2.881	1.416	1.335	1.858	-0.890007337	0.00000604
LOC_Os11g38260	1.8	1.435	1.811	3.007	2.962	3.484	0.889390705	0.0000712
LOC_Os10g22520	445.669	436.849	449.776	762.674	739.495	795.678	0.889164041	3.23E-132
LOC_Os08g16100	0.911	0.441	0.847	1.147	1.479	1.581	0.88894299	0.000128839
LOC_Os01g54420	4.039	3.462	3.146	1.716	1.616	1.793	-0.888804131	7.18E-08
LOC_Os03g44140	1.396	0.793	0.999	1.856	2.297	1.704	0.888734569	0.0000105
LOC_Os10g25060	2.508	2.405	3.005	1.21	1.029	1.35	-0.888281492	0.000214066
LOC_Os05g49730	9.828	9.39	11.478	17.833	18.266	17.587	0.888221857	8.62E-18
LOC_Os02g36830	18.861	22.351	19.19	7.658	11.212	10.922	-0.887976085	1.3E-14
LOC_Os03g14050	944.84	938.561	1102.81	1772.193	1624.454	1810.839	0.887767975	1.86E-21
LOC_Os03g25440	0.789	1.061	0.672	1.685	1.984	1.626	0.887461063	0.002780349
LOC_Os09g26920	0.698	0.895	0.772	1.267	1.546	1.611	0.886736352	0.0000783
LOC_Os10g13700	59.79	57.723	73.042	116.311	113.267	104.012	0.886083156	3.04E-14
LOC_Os03g45250	70.303	66.086	69.801	126.216	116.161	113.26	0.885857827	6.16E-60
LOC_Os05g19620	2.341	3.262	2.65	1.553	0.751	1.419	-0.88577706	0.000258902
LOC_Os05g08900	14.117	13.875	14.079	23.326	23.422	26.242	0.885664324	7.61E-24
LOC_Os12g34890	16.766	18.002	17.455	8.506	9.144	8.013	-0.8854145	2.29E-11
LOC_Os02g39910	0.405	0.458	0.493	1.064	0.655	0.799	0.885364449	0.0000458
LOC_Os02g09240	18.933	19.077	17.994	10.118	8.158	9.49	-0.88496787	2.41E-18
LOC_Os12g10340	3.874	2.668	2.922	1.325	1.478	1.66	-0.884880001	0.00000937
LOC_Os01g01302	5.914	4.257	3.847	2.243	2.224	2.051	-0.884424351	0.0000464

LOC_Os10g28230	25.095	25.613	25.086	11.713	13.065	12.836	-0.883774842	3.55E-17
LOC_Os06g45610	11.039	12.621	14.144	18.433	22.696	25.465	0.883727232	4.75E-11
LOC_Os06g42000	33.472	34.344	32.075	15.726	16.835	17.262	-0.883619875	1.5E-25
LOC_Os07g09280	1.565	1.908	0.773	0.871	0.235	0.326	-0.882946216	0.008316872
LOC_Os01g63210	16.985	16.406	15.517	8.163	8.334	7.577	-0.882754321	1.5E-11
MSTRG.25211	22.751	23.396	27.77	41.576	42.821	40.962	0.882734774	4.38E-69
LOC_Os01g45860	17.013	16.512	17.066	28.463	26.604	32.446	0.882532816	6.19E-25
LOC_Os11g04380	0.927	0.671	0.501	0.372	0.191	0.099	-0.882514817	0.009027483
LOC_Os10g02300	2.787	2.176	3.573	5.657	5.31	4.889	0.881635848	0.0000834
LOC_Os07g38640	0.89	0.992	1.071	0.402	0.408	0.426	-0.880129077	0.002376651
LOC_Os09g21290	5.083	5.695	5.749	1.879	2.88	3.085	-0.879501746	0.00000827
LOC_Os02g16820	84.09	88.562	101.307	171.042	163.273	142.351	0.879470351	1.92E-15
LOC_Os09g15510	5.992	6.728	6.04	3.163	3.37	2.468	-0.879413606	0.00000245
LOC_Os10g31500	6.319	6.956	5.744	3.23	2.271	3.507	-0.879385923	0.00000735
LOC_Os12g36750	3.577	2.949	3.6	5.894	6.852	6.482	0.879176441	2.79E-09
LOC_Os06g16170	0.944	1.013	1.117	2.124	1.499	2.34	0.878692086	0.000664249
LOC_Os03g48770	164.327	159.723	146.191	77.406	78.53	80.578	-0.87854582	1.82E-52
LOC_Os12g10910	1.013	0.845	0.88	2.006	1.512	2.068	0.878444704	0.00213743
LOC_Os10g26720	0.399	0.198	0.183	0.481	0.747	0.689	0.878265944	0.008085851
LOC_Os03g32220	1.461	1.344	1.196	0.654	0.459	0.423	-0.87823913	0.005452178
LOC_Os11g29920	0.761	0.798	0.989	1.49	1.519	1.574	0.877821177	0.00000122
LOC_Os02g56140	6.289	4.692	5.495	10.137	9.261	9.476	0.87779441	6.19E-11
LOC_Os03g63480	15.455	11.868	13.928	6.27	6.171	7.907	-0.87771121	1.25E-11
LOC_Os10g31440	110.851	113.745	115.366	181.109	213.614	188.864	0.877629573	1.14E-57
LOC_Os07g03120	27.241	26.657	26.565	14.44	12.001	13.717	-0.87726754	3.12E-19
LOC_Os09g28580	3.816	5.519	5.485	8.956	10.691	7.721	0.877015688	0.0000383
LOC_Os04g41110	5.554	5.004	6.36	10.047	9.057	10.263	0.876813669	3.6E-16
LOC_Os05g33140	119.348	107.689	97.074	52.471	55.446	55.313	-0.87524096	1.23E-39
LOC_Os07g47790	363.816	367.952	392.822	658.994	620.71	642.485	0.875061807	7.91E-98
LOC_Os02g32590	8.571	7.663	8.705	13.692	13.748	15.693	0.874943094	5.89E-19
LOC_Os03g58880	6.032	6.291	5.21	9.555	10.399	10.479	0.874291949	2.4E-13
LOC_Os06g40020	0.331	0.268	0.441	0.929	0.853	0.543	0.873822329	0.005260057
LOC_Os07g02900	13.609	12.135	10.98	21.925	21.424	21.479	0.872273808	4.67E-08
LOC_Os07g44480	12.817	14.481	10.904	5.945	6.5	6.438	-0.872229237	3.31E-11
LOC_Os06g48240	10.236	9.398	7.79	4.345	4.004	5.139	-0.871634742	1.07E-09

LOC_Os11g02450	2.828	3.39	2.592	1.407	1.175	1.292	-0.871623745	0.000916015
LOC_Os05g50690	1.249	0.679	1.034	0.428	0.394	0.291	-0.871515221	0.0065119
LOC_Os02g11980	2.458	2.555	1.836	3.98	3.858	4.115	0.870432618	8.71E-10
LOC_Os07g01490	7.043	7.15	6.398	3.384	3.299	3.572	-0.870333566	8.45E-13
MSTRG.5284	3.654	3.896	3.933	5.824	7.767	6.698	0.87013303	4.42E-08
LOC_Os02g09070	3.508	3.062	2.604	0.645	1.715	1.431	-0.868737761	0.003222047
LOC_Os01g58444	3.6	3.785	3.727	7.229	5.963	6.14	0.868372559	4.11E-11
LOC_Os07g32406	0.135	0.214	0.446	0.599	0.687	0.585	0.8682498	0.007026808
LOC_Os01g71780	1.187	1.143	0.684	0.229	0.63	0.306	-0.86765612	0.005677084
LOC_Os04g33760	1.582	1.822	1.02	3.425	1.924	3.047	0.867394849	0.000509812
LOC_Os09g39490	4.732	5.176	5.296	2.134	2.764	2.272	-0.866991973	0.0000599
LOC_Os11g09474	1.418	1.386	1.716	2.28	3.159	2.629	0.866706702	0.00000108
LOC_Os04g47360	1.597	1.792	1.658	0.613	0.847	0.92	-0.866116534	0.0000525
LOC_Os03g31360	25.328	23.537	25.698	41.516	45.155	40.6	0.866073816	6.22E-37
LOC_Os07g49350	0.276	0.273	0.295	0.754	0.539	0.912	0.864360275	0.009741735
LOC_Os07g11310	22.11	16.492	18.678	33.953	33.119	31.592	0.863377056	3.61E-14
LOC_Os08g04250	1832.265	1813.163	2220.018	3581.993	3202.531	3304.136	0.863082381	5.89E-15
LOC_Os02g16830	42.022	38.938	51.544	81.272	82.944	67.115	0.863044736	3.86E-09
LOC_Os05g49820	3.398	2.686	3.018	1.246	1.621	1.56	-0.862838664	0.000000789
LOC_Os08g43430	5.553	4.353	3.932	2.272	2.131	2.282	-0.862724443	0.00000344
LOC_Os03g07600	43.987	43.115	43.149	70.87	74.787	75.194	0.862213044	8.95E-73
LOC_Os01g72009	113.99	116.841	120.745	205.795	204.307	186.587	0.861200661	1.59E-52
LOC_Os03g49350	20.226	23.204	21.566	10.225	11.338	11.401	-0.86050069	3.09E-25
LOC_Os02g41670	48.162	47.988	49.433	24.527	24.286	25.432	-0.860435032	6.87E-57
LOC_Os02g54254	57.251	57.18	61.576	97.404	102.31	98.337	0.860381782	1.1E-95
LOC_Os07g02340	17.464	22.248	22.646	35.141	46.353	29.702	0.860375884	0.00000192
LOC_Os02g29140	9.451	7.804	8.635	14.31	15.563	14.473	0.859721558	7.94E-17
LOC_Os02g17900	1.466	2.575	1.65	0.386	0.912	0.902	-0.859182474	0.005947558
LOC_Os09g01360	1.669	1.554	1.377	0.676	0.767	0.752	-0.85721546	0.0000327
LOC_Os07g26940	35.712	34.143	32.848	57.357	60.654	56.292	0.857054773	1.32E-31
LOC_Os03g16709	1.925	1.908	1.387	0.73	0.605	0.682	-0.856895047	0.006084901
LOC_Os01g67540	4.244	3.807	3.418	1.964	1.654	1.965	-0.856327393	0.00000195
LOC_Os04g49450	4.943	6.246	5.734	8.887	10.362	9.856	0.856069894	6.87E-13
LOC_Os09g38540	1.67	1.82	1.927	0.818	0.724	0.853	-0.855998087	0.001181032
LOC_Os11g38220	19.841	18.528	23.665	36.757	35.368	34.284	0.855795856	1.42E-14

LOC_Os03g61260	13.201	14.497	13.356	6.697	6.909	6.928	-0.855295054	3.09E-10
LOC_Os07g10570	107.332	104.799	138.115	228.996	205.58	177.788	0.855088586	9.85E-08
LOC_Os01g55600	0.311	0.37	0.314	0.961	0.67	0.59	0.855059606	0.006204082
LOC_Os11g07100	6.508	6.783	6.761	11.485	11.892	10.766	0.854856069	1.08E-24
LOC_Os04g53560	10.449	10.805	11.077	6.489	5.364	4.064	-0.854568215	0.000000587
LOC_Os06g44970	4.785	5.694	5.181	2.412	2.633	2.754	-0.85454722	3.78E-09
LOC_Os05g18274	2.728	2.226	3.002	4.521	5.021	4.458	0.854234102	0.000000385
LOC_Os02g24270	1.928	2.206	1.768	3.743	2.896	3.706	0.853506177	0.000000517
LOC_Os01g03030	8.446	8.782	7.905	4.621	3.865	4.027	-0.853434184	6.89E-09
MSTRG.12840	0.714	0.991	0.589	1.758	1.187	1.61	0.853043462	0.001624063
LOC_Os01g01400	0.639	0.533	0.403	0.141	0.289	0.154	-0.853009947	0.006825122
LOC_Os07g44900	1.626	1.334	0.771	0.541	0.11	0.607	-0.853004191	0.009288543
LOC_Os10g28340	4.261	5.272	6.674	9.537	9.338	9.206	0.852322261	3.38E-11
LOC_Os04g58280	361.131	350.95	370.075	612.208	597.242	607.829	0.851287236	7.04E-141
LOC_Os11g32290	1.095	1.386	1.561	3.109	1.903	2.907	0.850840884	0.00163361
LOC_Os01g72810	97.091	97.582	99.957	168.645	158.525	168.091	0.850766549	4.68E-83
LOC_Os02g47840	18.238	21.113	24.264	35.574	39.846	33.236	0.850007371	2.02E-15
MSTRG.1737	28.338	31.717	35.124	53.411	58.883	50.288	0.849575353	1.32E-14
LOC_Os07g36544	10.096	10.062	9.493	17.502	16.396	16.159	0.849465173	6.76E-28
LOC_Os04g48270	5.489	6.26	5.806	9.253	10.882	10.013	0.849177495	6.34E-12
LOC_Os10g26560	1.73	1.508	1.163	0.645	0.722	0.666	-0.848984653	0.000421546
LOC_Os11g38210	15.047	14.61	17.935	25.161	30.113	26.598	0.848911637	7.96E-11
LOC_Os05g11320	20.146	21.909	24.125	10.114	11.357	11.68	-0.848682168	6.15E-09
LOC_Os01g17150	5.634	6.31	5.765	2.678	2.395	3.24	-0.848662921	0.000179081
LOC_Os06g23114	4.697	4.747	3.897	2.039	2.48	2.118	-0.848417216	5.46E-08
LOC_Os09g16030	3.647	2.441	2.922	1.49	1.51	1.304	-0.848405291	0.0000689
LOC_Os06g04990	386.873	374.803	381.9	646.739	634.233	636.046	0.848044085	1.17E-132
LOC_Os08g38710	6.38	7.127	7.086	11.421	12.121	11.353	0.84756456	5.49E-20
LOC_Os01g22370	14.114	16.349	13.824	22.181	25.636	27.271	0.847279425	7.05E-18
LOC_Os02g50630	7.663	6.652	7.594	3.731	2.819	4.311	-0.847154533	0.000000114
LOC_Os05g15530	32.4	32.32	36.8	57.382	57.001	56.619	0.846995367	4.95E-36
LOC_Os10g25900	1.357	0.607	1.124	0.296	0.556	0.276	-0.846335631	0.008275113
LOC_Os12g01370	66.805	68.234	62.877	33.422	33.742	34.681	-0.84584145	1.89E-50
LOC_Os03g11950	2.313	2.629	2.184	1.116	0.931	1.289	-0.844987031	0.000243073
LOC_Os03g42040	1.815	2.029	2.076	0.883	0.834	1.175	-0.844955893	0.00000505

LOC_Os05g38530	14.373	13.191	16.158	26.105	21.658	26.056	0.844933306	1.58E-21
LOC_Os12g17530	2.641	2.825	2.89	1.332	1.174	1.516	-0.844520725	0.0000377
LOC_Os09g36700	127.832	123.271	115.308	61.476	64.523	62.942	-0.844347587	1.57E-54
LOC_Os04g01740	2.463	2.149	2.589	4.294	3.689	4.463	0.843674591	3.38E-08
LOC_Os03g10850	4.131	4.592	5.201	8.802	7.23	8.823	0.843172741	0.000000035
LOC_Os12g02060	4.599	4.79	4.247	1.929	1.76	2.705	-0.841980484	0.000308806
LOC_Os04g41300	16.673	16.084	15.198	8.019	8.989	7.365	-0.841848155	3.68E-12
LOC_Os03g08520	8.441	9.066	9.453	3.606	5.356	4.359	-0.841660685	0.00000219
LOC_Os03g53900	16.534	14.929	18.011	29.768	25.792	27.963	0.841653982	3.49E-20
LOC_Os01g47070	10.013	8.146	7.623	15.554	13.609	14.707	0.841620864	1.64E-11
LOC_Os03g61670	13.992	13.636	11.38	5.793	7.304	6.763	-0.841113351	1.36E-13
LOC_Os02g45160	0.322	0.3	0.417	0.767	0.629	0.666	0.839392657	0.002436982
LOC_Os12g38150	230.661	227.676	248.284	378.288	354.339	472.66	0.839181635	2.03E-10
LOC_Os02g01590	36.977	39.379	33.225	18.407	19.194	19.033	-0.837968666	3.24E-36
LOC_Os03g15890	17.785	17.351	20.512	31.232	28.518	33.423	0.837526344	6.78E-22
LOC_Os01g04620	8.498	7.155	8.226	13.468	13.772	13.129	0.837372663	4.22E-14
LOC_Os03g05060	5.127	3.818	3.013	1.555	2.516	1.676	-0.836760016	0.0000474
LOC_Os05g34290	1.623	2.291	2.083	3.914	2.817	4.025	0.83672063	0.000078
LOC_Os07g11510	46.748	51.156	57.676	92.692	79.921	88.08	0.836492876	2.92E-25
LOC_Os08g03560	1.266	1.859	1.223	0.59	0.638	0.794	-0.836223877	0.000372456
LOC_Os08g30910	1.821	1.73	1.946	3.035	2.973	3.394	0.836144942	3.35E-09
LOC_Os03g59320	0.602	0.505	0.892	1.073	1.359	1.755	0.835702567	0.004709794
LOC_Os05g38480	4.7	5.573	5.534	2.899	2.135	2.896	-0.834584306	7.41E-08
LOC_Os07g27390	0.929	0.774	0.658	1.223	1.508	1.562	0.83436254	0.000233989
LOC_Os06g46740	14.969	12.31	12.124	19.159	20.395	27.182	0.834137923	3.19E-11
LOC_Os12g08810	86.272	89.33	84.349	44.135	45.711	45.382	-0.833224148	9.05E-71
LOC_Os07g07220	41.567	38.493	41.226	22.119	19.098	21.483	-0.833117472	1.25E-25
LOC_Os03g61290	2.358	3.188	2.647	1.233	1.573	1.232	-0.833047834	0.00000944
LOC_Os06g16200	0.649	0.675	0.684	0.25	0.19	0.293	-0.832839876	0.008794965
LOC_Os02g40680	6.323	5.847	5.652	2.897	2.731	3.136	-0.832508358	0.00000921
LOC_Os01g71140	4.24	3.16	3.302	6.262	5.723	6.429	0.832338052	0.000000231
LOC_Os11g20090	6.971	7.756	7.535	3.006	4.151	4.039	-0.832229021	9.25E-08
LOC_Os04g58190	29.824	27.868	29.158	47.833	47.825	50.387	0.831737683	9.36E-35
LOC_Os09g16510	24.649	26.216	23.234	13.049	11.585	13.594	-0.831730662	4.93E-21
LOC_Os01g31690	4.792	5.122	5.588	2.311	2.343	2.995	-0.831153962	0.00000685

LOC_Os12g34840	1.125	1.08	0.999	0.339	0.481	0.507	-0.831063706	0.004748866
LOC_Os03g30740	0.86	0.73	1.295	1.807	2.072	1.523	0.830989857	0.00069173
LOC_Os03g19720	17.671	23.603	18.15	11.113	7.655	10.793	-0.830386079	0.00000134
LOC_Os09g26670	0.764	0.874	1.402	0.284	0.402	0.318	-0.830319829	0.010735678
LOC_Os09g37690	3.207	3.031	2.771	1.28	2.018	0.997	-0.830272269	0.000259938
LOC_Os04g45730	6.74	6.502	6.899	3.957	3.208	2.991	-0.830240831	8.55E-08
LOC_Os03g10050	2.439	3.341	3.053	1.179	1.401	1.597	-0.830170787	0.000322872
LOC_Os11g42430	18.866	18.81	17.555	9.396	9.987	9.143	-0.829677031	1.75E-19
LOC_Os05g04240	0.692	0.397	0.969	1.266	1.924	1.019	0.829462441	0.003790476
LOC_Os11g05170	12.443	10.778	12.844	19.101	18.672	23.433	0.82929142	9.02E-10
LOC_Os07g11630	41.915	37.409	45.14	79.268	59.771	69.305	0.828566731	4.59E-17
LOC_Os03g55210	0.985	1.374	0.59	0.349	0.432	0.508	-0.828554825	0.002559432
LOC_Os06g07600	46.653	41.796	49.248	77.004	70.508	81.014	0.82847968	4.02E-41
LOC_Os12g07030	2.543	3.06	2.798	5.362	4.796	4.391	0.828249452	0.00000153
LOC_Os09g31000	39.616	40.216	42.886	68.056	66.806	69.202	0.827872319	6.99E-31
LOC_Os03g16670	117.101	126.737	115.763	62.743	64.342	60.569	-0.827868301	9.86E-56
LOC_Os07g20340	20.85	21.285	24.06	42.221	36.246	32.253	0.827803105	1.52E-18
LOC_Os10g35150	34.776	31.147	35.644	59.365	56.727	52.721	0.827441919	6.4E-34
LOC_Os07g08300	1.105	0.446	0.742	1.501	1.652	1.272	0.827225234	0.00106937
LOC_Os07g10820	7.083	6.396	5.814	3.141	2.849	3.761	-0.827006736	2.11E-08
LOC_Os12g40540	1.325	0.886	1.123	0.543	0.421	0.448	-0.826668775	0.004118123
LOC_Os09g30490	57.136	57.073	60.237	92.894	97.429	99.13	0.826638624	4.61E-37
LOC_Os06g11760	2.189	4.338	3.457	1.76	0.476	1.536	-0.825847331	0.007960389
LOC_Os12g21730	1.222	0.975	0.955	1.722	1.728	1.946	0.825713676	0.000000189
LOC_Os05g04740	13.178	13.35	12.446	5.856	6.082	8.025	-0.825530429	5.76E-12
LOC_Os06g14406	9.864	9.794	9.527	16.287	17.229	15.036	0.825392146	2.37E-24
LOC_Os04g33115	4.391	4.647	4.667	8.472	8.49	7.381	0.824762077	0.0000654
LOC_Os01g04370	15.879	14.943	17.957	28.136	25.593	28.052	0.824341007	4.87E-14
LOC_Os03g17220	0.155	0.288	0.454	0.774	0.558	0.758	0.824128939	0.0086362
LOC_Os06g05930	31.154	31.375	27.155	16.139	15.44	14.549	-0.824119176	2.57E-12
LOC_Os02g58470	2.795	2.77	2.6	1.251	1.505	1.133	-0.824034663	0.000327196
LOC_Os08g43920	1.175	1.423	1.197	0.126	0.639	0.53	-0.823822269	0.010383477
MSTRG.2946	1.689	1.323	0.973	2.085	2.343	2.87	0.823679599	0.000564291
LOC_Os02g32760	43.847	44.007	46.455	21.777	25.09	22.848	-0.823512799	4.22E-18
LOC_Os06g07220	174.331	180.878	161.228	84.718	92.271	93.157	-0.823411241	8.6E-52

LOC_Os03g14880	3.397	3.196	2.225	1.46	1.284	1.545	-0.8233827	0.0000614
LOC_Os03g22200	3.629	2.918	2.742	1.195	1.726	1.547	-0.823195272	0.000695161
LOC_Os11g31705	1.367	1.323	1.458	2.485	2.394	2.495	0.822473451	0.000087
LOC_Os10g32680	41.504	41.351	44.387	72.266	69.395	68.506	0.82149512	1.8E-44
LOC_Os04g43800	10.801	11.442	10.79	6.134	5.142	5.793	-0.821397252	4.86E-16
LOC_Os09g28180	11.026	11.167	11.561	19.774	18.156	17.876	0.82094903	2.39E-34
LOC_Os05g19570	7.805	9.144	7.592	3.816	4.641	3.9	-0.820876068	0.000000907
LOC_Os03g55640	14.55	16.89	14.925	7.564	8.309	8.392	-0.820788114	2.69E-18
LOC_Os05g41640	7.549	7.529	8.057	4.139	3.627	4.128	-0.820318032	8.44E-11
MSTRG.16265	10.751	11.148	11.577	18.935	19.07	19.167	0.820094775	0.00000021
LOC_Os11g40150	4.042	4.17	3.553	2.01	1.997	1.766	-0.820009507	0.0000475
LOC_Os07g46540	4.334	5.001	3.927	2.423	2.206	2.196	-0.819834548	2.53E-11
LOC_Os01g74410	1.438	0.877	1.15	1.886	2.056	2.428	0.819290837	0.000672905
LOC_Os03g46640	34.475	35.222	31.402	15.048	19.784	17.687	-0.819120699	2.18E-16
LOC_Os05g08860	4.115	3.663	3.262	1.75	1.228	2.076	-0.819062757	0.001249591
LOC_Os01g24090	2.059	1.209	2.075	0.844	0.684	0.727	-0.818465904	0.003612736
LOC_Os06g11240	68.344	68.83	68.811	33.285	37.603	37.165	-0.818415738	1.31E-42
MSTRG.23780	2.842	2.655	2.717	5.209	5.042	3.991	0.818264851	0.0000072
LOC_Os03g60100	6.653	7.305	6.595	3.643	2.872	3.676	-0.81819153	0.0000136
LOC_Os05g47950	0.633	0.408	0.552	0.977	0.991	1.199	0.817163419	0.003596052
LOC_Os03g57080	2.311	1.126	1.945	0.804	0.815	0.786	-0.817081599	0.002381047
LOC_Os03g30300	5.039	5.993	7.17	9.053	8.103	9.632	0.817011598	9.64E-15
LOC_Os11g40840	0.404	0.134	0.206	0.78	0.483	0.77	0.816756388	0.011396031
LOC_Os06g51260	2.683	2.111	3.114	4.098	4.337	4.896	0.816713647	0.000000712
LOC_Os12g01530	44.161	40.926	38.876	21.088	21.537	22.259	-0.816490832	9.46E-31
LOC_Os03g41060	5.362	5.388	5.609	10.346	9.01	8.787	0.815513153	0.00000304
LOC_Os01g42190	5.545	4.586	6.68	8.532	9.096	10.952	0.815444086	0.000000126
LOC_Os07g11770	2	2.505	2.759	4.276	4.368	3.923	0.815332129	0.00000539
LOC_Os03g05270	5.119	4.584	6.467	9.97	8.691	8.529	0.815208388	8.13E-09
LOC_Os03g53690	48.262	51.493	48.816	25.554	24.244	27.952	-0.815061828	2.25E-25
LOC_Os02g40730	0.759	1.127	0.749	0.535	0.342	0.184	-0.814462788	0.00668882
LOC_Os01g57510	2.316	2.137	2.977	4.469	4.174	3.994	0.813917637	0.00000013
LOC_Os01g63690	49.323	48.754	48.116	79.296	77.459	82.881	0.813605945	6.36E-60
LOC_Os03g51010	18.009	18.433	19.47	31.243	29.217	31.609	0.813535241	1.31E-28
LOC_Os04g04330	6.315	6.02	5.79	2.863	3.383	3.184	-0.813476653	2.01E-14

LOC_Os07g05840	1.345	1.924	1.98	2.85	2.983	3.3	0.813184805	0.0000168
LOC_Os10g31490	1.154	2.134	1.128	2.375	3.911	2.497	0.813049969	0.003713991
LOC_Os11g05640	16.404	15.262	20.237	28.788	26.995	30.101	0.812715919	1.15E-18
LOC_Os06g31800	41.656	35.581	34.375	60.383	58.054	65.402	0.812425436	3.28E-22
LOC_Os03g08170	2.967	2.046	3.227	4.574	4.608	4.906	0.812237406	0.00000108
LOC_Os05g51670	83.485	81.64	90.948	141.601	138.497	141.95	0.812195205	1.58E-62
LOC_Os09g37510	6.265	6.239	5.948	3.388	3.373	2.683	-0.811780972	7.25E-08
MSTRG.11570	1.502	1.941	1.676	3.717	3.321	2.533	0.811620767	0.001278538
LOC_Os03g25280	4.718	4.48	4.272	2.45	1.918	2.24	-0.811323494	0.0000516
LOC_Os03g19670	2.006	1.132	1.752	0.691	0.779	0.728	-0.811167423	0.002016985
LOC_Os07g10580	213.082	205.04	260.774	410.728	411.428	323.208	0.810493291	3.98E-08
LOC_Os04g46910	6.877	8.369	7.471	3.389	4.24	3.641	-0.81045858	0.0000377
LOC_Os09g39530	2.414	2.28	2.573	0.975	1.208	1.114	-0.810313395	0.002092165
LOC_Os03g46200	30.304	28.22	30.693	45.578	48.442	52.897	0.809894474	1.35E-21
LOC_Os07g31610	35.232	35.319	32.049	15.126	18.713	19.839	-0.809890852	5.54E-18
LOC_Os09g17680	9.345	9.809	9.246	15.977	14.43	16.731	0.809680259	1.47E-12
LOC_Os07g11900	0.957	1.962	2.013	4.06	3.367	2.869	0.809676697	0.004327419
LOC_Os03g36540	3.356	3.249	2.796	1.606	1.778	1.317	-0.808773279	0.0000123
LOC_Os12g41060	0.273	0.189	0.525	0.973	0.587	0.615	0.808597646	0.007717608
MSTRG.23325	0.9	0.861	0.726	1.542	1.244	1.54	0.808157753	0.0000482
LOC_Os08g01100	24.873	21.982	20.971	9.929	12.58	12.738	-0.808079244	7.1E-12
LOC_Os05g37970	19.814	22.062	24.43	37.352	36.926	35.112	0.807885612	1.65E-24
LOC_Os11g37950	25.738	21.583	24.98	40.455	39.593	39.009	0.80755506	1.31E-19
LOC_Os06g46350	3.08	3.095	3.187	2.207	1.075	1.151	-0.807152449	0.000727524
LOC_Os09g29430	2.824	3.215	3.471	5.102	5.553	5.393	0.806997952	7.35E-08
LOC_Os05g46510	32.581	31.808	30.698	52.82	49.879	52.527	0.806288789	4.82E-45
LOC_Os01g64640	74.692	76.137	74.434	38.029	38.198	42.56	-0.806171831	1.64E-28
LOC_Os09g36600	47.709	46.257	41.317	74.028	71.257	74.461	0.805843746	7.73E-52
LOC_Os02g12380	47.887	47.504	53.778	83.022	79.905	80.292	0.803584341	3.66E-49
LOC_Os07g02690	24.42	21.854	26.002	40.278	38.515	40.339	0.803473508	2.13E-15
LOC_Os04g54230	52.614	44.611	53.249	83.165	85.581	78.114	0.803461755	4.62E-22
LOC_Os02g08190	9.247	9.506	8.614	4.855	5.139	4.315	-0.803331482	1.96E-12
LOC_Os05g45820	0.858	0.502	0.787	0.188	0.191	0.352	-0.802750134	0.011134922
LOC_Os04g20070	1.095	0.795	0.937	1.971	2.426	1.514	0.802645389	0.00628159
LOC_Os01g15540	1.765	1.506	1.258	2.317	2.804	2.675	0.802534888	0.0000229

LOC_Os05g45580	1.391	1.296	0.918	0.698	0.463	0.452	-0.802489213	0.002884676
LOC_Os07g02940	320.206	320.082	350.164	568.369	543.275	500.603	0.802371208	2.85E-57
LOC_Os01g10900	0.474	0.956	1.033	0.37	0.455	0.239	-0.802087253	0.005354417
LOC_Os02g15120	0.548	0.814	1.054	1.585	1.928	1.432	0.801597086	0.00532519
LOC_Os05g03640	2.902	2.359	2.422	0.933	1.164	1.577	-0.799965403	0.000564958
LOC_Os10g42750	12.619	14.492	10.629	6.53	7.227	6.105	-0.799647084	1.49E-15
LOC_Os08g03240	1.699	1.311	1.36	2.493	2.62	2.417	0.799362364	0.000019
LOC_Os09g31031	98.188	91.124	90.389	51.459	50.91	46.211	-0.798876201	6.81E-31
LOC_Os01g33784	9.14	10.603	9.672	17.745	14.709	15.937	0.798747846	2.09E-13
LOC_Os02g55550	1.582	1.872	2.452	3.303	3.988	3.223	0.798264325	0.000331608
LOC_Os02g58670	8.426	9.379	8.779	13.7	16.039	14.261	0.797686731	2.21E-10
LOC_Os03g07350	1.217	1.068	1.118	2.348	2.04	1.788	0.796829593	0.000598212
LOC_Os08g41730	4.397	3.727	3.934	2.402	1.761	1.72	-0.796597803	0.000299883
LOC_Os05g45020	135.557	136.519	141.734	227.705	208.654	233.846	0.796053474	9.95E-58
LOC_Os01g69120	6.231	6.956	5.849	3.173	3.336	3.31	-0.795781556	1.43E-08
LOC_Os08g33100	30.489	27.968	27.033	12.04	18.428	14.202	-0.794912695	1.24E-09
LOC_Os02g51030	12.42	13.878	12.742	20.375	21.883	21.713	0.793957923	4.79E-14
LOC_Os01g58290	4.403	5.88	4.763	8.598	8.31	7.777	0.793480947	1.86E-12
LOC_Os03g43720	16.672	16.026	16.685	26.284	27.935	25.182	0.793036455	9.52E-31
LOC_Os12g02370	8.108	8.675	6.798	3.372	4.021	4.634	-0.792707172	0.00000169
LOC_Os10g41980	1.018	2.933	1.866	3.481	4.433	3.421	0.792506264	0.003773448
LOC_Os08g44940	4.988	3.272	4.573	7.523	7.763	6.78	0.792295546	0.0000362
LOC_Os02g29300	6.252	5.218	5.164	3.233	2.726	2.642	-0.791987514	0.000000215
LOC_Os08g34210	54.479	59.07	62.269	100.535	90.585	92.616	0.791769196	2.12E-43
LOC_Os06g48960	20.38	23.394	25.31	12.239	13.209	10.965	-0.791767272	1.19E-11
LOC_Os04g42470	23.476	25.973	26.901	41.97	41.781	40.584	0.79171861	1.82E-38
LOC_Os05g41940	6.926	7.491	6.932	11.902	10.745	12.367	0.791099684	1.64E-11
LOC_Os02g40784	125.599	124.515	147.288	224.295	211.871	210.234	0.79106055	1.16E-19
LOC_Os03g50530	0.536	0.893	0.532	1.341	1.303	1.814	0.790605095	0.007756536
LOC_Os07g08420	0.919	0.585	0.842	1.931	1.284	1.213	0.790548425	0.002473353
LOC_Os04g03860	2.043	1.92	1.633	2.813	3.466	3.055	0.79047191	0.000000227
LOC_Os08g31410	33.929	34.405	34.424	54.993	55.279	55.775	0.790154345	1.09E-58
LOC_Os02g03730	39.532	37.288	37.685	19.447	20.032	21.31	-0.789760512	5.11E-18
LOC_Os08g01370	182.365	195.797	198.844	299.222	349.389	286.611	0.789635928	8.23E-29
LOC_Os10g31320	78.526	81.933	82.444	119.541	157.951	116.181	0.789252631	3.34E-25

LOC_Os05g46460	50.075	54.404	62.155	84.515	91.066	94.807	0.789238297	8.5E-35
LOC_Os12g38250	0.841	0.667	0.977	1.894	1.535	1.568	0.789082069	0.005387961
LOC_Os01g57690	3.166	3.137	1.996	1.002	1.403	1.473	-0.789054159	0.001595215
LOC_Os01g16370	1.085	0.933	0.651	1.587	1.377	1.633	0.78781173	0.000134656
LOC_Os07g47140	2.504	4.466	3.575	1.622	1.959	1.645	-0.787111887	0.000205175
LOC_Os11g06700	1.515	1.527	0.942	0.619	0.502	0.718	-0.787000003	0.002064412
LOC_Os11g10090	7.39	8.707	9.889	14.535	13.979	13.923	0.786967815	3.86E-14
LOC_Os01g65920	28.798	28.256	28.904	45.128	44.552	49.146	0.786811454	2.5E-31
LOC_Os06g45060	54.728	56.274	58.457	91.897	82.295	99.651	0.78680896	1.82E-26
LOC_Os09g24530	1.373	1.122	0.913	0.43	0.369	0.65	-0.786498489	0.00571031
LOC_Os03g58170	9.171	8.32	9.89	16.671	14.149	16.657	0.786289951	7.65E-12
LOC_Os10g31660	7.609	9.159	7.433	12.897	12.307	14.943	0.786019037	0.000000166
LOC_Os02g41840	19.26	16.697	21.321	31.882	30.122	31.188	0.785931361	4.51E-16
LOC_Os04g35420	4.957	5.894	4.883	2.405	3.033	2.861	-0.785586438	5.78E-11
LOC_Os12g06980	2.108	2.081	1.919	0.973	1.206	1.031	-0.785557699	1.38E-09
LOC_Os05g48040	604.907	594.205	658.024	1003.193	991.594	990.304	0.785511978	4.74E-91
LOC_Os09g30414	1.53	1.336	1.745	2.89	2.562	2.403	0.785480431	0.0000214
LOC_Os01g65670	15.099	15.828	17.059	25.498	24.162	28.026	0.7853548	7.36E-21
LOC_Os01g15480	5.797	5.236	5.147	2.661	2.637	3.258	-0.784858083	1.09E-12
LOC_Os04g27060	9.057	9.095	9.987	14.366	16.795	14.792	0.784298268	8.08E-13
LOC_Os05g45950	103.643	101.025	94.689	51.853	57.167	51.914	-0.784140048	1.72E-44
LOC_Os12g04340	1.455	0.961	1.645	0.655	0.332	0.656	-0.783987269	0.007980536
LOC_Os01g02200	11.371	11.483	11.558	19.241	17.016	19.561	0.783981298	4.08E-16
LOC_Os07g48090	26.207	26.992	26.177	15.454	14.225	12.826	-0.783795572	1.77E-23
LOC_Os05g30190	3.968	4.332	3.874	1.985	1.94	2.253	-0.783067999	0.0000269
LOC_Os12g38750	3.002	2.661	2.982	4.505	5.371	4.454	0.782329033	0.000000161
LOC_Os09g07460	9.882	10.521	8.742	5.018	4.747	5.473	-0.781732091	3.46E-08
LOC_Os05g41750	7.957	6.986	7.503	3.807	3.985	3.996	-0.781373329	1.07E-08
LOC_Os05g43130	1.155	0.822	1.456	0.418	0.282	0.716	-0.780666638	0.007365556
LOC_Os12g38760	41.528	42.56	45.182	71.253	71.055	64.986	0.780600028	5.39E-46
LOC_Os12g02040	239.48	252.598	241.808	394.127	395.042	385.259	0.780520357	2.12E-93
LOC_Os10g39770	1.335	0.92	0.621	1.512	1.589	1.92	0.780312382	0.000379427
LOC_Os01g42860	29.551	32.969	37.117	50.527	60.024	51.679	0.780217328	4.12E-13
LOC_Os02g39730	4.198	3.342	5.414	8.227	7.12	7.535	0.779042888	0.0000258
LOC_Os03g07170	2.089	1.949	1.937	3.273	3.477	3.147	0.778947154	0.000000176

LOC_Os07g32560	7.293	6.88	5.655	3.443	3.452	3.394	-0.77885274	0.00000107
LOC_Os07g02920	53.215	48.886	53.607	84.732	80.579	84.883	0.778050982	1.29E-26
LOC_Os11g31540	40.049	41.59	38.112	62.461	66.792	62.922	0.777923496	3.48E-30
LOC_Os09g31350	1.114	0.797	1.05	0.418	0.242	0.586	-0.776844725	0.006743392
LOC_Os10g39300	1.932	1.994	1.355	2.996	3.318	2.863	0.776519884	0.000235965
LOC_Os01g45910	82.09	76.597	85.317	132.524	128.105	129.117	0.77618351	5.18E-63
LOC_Os02g56300	81.728	76.441	74.986	128.502	118.847	124.936	0.775986793	1.25E-56
LOC_Os04g32080	2.112	2.765	3.527	4.584	6.196	4.218	0.775259566	0.000780079
LOC_Os11g09130	3.505	3.892	2.881	5.597	5.171	6.159	0.774836772	0.000000324
LOC_Os04g32620	23.879	23.974	25.529	38.92	38.902	39.361	0.77466204	1.8E-54
LOC_Os01g41420	3.726	4.241	4.743	2.02	2.469	2.083	-0.77445623	0.00000873
LOC_Os04g47740	0.225	0.267	0.276	0.522	0.351	0.567	0.774376233	0.002415134
LOC_Os06g23420	0.507	0.291	0.343	0.077	0.183	0.072	-0.773998063	0.012321507
LOC_Os03g32490	4.367	4.938	4.961	8.975	7.333	7.32	0.773970537	0.0000008
LOC_Os08g33680	13.655	14.216	15.06	6.205	8.37	8.166	-0.773643262	2.92E-09
LOC_Os03g13160	41.055	43.529	39.311	21.248	25.27	20.129	-0.773337134	2.82E-17
LOC_Os06g04150	0.924	0.963	0.802	0.422	0.095	0.395	-0.773264313	0.012010319
LOC_Os07g09020	1.009	0.771	1.123	1.676	1.828	1.448	0.772827015	0.0000703
LOC_Os06g05020	368.868	362.339	352.537	579.602	555.785	589.56	0.772787841	2.7E-83
LOC_Os07g13770	0.216	0.321	0.231	0.521	0.529	0.813	0.77213947	0.011756611
LOC_Os05g35810	4.846	6.302	5.346	2.045	2.369	3.62	-0.771855964	0.000734489
LOC_Os12g06030	0.792	0.196	0.318	0.764	1.162	1.161	0.771299942	0.011215168
LOC_Os03g48490	2.208	2.315	1.801	1.053	1.117	1.123	-0.771025768	0.00000187
LOC_Os11g30560	5.25	6.222	5.24	3.045	2.404	3.237	-0.77098021	0.00000231
LOC_Os01g46410	1.136	1.86	1.902	1.048	0.58	0.445	-0.770932649	0.007126409
LOC_Os01g51890	13.102	12.443	13.626	21.967	20.332	20.36	0.770748833	1.98E-25
LOC_Os05g30940	2.184	2.656	2.139	1.149	1.311	0.806	-0.77073884	0.002550408
LOC_Os01g12440	23.633	21.427	22.865	12.076	11.835	12.697	-0.770621259	1.07E-19
LOC_Os03g25790	8.434	7.705	8.65	12.495	12.357	15.11	0.770268243	3.49E-13
LOC_Os01g71060	6.229	5.989	4.258	2.809	3.352	2.327	-0.76980102	0.0000205
LOC_Os05g44630	2.818	2.334	2.931	0.974	1.81	1.138	-0.76971185	0.001161774
LOC_Os01g13300	1.393	1.51	1.903	0.786	0.771	0.782	-0.769435102	0.000998601
LOC_Os12g04500	2.907	1.279	1.84	0.403	0.761	1.117	-0.76942814	0.010002866
LOC_Os05g03050	1.862	2.447	2.784	1.289	0.911	1.205	-0.769282309	0.001430475
LOC_Os04g44354	1.475	1.466	0.936	0.616	0.462	0.532	-0.769252303	0.00353517

LOC_Os03g43750	1.557	1.337	1.484	2.483	2.374	2.452	0.7688697	0.000011
LOC_Os01g52920	16.22	15.358	18.034	26.836	24.569	27.822	0.768728907	1.54E-26
LOC_Os07g48500	3.594	3.561	3.025	1.989	1.786	1.116	-0.768591075	0.001494612
LOC_Os08g30900	4.216	3.365	3.946	6.689	5.918	6.033	0.768562717	1.52E-12
LOC_Os05g06920	53.382	50.593	49.755	26.243	28.733	28.494	-0.768486621	5E-39
LOC_Os03g17164	6.391	6.356	5.794	3.014	3.545	3.291	-0.768439325	3.85E-09
MSTRG.23556	2.344	2.252	2.312	3.7	4.236	3.651	0.767886229	0.0000211
LOC_Os08g17820	0.257	0.196	0.29	0.124	0.077	0.096	-0.767698833	0.009093276
LOC_Os06g39240	17.028	12.326	12.808	22.931	19.505	26.076	0.767091043	0.000000012
LOC_Os07g37350	11.513	10.724	10.715	6.136	6.027	5.559	-0.767046294	8E-14
LOC_Os11g13890	11.705	14.001	12.044	7.187	6.649	6.294	-0.766688068	3.02E-09
LOC_Os07g30110	4.947	3.977	2.653	1.981	1.871	1.852	-0.76666614	0.000585255
LOC_Os01g23980	0.025	0.15	0.035	0.219	0.123	0.261	0.766041893	0.012136892
LOC_Os08g23280	2.02	2.076	2.23	0.938	1.189	1.13	-0.765527645	0.0000337
LOC_Os07g43800	20.059	20.611	16.915	8.899	10.655	11.112	-0.764955279	1.94E-09
LOC_Os04g38026	3.269	3.326	3.321	1.685	1.733	1.792	-0.764412876	0.00000131
LOC_Os03g16460	6.292	6.418	6.307	10.605	9.422	10.563	0.764269172	4.17E-12
LOC_Os07g31500	3.073	3.271	2.727	1.441	1.739	1.617	-0.764249187	0.000000117
LOC_Os04g17660	116.25	110.787	112.257	168.9	181.606	174.524	0.763975708	3.79E-62
LOC_Os05g47940	0.914	0.792	0.768	0.147	0.372	0.412	-0.763802559	0.01100687
LOC_Os06g35700	3.424	3.561	2.984	5.679	5.617	4.977	0.763751739	0.000000134
LOC_Os07g37240	2.656	2.899	2.765	1.172	1.012	1.827	-0.763746425	0.001292407
LOC_Os01g19460	0.55	0.545	0.489	0.945	0.925	0.93	0.763460561	0.001032503
LOC_Os06g48210	4.824	4.27	4.448	8.576	6.334	7.065	0.763076304	1.41E-08
LOC_Os05g33430	154.096	153.842	163.625	250.248	245.98	249.275	0.763010003	5.91E-84
LOC_Os02g50560	2.062	2.175	1.991	0.987	1.147	1.145	-0.76243587	0.0000014
LOC_Os12g05260	38.17	40.344	37.279	56.779	61.376	65.804	0.762140385	4.76E-25
LOC_Os10g31040	0.167	0.137	0.085	0.178	0.508	0.374	0.761777866	0.012580096
LOC_Os02g10500	40.57	41.801	41.981	65.868	60.87	71.233	0.761561023	1.52E-19
LOC_Os06g40180	33.853	35.613	34.447	18.351	17.821	20.406	-0.761517689	9.23E-33
LOC_Os04g48030	1.513	1.699	1.079	2.109	2.96	2.366	0.761507244	0.000502314
LOC_Os09g38510	0.722	1.208	1.532	0.305	0.751	0.285	-0.761320407	0.010582653
LOC_Os02g29190	0.419	0.29	0.729	1.009	0.9	1.17	0.760875758	0.009343671
LOC_Os07g45000	33.414	32.263	33.532	54.629	49.244	53.129	0.760658682	1.11E-39
LOC_Os01g41810	2.941	3.633	3.196	1.891	1.456	1.605	-0.760635509	0.000186799

LOC_Os02g47110	8.968	8.551	9.633	16.116	13.111	14.103	0.760405536	6.26E-11
LOC_Os04g56910	1.31	1.096	1.067	1.741	2.39	1.89	0.760289945	0.000508373
LOC_Os10g07400	5.934	5.905	5.997	9.273	10.807	8.492	0.760111123	3.01E-16
LOC_Os09g17190	0.554	0.481	0.699	0.368	0.102	0.062	-0.758904852	0.012400271
LOC_Os10g32720	16.294	17.23	21.371	38.065	25.65	28.601	0.758848041	0.0000754
LOC_Os06g45480	73.609	72.69	84.099	129.037	118.947	116.214	0.758773174	2.76E-37
LOC_Os07g44890	23.256	25.603	23.893	11.662	12.462	15.168	-0.758512363	1.16E-13
LOC_Os07g08950	11.941	12.296	13.122	19.48	21.355	18.463	0.758336881	3.1E-26
LOC_Os01g64310	2.065	1.72	2.177	3.77	3.314	2.976	0.758207966	0.00018022
LOC_Os07g17970	3.858	2.692	3.427	1.396	1.65	2.065	-0.757440901	0.000132794
LOC_Os11g43860	3.652	3.794	3.735	1.797	1.753	2.269	-0.757240137	0.0000158
LOC_Os01g14550	3.642	2.776	3.236	1.297	2.191	1.162	-0.756732575	0.001949102
LOC_Os03g16900	2.008	2.091	1.841	0.788	1.065	1.044	-0.756626812	0.001258692
LOC_Os05g26750	8.65	4.743	6.979	12.794	12.277	9.13	0.756576608	0.0000478
LOC_Os03g38570	0.242	0.193	0.192	0.375	0.352	0.438	0.756477533	0.003246939
LOC_Os03g36760	7.067	7.414	7.379	11.823	11.463	11.339	0.75641198	8.49E-24
LOC_Os10g17960	0.714	0.991	0.786	1.434	1.397	1.598	0.756188717	0.001497987
MSTRG.8363	3.198	2.897	2.806	1.366	1.429	1.607	-0.756135049	0.000776139
LOC_Os04g56470	50.516	48.019	51.504	79.52	76.859	80.171	0.755882974	4.1E-46
LOC_Os04g55970	41.464	43.101	44.87	69.665	65.368	68.976	0.755374566	5.96E-46
LOC_Os07g49110	7.746	7.519	6.368	4.097	3.404	4.145	-0.755354299	5.77E-11
LOC_Os01g60020	34.113	34.944	40.578	59.79	52.023	61.841	0.755099751	1.16E-21
LOC_Os08g39730	3.035	2.907	3	1.562	1.385	1.612	-0.754196901	0.000167466
LOC_Os12g36170	2.835	3.314	2.508	1.682	1.206	1.675	-0.754104069	0.00000176
LOC_Os03g51459	17.281	17.5	18.902	26.118	31.412	28.3	0.753261443	8.68E-12
LOC_Os12g13890	46.904	44.601	40.816	63.523	83.672	64.565	0.753043238	3.27E-15
LOC_Os01g64360	4.239	4.909	5.245	7.786	7.242	8.522	0.753038363	0.00000226
LOC_Os02g46473	6.565	4.403	7.223	10.426	10.173	10.021	0.752962703	0.00010067
LOC_Os01g48930	182.98	186.034	206.795	318.659	289.559	297.297	0.752768554	6.17E-50
MSTRG.24599	81.538	73.464	100.13	143.76	110.076	184.126	0.75263908	0.000480714
LOC_Os02g14800	5.956	5.631	5.903	9.28	9.371	8.539	0.751842109	1.56E-21
LOC_Os02g53450	3.113	2.246	2.512	1.322	1.315	1.415	-0.75121143	0.0000985
LOC_Os01g21170	4.193	3.881	4.521	7.779	6.984	5.901	0.750704264	0.00000452
LOC_Os01g13610	0.657	1.303	2.009	0.465	0.643	0.514	-0.750652623	0.009613342
LOC_Os02g53180	9.817	9.449	9.838	4.999	4.245	5.826	-0.750127763	2.46E-09

LOC_Os12g01260	37.836	33.594	38.795	18.562	17.125	23.848	-0.747695279	1.95E-10
LOC_Os05g50140	1.383	1.196	1.383	0.752	0.727	0.537	-0.747560598	0.0021327
LOC_Os03g53890	6.38	5.805	5.641	3.077	3.523	2.804	-0.747408139	0.00000483
LOC_Os09g30506	3.7	3.177	3.016	5.553	5.065	6.304	0.747070799	0.000535357
LOC_Os01g06890	0.536	0.581	0.935	0.344	0.224	0.276	-0.746990832	0.009563902
LOC_Os01g50100	3.435	2.879	3.018	1.726	1.553	1.717	-0.746969528	0.000000137
LOC_Os08g37370	71.265	69.332	73.949	37.751	39.511	41.434	-0.746876132	5.14E-38
LOC_Os02g56570	2.731	3.367	3.116	1.522	1.454	1.779	-0.746597237	0.000108543
LOC_Os03g13450	1.795	0.916	0.922	1.833	2.703	2.076	0.746359854	0.00208333
LOC_Os07g16040	42.88	40.728	35.037	20.795	24.283	19.963	-0.746340059	5.41E-18
LOC_Os02g33060	8.145	9.215	9.499	15.447	13.274	14.601	0.746337171	4.99E-09
LOC_Os11g47180	1.182	1.311	1.278	2.111	2.105	1.989	0.746255931	0.0000165
LOC_Os02g39490	15.738	16.88	15.715	7.516	9.599	9.041	-0.746212312	1.12E-09
LOC_Os09g15560	9.491	7.816	8.277	5.087	4.166	4.413	-0.745462191	0.000000511
LOC_Os04g40290	2.478	1.888	2.301	0.95	1.025	1.346	-0.745401111	0.000838475
MSTRG.22576	0.498	0.493	0.415	0.917	1.245	0.816	0.74522447	0.010331738
LOC_Os01g14630	0.758	0.543	0.541	0.284	0.124	0.152	-0.74502297	0.013040888
LOC_Os10g28320	3.52	3.777	3.658	1.654	1.632	2.283	-0.744999489	0.000308793
LOC_Os06g38960	8.474	8.133	9.736	4.69	5.091	4.445	-0.744897068	2.25E-08
LOC_Os01g54515	0.654	0.324	0.218	0.459	1.162	0.938	0.744716587	0.010219619
LOC_Os03g54910	10.275	11.298	8.985	6.073	5.227	4.928	-0.744547447	0.00000288
MSTRG.9264	1.657	1.786	2.01	2.897	3.128	2.802	0.744330948	0.00000105
LOC_Os03g10770	0.419	1.009	0.933	0.231	0.293	0.108	-0.744179001	0.012169535
LOC_Os11g07050	2.579	2.86	2.885	1.333	1.621	1.495	-0.743819692	0.000000904
LOC_Os10g03850	5.694	4.088	5.568	8.549	10.228	7.953	0.743688051	6.42E-08
LOC_Os12g39400	1.299	1.359	2.349	2.82	2.611	3.189	0.743605997	0.000637405
LOC_Os11g25560	6.667	6.606	4.555	2.951	3.221	3.325	-0.743582671	0.00000112
LOC_Os10g39170	3.42	3.872	2.277	1.335	0.916	2.35	-0.74347787	0.002117053
LOC_Os05g03460	0.394	0.174	0.281	1.057	0.557	0.553	0.743431095	0.012952451
LOC_Os08g05320	13.141	11.233	13.396	20.154	19.381	19.641	0.743036673	9.13E-26
LOC_Os03g49190	22.73	20.269	21.773	9.291	11.581	14.135	-0.742957822	5.52E-09
LOC_Os08g34720	75.51	76.604	76.145	42.444	41.16	42.779	-0.742772637	2.36E-56
LOC_Os06g02530	25.029	27.122	27.409	13.232	13.813	16.335	-0.742756168	2.74E-12
LOC_Os01g52110	175.427	170.83	195.847	275.973	272.159	291.322	0.742694027	9.79E-57
LOC_Os06g04940	202.644	202.63	200.306	306.672	325.742	311.502	0.742620527	1.8E-71

LOC_Os07g33360	3.133	2.808	3.19	1.889	1.397	1.517	-0.742297534	0.0000233
LOC_Os01g62260	23.057	25.53	25.612	37.881	37.576	41.057	0.742288648	1.68E-20
LOC_Os01g52660	2.435	2.037	1.637	1.044	0.635	1.074	-0.741775075	0.005687913
LOC_Os12g35610	87.943	86.085	85.417	129.466	136.104	136.286	0.741498352	6.34E-91
LOC_Os11g47590	547.635	549.081	572.144	870.593	811.136	919.128	0.74141387	7.76E-63
LOC_Os08g32160	7.179	5.87	5.353	3.254	3.129	3.315	-0.741325325	0.0000152
LOC_Os09g17850	9.242	8.967	7.772	4.058	5.297	4.685	-0.741104937	2.28E-08
LOC_Os08g33300	4.474	4.577	3.869	6.422	7.007	7.316	0.740895589	0.000000257
LOC_Os05g47670	8.524	10.119	10.359	15.516	16.709	13.816	0.740486051	4.99E-11
LOC_Os11g10060	13.666	14.269	13.611	22.135	21.332	21.459	0.740166907	2E-30
LOC_Os08g03700	2.197	1.944	1.835	0.908	0.882	1.097	-0.7398798	0.002289705
LOC_Os09g02770	13.377	13.144	11.299	20.931	16.896	21.891	0.739852758	2.56E-10
LOC_Os05g50750	14.929	14.321	13.619	22.589	22.557	22.144	0.739832907	3.25E-29
LOC_Os03g20120	16.907	16.752	19.76	28.102	28.046	27.815	0.739656578	5.38E-18
LOC_Os02g36500	19.46	20.463	20.576	11.051	11.367	10.792	-0.739503079	4.29E-14
LOC_Os01g68680	30.569	30.075	31.379	48.484	48.688	46.181	0.739133155	1.24E-53
LOC_Os10g12140	0.531	0.39	0.314	0.74	0.752	0.773	0.738960435	0.004118064
LOC_Os01g37350	0.326	0.183	0.273	0.388	0.638	0.45	0.738883139	0.005913322
LOC_Os12g29980	7.579	8.287	7.321	3.191	4.583	4.711	-0.738866987	0.000000244
LOC_Os01g71624	16.479	17.529	19.896	27.515	27.262	28.164	0.738516828	4.76E-15
LOC_Os07g48520	2.261	2.502	2.752	4.212	3.796	3.841	0.738477185	7.47E-10
LOC_Os02g03900	2.835	2.593	2.685	0.902	1.767	1.461	-0.738368518	0.000496443
LOC_Os01g64540	7.883	8.387	8.76	4.171	4.476	4.882	-0.737872823	6.28E-08
LOC_Os10g38740	67.3	74.427	69.72	37.639	37.317	42.174	-0.737573427	4.18E-32
LOC_Os01g12650	37.784	41.438	35.713	19.135	20.316	23.798	-0.737372218	7.18E-17
LOC_Os09g32988	2.835	3.036	2.283	1.072	1.461	1.555	-0.737122947	0.000881001
LOC_Os03g60560	17.167	17.26	18.113	27.156	27.772	27.722	0.736761323	4.61E-14
LOC_Os03g25920	9.341	8.368	9.421	4.769	4.909	5.02	-0.736201438	4.82E-08
LOC_Os09g36820	2.158	2.566	2.374	1.098	1.304	1.132	-0.73596716	0.001288696
LOC_Os05g37660	3.882	4.724	4.176	6.841	6.217	7.333	0.735912217	7.71E-08
LOC_Os01g47540	32.253	30.114	36.486	54.966	49.549	51.129	0.735777526	2.12E-25
LOC_Os11g09110	3.099	2.64	3.58	1.525	1.788	1.537	-0.735562353	0.000158867
LOC_Os06g30970	70.453	69.806	82.318	118.244	117.464	111.436	0.735556842	4.25E-29
LOC_Os01g65420	1.189	1.083	1.221	0.275	0.744	0.386	-0.735448646	0.0109538
LOC_Os10g30010	1.286	1.998	1.152	2.199	2.486	2.793	0.735436356	0.000444069

LOC_Os01g66860	8.756	8.964	7.788	13.544	12.841	13.845	0.73542613	6.98E-14
LOC_Os04g02050	23.512	23.387	22.364	11.783	13.952	12.34	-0.735136765	7.78E-15
LOC_Os03g16920	12.005	10.94	13.678	18.766	19.182	19.366	0.734334069	1.23E-18
LOC_Os12g13570	2.451	2.583	2.77	1.382	1.452	1.339	-0.734226692	0.0000053
LOC_Os03g03790	10.77	11.021	11.715	17.273	15.731	19.476	0.733933044	2.96E-15
LOC_Os11g10980	3.318	3.904	3.83	2.109	2.054	1.734	-0.73390719	0.00000578
LOC_Os01g53730	41.595	42.696	47.471	72.597	69.111	63.56	0.733537003	8.9E-28
LOC_Os08g40919	4.138	3.328	3.556	2.136	1.693	2.05	-0.733155461	0.0000026
LOC_Os06g06520	7.317	6.548	7.503	9.868	12.195	11.815	0.732961659	3.44E-09
MSTRG.5540	25.508	24.288	26.101	40.04	39.943	37.921	0.732710339	3.25E-37
LOC_Os08g32085	20.001	24.324	19.374	6.633	14.125	10.326	-0.732613947	0.00233451
LOC_Os01g03320	2.374	3.016	2.512	1.351	1.131	1.263	-0.732396312	0.003802321
LOC_Os02g43700	36.97	37.982	35.846	19.036	19.448	22.978	-0.732216581	2.02E-24
LOC_Os07g39860	70.196	63.612	66.989	110.046	99.393	102.879	0.732208051	5.27E-26
LOC_Os02g05470	4.411	4.543	5.021	2.533	1.695	3.172	-0.732200845	0.0000168
LOC_Os03g62490	21.467	20.995	21.571	11.272	13.671	10.395	-0.732098734	3.54E-13
LOC_Os10g32740	2.054	1.794	2.255	2.686	3.436	3.876	0.731660577	0.0000444
LOC_Os09g32330	34.625	34.775	38.482	55.742	53.475	58.504	0.731392563	2.75E-27
LOC_Os03g18220	287.31	290.33	289.066	459.428	431.962	448.508	0.730775678	8.6E-92
LOC_Os11g09170	27.343	28.161	28.744	15.474	15.867	15.548	-0.730767154	1.32E-30
LOC_Os11g41680	6.337	6.527	6.961	3.42	3.834	3.508	-0.73067394	0.000000182
LOC_Os01g42234	27.783	28.368	26.834	15.417	15.856	14.884	-0.730154265	1.36E-23
LOC_Os01g73460	2.099	2.079	1.735	0.962	0.908	1.055	-0.729403368	0.001684932
LOC_Os01g42040	2.308	3.032	2.51	1.269	1.339	1.477	-0.729220883	0.000188134
LOC_Os09g15480	3.52	2.616	4.707	5.613	6.359	5.909	0.728960701	0.0000847
LOC_Os09g04508	23.317	22.235	25.398	35.575	35.087	40.057	0.728929363	5.51E-16
LOC_Os01g22640	2.486	1.922	1.668	0.848	1.067	1.121	-0.72840257	0.001302726
LOC_Os12g12950	5.104	3.973	4.918	2.663	2.393	2.208	-0.72659947	0.000301497
LOC_Os03g17560	2.384	3.271	4.092	1.121	2.63	0.717	-0.726517351	0.006394488
LOC_Os01g65190	4.333	4.078	3.625	2.064	1.827	2.515	-0.726504523	0.0000163
LOC_Os07g23150	4.51	4.812	4.103	2.476	1.933	2.691	-0.726477509	0.0000406
LOC_Os05g35010	3.471	2.961	3.377	1.747	1.683	1.742	-0.72626239	0.0000808
LOC_Os01g08170	2.316	2.488	1.559	0.969	1.22	1.154	-0.72599622	0.0000997
LOC_Os05g48160	2.017	0.789	1.573	2.917	2.93	2.441	0.725462179	0.005003843
LOC_Os11g06070	0.181	0.179	0.299	0.699	0.602	0.392	0.72539285	0.013631636

LOC_Os05g27780	206.533	210.132	230.105	334.604	330.842	332.153	0.725363611	1.38E-60
LOC_Os11g39220	67.994	66.882	73.605	105.958	108.772	106.762	0.725183404	2.69E-58
LOC_Os08g04460	70.5	78.082	75.548	115.959	120.531	109.705	0.724432776	3.59E-37
LOC_Os01g67810	3.292	4.177	4.156	2.312	2.061	1.823	-0.724407106	0.0000339
LOC_Os03g22790	47.323	47.546	49.319	72.004	73.378	76.924	0.724150679	2.41E-47
LOC_Os02g05640	1.472	2.187	2.699	3.865	4.238	3.319	0.723990566	0.003301065
LOC_Os03g53340	0.239	0.261	0.357	0.424	0.427	0.85	0.723424931	0.010574451
LOC_Os01g60850	2.444	3.086	2.986	4.574	4.857	4.024	0.722909495	2.17E-08
LOC_Os03g43010	8.968	8.769	8.358	5.009	5.192	4.151	-0.722601519	6.08E-09
LOC_Os05g50160	5.031	5.879	4.613	2.885	2.413	2.868	-0.722408528	0.000111921
LOC_Os10g28630	3.953	3.242	3.442	1.646	1.587	2.289	-0.722010049	0.000465116
LOC_Os05g39450	3.938	4.78	3.701	2.327	2.359	1.731	-0.721871517	0.000496874
LOC_Os04g47310	26.195	29.554	30.387	47.406	44.872	41.445	0.721415666	1.05E-16
LOC_Os02g32980	17.872	16.014	15.531	8.596	8.358	10.185	-0.721330497	3.9E-09
LOC_Os10g28080	19.701	18.805	19.716	29.444	30.064	30.657	0.7213109	7E-19
LOC_Os01g52500	74.449	75.322	68.592	39.303	43.091	40.627	-0.720837126	2.74E-43
LOC_Os03g34040	14.006	11.34	13.554	7.175	7.838	6.422	-0.72078139	9.22E-09
LOC_Os05g11950	13.37	13.604	11.069	6.522	7.577	6.923	-0.720722734	1.24E-09
LOC_Os10g39890	46.69	51.533	39.716	24.582	29.973	21.832	-0.720656042	6.25E-11
LOC_Os04g01610	4.424	4.643	4.323	2.527	2.134	2.546	-0.720381435	0.00000728
LOC_Os07g45530	0.28	0.24	0.355	0.126	0.137	0.122	-0.720357125	0.008765911
LOC_Os08g32620	5.643	7.479	6.479	3.2	3.739	3.528	-0.719661133	0.0000304
LOC_Os03g01470	6.22	4.537	6.122	2.43	3.097	3.476	-0.719434678	0.0000405
LOC_Os02g08490	5.639	4.941	6.043	8.925	8.116	8.821	0.719365781	5.95E-13
LOC_Os03g31160	5.029	5.204	5.114	3.094	2.943	1.989	-0.718954342	0.000890985
LOC_Os01g65320	4.696	4.602	5.103	7.394	7.682	7.247	0.718732088	2.35E-16
LOC_Os10g01350	1.413	1.231	1.481	2.021	2.429	2.35	0.718703883	0.000160191
LOC_Os09g17146	3.411	5.492	4.15	2.183	2.213	2.514	-0.718074908	0.000094
LOC_Os04g57810	62.238	62.348	66.973	104.568	93.811	96.795	0.717688073	9.46E-38
LOC_Os02g08260	9.971	9.435	9.828	5.627	5.021	5.306	-0.717190509	0.000000611
LOC_Os07g32680	19.597	18.361	17.329	26.025	32.673	26.956	0.716967739	5.86E-15
LOC_Os02g19990	7.65	6.611	6.185	3.168	4.127	3.808	-0.716860033	0.00000256
LOC_Os11g16390	17.35	16.332	16.885	8.472	9.812	9.89	-0.716802079	4.34E-13
LOC_Os01g56810	6.195	5.539	5.68	8.075	8.864	10.316	0.716573567	5.47E-09
LOC_Os01g28300	4.585	3.226	4.082	5.482	6.661	6.773	0.716472673	0.00000237

LOC_Os02g10490	2.037	1.961	1.992	0.88	0.911	1.347	-0.716063515	0.000226398
LOC_Os02g03030	3.709	3.569	2.859	1.297	1.262	2.424	-0.71572921	0.002502915
LOC_Os04g05010	22.984	21.043	24.096	35.786	33.712	35.642	0.715534255	3.61E-18
LOC_Os12g35480	0.752	0.602	0.769	1.283	1.131	1.356	0.715421262	0.003492086
LOC_Os03g44890	13.924	14.759	14.232	7.392	8.475	8.218	-0.714923793	2.87E-20
LOC_Os07g47100	8.698	8.544	7.096	13.399	12.181	12.248	0.714716435	1.06E-12
LOC_Os06g01920	7.579	8.592	6.887	3.36	4.741	4.126	-0.71447694	0.000119813
LOC_Os03g01900	66.316	69.397	67.782	36.246	34.601	43.014	-0.714375371	5.05E-17
LOC_Os04g48850	3.046	2.277	3.013	1.057	1.672	1.639	-0.714286625	0.000314506
LOC_Os04g25784	21.175	20.453	21.279	35.191	32.809	30.95	0.713892233	6.28E-21
LOC_Os10g36500	15.18	14.715	13.449	6.984	7.918	8.68	-0.713625575	0.000000111
LOC_Os05g35290	53.458	55.429	55.593	30.312	30.496	32.066	-0.713261209	9.26E-43
LOC_Os07g41720	2.324	2.785	3.213	1.87	1.24	1.122	-0.713249548	0.001489874
LOC_Os04g47190	17.879	16.286	16.875	26.748	24.807	27.107	0.712500307	2.65E-20
LOC_Os05g50654	6.255	9.118	9.031	3.815	4.499	4.604	-0.712242105	0.000174259
LOC_Os03g60340	5.588	4.076	4.275	2.48	2.179	2.783	-0.712052273	0.0000582
LOC_Os12g36980	4.14	3.364	5.619	6.95	6.235	8.664	0.71197902	0.00055427
LOC_Os12g44190	5.492	5.72	5.009	7.689	8.372	9.195	0.71194055	2.7E-09
LOC_Os01g70710	9.352	10.338	10.009	15.013	14.927	15.715	0.711805567	3.15E-13
LOC_Os01g69930	0.45	0.223	0.488	0.908	0.66	0.627	0.711700675	0.007158501
LOC_Os03g52400	11.541	11.101	12.748	5.339	5.214	8.461	-0.711625305	0.0000262
LOC_Os09g37070	4.895	6.606	6.498	3.303	3.495	3.034	-0.711586112	0.00000273
LOC_Os04g46460	7.691	9.302	8.814	4.556	5.002	5.175	-0.711547417	0.000000152
LOC_Os12g29400	5.012	6.707	5.311	9.042	7.128	11.27	0.711337168	0.000067
LOC_Os01g61980	0.841	0.75	0.655	1.581	1.233	1.365	0.710968286	0.006623733
LOC_Os01g02520	2.167	2.137	2.041	3.75	2.9	3.459	0.710880655	0.00000944
LOC_Os02g06580	1.537	1.468	1.698	2.966	2.626	1.988	0.710649157	0.0000408
LOC_Os06g08500	3.807	3.196	3.976	1.479	2.275	1.86	-0.71042743	0.001230873
LOC_Os06g07560	9.594	9.686	8.174	4.453	4.824	5.714	-0.709909838	0.0000015
LOC_Os04g02530	1.457	1.848	1.937	2.75	2.816	2.654	0.709906348	0.0000318
MSTRG.14583	4.471	2.421	4.909	7.423	5.897	6.048	0.70941494	0.000261261
LOC_Os08g28030	7.684	8.441	7.833	4.05	4.897	4.384	-0.708575605	1.67E-09
LOC_Os06g29460	1.841	2.144	1.725	3.034	3.2	2.954	0.708404809	0.0000638
LOC_Os02g04460	11.43	11.557	11.125	6.503	6.362	5.868	-0.708372205	0.00000077
LOC_Os04g28990	40.853	39.69	36.814	20.382	23.127	22.596	-0.708034208	9.32E-19

LOC_Os06g09170	5.838	6.654	6.723	10.877	9.578	9.72	0.707461409	5.95E-09
LOC_Os08g08980	7.602	8.533	5.663	3.094	3.949	4.499	-0.707390783	0.000177935
LOC_Os01g14410	4.144	3.683	4.638	1.881	2.324	2.198	-0.706245043	0.001177736
LOC_Os03g03550	2.689	2.739	2.561	1.539	1.248	1.274	-0.706212419	0.001862556
LOC_Os02g39380	1.105	1.232	0.95	0.267	0.135	0.623	-0.70612173	0.013931318
LOC_Os01g64730	30.361	29.447	29.079	47.097	44.294	45.007	0.705955965	1.18E-28
LOC_Os12g42700	12.729	12.745	11.588	7.029	6.861	6.937	-0.705856283	2.57E-14
LOC_Os02g43280	9.416	9.305	8.977	5.862	4.924	4.709	-0.705721471	3.15E-09
LOC_Os05g48660	8.144	9.226	8.558	13.317	13.288	13.205	0.705688813	1.01E-13
LOC_Os10g33040	24.532	23.498	24.676	38.513	35.471	36.669	0.705650112	2.26E-45
LOC_Os01g72630	0.658	0.728	0.39	1.12	1.248	1.047	0.705351428	0.008776944
LOC_Os01g16030	78.342	79.878	79.533	46.051	45.951	43.019	-0.705340472	6.32E-27
LOC_Os03g03020	8.135	6.799	7.205	3.257	4.437	4.242	-0.705146544	0.0000461
LOC_Os06g22070	27.904	26.254	25.585	13.905	13.857	16.939	-0.704966559	1.05E-12
LOC_Os01g07990	1.267	1.188	1.137	1.879	2.212	1.632	0.704879692	0.0000194
LOC_Os04g32740	22.042	20.716	22.162	34.177	31.072	33.797	0.704445243	6.6E-25
LOC_Os12g32330	8.13	9.148	10.444	5.583	4.851	4.806	-0.704399186	0.00000201
LOC_Os01g24060	4.194	4.256	3.77	2.476	2.056	2.147	-0.704334882	0.0000108
LOC_Os02g32250	16.627	16.43	15.202	22.721	24.767	24.78	0.704185512	9.47E-31
LOC_Os10g42770	5.784	4.808	4.899	2.459	2.636	3.139	-0.704084947	0.000175906
LOC_Os06g08340	3.153	3.878	2.193	1.678	0.85	1.373	-0.703955829	0.010942045
LOC_Os03g63770	174.5	167.557	171.128	254.224	261.46	251.312	0.703603939	6.97E-100
LOC_Os01g52720	10.43	9.479	8.942	5.454	5.086	5.234	-0.703505382	0.00000248
LOC_Os10g26280	3.626	3.209	2.906	1.958	1.494	1.81	-0.703437275	0.0000335
LOC_Os08g31510	5.508	4.677	5.374	2.884	2.424	3.087	-0.703207242	0.0000448
LOC_Os08g23870	78.374	79.992	71.922	41.254	47.923	41.259	-0.703022036	6.28E-21
LOC_Os01g72800	4.905	4.903	4.059	2.136	2.632	2.623	-0.702868964	0.000163227
LOC_Os05g45460	1.623	1.876	1.786	0.574	0.529	1.22	-0.702750454	0.009495212
LOC_Os06g10650	78.242	72.282	73.942	111.577	108.155	121.486	0.70274825	1.48E-34
LOC_Os03g07340	1.271	0.755	1.165	2.248	1.553	2.235	0.702652357	0.00823631
LOC_Os06g39330	5.248	4.996	5.189	3.144	2.918	2.351	-0.702580704	0.0000167
LOC_Os03g45370	6.986	7.212	7.055	3.676	4.061	4.12	-0.701639302	5.96E-09
LOC_Os08g30930	11.773	12.94	12.197	19.156	19.82	17.405	0.70100264	2.75E-18
LOC_Os05g02220	35.816	38.637	37.218	57.989	55.104	56.776	0.700916922	1.86E-39
LOC_Os05g11510	2.667	3.682	3.095	1.752	1.581	1.714	-0.700874938	0.000158867

LOC_Os07g02150	1.549	2.479	2.403	0.747	0.757	1.397	-0.700650554	0.00852504
LOC_Os08g09570	1.533	1.277	1.548	2.765	2.172	2.104	0.700574686	0.000150133
LOC_Os03g04210	3.905	4.313	4.754	1.729	2.128	2.771	-0.700195954	0.00155659
LOC_Os09g37394	3.015	2.988	2.611	4.848	4.914	4.759	0.70017692	0.001547086
LOC_Os04g36580	0.749	0.98	1.114	1.636	1.437	1.517	0.699899402	0.000131759
LOC_Os11g47600	215.373	219.97	226.945	336.32	332.987	333.109	0.699781549	1.86E-78
LOC_Os02g31924	4.177	5.39	5.878	7.966	8.074	9.199	0.699317007	0.000368997
LOC_Os10g36550	100.923	95.938	103.186	149.384	152.823	151.659	0.699235427	9.26E-66
LOC_Os02g57670	6.015	5.8	4.53	2.643	2.888	3.149	-0.699160082	0.000256598
LOC_Os12g34802	3.48	4.925	5.216	7.939	7.561	6.326	0.699086601	0.0000616
LOC_Os03g40000	14.679	13.879	14.766	22.82	20.505	23.679	0.698371739	1.49E-08
LOC_Os11g31660	1.773	1.565	1.307	3.272	2.449	2.085	0.698225129	0.00148975
LOC_Os09g07154	2.314	2.814	2.459	3.603	4.474	4.364	0.697984816	0.000517033
LOC_Os03g18790	5.972	6.069	5.858	9.447	8.738	9.228	0.697527898	3.15E-12
LOC_Os12g16010	41.787	40.755	37.068	19.017	23.995	24.626	-0.697368303	2.03E-14
LOC_Os10g29650	22.112	21.851	22.605	34.598	32.25	32.066	0.697151424	1.96E-42
LOC_Os08g39330	40.772	40.272	47.972	60.828	61.407	74.626	0.697007219	1.35E-15
LOC_Os01g41770	9.715	9.698	9.687	15.76	13.533	14.979	0.696684905	3.96E-18
LOC_Os09g24850	6.93	6.498	6.629	10.366	11.233	9.021	0.696500491	2.29E-15
LOC_Os06g49160	3.121	2.188	2.377	1.204	1.502	1.126	-0.69638662	0.003252269
LOC_Os07g31250	1.758	1.808	1.694	0.773	0.871	1.104	-0.695738564	0.000778196
LOC_Os09g31430	104.338	97.4	91.434	53.959	57.596	55.895	-0.695723057	3.38E-38
LOC_Os03g42760	3.133	3.017	2.575	4.991	4.567	4.159	0.695596216	0.00000879
LOC_Os10g40460	33.962	35.695	38.944	19.411	20.524	21.17	-0.695472262	3.42E-11
LOC_Os01g64450	17.421	15.294	15.871	23.295	24.635	26.352	0.69541307	2.95E-12
LOC_Os01g64250	12.117	12.26	11.615	18.313	16.336	20.333	0.695060197	4.01E-12
LOC_Os06g11840	3.6	3.355	4.019	5.845	5.332	6.043	0.694937478	0.00000511
LOC_Os05g13940	27.931	26.456	22.82	12.776	15.534	15.436	-0.694569826	2.05E-14
LOC_Os03g48760	12.099	10.311	8.921	4.657	5.626	6.486	-0.694384314	0.000126055
LOC_Os11g16550	4.157	4.082	4.424	2.256	2.141	2.444	-0.694380354	0.000097
LOC_Os01g17320	11.874	12.113	12.232	6.283	6.85	7.342	-0.694282273	2.59E-12
LOC_Os02g13850	22.408	21.209	22.951	34.674	33.403	32.559	0.694201278	1.01E-36
LOC_Os02g19850	2.364	2.149	2.446	1.088	1.463	1.193	-0.694039834	0.000177302
LOC_Os08g32360	1.048	0.89	0.915	1.444	1.659	2.115	0.693903706	0.006911873
LOC_Os01g45670	13.677	12.824	12.709	19.261	19.209	21.401	0.693849723	3.75E-12

LOC_Os05g07600	0.906	1.102	0.944	0.238	0.564	0.409	-0.693729744	0.012745274
LOC_Os09g39780	72.326	63.185	67.562	37.09	36.779	41.582	-0.693729375	9.81E-18
LOC_Os01g18170	212.571	222.817	196.994	112.039	118.596	130.949	-0.693627322	4.87E-35
LOC_Os02g20360	0.954	1.575	1.166	1.908	2.417	1.943	0.693521332	0.002122208
LOC_Os08g07690	19.05	18.061	16.487	10.536	8.752	10.442	-0.693004173	0.000000799
LOC_Os02g06640	2321.645	2275.929	2506.884	3669.149	3417.162	3586.415	0.692790776	6.96E-62
LOC_Os01g47330	7.107	4.475	6.029	3.46	2.793	2.995	-0.692227544	0.000776966
LOC_Os02g33450	26.437	28.939	24.553	13.627	15.083	16.657	-0.692115292	7.4E-15
LOC_Os07g36390	1.502	1.447	1.843	0.674	0.952	0.878	-0.691483106	0.001083434
LOC_Os10g26390	2.767	2.034	2.619	1.033	0.698	1.449	-0.690413481	0.011461259
LOC_Os06g11060	7.581	7.856	7.27	3.951	3.238	4.794	-0.690071998	0.00058591
LOC_Os07g47660	1.079	1.159	1.072	2.083	1.847	2.677	0.689954696	0.011504469
LOC_Os07g47350	35.89	34.939	37.34	55.858	52.256	54.803	0.689880074	2.18E-40
LOC_Os04g49790	1.172	1.04	0.962	0.489	0.479	0.647	-0.689836339	0.002214142
LOC_Os08g06370	9.603	10.452	9.138	5.79	5.229	5.347	-0.689528639	5.23E-08
LOC_Os06g02650	0.27	0.421	0.354	0.819	0.717	0.731	0.689231832	0.013728112
LOC_Os07g48390	19.664	21.068	17.555	11.391	11.132	10.437	-0.689134093	1.71E-10
LOC_Os03g10030	13.144	10.742	15.042	6.479	7.74	7.528	-0.689010131	0.000000569
LOC_Os06g05700	57.087	55.965	61.948	88.559	84.83	90.297	0.688875794	2.23E-33
LOC_Os03g56670	2.981	2.29	1.835	0.547	1.76	1.022	-0.68849692	0.007744106
LOC_Os03g28130	3.756	4.398	3.2	1.94	2.151	1.984	-0.688195701	0.000320344
LOC_Os03g49750	2.656	2.613	2.955	1.621	1.337	1.55	-0.688064736	0.0000328
LOC_Os01g05010	35.601	40.061	36.574	21.501	21.601	20.99	-0.6879561	3.49E-17
LOC_Os07g17330	74.08	78.357	82.316	41.934	44.806	47.344	-0.687768735	2.51E-17
LOC_Os03g48080	181.285	192.349	179.742	101.475	110.309	106.39	-0.687510498	1.53E-40
LOC_Os02g06725	7.922	7.508	6.73	11.485	11.304	10.834	0.687440662	1.8E-13
LOC_Os06g33640	1.519	1.324	1.448	2.86	3.149	2.137	0.687391759	0.004853146
MSTRG.1196	16.01	14.61	15.32	23.098	23.068	23.497	0.687242407	3.42E-15
LOC_Os02g10800	1.805	1.945	1.314	2.867	3.237	2.225	0.687221857	0.000916994
LOC_Os09g01590	6.521	7.027	6.888	10.484	10.105	10.73	0.687007747	8.24E-09
LOC_Os09g31478	70.936	71.143	73.549	109.003	107.45	106.843	0.686664289	8.76E-57
LOC_Os09g15820	16.23	15.466	14.314	22.099	23.525	24.173	0.686441882	2.28E-13
LOC_Os01g49290	269.548	271.76	255.706	147.279	153.03	158.882	-0.686182569	2.4E-60
LOC_Os01g71094	48.134	46.154	49.859	71.131	70.062	75.593	0.686149543	6.69E-32
LOC_Os06g50870	9.084	8.599	10.239	13.955	14.823	14.194	0.686014115	5.51E-08

LOC_Os12g39130	5.819	4.442	5.598	2.301	2.892	2.839	-0.685852866	0.00294533
LOC_Os06g05690	21.415	22.495	20.246	13.03	12.078	11.38	-0.685743551	5.57E-12
LOC_Os03g24160	3.394	3.12	2.85	1.46	1.96	1.734	-0.685654913	0.0000246
LOC_Os06g14620	45.51	45.582	43.185	25.176	27.938	23.983	-0.685644277	1.39E-23
LOC_Os09g31970	186.153	183.522	187.524	289.762	267.249	278.002	0.685434035	2.39E-67
LOC_Os10g02220	1.561	1.384	0.781	0.534	0.434	0.793	-0.685337999	0.007284142
LOC_Os04g53360	2.914	2.251	2.355	1.263	1.328	1.448	-0.685162329	0.000290814
LOC_Os11g35090	8.745	7.585	7.818	4.043	4.832	4.763	-0.684912496	6.4E-09
LOC_Os03g60480	25.212	24.275	28.953	42.14	37.963	38.414	0.684767642	1.38E-17
LOC_Os11g41380	1.75	1.44	1.605	2.461	2.93	2.354	0.684603717	0.000395033
LOC_Os03g46600	13.264	12.246	13.359	6.64	6.878	8.292	-0.684166446	0.00000017
LOC_Os03g13950	30.832	32.442	31.063	48.657	45.857	48.221	0.68392046	1.14E-19
LOC_Os06g45590	3.501	4.955	4.005	2.091	1.858	2.767	-0.683518437	0.000221509
LOC_Os12g25120	36.169	36.153	33.797	19.117	20.835	20.754	-0.683369623	8.29E-16
LOC_Os10g42910	0.359	0.641	0.612	0.678	0.971	1.05	0.683195056	0.001710474
LOC_Os08g25140	96.084	93.98	99.773	150.206	141.669	142.442	0.683165444	9.33E-45
LOC_Os03g11060	17.556	16.869	16.744	8.98	8.641	11.158	-0.683036569	6.13E-08
LOC_Os03g12670	396.71	385.453	430.256	637.768	623.481	598.803	0.682929952	8.93E-64
LOC_Os02g33070	58.597	62.349	68.349	99.207	93.781	91.835	0.682739129	2.63E-23
LOC_Os07g15540	1.349	1.21	1.551	2.335	2.136	2.086	0.682523539	0.000173772
LOC_Os03g33090	3.308	3.277	2.881	5.265	4.42	5.469	0.68239297	0.000314306
LOC_Os07g40224	5.183	4.87	5.242	7.097	7.651	8.746	0.682011291	0.000000211
LOC_Os07g45570	0.155	0.102	0.142	0.431	0.319	0.186	0.681820983	0.014733184
LOC_Os04g50950	2.818	3.458	2.347	1.422	1.441	1.772	-0.681620259	0.00030315
MSTRG.25883	7.188	6.704	6.836	10.758	10.337	10.253	0.68135294	5.38E-13
LOC_Os03g61160	52.377	50.36	49.551	71.73	76.736	79.473	0.681207882	4.14E-31
MSTRG.11593	60.302	61.875	61.541	98.529	96.022	79.543	0.68077177	1.37E-21
LOC_Os07g26460	3.493	2.966	3.137	1.307	1.882	1.543	-0.680557883	0.005096922
LOC_Os07g46310	1.074	0.532	0.938	1.431	1.675	1.245	0.680246275	0.003325557
LOC_Os05g43610	4.108	3.386	3.466	1.561	2.435	2.098	-0.67934675	0.0000108
LOC_Os08g34360	3.517	3.145	3.481	5.704	4.656	5.361	0.679319697	0.00000619
LOC_Os02g58300	14.676	14.293	10.869	7.682	8.205	6.634	-0.679210142	5.21E-08
LOC_Os02g04110	3.318	3.071	3.109	1.618	1.925	1.546	-0.679130042	0.000543653
LOC_Os01g32380	11.557	9.674	13.336	16.82	17.243	19.588	0.67906951	0.00000634
LOC_Os04g42430	41.454	39.705	41.317	63.339	61.253	58.609	0.678890617	1.39E-34

LOC_Os03g09220	43.77	41.819	45.398	65.234	66.663	63.864	0.678666585	4.28E-41
LOC_Os11g36150	19.606	19.25	18.183	26.277	30.5	28.583	0.678171807	1.57E-28
LOC_Os12g02250	6.499	6.434	6.375	9.699	10.399	9.047	0.678026533	9.18E-14
LOC_Os03g57120	26.366	26.772	25.583	13.774	16.432	15.056	-0.678000738	3.28E-17
LOC_Os11g04910	6.927	7.283	5.832	10.296	9.123	11.793	0.677965561	0.0000276
LOC_Os01g55260	17.89	18.199	16.793	9.692	9.928	10.762	-0.677808645	6.67E-16
LOC_Os05g49380	3.372	2.682	3.434	1.624	1.918	1.558	-0.677284546	0.000435767
LOC_Os06g40940	2.535	3.217	2.857	1.58	1.764	1.422	-0.676816528	0.0000424
LOC_Os07g47420	65.506	63.955	61.379	37.49	35.95	36.669	-0.676689169	2.27E-22
LOC_Os09g20850	0.916	1.124	0.68	0.253	0.213	0.551	-0.676548009	0.014835327
LOC_Os09g30070	3.003	2.849	2.955	1.622	1.837	1.527	-0.676478299	0.000000143
LOC_Os04g58230	0.835	1.187	0.732	0.315	0.142	0.622	-0.67596516	0.014247145
LOC_Os04g37980	1.369	1.23	0.934	0.369	0.623	0.66	-0.675896555	0.00857042
LOC_Os06g05470	3.781	5.979	3.733	7.505	6.612	7.475	0.67566717	0.000453375
LOC_Os02g26800	0.605	0.599	0.448	1.032	1.205	0.692	0.675547665	0.006210723
LOC_Os11g39430	4.991	4.131	4.978	7.01	7.707	6.698	0.675445935	1.07E-09
LOC_Os05g33320	11.01	9.577	11.445	4.014	3.568	7.736	-0.67520218	0.007121072
LOC_Os10g19334	2.777	2.287	2.276	1.284	1.368	1.289	-0.675177644	0.000110431
LOC_Os01g67740	8.843	8.409	7.232	4.018	4.951	5.03	-0.675059693	4.59E-11
LOC_Os02g47150	18.294	17.186	16.035	9.357	10.854	9.583	-0.674665504	4.4E-23
LOC_Os01g10370	2.36	3.202	3.057	1.237	1.856	1.296	-0.674468736	0.003540583
LOC_Os03g61270	0.436	0.499	0.4	1.036	0.951	0.635	0.674458232	0.011820849
LOC_Os03g55930	5.752	6.269	6.17	2.719	4.106	3.061	-0.674337844	0.000314406
LOC_Os01g46870	6.331	6.119	5.734	3.073	3.001	3.994	-0.674007959	0.0000281
LOC_Os12g42200	7.694	7.949	6.505	10.518	12.249	10.566	0.673750985	2.08E-12
LOC_Os02g38290	3.664	4.795	4.165	2.311	2.342	2.33	-0.673627565	0.0000347
LOC_Os03g21030	30.614	33.924	31.007	47.237	45.41	49.881	0.673552537	9.03E-26
LOC_Os02g07160	51.715	52.681	52.201	75.585	75.617	81.951	0.673518696	2.86E-40
LOC_Os06g14460	3.61	3.577	3.627	1.853	2.366	1.628	-0.672969731	0.00049114
LOC_Os01g51540	22.342	21.577	21.596	11.537	13.906	12.243	-0.672637156	9.59E-13
LOC_Os02g52860	177.966	175.622	169.232	98.615	100.793	104.631	-0.672491935	2.57E-56
LOC_Os08g42420	32.832	31.579	34.053	50.381	48.268	48.339	0.672434471	9.98E-42
LOC_Os12g39520	10.366	11.34	10.317	17.845	14.771	15.675	0.671884737	2.16E-10
MSTRG.1706	1.529	1.724	2.563	0.865	0.928	0.951	-0.671753194	0.008736945
MSTRG.8766	4.381	4.419	3.432	6.07	6.931	6.609	0.671615219	0.000599657

MSTRG.3972	2.093	2.146	2.239	3.472	2.978	3.352	0.671423372	2.35E-09
LOC_Os06g16290	110.251	107.891	104.497	60.118	65.482	61.688	-0.671336137	2.5E-28
LOC_Os11g12240	0.668	0.993	0.875	1.109	1.54	1.571	0.671336132	0.002577326
LOC_Os07g41790	12.647	14.06	12.944	5.675	9.275	7.162	-0.671206154	0.0000245
LOC_Os11g35240	1.34	0.866	2.511	2.361	3.646	2.522	0.671183522	0.007390045
LOC_Os10g26400	4.031	2.52	3.045	1.758	2.009	1.226	-0.6711653	0.001824688
LOC_Os01g73950	15.725	14.256	15.574	9.264	9.318	7.494	-0.67058782	4.19E-12
LOC_Os07g48229	7.01	7.603	6.862	11.127	10.977	10.168	0.670378942	5.24E-12
LOC_Os11g11210	2.023	1.571	2.356	0.58	0.535	1.528	-0.670084129	0.011407052
LOC_Os11g26760	20.292	19.645	21.074	30.353	30.752	30.599	0.669614346	1.03E-11
LOC_Os03g38250	0.796	0.619	0.601	0.964	1.236	1.084	0.669347141	0.001306851
LOC_Os04g40310	25.904	24.214	25.563	14.687	13.426	15.573	-0.669192783	5.13E-15
LOC_Os06g49300	0.997	1.646	0.948	0.712	0.433	0.466	-0.669116391	0.011190657
LOC_Os12g38320	2.888	3.126	3.951	2.186	1.738	1.322	-0.668543322	0.001867317
LOC_Os06g04200	7.045	6.86	7.788	11.876	11.748	9.233	0.668371096	1.48E-09
LOC_Os09g16995	0.231	0.247	0.13	0.446	0.434	0.353	0.667426899	0.013489634
LOC_Os03g63720	17.926	17.521	18.213	9.862	10.28	10.662	-0.667414004	5.16E-10
LOC_Os05g33310	22.765	22.556	19.478	12.955	12.977	11.259	-0.667369278	8.39E-10
LOC_Os11g19840	2.453	1.772	2.44	0.883	1.22	1.275	-0.66725596	0.004206625
LOC_Os06g12250	51.802	53.89	49.466	78.622	76.045	75.48	0.667068398	1.22E-31
LOC_Os03g19290	15.695	14.601	14.149	8.926	7.882	8.35	-0.666989941	0.000000313
LOC_Os05g32230	15.267	16.457	13.547	8.06	8.938	9.012	-0.66671096	1.47E-10
LOC_Os03g60500	1.816	2.637	1.906	4.493	3.568	3	0.666573089	0.005604977
LOC_Os04g54310	5.796	5.742	4.066	8.123	8.515	8.245	0.666413232	0.000450438
LOC_Os03g43580	3.909	3.271	3.345	1.592	1.274	2.625	-0.66615949	0.002029883
LOC_Os10g02380	99.163	101.345	102.544	146.053	151.554	150.836	0.66569167	1.61E-50
LOC_Os08g39350	24.437	23.38	22.479	36.126	34.875	33.195	0.665222205	1.55E-29
LOC_Os03g06080	2.248	1.965	2.789	4.592	3.814	3.28	0.665170526	0.002731866
LOC_Os02g46650	4.945	4.871	5.223	3.236	2.743	2.478	-0.665033664	0.0000128
LOC_Os04g47720	11.18	11.397	12.112	6.403	6.805	6.742	-0.66458547	1.14E-09
LOC_Os02g55480	5.319	5.327	5.762	7.357	9.404	8.084	0.664248858	4.56E-08
LOC_Os09g39180	7.99	10.011	7.597	4.772	4.134	5.452	-0.664025863	0.0000117
LOC_Os11g36160	0.924	1.108	1.071	1.799	1.665	1.331	0.663645593	0.000354794
LOC_Os01g63010	0.963	1.125	0.978	1.394	1.985	1.8	0.66347723	0.003849335
LOC_Os05g34830	13.571	13.93	13.835	20.127	20.198	21.362	0.663429719	5.55E-14

LOC_Os07g27780	31.428	33.457	32.313	17.122	18.598	20.548	-0.663346755	9.46E-14
LOC_Os03g50490	27.726	26.02	26.889	15.806	16.886	14.193	-0.662987898	5.79E-16
LOC_Os10g25040	21.918	21.276	19.763	11.913	11.924	12.199	-0.662798772	4.07E-18
LOC_Os04g50880	13.849	12.045	13.923	17.829	19.29	22.612	0.662775026	6.05E-10
LOC_Os11g14544	136.194	133.378	143.946	203.844	197.705	204.956	0.662521897	1.58E-63
LOC_Os01g09320	158.548	157.133	168.055	245.402	228.037	240.32	0.662499392	4.56E-56
LOC_Os07g35330	9.496	8.985	9.512	14.056	13.584	14.07	0.662349288	3.75E-14
LOC_Os02g51470	6.425	6.852	7.297	3.917	3.339	3.566	-0.662243088	0.0000367
LOC_Os02g57720	30.001	30.472	31.999	19.016	17.064	17.71	-0.662126195	6.08E-17
LOC_Os07g37320	143.001	144.843	142.044	207.923	204.52	221.553	0.662116402	4.8E-59
LOC_Os10g26060	98.081	95.133	122.373	173.508	165.558	141.333	0.662100941	0.00000164
LOC_Os06g31960	367.803	373.876	316.848	202.954	214.72	201.247	-0.662093297	3.36E-33
LOC_Os05g41180	37.548	37.015	33.08	19.368	22.733	20.412	-0.66199305	7.26E-15
LOC_Os01g47262	31.585	30.032	35.846	49.414	49.269	46.117	0.661982038	2.68E-19
LOC_Os01g31800	10.597	8.801	8.56	5.64	4.278	5.395	-0.661480048	0.000201925
LOC_Os11g47520	316.713	314.244	307.274	436.005	443.237	505.46	0.661462542	1.83E-41
LOC_Os08g15420	11.335	10.782	9.363	5.27	6.08	6.776	-0.660926661	7.82E-10
LOC_Os01g42460	5.923	4.794	5.236	2.755	2.87	3.386	-0.66081951	0.00000578
LOC_Os01g34870	4.646	4.97	4.523	2.578	2.533	2.707	-0.660392564	0.000159043
LOC_Os05g07940	54.365	55.982	56.858	79.996	81.752	80.245	0.660143222	5.63E-41
LOC_Os03g48560	7.763	9.039	6.861	4.362	4.343	4.655	-0.659996617	0.00000703
LOC_Os11g02740	2.191	2.792	2.036	1.037	1.101	1.637	-0.65987488	0.000780216
LOC_Os07g46630	4.608	2.965	3.31	2.062	1.904	1.799	-0.659820278	0.001772467
LOC_Os04g38810	2.939	2.954	2.133	1.469	1.261	1.678	-0.659734099	0.000236652
LOC_Os01g32330	23.453	26.911	24.949	14.552	14.407	14.689	-0.659390878	7.78E-12
LOC_Os12g43363	18.218	19.838	18.736	10.124	12.588	10.214	-0.659357066	1.71E-10
LOC_Os01g09120	10.886	10.61	10.427	15.883	15.498	16.078	0.65858161	1.02E-12
LOC_Os04g57320	11.507	9.842	9.467	15.559	15.999	14.382	0.658530321	9.08E-11
LOC_Os11g04720	1.902	1.86	1.662	0.984	1.027	0.376	-0.658510079	0.011800126
LOC_Os12g40180	24.1	20.176	20.726	31.63	28.863	37.068	0.658469353	2.87E-08
MSTRG.1112	1.064	1.785	2.102	2.607	2.322	4.136	0.658440304	0.009768759
LOC_Os01g67160	21.551	21.554	23.749	12.055	13.01	13.643	-0.658166029	1.13E-10
LOC_Os01g62190	7.509	4.893	6.327	10.377	8.269	10.372	0.658077917	0.0000986
LOC_Os10g35480	9.383	8.937	7.732	4.649	5.032	5.168	-0.657707046	0.000000852
LOC_Os10g21590	25.64	26.141	25.424	38.873	37.65	37.296	0.657588082	2.11E-25

LOC_Os01g71090	323.687	315.028	331.597	475.576	444.745	506.49	0.657026865	3.57E-47
LOC_Os12g38770	25.635	22.111	26.46	37.904	36.626	35.264	0.656940826	2.27E-19
LOC_Os11g30910	13.232	12.649	13.138	19.597	19.213	19.071	0.656676208	1.23E-14
LOC_Os01g24940	2.083	1.865	2.094	1.211	0.957	1.087	-0.656483873	0.001058869
LOC_Os06g45184	6.733	4.771	5.425	8.307	9.229	8.061	0.656443755	0.000000864
MSTRG.17014	5.265	6.594	5.909	10.687	7.991	8.358	0.656114793	0.00000564
LOC_Os06g02000	2.099	2.038	2.007	3.476	2.96	3.37	0.655820212	0.001554899
LOC_Os05g25540	9.656	10.637	9.494	5.301	5.808	6.045	-0.655509232	2.43E-08
LOC_Os02g09750	6.438	7.53	6.335	4.029	3.531	3.994	-0.655328654	0.00000262
LOC_Os01g04100	7.104	8.236	7.178	10.677	10.964	12.292	0.655321874	0.000000447
LOC_Os03g60080	84.717	79.816	93.42	125.625	127.115	126.905	0.654887628	9.3E-33
LOC_Os09g20820	35.041	32.485	31.988	19.489	18.681	20.089	-0.654554629	2.49E-20
LOC_Os10g25340	9.676	8.899	8.532	4.064	5.568	5.721	-0.654185768	0.0000112
LOC_Os08g29760	2.356	2.723	2.393	1.292	1.4	1.437	-0.654020828	0.000269642
LOC_Os10g40660	0.932	1.231	1.076	0.499	0.506	0.498	-0.653810864	0.011024581
LOC_Os12g35940	4.864	4.288	4.953	7.336	6.847	6.784	0.65370968	3.87E-12
LOC_Os08g20570	8.99	9.247	7.928	4.947	5.497	4.722	-0.653504677	1.36E-09
LOC_Os08g24400	8.086	7.683	7.995	11.788	12.155	11.429	0.653135786	9.7E-11
LOC_Os02g35210	0.739	0.936	0.734	1.347	1.245	1.314	0.652622555	0.001979016
LOC_Os03g11900	62.823	64.322	61.506	92.493	91.433	92.509	0.652593424	9.77E-55
LOC_Os04g22660	2.182	2.595	1.601	3.895	3.094	4.429	0.652402949	0.009242758
LOC_Os04g33390	57.854	55.721	55.976	32.77	32.94	34.026	-0.652210975	3.76E-29
LOC_Os03g21800	32.258	32.969	30.556	47.796	46.683	47.637	0.652207197	1.24E-26
LOC_Os09g31410	5.357	4.965	3.734	2.616	2.339	2.886	-0.65216581	0.000126119
LOC_Os07g03250	6.681	7.266	5.939	3.577	3.267	4.336	-0.651983948	0.0000403
LOC_Os01g54670	15.659	19.231	20.305	27.819	26.72	27.473	0.651904812	2.31E-11
LOC_Os04g56450	27.79	30.902	30.464	45.019	43.77	42.279	0.651770208	4.39E-27
LOC_Os04g36740	8.764	9.734	9.702	5.479	5.726	5.397	-0.651593073	2.98E-09
LOC_Os04g53760	10.569	10.255	9.763	5.731	6.244	5.88	-0.651356696	9.71E-13
LOC_Os09g36900	10.779	10.643	9.054	5.781	5.344	6.357	-0.651266878	0.000000411
LOC_Os09g17740	20.96	17.6	17.662	10.625	10.537	11.499	-0.650995376	2.62E-10
LOC_Os03g22490	181.787	179.943	195.639	264.816	265.239	286.241	0.650675697	1.35E-47
LOC_Os08g40430	3.223	4.848	3.951	1.883	2.781	2.068	-0.650612978	0.000171766
LOC_Os11g47640	4.362	2.327	3.435	4.093	5.421	6.657	0.650345908	0.001400262
LOC_Os06g43210	51.673	48.738	51.916	76.179	72.995	73.777	0.650260767	3.27E-51

LOC_Os07g23480	0.549	0.609	0.39	0.821	0.888	0.742	0.650251601	0.000871491
LOC_Os04g51390	3.203	3.305	3.234	1.801	2.171	1.113	-0.650133821	0.001439983
LOC_Os04g28480	13.895	15.262	15.595	22.463	23.025	20.456	0.649701329	1.63E-18
LOC_Os03g06390	57.997	57.879	61.307	89.199	83.548	86.822	0.649605057	9.48E-36
LOC_Os12g08850	0.445	0.571	0.408	0.708	0.897	0.969	0.64952524	0.009899434
LOC_Os05g41080	6.613	6.345	5.548	3.53	3.99	2.537	-0.649308314	0.001092477
LOC_Os09g37120	4.235	4.337	4.45	2.249	1.861	2.961	-0.648557229	0.000933472
LOC_Os04g38640	0.86	0.789	0.854	0.367	0.305	0.406	-0.648543704	0.014472095
LOC_Os09g36619	5.099	4.86	5.401	6.903	7.685	7.806	0.648324342	0.000000125
LOC_Os11g01300	45.439	43.973	49.251	30.097	21.399	27.659	-0.64821261	0.00000519
LOC_Os06g07020	2.416	2.939	2.664	1.593	1.405	1.379	-0.648162414	0.000836726
LOC_Os07g13370	1.231	0.677	0.752	1.583	2.674	2.22	0.648104349	0.016064866
LOC_Os03g25270	28.522	27.921	32.726	43.609	43.401	41.557	0.647805933	7.67E-19
LOC_Os01g53930	12.697	12.309	12.195	7.189	7.098	7.489	-0.647597229	8.25E-14
LOC_Os04g54300	139.707	134.122	135.078	189.99	211.197	197.113	0.647326332	1.35E-34
LOC_Os03g49260	5.834	6.169	5.742	8.865	9.095	8.271	0.647153896	7.53E-12
LOC_Os11g11050	16.669	15.38	14.092	8.926	9.346	8.586	-0.64708218	1.43E-09
LOC_Os01g59060	6.41	9.641	9.488	3.874	6.192	3.9	-0.646870633	0.000988693
LOC_Os04g10650	5.929	6.721	5.705	3.684	3.238	3.613	-0.646378441	0.00000169
LOC_Os02g48340	12.945	13.031	12.127	18.265	19.862	17.638	0.646283304	1.71E-15
LOC_Os04g49780	4.36	5.268	4.648	2.291	3.085	2.845	-0.64615608	0.000000982
LOC_Os01g67860	1215.598	1192.617	1228.084	1840.3	1742.589	1721.689	0.645995325	7.07E-75
LOC_Os01g55700	14.005	13.685	12.415	7.763	8.058	7.502	-0.645578455	7.46E-09
LOC_Os01g33450	3.951	4.502	4.71	1.906	2.318	2.406	-0.645422671	0.006550794
LOC_Os02g57870	6.89	6.659	5.558	3.818	3.304	3.809	-0.645415295	0.00000722
LOC_Os01g55510	61.064	70.412	65.978	91.7	96.682	100.981	0.6454137	8.82E-21
LOC_Os02g29400	85.742	89.616	85.884	53.128	51.804	49.118	-0.645394642	1.04E-19
LOC_Os12g24020	33.63	33.128	34.521	46.913	52.419	48.939	0.644643598	1.19E-23
LOC_Os01g16310	0.826	0.483	0.843	0.833	1.229	1.523	0.644543625	0.004346514
LOC_Os09g04504	33.749	31.937	36.327	50.808	49.084	49.112	0.644222122	2.27E-32
LOC_Os12g23200	1.032	0.79	0.645	0.317	0.367	0.211	-0.644072509	0.01622979
LOC_Os06g37620	16.524	17.119	18.206	25.931	24.325	25.602	0.64377075	5.24E-23
LOC_Os06g03940	17.086	20.578	21.782	29.413	27.215	30.765	0.643743441	2.17E-14
LOC_Os12g15350	0.099	0.081	0.181	0.286	0.386	0.217	0.643536714	0.01634424
LOC_Os10g39740	1.432	1.514	1.663	0.829	0.607	0.732	-0.643319939	0.011260455

LOC_Os01g34010	0.525	0.421	0.55	0.652	1.003	1.015	0.643244992	0.009494877
LOC_Os07g39260	9.559	8.962	8.801	5.221	5.601	4.952	-0.643236112	0.00000102
LOC_Os03g40194	18.232	18.872	18.807	28.329	27.251	26.048	0.643221302	5.97E-30
LOC_Os06g40170	54.616	59	55.25	32.634	32.917	34.524	-0.643110175	5.21E-36
LOC_Os04g57860	22.067	22.595	16.442	10.873	12.875	11.97	-0.643059466	3.41E-10
LOC_Os01g59880	2.608	2.79	2.026	1.126	1.466	1.547	-0.642881129	0.000163793
LOC_Os07g12250	126.719	130.168	130.765	72.838	80.342	76.558	-0.642766429	7.12E-30
LOC_Os05g45110	5.906	5.675	5.154	3.878	2.505	3.048	-0.642710221	0.0000939
LOC_Os08g39870	3.288	2.245	2.4	1.293	1.342	1.641	-0.642685895	0.001627426
ChrSy.fgenesh.gene.37	13.383	13.523	15.875	21.566	20.75	20.487	0.64248087	5.02E-16
LOC_Os02g41860	3.399	2.794	2.626	1.612	1.294	1.928	-0.642384528	0.001845969
LOC_Os12g12290	1.419	1.573	0.991	0.587	0.826	0.518	-0.642310658	0.009572751
LOC_Os02g41780	4.461	3.902	4.538	7.498	5.726	6.256	0.641269687	0.0000128
LOC_Os01g16130	6.649	5.389	6.47	9.586	9.43	8.494	0.641131559	3.65E-08
LOC_Os02g16500	6.311	7.296	7.957	2.791	3.6	3.321	-0.640674281	0.013603253
LOC_Os07g11380	157.909	155.226	192.03	272.674	254.525	224.915	0.640341782	0.00000022
LOC_Os03g11380	9.479	8.172	5.749	4.444	4.345	4.341	-0.640300916	0.000102578
LOC_Os11g07124	0.904	0.84	1.071	1.199	1.492	1.784	0.64003032	0.001432542
LOC_Os07g23960	2.567	2.831	2.885	4.634	3.806	4.369	0.639974763	0.000318599
LOC_Os05g43810	1.016	1.032	1.328	2.157	1.64	1.627	0.63990863	0.001966029
LOC_Os12g10710	8.604	8.75	8.376	12.835	12.93	12.136	0.639629071	3.05E-14
LOC_Os07g23900	3.262	3.147	2.729	1.518	1.763	1.807	-0.638726767	0.000402682
LOC_Os03g04970	28.097	27.303	23.85	14.397	16.178	16.244	-0.638670272	3.64E-16
LOC_Os08g08084	2.978	2.235	3.309	4.527	5.029	4.557	0.638605527	0.004260816
LOC_Os04g51110	13.055	13.683	11.786	7.191	7.33	8.088	-0.638341501	5.68E-11
LOC_Os01g06010	99.47	96.396	94.055	51.432	58.926	61.437	-0.638252713	2.13E-21
LOC_Os03g28160	34.187	30.271	31.478	18.837	18.633	18.92	-0.638230619	5.25E-11
LOC_Os07g46720	4.894	3.32	5.135	6.678	6.326	6.964	0.638032349	0.00000155
LOC_Os07g42520	22.688	24.125	20.485	11.678	13.798	13.725	-0.638003274	6.39E-08
LOC_Os06g23650	2.706	2.062	1.717	1.054	0.661	1.408	-0.637923874	0.009898774
LOC_Os10g40510	3.797	3.549	3.941	4.976	5.604	7.236	0.637668699	0.00112422
LOC_Os04g56060	7.696	6.377	6.543	3.611	3.899	4.322	-0.637438132	0.0000104
LOC_Os10g39110	2.697	4.55	2.673	1.195	1.853	1.841	-0.637302937	0.008748193
LOC_Os07g48510	72.03	69.429	78.208	107.177	101.984	110.304	0.636809676	6.17E-30
LOC_Os01g57900	1.558	1.495	1.678	0.883	0.822	0.826	-0.636790463	0.002734975

LOC_Os03g51640	27.996	27.036	29.425	43.147	39.856	39.887	0.636777175	7.56E-25
MSTRG.1424	3.143	3.279	3.213	5.581	4.243	4.691	0.636704007	0.0000189
LOC_Os05g29900	18.842	22.877	20.841	11.83	12.225	12.516	-0.636662902	2.09E-08
LOC_Os02g07870	32.016	28.02	28.541	15.883	18.679	17.944	-0.63664029	3.75E-18
LOC_Os03g48300	18.088	17.686	19.75	26.602	26.902	27.227	0.636322758	1.46E-27
LOC_Os04g47300	1.718	1.362	1.747	2.953	2.413	2.241	0.636294845	0.001200143
LOC_Os12g07680	6.011	6.65	5.748	3.167	4.039	3.326	-0.636233915	0.0000339
LOC_Os03g16450	1.446	1.332	1.066	0.503	0.782	0.718	-0.636233605	0.004783207
LOC_Os07g43604	2.543	1.69	2.517	3.541	4.126	2.815	0.63588708	0.000449783
LOC_Os01g50820	2.124	1.64	2.226	2.981	3.453	2.812	0.635763466	0.00029812
LOC_Os09g16330	6.884	7.196	6.245	9.885	9.258	10.117	0.635449777	2.65E-13
LOC_Os08g15030	13.953	11.763	11.072	6.652	7.699	7.065	-0.635214098	0.000000308
LOC_Os06g09820	1.175	1.164	0.915	2.096	1.838	1.854	0.635104339	0.010305858
LOC_Os09g12650	1.394	1.72	1.713	2.107	3.01	2.42	0.635042478	0.000696508
LOC_Os02g46640	34.051	31.102	36.001	51.711	46.307	49.519	0.63503631	3.56E-16
LOC_Os09g04440	18.413	17.824	20.685	27.195	27.844	27.723	0.635021738	2.11E-28
LOC_Os03g04080	18.905	16.668	22.221	28.764	27.526	29.219	0.634887391	3.71E-08
MSTRG.6692	19.08	18.104	17.292	9.08	11.719	11.24	-0.634588195	5.24E-10
LOC_Os01g65560	10.097	9.042	9.97	5.183	6.202	5.254	-0.633550522	0.0000587
LOC_Os02g27360	32.944	33.691	35.262	49.949	50.474	47.361	0.633189131	1.46E-28
LOC_Os10g01720	3.556	4.205	3.926	1.77	2.393	2.345	-0.63307328	0.000607237
LOC_Os05g34800	0.782	1.316	1.267	0.701	0.39	0.58	-0.633065403	0.009117366
LOC_Os07g39990	4.812	3.796	4.081	6.869	7.041	6.775	0.632653213	0.003018352
LOC_Os07g06620	6.42	7.254	7.255	9.66	8.983	12.516	0.632329027	0.00000446
LOC_Os04g32010	19.833	18.3	18.825	11.062	11.502	11.226	-0.631853752	6.51E-15
LOC_Os06g05090	34.976	37.53	36.961	22.105	22.968	20.033	-0.631519799	7.88E-17
LOC_Os02g18390	29.769	28.267	28.659	17.072	14.474	19.167	-0.631071908	0.000000064
LOC_Os02g25640	78.29	74.13	78.935	112.903	120.357	101.752	0.631011668	1.48E-29
LOC_Os12g38940	1.391	1.35	0.885	0.712	0.666	0.41	-0.630948641	0.009500513
LOC_Os09g38710	5.522	6.187	5.036	2.66	3.7	3.459	-0.63088505	0.000000322
LOC_Os08g36480	10.819	11.974	10.413	15.738	15.926	16.54	0.630719598	2.51E-17
LOC_Os01g42650	104.2	95.963	101.759	64.762	55.716	59.533	-0.630567872	1.78E-22
LOC_Os08g37115	5.169	5.687	5.204	7.924	7.597	8.208	0.630454055	0.00000029
LOC_Os07g44190	68.469	66.404	66.959	38.644	41.451	40.652	-0.630430549	8.06E-37
LOC_Os12g38350	9.405	7.648	8.46	5.393	4.858	3.921	-0.630388671	0.00086979

LOC_Os06g50230	9.084	9.389	10.985	14.163	13.3	16.075	0.630364674	0.000000218
LOC_Os09g33630	9.903	9.203	10.297	13.856	16.105	13.442	0.630025828	0.000000109
LOC_Os03g06740	9.892	9.962	9.813	6.232	5.629	5.607	-0.629924963	5.25E-09
LOC_Os07g07620	1.699	1.515	1.636	0.84	0.872	0.939	-0.629905127	0.001649682
LOC_Os07g47370	1.458	0.963	1.572	0.74	0.625	0.692	-0.629836943	0.006282677
LOC_Os02g48870	45.914	51.084	45.518	27.192	27.343	30.396	-0.629611863	1.19E-20
LOC_Os05g46750	1.553	1.445	0.93	0.826	0.682	0.4	-0.628761964	0.010663676
LOC_Os09g34930	13.599	11.922	13.382	18.614	18.658	19.424	0.628407838	3.86E-12
LOC_Os12g36220	602.77	599.672	577.707	337.828	384.422	345.294	-0.628293194	2.58E-42
LOC_Os03g06850	29.466	28.516	33.028	44.929	44.417	43.382	0.628277196	2.04E-16
LOC_Os03g47280	10.437	11.098	9.725	15.308	16.595	14.694	0.628256183	0.00000747
LOC_Os11g30500	2.479	2.714	2.572	4.908	3.891	3.648	0.628211537	0.002754884
LOC_Os03g40540	131.816	128.095	136.583	193.938	187.121	190.263	0.62820947	1.05E-54
LOC_Os06g39370	4.72	4.846	5.895	7.853	6.923	8.205	0.627526771	0.0000034
LOC_Os04g46980	26.11	26.761	25.436	14.972	15.078	16.55	-0.627463879	1.9E-15
LOC_Os03g61810	18.097	18.861	19.297	9.441	12.387	11.123	-0.627404635	0.000000471
LOC_Os10g13850	116.31	115.801	128.094	175.835	162.751	181.358	0.627338113	3.21E-29
LOC_Os10g35950	0.981	1.543	1.583	0.784	0.695	0.549	-0.62729163	0.009981139
LOC_Os01g47040	4.226	2.521	3.677	2.288	2.024	1.283	-0.627279096	0.003340421
LOC_Os07g39520	14.805	13.757	12.621	19.456	18.586	21.978	0.626971614	1.89E-10
ChrSy.fgenesh.gene.47	18.359	22.192	17.338	28.692	28.439	29.473	0.626850364	0.0000324
LOC_Os11g02200	3.027	4.713	3.965	7.927	8.246	5.07	0.626831891	0.012021507
LOC_Os04g53790	0.065	0.27	0.387	0.619	0.37	0.434	0.626703207	0.015128117
LOC_Os02g10520	42.574	42.269	41.387	58.545	59.575	63.734	0.62664575	8.31E-37
LOC_Os09g26554	14.997	16.424	17.4	9.018	10.473	9.37	-0.626619459	1.47E-09
LOC_Os01g07500	21.191	23.765	21.364	13.014	12.41	14.019	-0.626536535	4.17E-14
LOC_Os05g42070	5.673	7.613	8.823	4.364	3.862	4.08	-0.626391292	0.000943566
LOC_Os05g45280	25.573	23.786	23.532	36.007	34.424	35.158	0.626271922	3.88E-16
LOC_Os07g10240	1.245	1.08	1.534	0.526	0.609	0.702	-0.626246506	0.01226088
LOC_Os05g45070	1.263	0.655	1.158	0.348	0.235	0.488	-0.626191079	0.016699393
LOC_Os01g42990	9.521	10.976	10.239	5.834	5.592	6.643	-0.62585003	5.25E-08
LOC_Os11g31980	5.186	5.194	4.964	3.051	3.037	2.801	-0.625843173	0.0000145
LOC_Os01g01170	4.577	3.841	4.105	2.196	2.446	2.572	-0.625798902	0.0000374
LOC_Os11g02130	6.263	7.413	6.809	9.027	10.499	10.795	0.625797525	0.0000019
LOC_Os11g42960	4.139	6.676	4.987	2.461	3.153	3.169	-0.6255938	0.000957535

LOC_Os03g07890	26.075	26.313	25.336	38.18	36.74	37.366	0.625502019	2.05E-20
LOC_Os07g10810	1.725	2.06	1.395	3.327	2.331	2.791	0.625393511	0.004662235
LOC_Os12g35630	4.062	4.665	4.586	3.064	2.745	2.546	-0.62526185	9.53E-08
LOC_Os06g02210	8.973	8.542	7.689	5.431	4.846	4.496	-0.62523905	0.000000459
LOC_Os03g40440	0.521	0.344	1.036	1.257	2.039	1.254	0.62484435	0.017038651
LOC_Os10g30280	4.975	4.603	4.712	2.38	2.975	2.744	-0.624491991	0.000303423
LOC_Os06g03670	2.849	1.796	2.28	3.648	3.444	3.878	0.624473303	0.002271264
LOC_Os04g25960	3.144	3.085	3.98	1.82	2.166	1.782	-0.624390807	0.000469192
LOC_Os02g48094	2.046	2.684	3.193	1.44	1.568	1.047	-0.624083179	0.007733283
LOC_Os02g43600	8.964	12.423	9.884	6.623	5.326	6.038	-0.62378157	0.0000549
LOC_Os09g39550	52.855	54.335	54.112	29.801	34.779	31.759	-0.623747158	7.92E-15
LOC_Os08g23754	1.89	1.618	2.2	3.816	3.478	3.27	0.623716814	0.00296163
LOC_Os03g18710	1.187	1.461	1.1	0.832	0.623	0.631	-0.623482975	0.000965121
LOC_Os11g05494	4.731	4.368	5.126	6.638	6.308	9.697	0.623443404	0.002752801
LOC_Os11g35710	29.56	28.511	30.719	41.274	44.837	41.78	0.623211525	3.35E-29
LOC_Os05g03830	35.791	35.393	35.77	19.621	22.022	22.374	-0.623063067	2.07E-18
LOC_Os12g02200	65.615	70.038	68.379	97.515	100.923	94.81	0.623030959	1.61E-43
LOC_Os10g20710	1.809	1.656	1.868	0.883	1.142	0.909	-0.622827887	0.001796982
MSTRG.5784	3.556	3.628	3.873	5.292	5.275	5.578	0.622766745	3.47E-09
LOC_Os02g13520	4.494	4.754	4.923	2.609	3.314	2.13	-0.622531063	0.000360291
LOC_Os09g35600	1.185	1.174	1.416	0.658	0.492	0.68	-0.622497799	0.011479277
LOC_Os09g28000	24.398	25.183	23.256	13.811	13.998	15.668	-0.622489503	1.58E-14
LOC_Os05g31760	7.72	7.285	6.462	3.947	3.816	4.636	-0.622169417	0.0000219
LOC_Os10g09110	69.195	68.932	65.056	41.422	36.486	40.437	-0.621557065	2.39E-25
LOC_Os06g36560	129.481	132.771	129.278	183.576	188.036	189.68	0.621485105	2.94E-60
LOC_Os04g40410	17.594	19.089	18.865	25.11	24.314	31.802	0.620993523	6.79E-08
LOC_Os08g37104	2.558	2.982	2.966	3.629	4.524	4.784	0.620819313	0.000309353
LOC_Os08g41520	3.173	2.138	2.502	3.551	3.661	5.323	0.620799326	0.003670136
LOC_Os02g30780	8.085	8.648	6.232	12.079	11.054	11.163	0.620756782	0.0000511
LOC_Os03g27230	46.523	50.795	52.516	29.797	29.042	31.189	-0.620723051	6.32E-24
LOC_Os03g05920	18.977	19.103	17.709	11.804	11.734	9.698	-0.620427788	1.01E-10
LOC_Os03g07870	9.02	6.305	8.496	10.728	12.626	12.147	0.62041494	0.0000224
LOC_Os06g36360	141.881	143.368	144.552	205.369	205.735	207.681	0.619945569	3.14E-79
LOC_Os05g51830	213.204	203.215	202.725	124.818	125.371	123.381	-0.619829698	4.66E-52
LOC_Os11g05556	3.246	3.929	3.591	2.165	2.042	1.944	-0.619656651	0.000245997

LOC_Os09g27950	4.559	5.145	5.08	7.789	7.802	6.253	0.619254941	0.00000306
LOC_Os03g63999	1.344	2.33	1.463	2.917	3.368	2.727	0.618974198	0.010897521
MSTRG.21563	1.808	2.44	1.772	3.067	3.17	2.98	0.618545018	0.000635239
LOC_Os08g42750	22.936	22.917	24.204	33.026	33.268	34.23	0.618072742	1.89E-28
LOC_Os03g04680	20.922	19.465	19.106	12.34	11.07	12.187	-0.617358632	1.82E-13
LOC_Os10g05069	104.06	104.438	99.077	149.047	145.848	144.191	0.617320414	6.03E-62
LOC_Os06g39260	31.06	36.635	35.405	21.314	21.268	19.272	-0.617227433	2.21E-14
LOC_Os01g24710	3077.154	3115.604	3360.733	4741.136	4590.339	4326.809	0.617203624	1.31E-49
LOC_Os04g01990	4.092	3.964	4.732	2.215	2.734	2.501	-0.617095704	0.0000237
LOC_Os06g02040	12.763	12.987	15.181	17.97	21.165	21.155	0.616912942	0.0000022
LOC_Os02g27592	1.32	2.111	1.936	2.479	3.468	2.426	0.616901605	0.002782566
LOC_Os05g48810	12.942	12.93	13.316	18.103	20.845	17.8	0.616887595	1.98E-11
LOC_Os11g30810	9.862	10.232	10.791	6.956	5.277	5.832	-0.6168151	0.00000782
LOC_Os05g20930	2.263	1.739	1.397	0.802	1.265	0.542	-0.616729066	0.012367297
LOC_Os02g54820	46.357	48.558	50.727	67.744	67.802	72.678	0.616520293	1.04E-38
LOC_Os06g41010	638.572	627.644	674.146	946.206	928.042	896.949	0.616423639	4.21E-61
LOC_Os01g49160	38.645	41.61	44.196	63.347	57.815	57.617	0.616389473	2.89E-20
LOC_Os05g41550	53.432	50.4	48.86	68.538	76.991	74.22	0.616141589	8.19E-39
LOC_Os05g33010	6.25	4.523	4.851	8.099	7.88	7.468	0.616084328	0.000126075
LOC_Os02g35100	7.351	8.206	7.616	4.766	4.668	4.305	-0.616067482	6.69E-08
LOC_Os03g26870	11.962	12.456	12.314	16.073	16.761	18.364	0.615761883	5.32E-14
LOC_Os04g44100	4.487	4.066	4.565	7.443	5.939	6.415	0.615611155	0.000234119
LOC_Os01g37090	13.766	14.038	11.977	7.27	7.762	7.794	-0.615278073	0.000176289
LOC_Os05g02790	2.444	2.273	1.879	1.088	1.434	1.221	-0.615268962	0.000485933
LOC_Os12g04010	13.278	14.588	12.071	8.019	8.156	7.648	-0.615201155	3.22E-10
LOC_Os03g10200	21.315	21.338	20.656	13.541	13.268	11.462	-0.614991461	7.09E-10
LOC_Os06g40710	6.435	5.808	5.922	3.136	3.913	3.55	-0.61474166	0.0000158
LOC_Os11g01530	41.945	42.099	40.165	25.123	24.657	25.476	-0.614728268	1.89E-18
LOC_Os05g43390	5.11	3.832	4.659	2.369	2.402	3.055	-0.614629122	0.000129474
LOC_Os03g19190	4.082	4.51	3.221	2.208	2.395	2.354	-0.614624784	0.00000118
LOC_Os09g15835	5.25	4.928	6.044	7.807	8.53	7.619	0.614588	0.00000633
LOC_Os07g46700	5.635	5.163	6.327	8.615	7.572	8.489	0.61455641	0.000000398
LOC_Os04g35840	8.069	7.319	7.48	10.303	11.502	11.09	0.614454001	8.27E-15
LOC_Os03g29190	77.501	73.784	76.463	106.326	113.545	105.439	0.614103542	6.03E-44
LOC_Os08g25720	331.405	329.143	324.335	478.376	474.475	451.541	0.614043014	3.23E-74

LOC_Os06g08080	164.466	165.106	157.159	97.055	96.091	101.756	-0.61373393	1.99E-54
LOC_Os01g64330	7.625	7.877	7.845	11.661	10.507	11.508	0.61365474	1.54E-11
LOC_Os01g43040	4.17	3.903	3.439	2.123	2.377	2.211	-0.613617594	0.000025
LOC_Os10g08780	104.926	112.162	102.405	59.756	64.571	68.145	-0.613541723	9.59E-19
LOC_Os02g44010	15.619	18.801	19.939	26.249	26.761	26.221	0.613509605	1.07E-12
LOC_Os07g20164	50.949	55.279	53.127	29.816	32.49	33.879	-0.613488604	1.58E-22
LOC_Os02g03010	1.942	2.719	2.632	1.425	1.115	1.333	-0.613378251	0.005314521
LOC_Os07g35510	5.704	6.062	5.118	8.795	7.508	8.222	0.613069897	5.81E-08
LOC_Os10g37210	19.489	16.507	22.407	29.389	28.156	26.684	0.612971257	2.36E-12
LOC_Os08g37920	22.457	23.027	22.557	32.818	30.968	33.307	0.612620338	2.91E-27
LOC_Os07g29780	3.768	3.886	3.525	1.92	2.591	1.969	-0.612537409	0.000170191
LOC_Os03g04890	15.394	16.61	17.264	23.242	23.111	24.238	0.612288372	3.05E-19
LOC_Os01g44394	9.777	10.325	9.628	5.628	6.728	5.189	-0.612274609	0.00000268
LOC_Os06g19444	6.318	6.488	6.446	9.597	9.057	9.228	0.612235512	3.21E-09
LOC_Os12g07710	11.373	12.865	13.382	8.137	7.507	5.983	-0.612071058	0.000263507
LOC_Os01g24700	42.433	38.212	34.563	19.955	25.708	22.84	-0.611597559	6.83E-08
LOC_Os02g26700	327.95	324.493	351.76	474.759	449.914	505.816	0.611580345	1.01E-39
LOC_Os02g35500	12.286	14.736	12.366	7.753	7.497	8.04	-0.61148434	0.000000697
LOC_Os05g33440	5.516	5.466	6.36	2.056	3.392	4.334	-0.611446774	0.000852429
LOC_Os06g05920	3.903	3.813	5.144	6.458	5.804	6.673	0.611392526	0.00000693
LOC_Os09g27010	10.522	11.173	11.451	16.887	15.225	15.699	0.611366618	7.34E-11
LOC_Os11g03484	2.437	1.922	2.563	3.485	3.176	4.203	0.61132609	0.002729755
MSTRG.4137	25.663	25.985	28.831	40.365	35.561	39.436	0.611251	2.63E-17
LOC_Os02g03840	4.997	4.401	4.112	6.13	7.039	6.209	0.610925038	4.81E-15
MSTRG.7367	15.674	15.331	15.54	22.455	22.148	21.981	0.610776842	9.33E-20
LOC_Os03g06170	0.805	1.14	1.951	2.83	2.081	1.971	0.610749128	0.011866769
LOC_Os03g18264	16.008	15.081	17.551	24.717	22.153	22.702	0.610291623	6.3E-20
LOC_Os04g31520	1.472	2.106	1.349	0.789	0.72	0.442	-0.610234456	0.017814228
LOC_Os11g28950	1.061	0.572	0.529	0.346	0.381	0.352	-0.610233481	0.010598564
LOC_Os05g36270	13.209	13.61	11.894	16.448	19.79	19.44	0.609920683	5.31E-10
LOC_Os02g28340	18.634	17.985	17.432	11.165	11.586	9.692	-0.609870014	1.53E-10
LOC_Os05g09520	0.782	1.433	1.003	0.415	0.535	0.493	-0.609868865	0.016193045
LOC_Os09g33680	26.992	27.179	28.034	40.682	38.027	38.166	0.609611657	7.96E-24
LOC_Os05g33400	135.395	131.965	130.641	182.685	177.502	206.779	0.609568617	1.2E-30
LOC_Os03g16960	624.273	596.63	719.301	993.372	900.061	907.947	0.609474082	1.59E-10

LOC_Os07g46846	27.4	31.465	31.182	18.703	16.646	18.85	-0.609440399	3.53E-13
LOC_Os04g42000	32.327	32.508	31.605	20.532	18.452	19.002	-0.609421426	3.41E-12
LOC_Os01g53294	15.13	14.014	14.469	20.589	21.998	19.816	0.609394591	3.94E-18
LOC_Os06g11720	2.17	2.059	2.326	3.155	2.929	3.832	0.609230288	0.000486687
LOC_Os01g52290	4.366	4.784	4.872	6.909	6.371	7.084	0.60905967	0.00000013
LOC_Os11g07440	19.308	22.35	20.724	12.766	12.526	12.684	-0.609009379	3.28E-14
LOC_Os09g14670	16.148	15.715	15.582	9.079	10.163	9.435	-0.608961292	4.62E-17
LOC_Os03g11510	8.539	9.428	7.957	4.539	5.487	5.281	-0.60864994	0.00000289
LOC_Os01g11952	6.143	5.849	5.881	3.879	3.198	3.583	-0.608599534	0.000000026
LOC_Os11g31700	7.856	7.207	7.323	10.811	11.038	10.534	0.608537188	2.44E-08
LOC_Os11g32369	0.637	0.715	0.973	1.024	1.308	1.417	0.608529962	0.004818465
LOC_Os12g24550	5.408	5.282	4.016	2.454	3.153	3.105	-0.608201868	0.000000897
LOC_Os08g38080	1.336	1.528	1.131	0.446	0.402	0.881	-0.608200133	0.017070348
LOC_Os02g47670	6.659	6.056	6.501	8.249	9.709	9.736	0.608054557	9.67E-11
LOC_Os01g16520	9.441	8.728	8.699	13.47	13.653	11.537	0.607946218	5.62E-11
LOC_Os01g48910	49.685	49.915	50.29	71.545	71.047	69.885	0.607880056	1.23E-42
LOC_Os11g02610	21.359	20.642	20.494	12.919	11.288	13.422	-0.607837635	3.84E-13
LOC_Os07g37470	1.545	1.236	1.568	2.201	2.328	1.972	0.607534406	0.00014914
LOC_Os01g67100	7.459	6.83	5.81	3.458	4.352	4.17	-0.607523106	6.14E-08
LOC_Os06g43520	9.067	9.369	8.69	4.947	5.798	5.503	-0.607430652	3.1E-09
LOC_Os02g18520	1.242	1.23	1.301	1.907	2.403	1.577	0.607129849	0.002148792
LOC_Os07g29330	28.314	29.228	27.327	39.907	36.864	41.179	0.606865446	2.85E-21
LOC_Os02g09480	27.6	31.21	31.138	17.256	18.026	19.063	-0.606695699	1.08E-14
LOC_Os06g08770	31.623	30.947	28.34	19.087	17.443	18.4	-0.606543212	1.27E-15
LOC_Os01g38530	29.443	29.579	32.488	44.256	45.13	42.227	0.606460931	2.07E-27
LOC_Os05g28510	35.862	37.076	33.711	20.622	21.419	22.709	-0.606108573	2.81E-25
LOC_Os03g20680	81.689	81.986	77.444	46.928	50.366	49.236	-0.605728646	4.24E-27
LOC_Os02g15750	4.638	4.655	5.158	2.771	3.336	2.321	-0.605563297	0.000110672
LOC_Os04g57840	1.296	1.569	1.474	1.991	2.37	2.251	0.605360735	0.000885317
LOC_Os12g12664	1.536	1.369	1.632	2.6	2.062	2.409	0.605134622	0.000230929
LOC_Os09g37600	19.932	20.911	19.128	13.339	9.093	13.273	-0.604903	0.00000019
LOC_Os05g34260	64.896	62.84	65.423	90.686	91.184	91.819	0.604852967	1.56E-59
LOC_Os04g57300	70.23	65.756	74.555	104.319	97.516	96.897	0.604818075	3.32E-32
LOC_Os05g28840	7.593	8.59	6.961	4.454	4.164	4.812	-0.604646983	0.000124625
LOC_Os01g54570	18.682	16.849	16.47	9.878	9.31	11.665	-0.604449756	0.00000104

LOC_Os01g61920	69.145	67.773	59.435	39.607	41.497	37.62	-0.604116453	1.88E-14
LOC_Os03g21210	17.678	16.943	17.797	25.339	25.487	23.87	0.603966028	1.33E-18
LOC_Os01g70080	6.941	7.617	6.435	10.94	8.957	10.227	0.603871435	1.14E-09
LOC_Os01g09020	21.125	20.121	22.448	30.122	30.337	30.134	0.603362861	1.72E-22
LOC_Os01g74170	3.164	2.483	3.24	1.585	1.446	1.853	-0.603255647	0.003186582
LOC_Os04g42670	12.429	13.065	12.335	17.47	17.621	18.804	0.602867085	7.9E-16
LOC_Os10g38580	14.38	15.558	14.186	8.212	8.324	9.649	-0.602714555	0.0000019
LOC_Os01g71700	3.723	3.816	3.384	2.167	1.726	2.345	-0.602207555	0.00060138
LOC_Os03g01350	14.226	14.883	15.333	20.715	22.846	19.741	0.602055009	6.34E-18
LOC_Os03g56060	35.245	34.492	33.904	50.324	47.767	48.746	0.601895173	5.82E-32
LOC_Os03g57145	27.817	30.17	30.018	39.094	43.431	44.085	0.601717926	8.05E-09
LOC_Os03g27019	35.478	35.656	42.639	53.804	54.022	53.907	0.601631911	1.08E-21
LOC_Os07g28400	15.765	17.974	16.369	9.237	11.333	9.154	-0.601481218	0.00000649
LOC_Os08g21840	30.763	31.338	26.952	17.323	18.316	18.168	-0.601370999	6.11E-12
LOC_Os12g42850	55.179	57.39	55.938	80.48	81.174	76.861	0.601323928	6.52E-43
LOC_Os06g35630	2.434	2.509	2.081	3.871	4.052	2.729	0.601320693	0.000829807
LOC_Os06g23870	142.264	128.542	136.993	190.652	195.842	187.377	0.600740894	5.29E-47
LOC_Os01g55690	168.791	161.345	202.37	280.507	263.91	228.774	0.600704092	0.00000205
LOC_Os12g29950	16	16.192	14.957	8.858	9.405	10.137	-0.60065178	7.51E-11
LOC_Os07g44960	1.378	1.783	1.685	0.591	1.011	0.863	-0.600559835	0.010596816
LOC_Os11g05930	21.456	20.707	21.256	29.78	31.433	28.765	0.600376813	2.64E-21
LOC_Os12g36910	25.024	23.915	25.046	35.582	36.379	33.042	0.600320102	1.4E-22
LOC_Os08g36820	2.532	2.23	1.29	1.131	0.963	0.93	-0.600219848	0.011620305
LOC_Os06g06180	2.936	3.075	3.384	1.942	1.312	1.929	-0.600131469	0.003089845
LOC_Os02g19200	4.138	3.285	3.291	5.146	4.826	5.418	0.599984087	2.51E-08
LOC_Os12g41680	1.029	0.717	0.699	1.287	1.379	1.512	0.599941652	0.011318695
LOC_Os10g25570	7.465	8.778	7.655	9.49	12	13.865	0.599607649	0.000135559
LOC_Os03g12950	5.183	4.283	6.073	7.982	6.795	7.756	0.599256437	0.00000131
LOC_Os03g63010	4.677	5.438	4.763	6.512	6.672	6.186	0.59917809	0.000000113
LOC_Os05g33840	6.132	5.903	5.462	3.608	3.381	3.451	-0.598934388	0.00000099
LOC_Os11g03460	6.518	6.677	6.15	3.32	3.915	4.281	-0.59860758	0.00000163
LOC_Os02g35300	1.829	2.718	2.498	0.983	1.341	1.669	-0.598383729	0.001299971
LOC_Os03g44170	1.02	0.914	1.113	0.234	0.57	0.438	-0.598123184	0.018559447
LOC_Os09g34340	6.417	5.499	5.311	7.697	9.667	7.696	0.598089584	0.00000553
LOC_Os01g06660	6.593	6.783	7.157	9.48	10.523	9.455	0.597854747	4.06E-09

LOC_Os03g16020	5.399	2.861	4.11	5.693	8.32	6.22	0.597683672	0.007360227
LOC_Os02g51750	94.827	100.608	98.825	141.154	132.942	141.374	0.597593593	6.93E-35
LOC_Os08g05510	57.688	60.299	65.631	89.426	82.098	88.261	0.59754327	2.07E-22
LOC_Os05g48310	118.522	122.654	112.827	68.555	70.285	77.228	-0.59729118	1.34E-23
LOC_Os05g24650	11.966	12.216	12.592	7.259	7.092	7.645	-0.597212423	0.000000757
LOC_Os01g65900	34.692	32.552	37.082	49.025	48.947	49.646	0.59718988	3.49E-25
LOC_Os03g48600	5.895	6.085	5.809	3.277	3.225	3.878	-0.597181209	0.00000529
LOC_Os01g12260	17.973	20.191	17.321	11.813	10.477	11.122	-0.597088414	1.14E-08
MSTRG.1231	1.236	1.119	1.528	1.919	2.471	2.133	0.596839265	0.009871771
LOC_Os04g35600	2.333	2.527	2.595	1.44	1.49	1.262	-0.596757151	0.002108234
LOC_Os05g49900	1.149	2.379	1.545	1.872	2.897	3.133	0.596180259	0.003176782
LOC_Os03g49550	1.983	2.548	1.847	2.989	2.879	3.745	0.595974271	0.000947704
LOC_Os07g46510	1.433	1.358	0.628	0.361	0.305	0.562	-0.595913655	0.017912948
LOC_Os03g09900	59.778	61.829	61.434	35.707	38.858	37.5	-0.595528154	1.68E-26
LOC_Os06g13180	7.594	7.799	7.906	4.808	4.293	4.709	-0.595527071	0.0000166
LOC_Os09g15790	3.94	4.62	3.778	6.452	6.61	5.086	0.595452468	0.0000784
LOC_Os02g34590	4.732	5.38	5.979	7.7	7.378	8.045	0.595417458	6.85E-08
LOC_Os03g18500	18.452	16.845	16.754	9.555	9.379	12.068	-0.595312178	0.00000029
LOC_Os01g67950	15.8	16.562	19.353	27.602	21.594	25.144	0.595221593	0.000000123
LOC_Os02g11720	11.181	11.855	12.408	14.568	17.429	18.829	0.595182446	2.85E-08
LOC_Os06g46730	3.097	1.984	3.328	3.833	4.528	4.233	0.595107087	0.00047536
LOC_Os05g07070	42.939	41.861	44.348	59.397	61.106	56.71	0.595105888	7.29E-37
LOC_Os05g47780	21.091	22.974	24.386	30.97	33.834	32.066	0.595056188	5.58E-21
LOC_Os01g28840	1.524	1.559	0.893	0.651	0.733	0.698	-0.594661184	0.008446955
LOC_Os05g07890	9.045	8.517	8.499	4.471	6.067	4.517	-0.594392674	0.000598077
LOC_Os03g04240	7.769	7.188	7.018	4.684	3.966	4.122	-0.594344846	0.000307036
LOC_Os07g34650	2.572	3.777	2.905	4.595	4.743	4.373	0.593972183	0.000177779
LOC_Os01g51634	4.126	4.694	3.167	6.133	5.978	5.403	0.59384234	0.0000479
LOC_Os08g14760	3.892	3.503	2.84	5.262	4.556	5.117	0.593607496	0.0000465
LOC_Os04g39980	15.622	14.934	13.671	20.75	21.665	20.332	0.593326679	8.3E-13
LOC_Os10g33104	0.904	0.749	0.964	1.458	1.398	1.155	0.593262922	0.001897412
LOC_Os03g49440	12.474	12.844	10.771	17.057	17.123	17.322	0.593035988	4.01E-08
LOC_Os02g02890	624.428	635.753	593.722	367.346	377.104	391.779	-0.592780045	1.91E-54
LOC_Os08g34550	10.237	10.019	9.271	5.815	6.016	6.02	-0.592685808	9.62E-09
LOC_Os01g54340	13.469	12.686	14.327	18.103	19.159	20.54	0.592667171	4.92E-09

LOC_Os08g34170	27.398	27.438	30.455	40.287	41.411	38.727	0.592665381	7.24E-23
LOC_Os06g44300	74.562	74.652	73.963	106.526	102.027	105.237	0.592634905	5.4E-44
LOC_Os07g40220	1.605	2.965	3.745	4.01	4.421	5.002	0.592574678	0.006744065
LOC_Os06g47620	35.318	32.873	35.108	50.103	46.572	48.895	0.591961176	2.73E-24
LOC_Os02g11960	0.262	0.44	0.333	0.739	0.513	0.6	0.591865988	0.01515143
LOC_Os07g02130	7.469	7.482	9.016	3.843	5.234	5.09	-0.591786668	0.0000645
LOC_Os06g27980	0.558	0.518	0.767	0.168	0.273	0.252	-0.591327912	0.018707048
LOC_Os02g30955	0.766	0.759	0.983	1.404	1.573	2.004	0.591326243	0.018602503
LOC_Os02g41954	8.438	9.139	10.882	12.513	12.954	15.429	0.591318324	0.000000341
LOC_Os08g44460	38.265	47.115	45.778	24.367	27.794	27.279	-0.591017969	3.66E-08
LOC_Os01g14090	3.949	4.226	3.534	1.89	2.78	2.108	-0.590914898	0.000605331
LOC_Os02g11870	5.16	4.445	5.039	6.817	7.732	7.081	0.590912765	0.000208096
LOC_Os05g03540	27.412	27.95	26.708	17.571	14.692	17.422	-0.590309659	6.73E-09
LOC_Os12g09580	27.946	30.049	27.488	17.431	17.616	17.31	-0.590301904	1.12E-17
LOC_Os12g20390	56.893	51.357	60.055	86.757	76.177	76.209	0.59016645	2.53E-19
LOC_Os02g42690	2.812	3.906	3.463	4.699	4.166	6.785	0.590128577	0.0000563
LOC_Os02g35660	0.648	1.061	0.724	0.326	0.303	0.483	-0.590082606	0.017262913
LOC_Os03g21664	11.108	9.584	9.885	13.859	13.878	15.082	0.590016213	0.0000001
LOC_Os01g54520	11.6	10.579	12.034	6.71	7.357	6.507	-0.590006519	0.00000114
LOC_Os07g47230	0.346	0.172	0.238	0.418	0.762	0.469	0.589894213	0.018975621
LOC_Os05g04170	21.753	20.909	22.017	30.052	31.732	29.429	0.589593077	5.65E-21
LOC_Os11g14190	5.462	5.824	5.191	2.784	3.108	3.697	-0.589581245	0.000633934
LOC_Os09g32200	20.584	23.297	20.384	12.367	14.182	12.585	-0.589549611	1.5E-10
LOC_Os02g37690	5.111	3.993	4.231	2.592	2.684	2.555	-0.589471259	0.000166582
LOC_Os02g45420	0.238	0.504	0.311	0.655	0.929	0.582	0.589374636	0.018944561
LOC_Os02g12440	40.619	38.638	42.002	57.888	59.062	53.505	0.589152802	2.9E-30
LOC_Os02g43970	12.267	9.803	10.796	6.09	6.435	7.107	-0.58910541	0.00000773
LOC_Os04g51040	6.357	5.938	6.763	9.293	9.823	8.141	0.588962951	4.11E-08
LOC_Os05g36260	24.89	23.31	22.215	14.28	16.051	12.53	-0.58885328	1.08E-09
LOC_Os05g06840	3.795	3.44	3.529	1.87	1.921	2.476	-0.588667488	0.000355421
MSTRG.12659	29.35	31.016	32.258	45.89	42.549	41.999	0.588254824	4.52E-20
ChrSy.fgenesh.gene.82	8.873	11.168	10.373	11.637	14.674	17.721	0.58819573	0.0000141
LOC_Os01g02300	9.182	8.019	7.811	12.876	11.246	11.397	0.588089643	1.57E-09
LOC_Os12g26060	1.78	2.092	1.632	0.999	1.012	0.859	-0.58796838	0.009966406
LOC_Os07g44250	1.047	1.232	1.32	0.695	0.32	0.354	-0.587809159	0.01882801

LOC_Os01g61510	2.461	2.536	2.377	1.298	1.187	1.628	-0.587535753	0.003092196
LOC_Os03g60580	8.506	7.151	5.554	11.065	10.334	10.81	0.587173627	0.002005275
LOC_Os02g55600	9.896	9.577	8.511	13.174	13.203	14.184	0.587027164	0.0000297
LOC_Os11g03910	8.355	8.087	9.818	13.108	13.602	10.989	0.586638729	0.000000331
LOC_Os08g38040	0.039	0.059	0.199	0.228	0.231	0.391	0.586395884	0.01559985
LOC_Os09g20500	1.964	2.186	2.161	1.545	1.034	0.899	-0.586323004	0.004825067
LOC_Os11g02010	9.769	10.936	10.335	15.95	15.294	13.174	0.586173667	0.000000311
LOC_Os03g03810	32.77	35.066	36.055	48.443	51.27	47.561	0.58592909	1.65E-10
LOC_Os01g34890	1.017	0.944	1.063	1.594	1.336	1.605	0.585872569	0.001040168
LOC_Os10g40824	7.59	7.199	7.006	4.3	4.521	4.282	-0.585703244	0.00000228
LOC_Os02g15070	0.408	0.438	0.81	0.951	1.562	0.644	0.585576807	0.017798991
LOC_Os01g07560	9.382	9.325	7.937	5.12	5.048	6.042	-0.58553555	1.22E-09
LOC_Os06g11980	3.96	4.262	3.694	2.503	1.669	2.279	-0.585463388	0.00637841
LOC_Os07g32420	54.111	52.728	59.677	80.249	76.63	76.564	0.58545405	4.87E-28
LOC_Os11g31680	0.609	0.54	0.605	0.93	0.98	0.789	0.585392466	0.002903762
LOC_Os01g06150	1.178	1.389	0.668	2.056	2.468	1.265	0.585358457	0.015672404
LOC_Os11g02020	4.888	5.115	4.984	3.041	2.635	3.008	-0.58528188	0.000713887
LOC_Os12g40860	5.234	4.473	4.748	2.779	2.491	3.272	-0.585117795	0.0000967
LOC_Os06g48600	11.034	9.872	10.57	6.593	6.119	5.718	-0.585066108	0.000358383
LOC_Os03g38000	290.325	288.72	279.484	169.134	178.179	183.286	-0.584938041	2.33E-46
LOC_Os11g29380	20.513	20.433	19.324	11.734	12.017	13.262	-0.584892758	7.95E-17
LOC_Os06g11730	3.087	3.757	3.169	4.467	5.512	4.69	0.58462112	0.000157101
LOC_Os12g42830	0.641	0.876	0.615	0.324	0.239	0.358	-0.584334831	0.019046789
LOC_Os05g10690	1.197	1.032	1.209	0.536	0.628	0.736	-0.584273514	0.004623568
LOC_Os03g06000	1.918	2.435	2.418	0.752	0.879	1.676	-0.584245041	0.014940111
LOC_Os01g46910	0.154	0.381	0.493	0.482	0.789	1.005	0.583847916	0.019290394
LOC_Os04g02850	1.156	1.336	0.994	0.488	0.801	0.543	-0.583507863	0.009896817
LOC_Os10g40720	313.967	318.037	298.34	423.318	424.679	451.314	0.583489465	4.58E-49
LOC_Os08g31660	7.851	7.081	7.968	3.884	2.854	5.659	-0.583382224	0.006641302
LOC_Os03g51260	4.862	5.197	5.932	2.844	3.594	2.969	-0.583362225	0.000313393
LOC_Os10g11889	2.476	1.582	4.023	3.538	6.233	4.458	0.583283889	0.014574555
LOC_Os12g43830	11.833	10.07	9.679	6.158	5.569	7.085	-0.583107535	0.0000316
LOC_Os02g28180	0.197	0.391	0.29	0.762	0.579	0.641	0.583036952	0.019250708
LOC_Os03g42130	10.474	11.867	11.02	6.046	6.758	7.279	-0.583026065	0.00000343
LOC_Os01g48000	7.355	7.349	6.591	10.432	9.637	10.016	0.582775637	4.89E-10

LOC_Os01g52490	120.339	114.418	113.801	72.209	72.332	70.672	-0.582709683	7.37E-25
LOC_Os07g01070	0.091	0.361	0.167	0.498	0.624	0.384	0.582635153	0.018127356
LOC_Os03g07226	1.96	1.651	1.416	2.656	3.012	2.211	0.582562751	0.003898561
LOC_Os04g56790	3.72	3.765	2.631	1.718	2.183	1.965	-0.582524136	0.000784496
LOC_Os02g13150	12.233	11.356	11.279	7.742	6.855	6.544	-0.582412517	0.000000294
LOC_Os05g48650	77.734	80.587	79.407	117.234	96.256	120.153	0.582271067	1.3E-15
LOC_Os09g35790	4.017	4.899	3.844	6.708	5.689	6.615	0.582070683	0.0000289
LOC_Os06g50950	23.539	25.319	24.455	15.007	14.372	15.578	-0.582018383	3.82E-12
LOC_Os12g10320	1.066	1.793	0.741	0.623	0.411	0.67	-0.581959798	0.016527864
LOC_Os06g32980	1.146	1.646	1.944	0.553	0.672	0.93	-0.581843604	0.018327676
LOC_Os09g30486	11.986	10.973	11.408	5.848	5.704	8.878	-0.581826483	0.0000518
LOC_Os09g07780	1.311	1.136	1.218	1.054	0.32	0.542	-0.581777514	0.010191169
LOC_Os07g23340	5.189	7.363	6.016	8.623	9.558	8.484	0.581605223	0.00000564
LOC_Os04g45920	2.64	3.342	3.124	4.502	4.615	4.12	0.581483686	0.0000359
LOC_Os03g56250	17.978	18.142	17.322	26.066	24.449	24.331	0.581325802	1.81E-19
LOC_Os06g41030	0.986	0.958	1.134	0.69	0.416	0.54	-0.581324228	0.008329173
LOC_Os05g15690	0.093	0.924	0.257	0.675	1.505	0.925	0.581268386	0.017506924
LOC_Os02g42650	4.773	4.048	3.466	2.136	2.203	2.721	-0.58106103	0.001288651
LOC_Os01g17390	33.087	31.589	31.463	44.702	45.598	44.052	0.581058017	1.84E-27
LOC_Os03g63850	8.482	12.258	12.215	15.168	15.172	16.755	0.581018305	0.00000115
LOC_Os05g16670	17.208	16.98	17.992	24.522	25.18	23.367	0.58092047	8.05E-22
LOC_Os09g30438	24.768	22.296	23.244	14.023	13.919	14.988	-0.580829131	1.58E-11
LOC_Os03g05750	30.331	31.683	32.85	42.972	40.667	49.483	0.580618015	1.03E-14
LOC_Os07g48730	13.55	14.133	14.095	19.797	19.017	20.023	0.58052427	1.76E-11
LOC_Os05g19000	2.474	1.765	1.935	3.118	3.064	3.064	0.58023792	0.001329744
LOC_Os01g49529	22.353	20.629	24.928	31.472	29.688	30.468	0.580196024	1.25E-18
LOC_Os03g57240	3.914	3.624	3.689	2.223	2.432	1.848	-0.580174845	0.001136256
LOC_Os01g07360	25.166	25.212	25.165	15.072	15.558	16.339	-0.580158169	1.14E-15
LOC_Os06g48720	8.359	7.622	6.779	11.566	11.072	9.762	0.580148327	0.000000472
LOC_Os05g02410	18.837	18.738	18.574	11.322	11.768	10.854	-0.580095416	0.00000269
LOC_Os06g30750	53.699	55.8	55.046	79.106	78.138	72.772	0.580062681	2.55E-25
LOC_Os05g35110	42.602	39.319	44.007	59.015	55.891	61.17	0.580008758	3.5E-22
LOC_Os04g40030	4.764	3.833	4.63	6.876	5.814	6.461	0.579948179	0.0000545
LOC_Os08g43440	14.156	12.426	12.397	8.455	7.33	7.983	-0.579924412	1.44E-08
LOC_Os04g43990	6.732	6.671	6.308	8.534	8.265	11.745	0.579683828	0.0000811

LOC_Os01g04660	5.159	4.559	5.64	7.768	7.559	6.821	0.579521115	0.0000198
LOC_Os09g38320	3.424	3.894	3.166	1.924	1.892	2.282	-0.579313554	0.000713897
LOC_Os07g04840	8.853	7.335	9.166	5.154	5.97	3.51	-0.579220692	0.00156864
LOC_Os01g73000	5.898	7.387	7.876	9.175	11.306	9.977	0.579160889	0.000000486
LOC_Os09g36110	1.516	0.901	0.904	0.512	0.519	0.661	-0.579153831	0.012711291
LOC_Os12g03880	57.365	57.217	57.946	35.03	35.554	35.98	-0.579134593	1.74E-20
LOC_Os12g09540	7.679	6.975	6.984	9.896	11.008	9.798	0.579040417	8.05E-08
LOC_Os04g46650	1.333	2.133	1.974	0.593	1.053	1.017	-0.57885962	0.015719935
LOC_Os07g34690	1.246	0.988	1.257	2.044	1.523	2.529	0.578530346	0.015368972
LOC_Os04g40680	1.117	1.373	1.201	0.786	0.48	0.584	-0.578482019	0.01205971
LOC_Os12g36210	722.439	714.472	673.731	420.125	465.069	424.953	-0.578448567	8.83E-40
LOC_Os03g57840	11.664	10.903	12.76	16.935	16.585	16.151	0.577996548	6.61E-11
LOC_Os01g66940	106.343	106.624	102.339	147.888	149.961	141.076	0.577991567	6.71E-43
LOC_Os07g07320	44.956	42.755	39.564	27.481	25.703	25.131	-0.577962316	1.96E-12
LOC_Os01g64520	266.391	266.952	265.351	365.117	383.202	362.825	0.577781703	9.63E-65
LOC_Os03g06410	30.303	31.385	31.257	44.156	45.25	40.213	0.577620981	1.48E-28
LOC_Os05g46520	13.845	13.16	12.04	7.927	8.145	7.715	-0.577373233	0.00000015
LOC_Os01g10530	20.061	19.967	20.493	27.989	29.712	27.363	0.577316221	1.29E-21
LOC_Os12g40730	3.11	2.232	3.671	4.691	3.391	5.417	0.577258796	0.001485757
LOC_Os05g09400	8.825	7.52	8.847	4.942	5.404	4.899	-0.577245915	0.000117204
LOC_Os12g02980	0.717	0.982	0.783	0.428	0.367	0.33	-0.577202194	0.019234567
LOC_Os08g17784	0.614	0.755	0.558	0.294	0.341	0.247	-0.577104165	0.016525438
LOC_Os11g18570	48.335	52.375	45.641	30.684	29.972	29.902	-0.577098444	2.01E-19
LOC_Os03g44430	7.979	7.234	8.305	5.29	4.941	3.973	-0.5770982	0.0000107
LOC_Os01g04300	0.612	0.866	0.909	0.197	0.171	0.631	-0.577048986	0.019623059
LOC_Os03g50120	14.743	15.227	15.388	8.826	9.397	9.587	-0.576987043	1.77E-09
LOC_Os05g36994	12.086	12.141	13.605	17.198	18.613	17.471	0.576866529	1.34E-10
LOC_Os07g36490	11.267	11.453	13.684	6.917	7.798	6.994	-0.576574536	0.000124355
LOC_Os11g01340	62.699	61.069	55.693	37.182	38.143	35.838	-0.576456542	1.98E-22
LOC_Os04g08060	4.469	4.428	4.745	1.543	3.174	3.012	-0.576305115	0.003092739
LOC_Os01g11700	1.015	1.028	1.456	0.827	0.435	0.421	-0.576246673	0.017461598
LOC_Os08g05780	13.719	14.155	14.233	19.404	18.256	21.815	0.576002442	3.59E-08
LOC_Os04g45940	67.779	67.624	70.327	98.31	94.666	93.66	0.575931231	4.52E-26
LOC_Os03g37930	0.222	0.198	0.204	0.365	0.5	0.421	0.575794748	0.02004574
LOC_Os09g24970	34.693	33.138	33.828	46.845	45.618	49.217	0.575686757	1.17E-21

LOC_Os07g41250	15.506	17.972	18.195	24.66	23.24	24.659	0.57561182	2.46E-15
LOC_Os10g40270	1.934	1.597	1.625	0.505	1.34	0.763	-0.575384183	0.01415296
LOC_Os10g42980	0.269	0.183	0.612	1.092	0.48	1.314	0.575271483	0.016576802
LOC_Os03g55090	14.6	15.115	14.043	8.345	9.738	8.908	-0.575209693	2.89E-12
LOC_Os07g02330	3.039	2.179	2.713	4.051	3.675	4.075	0.574822058	0.00120892
LOC_Os08g31930	1.68	2.164	1.617	1.026	0.849	1.136	-0.574571402	0.005722207
LOC_Os05g49070	1.458	1.163	1.633	0.742	0.831	0.548	-0.57446525	0.01540088
LOC_Os05g03120	11.31	11.365	10.843	6.669	7.206	6.673	-0.574377342	2.91E-09
LOC_Os01g50930	13.183	13.119	13.668	6.913	8.409	8.73	-0.574375734	0.00000399
LOC_Os01g51810	1.333	1.678	1.553	0.964	0.814	0.701	-0.574366204	0.007794429
LOC_Os03g21850	14.986	14.113	14.977	19.504	21.548	20.137	0.574233023	6.77E-20
LOC_Os03g13640	2.074	2.447	1.359	0.667	0.966	0.713	-0.574176779	0.019844809
LOC_Os02g47605	2.047	1.767	2.061	2.887	2.653	2.909	0.574090805	0.0000372
LOC_Os01g59200	1.573	0.944	1.573	0.598	0.326	0.903	-0.574069475	0.019262655
LOC_Os08g36910	373.615	356.899	411.036	534.693	489.286	592.497	0.573893881	1.11E-08
LOC_Os11g10100	20.135	20.189	20.109	28.254	28.692	27.547	0.573658974	4.64E-19
LOC_Os02g09810	31.124	31.672	32.166	45.177	44.511	42.542	0.573598947	3.84E-22
LOC_Os03g02460	15.047	14.633	14.94	20.718	20.657	21.093	0.573585704	3.03E-12
LOC_Os01g43490	35.507	33.956	36.723	50.301	47.709	49.771	0.57333391	7.18E-23
LOC_Os04g27750	4.044	5.856	6.37	2.701	3.042	2.993	-0.57330987	0.009222876
LOC_Os12g31620	0.327	0.36	0.666	0.911	0.675	0.704	0.57295202	0.013402386
LOC_Os11g41100	0.95	0.75	0.855	0.764	1.841	1.428	0.572830125	0.006898169
LOC_Os01g64230	0.567	1.733	1.127	2.189	1.561	2.125	0.572557199	0.014982505
LOC_Os01g19130	19.785	23.607	23.41	31.421	32.154	29.814	0.572437671	1.72E-13
LOC_Os06g15760	23.902	24.913	24.008	33.765	36.033	31.697	0.572409815	5.87E-18
LOC_Os05g50190	48.332	47.856	48.178	70.488	67.199	62.974	0.572386578	7.54E-25
LOC_Os06g46900	10.237	11.263	9.349	14.374	14.515	14.154	0.572378456	7.97E-08
LOC_Os03g62480	7.224	7.416	6.087	3.673	4.137	4.636	-0.572194089	0.000039
LOC_Os01g50910	454.184	459.567	434.043	247.986	255.719	319.955	-0.572181529	0.000000747
LOC_Os10g28350	103.328	112.929	107.453	144.776	156.793	148.023	0.572114862	1.35E-28
LOC_Os01g16910	30.318	30.164	30.861	18.25	18.825	19.435	-0.572010667	3.11E-13
LOC_Os05g05690	2.348	2.882	2.795	1.251	1.611	1.675	-0.571852465	0.003616778
LOC_Os01g06560	108.723	108.414	114.951	156.706	147.573	156.128	0.571800197	2.55E-36
LOC_Os02g01355	2.719	2.977	3.017	4.901	3.778	4.775	0.571796591	0.004918948
LOC_Os11g10480	682.792	674.684	687.787	976.12	951.829	904.013	0.571700338	3.01E-59

LOC_Os03g02550	5.65	6.031	5.057	7.722	7.728	8.341	0.571439085	0.0000051
MSTRG.3763	2.579	3.27	2.553	1.493	1.109	1.302	-0.571102419	0.018730903
LOC_Os07g07709	142.182	139.947	145.106	87.358	91.213	87.627	-0.571093804	3.89E-27
LOC_Os05g23720	93.178	95.937	90.301	54.519	58.683	60.895	-0.570927072	3.93E-30
LOC_Os04g25740	3.209	2.779	3.378	4.242	4.881	4.331	0.570913045	0.000036
LOC_Os10g16974	20.23	20.453	20.035	13.338	13.116	11.106	-0.570883709	9.57E-12
LOC_Os03g42520	3.26	3.29	3.681	4.63	4.251	5.935	0.570691536	0.00018964
LOC_Os01g32770	109.464	104.212	104.754	147.286	137.47	156.708	0.570593784	3.55E-28
LOC_Os05g18660	27.576	27.775	29.381	39.374	38.482	39.913	0.570347171	2.37E-20
LOC_Os10g26540	33.91	29.523	32.698	44.225	43.244	46.817	0.57016546	7.54E-12
LOC_Os12g39320	10.981	12.235	12.308	17.167	16.217	16.211	0.570134687	5.55E-13
LOC_Os01g60790	184.498	184.764	180.993	114.225	112.171	116.763	-0.570082181	1.35E-32
LOC_Os10g15310	28.401	27.988	27.063	37.789	39.297	39.647	0.569934696	2.12E-20
LOC_Os10g21090	0.873	0.865	0.948	0.576	0.449	0.352	-0.569895219	0.013867454
LOC_Os01g25484	21.507	21.134	22.149	30.078	28.625	31.619	0.569850425	1.69E-19
LOC_Os01g56050	5.698	7.077	5.555	8.787	9.573	7.853	0.569679252	0.0000238
LOC_Os11g07450	10.275	9.775	11.711	14.784	14.577	15.258	0.569538221	5.99E-09
LOC_Os05g08350	1.883	1.221	1.413	2.973	2.511	1.606	0.569520877	0.006793326
LOC_Os12g37400	13.62	13.711	12.761	8.536	7.729	8.491	-0.569512027	5.9E-11
LOC_Os10g37760	13.443	14.083	12.398	7.653	9.706	7.059	-0.569188168	0.000004
LOC_Os12g13950	6.404	6.243	5.235	3.647	3.722	3.456	-0.569162507	0.0000157
LOC_Os02g15340	9.445	9.248	7.296	12.197	12.181	12.275	0.568955098	0.000000569
LOC_Os05g10730	9.306	9.41	9.444	13.199	12.418	13.497	0.568832347	2.76E-20
LOC_Os06g12310	12.818	10.79	12.273	8.171	6.766	6.957	-0.568778871	0.00000191
LOC_Os10g32170	1.276	1.523	1.109	0.79	0.674	0.615	-0.568735497	0.010572356
LOC_Os06g45500	17.922	17.477	17.608	23.879	24.757	24.847	0.568497789	2.55E-23
LOC_Os05g46550	29.715	28.996	28.892	17.599	18.775	18.101	-0.568369713	2.68E-16
LOC_Os01g56270	6.217	6.975	7.016	9.633	9.255	9.535	0.568191413	6.19E-08
LOC_Os08g04450	8.122	7.386	9.929	5.3	4.644	5.423	-0.56811302	0.0000428
MSTRG.1703	4.895	4.401	3.782	2.799	2.216	2.535	-0.568010924	0.001949728
LOC_Os06g51460	0.903	1.209	0.95	1.405	1.699	1.462	0.56779775	0.001798565
MSTRG.5998	2.451	2.607	1.751	3.581	3.066	3.478	0.567769406	0.001872562
LOC_Os12g11830	4.36	5.199	5.624	6.345	6.865	9.398	0.567763259	0.001757979
LOC_Os03g40780	8.518	7.413	7.393	4.555	4.386	5.389	-0.567574341	6.46E-08
LOC_Os06g16050	38.354	38.699	40.88	24.957	25.468	23.226	-0.567570006	6.36E-24

LOC_Os02g35180	9.127	9.078	9.348	6.546	5.153	5.063	-0.567490022	0.00000785
LOC_Os05g48050	9.746	7.879	9.736	5.286	5.5	5.685	-0.567459137	0.0000749
LOC_Os03g61470	16.238	17.494	16.07	24.618	22.81	23.712	0.567417212	0.0000318
LOC_Os06g51524	9.86	11.502	9.842	14.76	15.388	13.886	0.567297753	0.000000614
LOC_Os08g11470	7.592	7.269	7.898	11.074	10.993	9.744	0.567101765	1.02E-10
LOC_Os03g58540	2.84	2.198	2.366	1.303	1.499	1.398	-0.5670774	0.003059851
LOC_Os10g25550	23.274	27.734	25.232	33.641	37.328	35.138	0.566886898	1.14E-13
LOC_Os03g13300	74.382	66.781	68.622	97.443	92.073	100.579	0.566774713	1.24E-27
LOC_Os02g49650	1.812	1.662	2	2.751	2.723	2.723	0.566770655	0.002185737
MSTRG.24290	0.993	0.733	0.867	1.325	1.814	1.29	0.56659582	0.015253491
LOC_Os05g45480	3.373	4.379	3.306	1.29	2.502	2.255	-0.566156806	0.007997622
LOC_Os05g41110	240.322	235.06	242.98	144.63	156.139	148.913	-0.565994313	1.6E-33
LOC_Os11g38610	2.912	2.786	2.38	1.605	1.578	1.651	-0.565917975	0.000163606
LOC_Os10g27040	1.142	0.963	1.174	1.742	1.63	1.461	0.565896645	0.000999757
LOC_Os02g55870	1.693	2.289	2.685	1.218	1.603	0.945	-0.565880581	0.005017539
LOC_Os05g30530	86.47	92.174	86.489	54.39	55.912	55.318	-0.565840162	7.16E-24
LOC_Os05g39950	2.615	2.656	1.948	3.627	3.452	3.508	0.565653529	0.000947976
LOC_Os02g57400	42.175	43.084	46.923	60.591	61.302	60.939	0.565555089	8.2E-31
LOC_Os05g47750	6.072	5.058	6.372	7.888	7.894	9.271	0.565527268	0.0000437
LOC_Os01g41010	5.742	6.303	7.011	9.081	8.996	8.962	0.565158275	0.00000765
LOC_Os09g24690	168.941	176.483	171.425	105.384	116.358	101.579	-0.565125157	2E-23
LOC_Os01g64790	21.92	19.67	21.568	29.089	27.742	30.924	0.565092278	2.82E-13
LOC_Os03g60509	51.775	56.28	54.3	32.342	36.794	32.015	-0.564966713	3.4E-13
LOC_Os09g39500	194.389	200.174	205.456	119.175	134.18	122.325	-0.564922119	2.29E-27
LOC_Os05g32474	3.532	4.864	4.173	6.922	4.796	6.549	0.564911758	0.000425261
LOC_Os08g26870	29.277	29.915	29.156	42.41	41.63	38.407	0.564859079	5.02E-18
LOC_Os01g19260	8.725	7.775	8.083	10.639	11.909	11.913	0.564857897	3.94E-08
LOC_Os01g46380	364.81	358.263	402.984	535.717	493.503	524.888	0.564790465	8.55E-34
LOC_Os11g01180	8.305	10.032	10.03	13.745	14.004	12.572	0.564785948	0.00000854
LOC_Os06g50920	3.376	2.628	2.533	4.015	4.49	3.797	0.564783465	0.000110817
LOC_Os05g48220	136.234	135.015	129.108	77.439	83.198	89.27	-0.564623816	1.93E-21
LOC_Os11g40950	0.76	0.581	0.335	0.943	0.876	0.869	0.56452381	0.010351559
LOC_Os02g42520	18.777	16.981	18.986	10.973	12.348	10.606	-0.564239606	1.68E-09
LOC_Os07g47300	3.149	3.553	3.348	1.751	2.352	1.913	-0.563954892	0.000267535
LOC_Os09g19910	11.391	12.762	13.352	7.992	7.595	7.122	-0.563800391	0.000054

LOC_Os06g36390	45.055	44.341	50.269	65.131	59.23	69.044	0.563755328	5.9E-17
LOC_Os05g50380	70.403	70.871	76.648	46.902	44.353	44.73	-0.563634634	1.09E-26
LOC_Os01g08780	6.113	6.254	6.428	3.923	4.123	3.443	-0.563602519	0.00000658
LOC_Os01g73234	8.799	8.898	7.915	4.705	4.738	5.642	-0.563582795	0.000741781
LOC_Os01g17010	3.999	3.962	3.797	5.666	5.05	5.765	0.563553088	0.000000127
LOC_Os06g14740	32.17	31.452	32.514	43.932	45.361	43.239	0.563337041	4.26E-39
LOC_Os03g25340	3.034	3.092	3.815	5.101	5.679	3.909	0.563234882	0.001198711
LOC_Os12g12690	50.357	49.737	53.799	70.174	71.615	70.675	0.563143301	3.7E-28
LOC_Os05g31750	62.869	63.404	59.386	38.49	37.703	39.975	-0.562981928	2.2E-24
LOC_Os03g49490	14.15	14.916	16.023	21.58	20.46	20.884	0.562929999	4.28E-09
LOC_Os03g10620	67.998	65.648	64.88	94.04	90.422	89.225	0.562775885	1.82E-30
LOC_Os06g48520	1.541	1.191	1.94	2.243	2.375	2.379	0.562539114	0.002446371
LOC_Os01g61880	10.442	11.532	8.667	6.328	6.216	5.967	-0.562342781	0.000000328
LOC_Os01g57930	4.764	3.655	4.663	2.73	2.031	2.907	-0.562266068	0.001223364
LOC_Os05g40420	100.532	96.999	92.719	59.063	59.237	63.367	-0.562197504	1.4E-30
LOC_Os08g09520	22.444	23.465	23.686	31.824	33.656	30.732	0.561921693	8.35E-21
LOC_Os01g74600	2.336	2.723	2.52	1.503	1.433	1.529	-0.561907442	0.001006545
LOC_Os02g49720	301.037	301.085	321.901	423.158	419.78	427.272	0.561832379	7.45E-56
LOC_Os05g36290	37.951	38.623	41.448	60.643	56.95	46.398	0.561764399	2.45E-14
LOC_Os04g33150	23.63	24.08	23.352	31.968	32.095	34.415	0.561718029	2.57E-13
LOC_Os03g55290	29.919	33.552	31.283	42.676	44.513	44.69	0.561694575	3.09E-10
LOC_Os03g02040	8.141	6.687	7.219	9.973	10.946	10.434	0.561354847	0.0000311
LOC_Os03g42464	15.49	16.535	16.219	21.869	24.516	20.334	0.560886712	6.46E-18
LOC_Os01g02830	0.789	0.784	0.794	1.575	1.137	1.049	0.56080659	0.009319856
LOC_Os08g33460	80.426	83.377	74.855	47.668	50.472	50.94	-0.560561696	1.73E-15
LOC_Os05g26820	11.837	12.687	12.653	7.951	7.896	7.208	-0.560300515	1.02E-08
LOC_Os04g46250	9.73	9.719	10.988	12.821	13.498	16.41	0.560138552	0.000000742
LOC_Os04g58310	1.156	1.002	1.237	0.511	0.707	0.261	-0.55993009	0.020797979
MSTRG.13664	23.492	24.718	26.203	35.544	32.159	35.182	0.55955576	2.76E-15
LOC_Os11g46080	0.804	0.896	0.875	0.291	0.417	0.566	-0.559266343	0.017350378
LOC_Os02g48060	12.871	14.161	13.296	7.62	7.764	9.385	-0.559156003	0.00000152
LOC_Os03g28040	1.808	1.024	1.816	0.249	0.758	0.699	-0.55910526	0.019606195
LOC_Os09g13940	6.183	5.806	6.046	4.092	3.795	3.325	-0.558719227	0.0000303
LOC_Os06g42754	6.089	5.607	5.78	9.606	9.736	6.331	0.558678052	0.001206144
LOC_Os12g22970	0.43	0.923	0.788	1.037	1.721	1.615	0.558477437	0.021201016

LOC_Os09g33710	3.429	2.822	3.251	4.766	4.547	4.298	0.558421277	0.000146908
LOC_Os03g48540	46.863	44.867	46.608	67.534	61.072	61.655	0.558392294	7.33E-27
LOC_Os04g47970	57.974	56.404	60.663	82.754	81.354	76.983	0.558322612	1.15E-25
LOC_Os02g44770	3.868	3.637	3.352	5.287	4.776	5.168	0.558321704	0.00000104
LOC_Os07g14610	1.728	2.903	2.549	2.827	4.408	4.269	0.558295545	0.01067651
LOC_Os02g26349	8.441	9.312	8.643	4.5	5.129	6.448	-0.558103577	0.0000588
LOC_Os08g44680	7.196	6.842	5.747	4.591	3.064	3.925	-0.558046469	0.001564751
LOC_Os11g39190	12.086	11.168	11.461	15.801	16.582	15.362	0.55788576	1.02E-15
LOC_Os07g04020	1.55	1.483	2.01	0.67	1.15	0.627	-0.557872369	0.018825815
LOC_Os11g03290	0.732	0.621	0.926	0.403	0.17	0.377	-0.557600368	0.021151361
LOC_Os04g56930	3.114	2.283	2.407	3.605	3.095	4.69	0.557351527	0.001408709
LOC_Os07g08320	1.046	1.214	1.288	0.461	0.731	0.647	-0.557115018	0.014781055
LOC_Os11g07470	42.959	42.805	42.211	26.695	26.318	27.539	-0.557112276	2.95E-34
LOC_Os07g45074	6.113	6.499	6.065	8.335	8.83	8.764	0.556908573	1.38E-09
LOC_Os07g41260	25.306	24.724	25.901	15.784	15.509	16.269	-0.556770941	3.95E-14
LOC_Os04g38400	4.538	5.101	4.361	2.736	1.963	3.424	-0.55642022	0.002335156
LOC_Os03g39740	8.165	8.764	8.725	4.978	5.406	5.577	-0.556393854	1.96E-09
LOC_Os04g29000	27.412	28.625	27.478	16.191	17.818	18.164	-0.556337999	7.67E-12
LOC_Os03g19930	24.944	24.813	23.41	15.221	15.535	15.099	-0.556266997	1.96E-14
LOC_Os04g37480	13.187	10.6	11.69	7.583	7.686	6.498	-0.556266935	0.0000104
LOC_Os02g52460	7.117	6.528	7.099	3.711	4.091	4.641	-0.555379303	0.000368686
LOC_Os01g12464	10.039	9.641	11.045	14.751	13.366	15.462	0.554989382	0.0000507
LOC_Os01g07980	4.9	4.718	6.074	7.589	8.95	6.473	0.55489473	0.001291501
LOC_Os01g37470	1.145	0.986	0.844	0.672	0.511	0.414	-0.554836806	0.015095042
LOC_Os01g08540	3.783	3.962	3.561	1.984	2.644	2.309	-0.554808419	0.0000115
LOC_Os02g55610	42.139	37.809	40.669	55.063	54.663	56.537	0.554768193	3.47E-14
LOC_Os02g42210	9.453	11.53	11.088	6.887	6.662	6.262	-0.554653875	0.00000148
LOC_Os03g59070	18.499	17.964	17.861	24.387	24.209	26.258	0.554559866	7E-14
LOC_Os02g24020	41.294	36.883	36.033	21.194	25.54	24.731	-0.554390708	6.26E-12
LOC_Os04g51340	31.735	30.191	30.464	45.939	43.964	37.451	0.554290548	2.42E-14
LOC_Os06g34830	2.719	2.879	2.579	1.809	1.65	1.395	-0.554226336	0.00103489
LOC_Os01g61410	5.097	5.404	5.877	2.738	3.849	3.204	-0.554170918	0.000208078
LOC_Os09g38110	8.208	10.456	9.223	11.848	14.423	13.191	0.554072314	0.0000337
LOC_Os03g19330	2.548	2.89	2.844	1.71	1.48	1.565	-0.553429555	0.004278557
LOC_Os11g06900	0.453	0.199	0.507	0.752	0.787	0.59	0.553302744	0.020217746

LOC_Os05g25860	1.502	1.275	1.77	2.277	2.308	2.177	0.553119747	0.002669745
MSTRG.24800	80.163	80.303	85.665	111.044	121.202	106.704	0.552827437	3.14E-15
LOC_Os01g58080	17.494	16.862	16.536	10.934	9.559	11.157	-0.552783366	2.73E-08
LOC_Os03g60010	0.438	0.901	0.504	1.078	1.102	0.753	0.552740979	0.010426515
LOC_Os01g09830	135.794	122.054	130.617	178.03	176.052	178.169	0.552465534	2.19E-25
LOC_Os09g20650	11.001	12.081	12.04	15.232	16.642	16.794	0.552404502	3.07E-09
LOC_Os01g62630	4.357	4.179	3.931	2.253	2.692	2.483	-0.552400315	0.000846723
LOC_Os03g22330	29.302	28.147	29.178	17.091	19.757	17.59	-0.552140747	1.3E-13
LOC_Os11g07911	9.994	11.965	11.607	15.667	15.066	16.75	0.552088521	0.0000501
LOC_Os02g40330	8.614	8.014	9.42	13.498	12.038	13.374	0.55198328	7.69E-11
LOC_Os06g49770	29.353	29.013	25.619	16.418	17.861	17.521	-0.551909157	0.000000069
LOC_Os03g38970	38.28	37.355	39.005	50.513	52.502	53.72	0.551741606	1.56E-38
LOC_Os02g42910	1.776	1.853	1.513	0.933	1.141	1.033	-0.551711111	0.000242286
MSTRG.9279	9.23	9.255	9.533	13.41	13.266	12.637	0.551670992	0.00000606
LOC_Os03g10820	9.309	10.048	10.764	13.579	14.295	13.906	0.55147487	9.83E-09
LOC_Os06g03800	11.432	10.181	11.371	15.862	15.005	14.849	0.55097448	8.98E-10
LOC_Os02g12420	8.051	9.068	9.087	12.226	12.003	11.902	0.550831933	1.13E-11
LOC_Os08g29160	6.813	6.697	7.057	10.238	8.272	9.904	0.550821586	1.21E-09
LOC_Os01g43480	27.554	27.142	29.051	37.533	37.752	38.507	0.550762026	2.19E-25
LOC_Os07g37510	0.543	0.717	0.862	1.361	0.99	1.272	0.550667889	0.017349938
LOC_Os07g23244	21.053	22.078	23.207	12.734	14.156	14.508	-0.550667651	1.36E-08
LOC_Os07g01100	5.476	6.328	4.231	3.117	3.611	2.775	-0.550607334	0.001265719
LOC_Os06g11830	1.667	2.392	1.516	2.484	3.226	2.452	0.55043352	0.001478649
LOC_Os02g40410	2.441	1.879	2.548	1.286	1.275	1.507	-0.550353585	0.001189423
LOC_Os09g07614	12.063	10.418	9.523	6.535	6.497	6.342	-0.550171234	0.000221522
LOC_Os10g33520	2.409	2.218	1.871	3.336	2.383	3.783	0.550143255	0.001931983
LOC_Os01g70380	27.652	27.91	29.462	40.62	38.138	37.708	0.549802674	2.12E-20
LOC_Os04g45480	2.699	3.277	3.166	4.619	3.603	4.868	0.549631266	0.000400439
LOC_Os07g02810	54.273	54.779	53.202	74.965	68.916	77.837	0.549485052	3.12E-25
LOC_Os03g53400	3.915	1.743	2.809	4.434	3.717	4.81	0.549469992	0.007018062
LOC_Os06g50400	31.355	32.479	34.75	21.235	19.149	21.376	-0.549436152	3.67E-10
LOC_Os01g41050	0.871	1.085	0.434	1.32	1.119	1.392	0.549358927	0.012781796
LOC_Os02g03960	69.389	67.54	65.258	40.886	40.084	46.542	-0.549352444	2.78E-19
LOC_Os01g59740	53.519	51.02	48.141	29.611	32.588	34.047	-0.54909165	4.35E-17
LOC_Os05g02310	139.807	143.138	145.679	90.029	91.204	90.2	-0.549018796	2.41E-32

LOC_Os03g57660	7.165	7.609	8.587	10.999	10.293	11.063	0.548598378	7.46E-08
LOC_Os06g48780	170.724	177.407	169.777	104.855	100.463	121.25	-0.548511376	1.58E-17
LOC_Os01g13720	14.357	13.568	16.169	7.962	8.842	9.949	-0.548416719	0.000286741
LOC_Os02g45240	24.318	25.617	24.47	35.445	33.136	33.572	0.54825277	3.28E-13
LOC_Os04g33530	0.445	0.305	0.283	0.991	0.502	0.618	0.548231927	0.021795068
LOC_Os07g46560	2.561	2.175	1.945	1.553	1.002	1.155	-0.548215044	0.008731382
LOC_Os11g45710	33.613	34.142	35.888	46.248	51.574	43.974	0.548148853	1.86E-22
LOC_Os09g29310	7.266	6.108	6.964	10.16	8.324	9.995	0.547931593	0.0000118
LOC_Os02g16690	3.565	3.715	3.322	1.996	2.348	2.149	-0.547848028	0.0000749
LOC_Os08g21541	1.023	0.881	1.132	1.565	1.476	1.365	0.547591505	0.000762465
LOC_Os07g43120	1.627	1.81	2.075	2.772	2.347	2.85	0.547315642	0.00090063
LOC_Os02g08150	19.813	19.01	20.751	12.617	10.95	13.631	-0.547247175	2.69E-08
LOC_Os04g40050	16.729	17.103	14.893	10.174	11.517	9.058	-0.547223033	0.000000297
LOC_Os07g46080	0.684	0.985	0.748	0.132	0.577	0.458	-0.547197044	0.019907665
LOC_Os02g57090	2.678	2.879	3.021	4.353	3.746	3.908	0.547194519	0.00000896
LOC_Os02g49700	81.053	77.873	74.64	47.576	47.278	52.986	-0.547109479	4.64E-23
LOC_Os03g37984	41.209	41.044	45.589	59.855	56.901	57.893	0.546953276	9.88E-23
LOC_Os01g38660	6.149	7.927	6.426	10.588	10.926	7.931	0.546908855	0.000544128
LOC_Os03g17980	64.682	64.136	72.115	91.335	92.524	90.416	0.54687347	4.29E-28
LOC_Os02g36110	46.346	50.911	46.399	30.46	29.948	30.367	-0.546862877	2.85E-18
LOC_Os09g30240	5.845	5.719	4.517	2.908	3.451	3.316	-0.546777753	0.000540782
LOC_Os11g31590	8.79	9.843	8.809	5.561	5.037	6.231	-0.546737781	0.0000381
LOC_Os01g36850	2.493	2.201	1.872	2.83	3.253	3.496	0.546689654	0.001485127
LOC_Os03g32314	71.986	71.303	70.615	45.834	46.115	43.757	-0.546667914	1.04E-33
LOC_Os05g12481	48.267	46.644	50.187	66.869	67.85	64.191	0.546560822	1.1E-14
LOC_Os01g68020	1.851	2.693	3.467	3.914	3.929	4.015	0.546398208	0.002820001
LOC_Os04g44870	283.398	267.632	282.437	382.337	396.933	356.597	0.546368348	3.43E-35
LOC_Os01g03310	1.987	2.984	2.997	1.298	1.379	1.503	-0.545945899	0.015347776
LOC_Os08g24182	10.117	9.066	11.403	7.149	5.419	5.928	-0.545674099	0.000440067
LOC_Os01g48420	112.047	117.191	103.813	68.344	72.402	70.172	-0.545358695	2.96E-21
LOC_Os12g32499	0.194	0.773	0.437	1.078	0.819	0.705	0.545159552	0.021257321
LOC_Os01g28970	0.758	0.918	1.468	1.788	2.389	1.824	0.545054285	0.021967978
LOC_Os03g20550	55.106	52.139	51.252	33.212	32.249	31.545	-0.544770532	1.55E-12
LOC_Os01g03390	14.302	12.08	13.919	17.473	18.857	19.613	0.544350158	0.000000996
LOC_Os04g52600	13.298	11.352	12.39	16.972	17.462	16.255	0.544212377	2.94E-14

LOC_Os10g09870	12.59	11.519	10.463	7.116	7.343	6.941	-0.544170804	0.00000412
LOC_Os04g54800	57.304	51.349	58.571	73.919	77.925	76.021	0.543763897	4.78E-20
LOC_Os07g31599	22.171	21.587	23.021	13.189	14.58	14.656	-0.543666166	1.96E-10
LOC_Os04g10530	6.173	6.025	5.966	9.27	8.831	7.24	0.543577747	0.00000675
LOC_Os10g35660	0.924	0.523	0.786	1.782	0.903	1.725	0.543496301	0.022306407
LOC_Os01g09030	44.926	44.227	43.927	59.999	62.997	58.001	0.543141076	2.96E-32
LOC_Os02g15950	7.767	7.823	8.499	10.815	10.73	11.515	0.542890074	1.1E-10
LOC_Os06g08790	9.977	10.313	10.355	6.728	6.377	6.16	-0.542714707	4.48E-09
LOC_Os07g39510	38.759	33.963	34.773	46.61	48.571	51.742	0.542703941	1.49E-11
LOC_Os04g54370	1.87	2.021	2.12	1.181	1.164	1.043	-0.542645146	0.008193494
MSTRG.7559	15.274	16.634	18.354	21.95	22.895	22.662	0.542558643	1.6E-09
LOC_Os09g19310	2.611	1.176	1.668	1.068	0.851	0.285	-0.54229211	0.021338463
LOC_Os06g03580	6.172	4.303	5.186	7.314	7.817	7.025	0.542110731	0.000156629
LOC_Os01g13080	82.318	79.918	79.604	45.042	43.337	59.922	-0.542099347	0.0000938
LOC_Os02g16680	27.787	30.297	28.572	40.49	39.804	38.099	0.542061274	4.57E-14
MSTRG.6549	3.977	4.761	3.393	1.812	3.078	1.993	-0.541875073	0.00740033
LOC_Os09g32940	10.368	10.649	11.019	14.555	13.169	15.87	0.541803452	1.72E-09
LOC_Os03g42770	17.201	16.374	15.573	9.691	11.064	10.204	-0.541747937	5.07E-10
LOC_Os04g25800	4.429	5.676	5.962	2.882	4.227	2.536	-0.541660153	0.00128154
LOC_Os05g11140	11.438	12.779	13.238	17.079	16.768	17.535	0.541578023	2.73E-12
LOC_Os06g43270	14.197	15.427	13.659	19.491	20.841	18.858	0.54153203	4.63E-13
LOC_Os01g07890	3.203	4.682	4.332	6.49	6.578	5.2	0.541079771	0.00476943
LOC_Os08g14860	20.028	22.18	20.262	29.603	27.489	27.892	0.540939366	5.17E-13
LOC_Os05g39830	4.487	6.213	4.708	2.764	2.484	3.705	-0.540910763	0.003924876
LOC_Os01g13770	16.878	18.357	16.397	10.235	12.337	10.506	-0.540845664	9.6E-09
LOC_Os05g31000	223.768	230.662	218.939	139.336	148.094	141.668	-0.540771216	2.47E-37
LOC_Os12g38140	375.92	366.535	354.484	197.025	184.759	276.608	-0.54071627	0.001027028
LOC_Os05g47490	0.445	0.668	0.434	0.776	0.744	0.867	0.540642843	0.009435301
LOC_Os12g25200	1.748	2.138	1.484	1.124	0.865	1.109	-0.540578857	0.00486657
LOC_Os05g03480	106.34	113.217	116.45	154.573	153.36	149.496	0.540226082	1.36E-31
LOC_Os10g30200	4.051	3.752	3.472	2.124	1.55	2.303	-0.540193983	0.014940949
LOC_Os08g37130	7.056	6.032	6.247	7.935	10.093	9.018	0.540007886	0.000034
LOC_Os05g51060	5.733	6.718	6.537	9.09	9.214	8.408	0.539961886	0.0000907
LOC_Os02g16000	41.896	41.096	44.189	58.326	56.865	57.579	0.539926319	8.62E-25
LOC_Os01g34080	6.43	5.505	6.137	8.494	8.367	7.955	0.539805982	4.34E-09

MSTRG.4086	2.091	1.124	1.593	2.086	2.53	2.685	0.539786122	0.007358379
LOC_Os01g52140	0.934	1.019	0.817	1.206	1.63	1.378	0.53970954	0.007510613
LOC_Os11g33100	11.659	14.374	13.382	7.696	7.457	9.29	-0.539539358	0.0000115
LOC_Os04g35380	11.599	11.608	10.497	14.502	16.087	16.662	0.539532356	1.49E-08
LOC_Os11g06234	1.49	1.487	2.09	2.45	2.946	2.206	0.53934348	0.004710032
MSTRG.20515	5.953	5.951	7.179	8.865	8.868	8.443	0.539336758	5.46E-10
LOC_Os03g59640	5.783	6.36	5.584	3.466	3.941	3.616	-0.53920699	0.0000081
LOC_Os07g36170	31.649	28.626	30.118	41.63	42.901	38.355	0.539075213	1.59E-20
LOC_Os03g06240	563.462	556.154	578.482	782.185	757.536	758.466	0.538822679	2.59E-62
LOC_Os07g48690	6.612	6.89	7.592	9.965	9.845	9.171	0.538704323	8.12E-09
LOC_Os02g02970	3.987	3.983	5.201	6.63	6.181	5.759	0.538542752	0.000126582
LOC_Os12g06610	21.264	21.156	20.933	13.548	13.106	13.542	-0.538533912	1.01E-14
LOC_Os01g55030	21.17	26.235	19.627	13.199	13.551	14.998	-0.538431236	0.00000347
LOC_Os09g37344	5.616	5.62	5.669	9.763	6.767	7.191	0.538266028	0.0000723
LOC_Os05g01110	14.509	18.551	15.583	8.932	11.62	9.471	-0.538171018	0.0000731
LOC_Os11g38010	10.295	10.201	9.722	6.007	6.149	6.728	-0.538149966	0.00000113
LOC_Os09g27590	1.056	1.098	1.307	0.356	0.671	0.476	-0.538117614	0.022750459
LOC_Os01g54370	6.147	7.108	6.089	4.116	4.207	3.677	-0.537826263	0.0000164
LOC_Os02g45930	14.058	12.057	14.909	19.179	17.234	20.248	0.537792894	0.00000108
LOC_Os03g53230	84.426	81.611	81.37	49.742	54.931	53.158	-0.53775756	7.27E-28
LOC_Os02g10470	1.237	1.226	1.265	1.79	2.14	2.017	0.537601128	0.013617586
LOC_Os01g42370	0.377	0.467	0.543	0.637	0.882	0.644	0.537586722	0.010481756
MSTRG.7515	16.817	17.233	18.454	20.401	26.119	26.144	0.53749402	0.00000851
LOC_Os07g08810	4.545	4.403	5.024	6.702	6.051	6.65	0.537438009	0.00000775
LOC_Os02g33820	2.912	2.349	2.732	3.855	4.636	3.665	0.537390825	0.007599723
LOC_Os10g41850	1.946	2.114	1.304	1.119	0.915	0.844	-0.537339482	0.014309575
LOC_Os06g25500	12.607	13.163	11.644	16.682	16.758	17.681	0.537148326	6.71E-10
LOC_Os01g50310	44.851	44.92	45.646	28.449	28.507	29.192	-0.537145754	1.69E-17
LOC_Os04g38410	17.819	16.855	19.1	10.598	13.401	9.808	-0.537122374	0.000000406
LOC_Os05g30710	2.296	2.064	2.149	0.868	1.482	1.452	-0.537114873	0.004183581
LOC_Os08g32660	24.111	24.224	24.384	15.526	15.102	15.372	-0.536968393	0.000000103
LOC_Os04g53720	2.659	2.874	3.255	3.892	4.078	4.224	0.536584671	0.0000061
LOC_Os12g33230	1.904	1.488	1.598	0.724	1.232	0.847	-0.536456114	0.009630742
LOC_Os11g32750	2.035	2.696	2.28	1.301	1.316	1.58	-0.536388224	0.001431598
LOC_Os02g37490	2903.047	2859.413	3154.268	4136.656	3931.554	3987.604	0.536307338	3.29E-40

LOC_Os03g04920	32.13	32.034	33.495	44.499	44.433	43.182	0.536289031	1.22E-37
LOC_Os06g48000	3.837	2.812	3.021	5.803	4.254	4.127	0.536222567	0.003312678
LOC_Os05g49130	1.084	1.441	1.875	1.248	0.655	0.536	-0.536126743	0.012969215
LOC_Os03g11540	17.159	19.34	17.804	10.113	11.517	12.683	-0.535722771	1.25E-09
LOC_Os01g72100	36.128	34.934	37.574	22.661	23.238	23.085	-0.535718448	1.69E-12
LOC_Os09g12720	5.563	5.237	5.124	3.21	3.253	3.329	-0.535702081	0.0000587
LOC_Os07g40940	16.211	15.063	15.754	9.333	10.555	9.736	-0.535626634	7.77E-08
LOC_Os01g53030	2.223	1.909	2.106	0.858	1.087	1.069	-0.535559682	0.021569926
LOC_Os11g37970	349.604	347.658	331.247	218.052	230.766	208.694	-0.535493398	5.48E-31
LOC_Os11g19210	0.613	0.904	0.556	0.274	0.349	0.399	-0.535482912	0.02042164
LOC_Os01g62850	5.171	5.528	7.237	9.19	7.652	8.898	0.53540186	0.001071485
LOC_Os11g05394	6.486	5.635	7.972	9.707	9.007	9.038	0.53537086	0.00000106
LOC_Os02g27470	32.215	32.564	30.327	18.595	20.757	21.234	-0.535304873	3.57E-18
LOC_Os02g10860	2.048	3.525	3.41	1.508	1.581	1.848	-0.535160596	0.012345054
LOC_Os05g33690	8.714	8.645	6.696	5.13	4.963	4.967	-0.535057062	0.00000544
LOC_Os04g56120	4.135	4.373	3.919	6.537	5.777	5.171	0.534934367	0.000203342
LOC_Os08g45180	4.83	5.061	4.925	7.041	6.938	7.089	0.534678027	0.0000332
LOC_Os11g07870	4.182	3.665	3.403	2.244	2.326	2.44	-0.534632173	0.00000881
LOC_Os03g03700	8.194	7.948	8.14	11.429	10.384	11.341	0.534372713	6.88E-09
LOC_Os03g20870	7.466	6.768	7.187	9.255	10.768	9.629	0.534285752	0.00000587
LOC_Os11g44870	20.579	21.155	17.892	26.615	28.634	25.919	0.53414351	3.57E-12
MSTRG.4832	18.943	18.993	19.703	26.605	25.464	25.946	0.534025253	2.94E-22
LOC_Os07g39940	4.726	3.512	5.162	1.944	2.99	2.633	-0.533997642	0.009369363
LOC_Os01g15390	2.486	3.449	4.012	2.015	2.14	1.48	-0.533997603	0.009254019
LOC_Os06g45090	16.144	15.91	20.426	25.855	24.386	21.669	0.533850573	0.000000201
LOC_Os04g44300	4.838	4.453	4.409	6.036	6.108	6.72	0.533797759	0.00000112
LOC_Os02g45040	2.468	2.023	1.872	3.169	2.958	2.829	0.533773357	0.00027903
LOC_Os01g21120	559.475	549.981	543.44	728.169	721.503	780.217	0.533727069	3.3E-44
LOC_Os10g32400	9.991	9.799	9.734	13.418	14.847	13.329	0.533574893	0.0000132
LOC_Os03g60850	11.205	12.014	11.266	15.98	14.715	16.306	0.533572995	5.48E-10
LOC_Os01g16960	159.229	157.504	164.218	220.763	214.573	206.438	0.533531333	3.09E-43
MSTRG.13819	22.023	20.961	25.916	30.893	30.918	32.479	0.53352579	8.73E-09
LOC_Os03g40670	12.835	11.99	10.658	14.859	16.854	16.868	0.533365406	7.78E-08
LOC_Os07g03050	9.183	10.122	9.893	14.02	14.569	11.409	0.533331163	0.000000021
LOC_Os01g43280	4.853	4.779	3.967	2.284	3.173	2.821	-0.533223137	0.000593896

LOC_Os01g55280	32.75	33.909	29.668	19.469	21.126	20.459	-0.532990681	3.48E-10
LOC_Os06g51050	389.468	398.515	390.307	521.818	517.509	548.448	0.532762689	2.3E-49
LOC_Os02g11859	5.026	4.979	5.683	7.273	8.027	7.479	0.53275888	0.002381597
LOC_Os11g46210	3.102	3.425	2.703	4.645	3.924	4.189	0.532596582	0.0000122
LOC_Os05g51050	2.906	3.455	3.052	4.434	4.443	4.813	0.53255597	0.002839382
LOC_Os05g01970	11.401	10.006	9.634	5.905	6.548	6.928	-0.532514031	0.0000166
LOC_Os02g53490	5.719	6.002	6.256	8.546	8.143	8.179	0.532500448	0.0000038
LOC_Os02g54710	8.301	7.257	7.014	3.978	5.04	4.698	-0.532488534	0.000742029
LOC_Os01g01660	11.85	9.729	13.881	5.786	7.662	8.376	-0.532421808	0.000212968
LOC_Os02g51640	7.322	7.387	6.197	4.603	4.147	4.184	-0.532307445	0.0000799
LOC_Os08g28670	15.802	15.025	14.306	19.711	21.734	20.119	0.532295114	2.36E-09
LOC_Os07g40020	0.599	0.594	0.698	0.267	0.203	0.416	-0.532265719	0.022677439
LOC_Os03g51740	1.921	2.167	2.846	2.538	3.613	3.946	0.53211263	0.002717603
MSTRG.7344	7.714	7.92	8.858	11.51	10.701	11.226	0.532004892	3.07E-10
LOC_Os05g42000	1.938	1.293	2.297	1.015	1.234	0.86	-0.531931008	0.009865715
LOC_Os09g02440	3.465	2.836	3.661	0.945	2.504	1.766	-0.531909568	0.017790106
LOC_Os02g01765	1.02	1.769	1.17	2.133	1.788	2.301	0.531869237	0.013177397
LOC_Os02g06410	8.137	6.027	7.714	3.734	4.48	5.166	-0.531828811	0.00042215
LOC_Os03g08450	75.19	77.599	75.697	46.807	50.71	48.541	-0.531800374	4.93E-19
LOC_Os05g19150	4.425	5.637	4.264	3.267	3.002	2.24	-0.531730205	0.002966853
LOC_Os01g05970	118.93	122.428	111.415	74.455	76.771	74.279	-0.53169153	6.82E-21
LOC_Os03g06980	32.397	34.354	29.135	19.04	22.819	18.994	-0.531647839	9.96E-10
LOC_Os06g06490	13.724	13.763	13.044	7.877	9.235	8.736	-0.531542303	1.27E-08
LOC_Os02g31030	14.132	16.006	13.185	18.889	20.631	19.541	0.531398613	3.53E-09
LOC_Os01g65992	0.956	0.952	1.611	2.322	2.116	1.636	0.531396675	0.020585141
LOC_Os02g55770	49.988	49.311	51.995	66.527	64.712	61.832	0.531367354	2.5E-31
LOC_Os01g16152	5.312	5.408	5.451	3.338	3.669	2.725	-0.531352278	0.001757492
LOC_Os09g36680	1.569	0.536	0.794	0.365	0.423	0.244	-0.531134294	0.019798699
LOC_Os07g41240	0.961	1.011	1.238	1.535	1.644	1.733	0.531133191	0.009475824
LOC_Os01g31450	0.495	0.368	0.34	0.776	0.484	0.781	0.531123031	0.020054406
LOC_Os08g39150	8.663	8.584	9.395	12.373	11.9	12.036	0.530957416	1.15E-09
LOC_Os12g28015	121.839	108.517	116.02	155.271	153.519	159.149	0.530669314	1.17E-19
LOC_Os01g10210	14.92	13.066	13.989	18.805	18.166	20.445	0.53049766	0.000000155
LOC_Os05g31290	15.164	16.254	17.758	10.44	9.571	10.63	-0.530343942	0.0000324
LOC_Os01g03050	8.402	9.641	9.28	6.145	5.732	5.146	-0.53032007	0.0000406

LOC_Os07g02610	0.623	0.508	0.605	0.813	1.218	1.025	0.530272698	0.021757556
LOC_Os01g70490	7.814	8.057	7.669	10.848	11.803	9.511	0.530245172	2.12E-08
LOC_Os07g40130	162.253	152.712	144.58	86.225	91.271	110.962	-0.530061843	0.00000322
LOC_Os03g12064	47.32	44.042	50.644	62.821	62.976	63.24	0.530025767	3.21E-23
LOC_Os08g28740	3.948	2.329	4.827	6.077	5.516	5.511	0.529357592	0.010457265
LOC_Os10g36626	17.877	17.542	15.995	10.664	11.569	9.658	-0.52913143	0.000103996
LOC_Os08g16660	12.593	13.563	12.499	7.972	8.466	8.062	-0.528526039	2.96E-08
LOC_Os01g14770	5.738	5.708	6.129	8.121	7.785	7.886	0.528451004	1.43E-14
LOC_Os02g06520	22.201	22.185	23.197	30.358	28.513	32.574	0.528426973	2.72E-13
LOC_Os06g04399	18.296	17.625	18.372	24.329	24.946	24.434	0.528409779	1.72E-09
LOC_Os07g05570	12.594	12.75	13.266	16.966	17.225	18.087	0.528371906	3.12E-13
LOC_Os10g20470	41.285	41.741	46.144	60.08	55.58	58.633	0.528262484	2.4E-19
LOC_Os01g60650	17.633	18.257	18.449	12.147	12.179	10.291	-0.528226526	2.82E-10
LOC_Os12g42260	7.975	8.92	8.662	11.323	11.762	11.408	0.528074731	8.83E-09
LOC_Os12g34440	9.018	9.562	8.298	13.89	11.417	11.501	0.528041705	0.000000978
LOC_Os03g11910	8.48	8.502	8.929	12.017	10.167	13.177	0.527858208	0.000000221
LOC_Os09g32670	42.161	41.346	40.001	55.018	53.206	58.101	0.527722357	1.63E-21
LOC_Os01g66890	2.382	2.681	3.214	3.477	4.858	3.527	0.527592464	0.002234904
MSTRG.23494	3.752	3.573	4.155	6.065	5.623	4.747	0.527497509	0.00145352
LOC_Os01g40860	17.712	19.047	17.739	11.431	11.073	12.134	-0.527469422	1.33E-09
LOC_Os07g28720	8.137	6.953	8.486	3.14	4.873	5.596	-0.527450144	0.007130278
LOC_Os02g45570	18.366	17.801	18.751	11.426	10.617	12.196	-0.527384674	0.00000256
LOC_Os04g34180	12.494	12.379	10.673	8.542	6.944	6.688	-0.527330328	0.0000375
LOC_Os08g20410	2.073	1.32	1.765	0.905	1.013	0.712	-0.527300619	0.020304081
LOC_Os08g25380	3.629	3.813	3.354	4.884	5.151	4.738	0.527246499	0.000000649
LOC_Os02g52510	0.691	0.618	0.664	0.293	0.214	0.501	-0.526884386	0.01976488
LOC_Os05g43760	6.833	5.963	7.401	3.996	4.535	3.991	-0.526842575	0.000177065
LOC_Os12g08564	2.207	1.665	1.819	2.526	2.643	3.047	0.526785699	0.007372695
LOC_Os06g37080	9.422	10.485	8.588	6.051	5.241	6.579	-0.526705348	0.00000612
LOC_Os08g40550	0.535	0.458	0.637	1.019	0.849	0.611	0.526697244	0.009095843
LOC_Os08g36490	4.407	4.773	5.206	3.322	2.846	2.81	-0.526601948	0.0000527
LOC_Os12g06920	0.285	0.358	0.366	0.202	0.13	0.034	-0.526550033	0.020285615
LOC_Os03g23970	15.777	14.363	14.204	8.788	9.489	10.266	-0.526471021	0.00000346
LOC_Os10g30640	8.378	7.989	9.505	4.799	5.427	5.714	-0.526451263	0.000356084
LOC_Os03g12879	6.411	6.885	7.628	8.876	9.618	10.593	0.52642938	0.0000863

LOC_Os07g34589	1705.634	1720.7	1859.515	2400.274	2395.828	2298.333	0.526425287	1.06E-43
LOC_Os09g30010	9.42	8.335	9.93	4.762	6.255	6.088	-0.526004434	0.000271395
LOC_Os05g31740	2.223	2.56	1.708	1.159	1.028	1.518	-0.525851387	0.008433537
LOC_Os04g34270	3.119	2.929	2.896	1.778	2.02	1.699	-0.525818948	0.000518926
LOC_Os03g02070	26.328	24.042	27.711	37.621	33.553	35.472	0.525723154	0.000000297
LOC_Os09g32030	3.092	4.235	3.293	1.491	2.267	2.378	-0.525554262	0.007205086
LOC_Os06g04900	0.725	0.988	1.081	0.437	0.325	0.6	-0.525505741	0.022432052
LOC_Os06g38780	0.863	0.952	0.953	1.475	1.342	1.273	0.525422181	0.0057185
MSTRG.14061	26.94	24.243	23.939	14.511	16.382	17.196	-0.525232418	5.94E-14
LOC_Os03g59670	3.384	4.108	3.142	1.959	2.317	1.984	-0.52521212	0.004755871
LOC_Os04g42090	179.098	181.599	181.151	113.005	116.558	119.83	-0.524994513	4.21E-48
LOC_Os07g01560	119.972	118.005	111.775	154.265	153.682	160.211	0.524980628	3.94E-39
LOC_Os03g57910	5.709	5.956	6.737	7.42	8.521	9.662	0.524785934	0.000117246
LOC_Os04g46390	144.901	150.185	161.185	212.429	209.707	190.858	0.524755222	3.48E-26
LOC_Os04g50920	7.076	6.661	6.193	4.096	4.655	3.655	-0.524748103	0.000113465
LOC_Os10g30450	65.03	64.864	66.846	89.924	82.98	91.529	0.52467048	5.37E-22
LOC_Os12g43380	23.672	22.924	30.483	34.383	33.16	37.721	0.52448252	0.000000349
LOC_Os12g04690	0.724	0.756	0.843	0.321	0.325	0.565	-0.524416235	0.018999672
LOC_Os07g19460	5.416	5.051	4.856	4.034	2.687	2.514	-0.52440762	0.00206328
LOC_Os01g66150	6.291	6.323	8.714	9.78	11.019	8.98	0.524369107	0.000152726
LOC_Os03g62590	6.332	6.198	6.443	9.35	8.608	8.218	0.524112913	0.0000258
LOC_Os02g44890	4.274	3.821	3.675	2.41	2.333	2.208	-0.524024732	0.005615896
LOC_Os02g58220	13.68	12.691	12.583	7.734	9.373	7.427	-0.523968529	0.00000941
LOC_Os03g01020	4.369	3.769	4.144	5.523	5.672	5.979	0.52394067	0.000309205
LOC_Os11g17080	8.062	8.231	7.958	4.847	5.073	5.426	-0.523914331	0.00000219
LOC_Os05g36010	0.835	0.517	0.785	0.222	0.449	0.395	-0.523873055	0.021842361
LOC_Os01g71460	0.987	0.9	0.507	0.305	0.232	0.427	-0.523818547	0.023068602
LOC_Os01g36600	8.171	7.816	7.276	4.329	5.265	4.771	-0.523762514	0.000336611
MSTRG.10250	3.399	2.626	2.895	4.492	5.029	3.996	0.52375119	0.001265396
LOC_Os02g02410	274.905	277.331	251.166	169.466	173.89	174.719	-0.52359271	4.91E-38
LOC_Os03g27840	38.405	37.271	40.353	53.054	51.119	51.758	0.523272695	4.68E-21
LOC_Os08g09350	164.354	156.194	149.725	95.921	99.936	107.084	-0.523214592	5.98E-28
LOC_Os09g30130	8.797	9.638	8.799	5.257	6.092	5.972	-0.523032316	4.15E-08
LOC_Os10g27470	3.901	4.043	3.835	2.401	2.71	2.152	-0.52299275	0.000485925
LOC_Os08g06110	40.26	39.777	47.156	57.91	55.304	57.664	0.522875668	2.41E-18

LOC_Os06g03720	17.51	16.398	13.665	8.997	10.818	10.143	-0.522774184	0.00000684
LOC_Os05g38000	1.342	1.33	1.762	0.965	0.746	0.783	-0.522748998	0.011836173
LOC_Os04g39440	14.89	16.004	15.638	18.297	23.536	21.995	0.522648955	0.000000292
LOC_Os12g29760	18.492	17.114	18.63	11.669	11.741	11.064	-0.522577792	0.000000152
LOC_Os01g06740	29.005	27.526	31.933	39.576	38.989	40.678	0.522567528	6.58E-14
LOC_Os09g39380	35.75	35.507	34.251	22.965	23.447	21.195	-0.522503828	5.58E-12
LOC_Os01g03060	101.173	102.185	114.975	146.428	138.007	146.466	0.522492837	5.78E-23
LOC_Os12g40115	43.678	46.875	38.461	26.701	25.034	30.032	-0.522121924	0.000000262
LOC_Os08g06420	7.675	6.465	5.939	4.747	3.713	4.052	-0.52202534	0.000089
MSTRG.1326	5.345	3.837	4.578	5.685	6.35	6.519	0.521917813	0.000581305
LOC_Os12g28090	0.232	0.077	0.497	0.672	0.681	0.395	0.521870584	0.022890731
LOC_Os04g36890	52.872	52.475	52.211	33.745	30.946	36.649	-0.521643587	3.81E-17
LOC_Os08g42030	29.062	29.269	25.261	37.275	35.585	39.665	0.521555438	2.02E-12
LOC_Os01g64350	8.097	7.378	7.23	9.835	10.884	10.137	0.521460779	0.000000191
LOC_Os09g06634	26.73	27.533	27.651	37.229	36.473	36.025	0.521430979	8.08E-29
MSTRG.20251	8.272	8.548	9.38	5.698	5.546	5.286	-0.521264629	0.00000296
LOC_Os03g48170	3.954	4.45	4.817	2.289	3.342	2.496	-0.521192568	0.000841192
LOC_Os06g38670	0.902	0.73	0.744	0.522	0.367	0.434	-0.521070257	0.013024663
LOC_Os08g40870	1.185	1.468	1.004	0.722	0.628	0.267	-0.520905754	0.024000033
LOC_Os01g18240	3.135	2.718	1.916	1.31	1.328	1.908	-0.520886875	0.005667426
LOC_Os01g65690	33.184	33.968	31.138	22.21	21.82	18.524	-0.520837216	1.25E-09
LOC_Os02g17534	39.509	41.221	37.382	53.912	50.574	53.814	0.520820489	5.76E-21
LOC_Os04g41100	32.103	30.39	32.398	42.827	42.503	41.675	0.520775185	3.01E-30
MSTRG.15195	1.475	1.574	2.237	0.602	1.221	0.768	-0.520744912	0.02306535
LOC_Os01g73150	4.309	4.888	5.318	3.164	2.942	2.909	-0.520709152	0.000265517
LOC_Os04g48880	9.085	9.667	10.032	13.147	11.974	14.143	0.520635296	0.00000188
LOC_Os09g03990	1.396	1.064	1.182	0.57	0.735	0.291	-0.520633309	0.024032996
LOC_Os03g18510	71.789	72.522	68.433	44.303	47.95	44.946	-0.520188977	5.71E-21
LOC_Os02g07709	0.756	0.681	0.441	1.325	0.806	1.796	0.520085971	0.023506294
LOC_Os03g08820	26.756	26.641	28.103	36.736	36.606	35.832	0.520027581	4.05E-25
LOC_Os04g28470	2.35	2.497	3.193	3.387	4.54	3.498	0.520017111	0.001287644
LOC_Os04g39880	164.504	176.298	149.298	106.492	108.322	99.939	-0.519993674	6.97E-22
LOC_Os10g35100	86.257	85.092	89.213	51.429	55.654	60.82	-0.519980241	5.34E-19
LOC_Os10g30560	16.411	16.622	15.437	8.766	11.144	10.964	-0.519951086	7.46E-08
LOC_Os02g04800	1.761	2.291	1.662	1.144	0.904	1.095	-0.519950725	0.015297217

LOC_Os09g37500	4.717	3.907	3.758	6.863	5.293	5.928	0.519905041	0.005226252
LOC_Os01g64890	1.778	2.595	1.899	3.279	2.669	3.133	0.51982114	0.004028757
LOC_Os05g35570	2.596	4.516	3.266	2.301	2.302	1.552	-0.519775752	0.004840314
LOC_Os02g55410	17.162	17.534	15.834	10.47	11.036	10.912	-0.519772792	2.34E-11
LOC_Os01g65110	1.297	1.408	0.935	1.55	2.205	1.755	0.519587785	0.010433657
LOC_Os04g46010	5.832	5.336	6.223	3.32	4.269	3.219	-0.519549701	0.000326159
LOC_Os04g44900	63.927	63.747	63.216	86.597	83.868	84.703	0.519507837	1.19E-34
LOC_Os08g44640	37.311	35.94	41.004	49.053	55.088	49.456	0.519263237	4.4E-16
LOC_Os02g35000	7.372	7.799	6.473	3.918	4.918	4.705	-0.519082994	0.0000803
LOC_Os12g09220	0.602	0.954	0.689	1.248	1.471	0.922	0.519060572	0.018250644
LOC_Os11g38810	9.138	10.685	9.979	6.093	6.537	6.211	-0.51902942	0.00000729
LOC_Os07g01870	103.003	109.723	97.622	64.841	65.01	70.265	-0.518857827	8.85E-20
LOC_Os09g36440	51.884	50.951	54.077	73.125	66.054	71.189	0.518597894	3.16E-17
LOC_Os05g41610	1.914	1.979	2.289	2.85	2.645	3.603	0.518466139	0.006404685
LOC_Os04g36070	1.153	1.505	1.911	0.655	0.68	1.086	-0.518408929	0.017463168
MSTRG.3436	49.707	51.401	54.056	32.325	33.195	34.388	-0.518240461	2.85E-14
LOC_Os11g31640	32.938	34.1	32.985	45.422	42.294	46.189	0.518060511	7.2E-19
LOC_Os01g74610	27.095	26.723	27.017	37.802	35.279	34.937	0.518042726	3.2E-29
LOC_Os09g32500	88.146	96.8	88.793	60.31	58.384	57.629	-0.517929262	2.1E-15
LOC_Os04g58810	50.332	53.081	49.955	31.657	33.761	33.504	-0.517922818	1.67E-17
LOC_Os11g33090	19.36	20.605	18.259	25.17	26.498	26.441	0.517839692	2.72E-14
LOC_Os05g28280	95.306	96.539	90.128	60.366	63.398	58.594	-0.51775738	1.62E-25
LOC_Os05g45430	18.763	18.339	19.702	25.715	23.523	27.185	0.517646887	3.55E-11
LOC_Os02g22130	36.506	37.556	40.803	52.482	51.266	50.261	0.517601212	3.5E-17
LOC_Os01g66010	0.752	0.497	0.489	1.059	0.858	1.018	0.51742877	0.021810069
LOC_Os10g39260	3.184	4.071	2.474	4.99	4.068	4.814	0.51742581	0.002330872
LOC_Os10g41360	34.053	34.244	33.318	44.514	45.69	45.646	0.517412457	6.99E-23
LOC_Os03g45170	11.454	10.015	10.719	13.648	14.226	15.528	0.517397403	1.9E-09
LOC_Os04g44780	3.663	3.329	2.999	1.53	1.92	2.422	-0.517396763	0.005461282
LOC_Os11g24484	1.223	1.939	1.495	0.629	0.917	0.882	-0.51735332	0.020317382
LOC_Os04g53950	44.997	40.778	37.657	24.848	24.368	29.725	-0.517232222	1.12E-09
LOC_Os01g57599	2.849	2.884	3.502	4.681	4.408	3.896	0.517116832	0.000770197
LOC_Os01g16870	38.528	37.668	36.509	23.672	24.377	24.785	-0.517022512	1.58E-22
LOC_Os01g28580	0.803	0.796	0.737	1.329	0.935	1.622	0.516979501	0.021055764
LOC_Os01g45390	15.466	14.741	13.765	9.423	8.402	9.992	-0.516900293	0.00000508

LOC_Os03g06070	36.843	35.803	36.91	51.21	47.569	48.733	0.516896843	1.57E-22
LOC_Os03g05500	32.127	33.865	36.399	21.983	21.728	21.764	-0.516880406	4.26E-08
LOC_Os06g49820	0.472	0.639	0.612	0.708	0.863	1.097	0.516824583	0.013608447
LOC_Os05g46340	5.85	5.306	3.917	2.747	2.27	4.048	-0.516815461	0.002613291
LOC_Os08g36610	1.522	0.274	0.761	1.468	3.247	1.872	0.51681007	0.01892217
LOC_Os01g13940	5.992	4.784	4.507	3.424	2.816	3.201	-0.516787967	0.000524826
LOC_Os07g13234	14.796	13.533	15.275	20.794	20.124	18.896	0.516678146	1.31E-11
LOC_Os08g42350	0.483	0.589	0.613	0.968	1.017	0.972	0.516609437	0.023492984
LOC_Os01g68320	114.772	113.771	118.028	156.53	155.421	156.077	0.516532181	3.44E-50
LOC_Os01g74540	20.127	20.809	20.345	12.482	12.346	14.037	-0.516449763	0.00000279
LOC_Os07g12510	19.396	20.222	19.42	25.182	23.165	35.03	0.516322324	0.001423364
LOC_Os05g01760	22.679	22.936	24.108	32.5	31.97	29.064	0.516202678	1.83E-14
LOC_Os04g51060	10.741	10.658	11.073	15.596	14.143	13.863	0.516145151	1.53E-12
LOC_Os01g71990	13.438	14.448	13.307	8.646	8.326	9.295	-0.516136118	9.08E-08
LOC_Os02g02340	1.227	1.933	1.645	2.156	2.585	2.328	0.516135721	0.00646848
LOC_Os03g26220	4.152	5.097	5.114	2.87	3.091	2.515	-0.516134768	0.006309714
LOC_Os04g57830	4.04	4.285	3.626	5.929	5.675	4.901	0.516108721	0.000138534
LOC_Os02g58460	20.163	16.921	18.711	12.319	11.86	11.572	-0.515895606	7.97E-09
LOC_Os01g57870	3.812	3.572	4.139	5.321	4.913	5.452	0.515892402	0.00000209
LOC_Os01g74490	1.477	1.951	1.806	2.85	2.958	2.538	0.515853128	0.01729847
LOC_Os06g09450	684.686	661.158	651.511	895.288	891.938	872.756	0.515737753	7.53E-61
LOC_Os04g23220	1.707	1.808	2.195	1.243	0.9	1.298	-0.515635472	0.00292321
LOC_Os03g55830	10.399	10.608	9.137	6.025	7.027	6.191	-0.515367523	9.71E-08
LOC_Os09g06740	14.247	12.691	17.828	18.893	20.648	21.43	0.515357399	0.00000255
LOC_Os07g31000	1.497	1.813	0.458	4.012	1.626	3.15	0.515248774	0.02107125
LOC_Os01g20980	153.39	147.041	157.284	206.694	189.352	214.415	0.515062689	9.98E-27
LOC_Os08g35780	2.615	3.538	2.494	5.636	4.202	3.646	0.515044267	0.014605572
LOC_Os05g32180	6.645	6.951	6.914	9.839	8.572	9.477	0.51480727	0.00000387
LOC_Os06g51350	2.715	3.353	3.409	1.507	2.291	1.811	-0.514796354	0.005776726
LOC_Os11g05562	147.244	155.532	145.03	92.375	95.145	101.969	-0.514647305	2.15E-18
LOC_Os06g16140	2.203	2.285	1.783	1.295	1.38	1.149	-0.514577617	0.001821533
LOC_Os05g49030	193.239	202.221	201.654	125.408	132.275	129.655	-0.514551537	2.2E-30
LOC_Os05g32630	64.336	65.144	66.023	87.029	90.643	83.134	0.514485196	2.62E-27
LOC_Os07g46852	19.475	18.003	18.87	24.142	25.125	26.518	0.514458379	1.45E-09
LOC_Os07g38130	574.507	562.752	541.454	352.901	339.583	395.303	-0.514428342	3.19E-26

LOC_Os08g42310	14.373	12.705	14.764	19.301	18.529	18.938	0.514264313	3.92E-09
LOC_Os04g47820	11.485	15.33	7.02	6.925	6.239	5.898	-0.514045815	0.012629308
LOC_Os03g51650	26.664	26.183	26.558	36.212	34.299	35.572	0.514033679	1.06E-17
LOC_Os01g02100	7.172	7.976	8.802	11.327	9.214	11.601	0.514015456	0.000001
LOC_Os02g31910	9.027	9.144	8.74	11.432	12.507	12.24	0.513940809	9.61E-10
LOC_Os01g37690	18.914	17.333	17.286	12.27	10.733	12.437	-0.51388516	5.02E-09
LOC_Os03g08900	0.705	0.334	0.646	0.828	0.929	1.133	0.513875914	0.022994454
LOC_Os05g39760	17.242	18.611	17.513	23.125	24.939	23.329	0.513826053	1.26E-15
LOC_Os03g12690	1.643	3.506	1.506	0.488	0.989	0.912	-0.513565408	0.020941408
LOC_Os01g73720	2.546	3.335	2.097	1.582	1.477	1.479	-0.513413101	0.011588869
LOC_Os05g04610	4.8	5.524	4.429	3.078	3.236	2.851	-0.5132688	0.000480199
LOC_Os11g03730	15.36	15.878	15.531	20.021	22.323	20.23	0.513247075	1.43E-13
LOC_Os08g33350	7.208	7.648	8.484	10.801	10.148	11.112	0.513203789	0.0000694
LOC_Os06g37690	1.809	1.816	1.857	2.326	2.476	2.958	0.51283825	0.002006587
LOC_Os02g39970	21.265	20.65	20.298	27.834	28.45	26.891	0.512776245	6.56E-17
LOC_Os01g65100	10.258	11.254	9.432	13.422	14.945	13.205	0.51276043	5.06E-08
LOC_Os06g12370	0.794	0.787	1.116	1.532	1.216	1.384	0.512708197	0.013056312
LOC_Os04g53210	60.446	59.079	65.583	37.939	42.489	39.429	-0.512437249	1.87E-16
LOC_Os04g46710	21.138	19.697	22.995	26.542	29.118	30.942	0.512316362	0.00000195
LOC_Os03g08100	1.792	2.135	2.364	1.094	1.022	1.302	-0.512270959	0.018087276
LOC_Os07g06060	8.834	9.742	9.086	6.003	5.033	5.889	-0.511699468	0.001657595
LOC_Os05g29010	8.113	9.265	8.265	5.162	5.855	5.296	-0.511648135	0.0000152
LOC_Os01g74040	10.062	9.022	9.26	5.61	5.836	6.452	-0.511586785	0.0000378
LOC_Os09g07150	4.08	4.478	3.569	6.117	5.831	5.379	0.51146789	0.003271666
LOC_Os02g27000	17.246	18.125	19.931	25.085	24.812	24.018	0.511458371	4.76E-15
LOC_Os05g43850	33.478	33.821	39.159	47.923	50.02	44.506	0.511422234	7.83E-14
LOC_Os01g67850	13.777	13.945	13.294	18.418	19.292	17.564	0.511358498	5.44E-08
LOC_Os04g19140	2.493	1.98	2.267	1.355	1.161	1.57	-0.511336062	0.002612792
LOC_Os09g20490	1.892	1.315	1.502	0.736	1.077	0.841	-0.511263263	0.013739516
LOC_Os01g55590	23.825	22.892	22.744	31.25	30.433	31.01	0.511042434	2.63E-15
LOC_Os07g01020	178.517	178.48	186.61	118.375	111.713	123.131	-0.510942504	1.24E-27
LOC_Os05g45100	1.602	1.728	1.534	0.446	1.218	0.931	-0.510895025	0.0196878
LOC_Os08g39860	1.603	1.376	1.204	0.533	1	0.797	-0.510474431	0.016123861
LOC_Os06g34660	6.178	8.529	9.102	3.616	6.449	4.195	-0.510345737	0.005201318
MSTRG.19873	2.307	2.569	2.66	1.583	1.628	1.394	-0.510118675	0.002610771

LOC_Os05g44590	12.779	12.7	13.374	17.447	17.201	17.439	0.510111084	1.82E-10
LOC_Os05g19380	1.155	1.707	0.937	0.84	0.685	0.632	-0.510053012	0.012521839
LOC_Os01g68545	2.138	3.178	1.99	1.281	1.647	1.315	-0.510024175	0.009397587
LOC_Os04g39780	1.865	1.816	1.882	2.751	2.444	2.346	0.509958418	0.00000317
LOC_Os09g32270	3.906	3.668	3.903	5.362	5.291	4.799	0.509506796	0.000000056
LOC_Os05g29030	23.064	24.232	25.704	17.101	14.893	15.208	-0.509451762	1.32E-11
LOC_Os11g13810	9.989	10.102	9.38	5.759	7.541	5.617	-0.509249416	0.000000306
LOC_Os05g10370	3.923	3.12	2.414	2.042	1.918	1.397	-0.509224233	0.013209846
LOC_Os11g01140	30.054	27.334	26.072	36.661	38.826	35.64	0.509219767	4.63E-19
LOC_Os02g01520	62.736	61.616	60.676	81.677	80.166	83.856	0.509106801	3.14E-27
LOC_Os07g05420	6.921	6.224	6.833	4.143	3.679	4.673	-0.509029142	0.000281234
LOC_Os09g38400	65.117	67.114	63.753	38.703	44.426	43.608	-0.509009364	1.01E-11
LOC_Os09g17600	44.238	44.555	40.486	28.267	26.362	29.101	-0.508968242	6.45E-16
LOC_Os07g37030	7.974	6.191	7.183	4.544	4.149	4.542	-0.508952288	0.000793737
LOC_Os03g27290	81.215	85.422	79.103	50.368	53.285	55.66	-0.508932029	1.52E-15
LOC_Os01g73780	24.997	27.752	24.786	17.596	17.118	14.687	-0.508900045	0.00000858
LOC_Os08g37180	1.291	1.599	1.315	0.623	0.666	0.938	-0.508869156	0.020401883
LOC_Os01g64090	26.3	29.447	27.249	16.653	20.621	15.642	-0.508862648	0.00000948
LOC_Os02g51790	4.352	4.595	4.514	3.156	1.988	3.094	-0.508861481	0.001766511
LOC_Os01g12710	4.716	4.995	4.473	2.876	3.021	2.982	-0.508816252	0.000287387
LOC_Os01g50420	12.485	12.968	14.406	17.246	17.133	19.225	0.508798082	4.62E-08
LOC_Os01g71170	18.107	18.869	19.121	12.409	9.769	13.347	-0.508569444	0.000029
LOC_Os02g37254	11.194	8.704	10.62	15.449	12.999	13.672	0.508560921	0.000299328
LOC_Os08g43490	10.959	10.47	10.048	12.901	12.757	17.532	0.508061982	0.000191645
LOC_Os04g33990	149.568	149.616	151.223	95.152	99.565	98.69	-0.508046773	2.56E-28
LOC_Os01g14980	18.294	17.7	21.621	10.961	13.346	12.245	-0.508037381	0.0000451
LOC_Os04g46740	31.359	30.444	33.304	40.384	39.721	45.077	0.507821747	1.13E-14
LOC_Os02g51930	79.711	85.427	75.38	48.465	51.31	56.357	-0.507797762	6.83E-17
LOC_Os07g43950	14.127	11.081	12.658	18.43	15.017	17.954	0.50763878	0.000016
LOC_Os03g45344	6.195	6.253	6.108	3.495	4.629	3.162	-0.507628175	0.00322414
LOC_Os01g10350	13.512	13.115	11.197	7.677	7.781	8.598	-0.507582718	0.0000101
LOC_Os03g58890	52.783	50.862	51.74	67.22	70.919	68.432	0.506993646	3.13E-18
LOC_Os12g04130	1.054	0.907	0.966	0.321	0.325	0.8	-0.506974342	0.024315303
LOC_Os04g39380	6.297	5.169	5.361	8.591	7.565	7.87	0.506876066	0.00381661
LOC_Os09g19560	41.285	43.717	41.255	26.459	27.245	28.246	-0.506803433	9.13E-15

LOC_Os01g55880	2.142	1.39	2.065	2.422	2.564	3.225	0.506708591	0.00810096
LOC_Os03g14890	14.999	14.957	15.163	9.841	10.54	8.598	-0.506704687	0.00000314
LOC_Os01g32280	20.406	19.179	18.496	12.599	12.2	12.585	-0.5066684	0.000000038
LOC_Os04g55500	11.44	10.548	9.969	6.372	6.77	7.37	-0.506655031	0.000000225
LOC_Os06g38990	3.939	3.841	2.59	1.877	2.287	2.293	-0.506636387	0.000569337
LOC_Os10g34409	0.663	1.117	0.912	1.792	1.297	1.795	0.50662136	0.025648688
LOC_Os01g02400	7.375	6.788	5.693	9.091	9.524	8.711	0.506296359	0.00000289
LOC_Os09g37860	41.782	43.036	41.298	25.911	27.924	28.267	-0.506101406	5.54E-20
LOC_Os11g36070	34.735	34.195	35.251	47.892	45.571	44.891	0.505942828	2.79E-21
LOC_Os01g74330	8.373	7.992	6.55	5.188	4.394	4.78	-0.505895316	0.000293284
LOC_Os08g24750	0.51	0.759	1.014	0.274	0.416	0.384	-0.5057013	0.025715262
LOC_Os06g04290	131.697	132.504	134.995	85.283	91.384	83.471	-0.505650184	1.49E-19
LOC_Os11g04460	21.667	21.32	22.134	29.148	28.382	28.907	0.50564578	2.51E-20
LOC_Os03g23935	16.17	16.52	16.607	11.049	11.275	9.716	-0.505513701	9.7E-14
LOC_Os01g36550	2.366	2.555	2.979	3.646	4.248	3.313	0.505414635	0.002630862
LOC_Os01g68650	24.954	26.321	25.834	31.596	33.398	38.072	0.505265738	2.81E-09
LOC_Os03g62550	2.532	2.849	2.98	1.417	1.803	1.804	-0.505165194	0.004896265
LOC_Os01g66100	4.03	3.732	4.317	2.071	3.241	2.004	-0.505146743	0.004336428
LOC_Os06g13640	2.766	2.036	2.204	1.414	1.142	1.266	-0.505082884	0.017535151
LOC_Os03g22350	32.51	31.046	31.998	20.933	20.476	20.596	-0.504800991	5.07E-12
LOC_Os03g62740	8.991	10.331	10.132	5.658	5.928	7.139	-0.504798839	0.0000149
LOC_Os02g26390	5.554	5.566	6.433	8.426	7.877	7.38	0.50471582	0.00000147
LOC_Os01g53990	1.583	1.222	1.61	1.913	2.342	2.635	0.504541829	0.011071346
LOC_Os05g04450	0.961	1.088	0.252	0.309	0.224	0.206	-0.504295815	0.018110757
LOC_Os02g42370	2.598	1.99	2.191	2.824	3.167	3.327	0.504210598	0.000318254
LOC_Os06g48630	1.721	2.332	2.254	1.051	1.374	1.204	-0.504088354	0.01234963
LOC_Os03g59180	7.102	7.687	9.104	10.96	10.846	11.099	0.503924382	0.000158475
LOC_Os02g16909	21.799	22.857	18.713	29.609	30.89	25.806	0.503785571	0.000000304
LOC_Os04g05050	22.551	22.526	23.238	30.562	29.358	30.82	0.503765011	8.81E-15
LOC_Os02g01332	180.407	181.192	186.007	117.521	121.506	118.859	-0.503755596	1.72E-28
LOC_Os07g17400	4.812	4.135	4.412	6.016	6.962	5.668	0.503746735	0.001247327
LOC_Os10g40390	8.905	10.317	10.408	6.258	5.814	6.877	-0.503510757	0.00000662
LOC_Os03g55760	0.917	0.984	1.156	0.516	0.486	0.482	-0.503415325	0.025630065
LOC_Os05g31690	7.625	7.583	6.81	9.435	9.424	10.482	0.503365516	7.88E-10
LOC_Os10g40810	30.365	28.838	30.057	18.916	18.741	20.294	-0.503321862	7.09E-12

LOC_Os02g42000	2.937	2.851	3.297	4.51	4.161	4.487	0.503290893	0.006410953
LOC_Os06g03770	27.143	25.032	27.596	37.202	34.942	33.713	0.503262713	1.28E-16
LOC_Os02g01980	1.043	0.443	0.655	0.201	0.408	0.322	-0.503137187	0.025495337
LOC_Os05g30030	28.776	32.244	35.156	21.584	19.667	20.586	-0.503101787	0.00000116
LOC_Os06g04450	6.366	5.795	5.218	3.906	3.476	3.652	-0.503066982	0.0000518
LOC_Os02g51390	3.638	4.126	4.088	5.254	5.477	5.26	0.503011931	0.0000014
LOC_Os08g24380	0.627	0.704	0.767	0.383	0.307	0.396	-0.502736461	0.023292377
LOC_Os02g19970	51.634	51.944	50.954	32.159	33.952	34.669	-0.502715311	7.96E-18
LOC_Os03g06890	1.895	1.613	1.371	1.03	0.914	0.867	-0.502613087	0.012032385
LOC_Os02g28810	48.936	50.261	46.212	30.007	34.714	29.375	-0.502609658	1.41E-08
LOC_Os09g21210	62.573	61.273	58.75	38.654	41.197	39.51	-0.502557555	1.5E-25
LOC_Os03g05910	12.467	13.157	12.774	8.896	8.253	7.349	-0.502497944	0.00000529
LOC_Os06g20570	2.952	2.217	1.823	1.259	1.276	1.541	-0.502476903	0.009790436
LOC_Os03g14280	15.339	16.694	17.266	10.336	11.814	9.492	-0.502407027	0.00000577
LOC_Os02g15820	3.294	3.391	3.89	2.424	2.002	2.232	-0.502209267	0.000324756
MSTRG.14605	1.853	2.295	3.469	3.278	4.002	4.527	0.502069556	0.016562066
LOC_Os05g50180	23.544	20.11	19.354	12.817	13.424	13.912	-0.50201481	0.0000261
LOC_Os03g56050	1.632	2.776	2.22	3.202	2.607	3.607	0.501882996	0.00391772
LOC_Os05g11810	50.605	51.125	49.441	34.392	31.623	32.935	-0.501850839	1.26E-19
LOC_Os06g51410	18.992	18.347	16.245	11.203	11.887	11.637	-0.501757786	4.03E-10
LOC_Os10g40740	5.001	3.97	4.171	2.587	2.357	3.179	-0.501723031	0.001442787
LOC_Os05g33000	12.213	11.615	10.589	15.29	16.39	14.704	0.501637354	0.00000107
LOC_Os08g01920	9.039	10.022	9.51	12.919	12.47	12.616	0.501539453	1.31E-10
LOC_Os04g59624	13.024	11.471	11.085	7.786	8.146	7.879	-0.501537208	3.97E-09
LOC_Os10g30910	10.848	12.334	12.539	15.642	16.242	16.122	0.501503492	8.96E-12
LOC_Os01g46120	6.536	8.495	8.727	4.644	5.018	5.271	-0.501471205	0.000544502
LOC_Os09g35010	11.415	11.604	13.954	8.374	7.267	7.721	-0.501464793	0.000224068
LOC_Os05g15520	45.363	43.423	40.331	28.161	29.018	26.327	-0.501299213	0.000000036
LOC_Os04g44650	3.888	5.18	4.609	1.746	2.36	3.688	-0.501280342	0.013620279
LOC_Os08g21330	4.485	4.006	4.602	2.787	3.142	2.27	-0.501220842	0.000891589
LOC_Os08g42550	23.759	24.823	24.8	31.303	32.303	35.968	0.500890981	1.86E-22
LOC_Os10g33540	10.003	9.228	10.12	12.645	13.042	13.559	0.500826585	6.28E-08
LOC_Os01g49440	2.04	1.841	2.41	0.7	1.152	1.553	-0.500825481	0.02014453
LOC_Os04g56590	27.379	24.734	25.807	16.844	16.679	17.067	-0.500792261	3.64E-10
LOC_Os08g01680	5.385	6.385	4.476	3.168	3.446	3.652	-0.500714551	0.000188957

LOC_Os02g47450	3.481	3.025	4.675	2.121	2.209	2.506	-0.500378438	0.003352762
LOC_Os09g32730	7.159	6.359	5.998	7.263	8.509	11.41	0.500113787	0.00170063

Supplementary Table S3. miRNA Expression Profile of NIP and *rdr3-c1* .

ID	Length	Sequence	NIP-1_count	*rdr3-c1-1* _count	NIP-1_TPM	*rdr3-c1-1* _TPM
**osa-miR396f-5p**	**22**	**TCTCCACAGGCTTTCTTGAACT**	**9359**	**65402**	**40650.124**	**58771.7793**
**osa-miR396e-5p**	**21**	**TCCACAGGCTTTCTTGAACTG**	**9360**	**65387**	**40654.4674**	**58758.2999**
**osa-miR396d**	**21**	**TCCACAGGCTTTCTTGAACGG**	**30**	**256**	**130.3028**	**230.0476**
**osa-miR396g**	**21**	**TCCACAGGCTTTCTTGAACGG**	**30**	**256**	**130.3028**	**230.0476**
**osa-miR396h**	**21**	**TCCACAGGCTTTCTTGAACGG**	**30**	**256**	**130.3028**	**230.0476**
**osa-miR396a-5p**	**21**	**TTCCACAGCTTTCTTGAACTG**	**319**	**2500**	**1385.5529**	**2246.5589**
**osa-miR396b-5p**	**21**	**TTCCACAGCTTTCTTGAACTG**	**319**	**2500**	**1385.5529**	**2246.5589**
**osa-miR396c-5p**	**21**	**TTCCACAGCTTTCTTGAACTT**	**222**	**1155**	**964.2406**	**1037.9102**
osa-miR166a-3p	21	TCGGACCAGGCTTCATTCCCC	138022	654973	599488.3444	588574.1809
osa-miR166d-3p	21	TCGGACCAGGCTTCATTCCCC	138022	654973	599488.3444	588574.1809
osa-miR166f	21	TCGGACCAGGCTTCATTCCCC	138022	654973	599488.3444	588574.1809
osa-miR166b-3p	21	TCGGACCAGGCTTCATTCCCC	137946	654716	599158.244	588343.2347
osa-miR166c-3p	21	TCGGACCAGGCTTCATTCCCC	123226	577268	535223.0132	518746.6358
osa-miR166j-3p	21	TCGGACCAGGCTTCATTCCCC	123212	577199	535162.2052	518684.6308
osa-miR156d	20	TGACAGAAGAGAGTGAGCAC	12918	92065	56108.3772	82731.7797
osa-miR156f-5p	20	TGACAGAAGAGAGTGAGCAC	12908	91981	56064.9429	82656.2954
osa-miR156h-5p	20	TGACAGAAGAGAGTGAGCAC	12908	91981	56064.9429	82656.2954
osa-miR156j-5p	20	TGACAGAAGAGAGTGAGCAC	12908	91981	56064.9429	82656.2954
osa-miR166g-3p	21	TCGGACCAGGCTTCATTCCTC	16013	86782	69551.2807	77984.3514
osa-miR319b	20	TTGGACTGAAGGGTGCTCCC	9687	52174	42074.7677	46884.7866
osa-miR319a-3p.2-3p	20	TTGGACTGAAGGGTGCTCCC	9240	50188	40133.2563	45100.1201
osa-miR156b-5p	20	TGACAGAAGAGAGTGAGCAC	4567	31428	19836.4266	28241.9418
osa-miR156c-5p	20	TGACAGAAGAGAGTGAGCAC	4566	31419	19832.0832	28233.8542
osa-miR156g-5p	20	TGACAGAAGAGAGTGAGCAC	4566	31419	19832.0832	28233.8542
osa-miR156a	20	TGACAGAAGAGAGTGAGCAC	4560	31347	19806.0226	28169.1533
osa-miR156e	20	TGACAGAAGAGAGTGAGCAC	4560	31347	19806.0226	28169.1533
osa-miR156i	20	TGACAGAAGAGAGTGAGCAC	4560	31347	19806.0226	28169.1533
miR397-x	22	TTGAGTGCAGCGTTGATGAACC	6306	23665	27389.6444	21265.927
osa-miR535-5p	21	TGACAACGAGAGAGAGCACGC	3769	22681	16370.3726	20381.6814
osa-miR166m	21	TCGGACCAGGCTTCATTCCCT	2319	17452	10072.4049	15682.7787

osa-miR166l-3p	21	TCGGACCAGGCTTCAATCCCT	2313	12564	10046.3444	11290.3066
osa-miR166k-3p	21	TCGGACCAGGCTTCAATCCCT	2223	12178	9655.436	10943.4379
osa-miR167d-5p	21	TGAAGCTGCCAGCATGATCTG	2457	12101	10671.7977	10874.2439
osa-miR167j	21	TGAAGCTGCCAGCATGATCTG	2457	12101	10671.7977	10874.2439
osa-miR167f	21	TGAAGCTGCCAGCATGATCTG	2455	12080	10663.1108	10855.3728
osa-miR167h-5p	21	TGAAGCTGCCAGCATGATCTG	2455	12080	10663.1108	10855.3728
osa-miR167g	21	TGAAGCTGCCAGCATGATCTG	2455	12078	10663.1108	10853.5756
osa-miR166h-3p	21	TCGGACCAGGCTTCATTCCTC	1493	9849	6484.735	8850.5436
osa-miR1859	22	TTTCCTATGACGTCCATTCCAA	2069	9122	8986.5484	8197.2443
osa-miR164e	21	TGGAGAAGCAGGGCACGTGAG	1776	8513	7713.9246	7649.9825
osa-miR159a.1	21	TTTGGATTGAAGGGAGCTCTG	1205	8013	5233.8283	7200.6707
osa-miR159b	21	TTTGGATTGAAGGGAGCTCTG	1205	8013	5233.8283	7200.6707
osa-miR166i-3p	21	TCGGATCAGGCTTCATTCCTC	1328	7457	5768.0697	6701.036
osa-miR1876	24	ATAAGTGGGTTTGTGGGCTGGCCC	1968	7410	8547.8624	6658.8007
osa-miR1423-5p	24	AGGCAACTACACGTTGGGCGCTCG	1465	7187	6363.1191	6458.4077
osa-miR820a	21	TCGGCCTCGTGGATGGACCAG	1150	5979	4994.9399	5372.8704
osa-miR820b	21	TCGGCCTCGTGGATGGACCAG	1150	5979	4994.9399	5372.8704
osa-miR820c	21	TCGGCCTCGTGGATGGACCAG	1150	5979	4994.9399	5372.8704
osa-miR162a	21	TCGATAAACCTCTGCATCCAG	1205	5645	5233.8283	5072.7301
osa-miR444b.1	21	TGTTGTCTCAAGCTTGCTGCC	1116	5194	4847.2634	4667.4509
osa-miR444c.1	21	TGTTGTCTCAAGCTTGCTGCC	1116	5194	4847.2634	4667.4509
osa-miR168a-5p	21	TCGCTTGGTGCAGATCGGGAC	675	3965	2931.8126	3563.0425
osa-miR1425-5p	21	TAGGATTCAATCCTTGCTGCT	710	3915	3083.8325	3518.1113
osa-miR167i-5p	21	TGAAGCTGCCAGCATGATCTG	468	3417	2032.7234	3070.5968
osa-miR167e-5p	21	TGAAGCTGCCAGCATGATCTG	468	3412	2032.7234	3066.1036
osa-miR167a-5p	21	TGAAGCTGCCAGCATGATCTA	463	3356	2011.0062	3015.7807
osa-miR167c-5p	21	TGAAGCTGCCAGCATGATCTA	463	3356	2011.0062	3015.7807
osa-miR167b	21	TGAAGCTGCCAGCATGATCTA	461	3354	2002.3194	3013.9835
osa-miR1862d	24	ACTAGGTTTGTTTATTTGGGACG	969	3064	4208.7798	2753.3826
osa-miR444b.2	21	TGCAGTTGTTGTCTCAAGCTT	485	2870	2106.5616	2579.0497
osa-miR444c.2	21	TGCAGTTGTTGTCTCAAGCTT	485	2870	2106.5616	2579.0497
osa-miR408-3p	21	CTGCACTGCCTCTTCCCTGGC	681	2713	2957.8731	2437.9658

osa-miR5144-5p	21	TTCTTGTGCTGCTGAAGAGAC	356	2099	1546.2597	1886.2109
osa-miR159f	21	CTTGGATTGAAGGGAGCTCTA	301	2013	1307.3712	1808.9293
osa-miR528-5p	21	TGGAAGGGGCATGCAGAGGAG	648	1522	2814.5401	1367.7051
miR171-y	21	TTGAGCCGCGTCAATATCTCT	206	1496	894.7458	1344.3409
novel-m0001-5p	24	ACAACGGATGGCCTAGATTTCATC	659	1492	2862.3177	1340.7464
miR894-z	19	ATTCACGTCGGGTTCACCA	410	1449	1780.8047	1302.1056
osa-miR1871	24	ATGGCTCTGATATCATGTTGGTTT	154	1020	668.8876	916.596
miR166-y	21	TCGGACCAGGCTTCATTTTTT	293	921	1272.6238	827.6323
osa-miR1873	24	TCAACATGGTATCAGAGCTGGAAG	121	880	525.5545	790.7887
miR5054-z	18	TCCCCACGGACGGCGCCA	180	859	781.8167	771.9177
novel-m0002-3p	24	AGTAACACCAACCGAGACTAAAGT	237	830	1029.392	745.8576
novel-m0003-5p	24	AGTAACACCAACCGAGACTAAAGT	237	830	1029.392	745.8576
osa-miR160b-5p	21	TGCCTGGCTCCCTGTATGCCA	112	785	486.4637	705.4195
osa-miR160c-5p	21	TGCCTGGCTCCCTGTATGCCA	112	785	486.4637	705.4195
osa-miR160d-5p	21	TGCCTGGCTCCCTGTATGCCA	112	784	486.4637	704.5209
osa-miR160a-5p	21	TGCCTGGCTCCCTGTATGCCA	112	778	486.4637	699.1291
osa-miR5150-3p	24	AGAAGCTGCAGCTGTCAGAAGCTC	149	761	647.1705	683.8525
osa-miR2877	24	TTGCATCCTCTGCACTTTGGGCCT	227	740	985.9577	664.9814
novel-m0004-3p	24	ACCAGTGACTGTAATGATAGTGAC	146	740	634.1402	664.9814
novel-m0005-3p	24	ACCAGTGACTGTAATGATAGTGAC	146	740	634.1402	664.9814
osa-miR390-5p	21	AAGCTCAGGAGGGATAGCGCC	131	701	568.9888	629.9351
novel-m0006-5p	24	AAGCGTGCTCACGGAAAACGAGGG	193	674	838.2812	605.6723
osa-miR168a-3p	24	GATCCCGCCTTGCACCAAGTGAAT	200	636	868.6852	571.5246
osa-miR162b	21	TCGATAAGCCTCTGCATCCAG	73	615	317.0701	552.6535
osa-miR160e-5p	21	TGCCTGGCTCCCTGTATGCCG	74	584	321.4135	524.7962
miR166-z	20	TCGGACCAGGCTTCATTTTT	156	580	677.5745	521.2017
osa-miR1432-5p	21	ATCAGGAGAGATGACACCGAC	84	493	364.8478	443.0214
osa-miR5794	21	TGAGGAATCACTAGTAGTCGT	41	490	178.0805	440.3256
osa-miR1874-3p	24	TATGGATGGAGGTGTAACCCGATG	138	450	599.3928	404.3806
novel-m0014-3p	24	AATAACCGGGAGTAAAGATCGATC	120	435	521.2111	390.9013
novel-m0007-5p	21	AGCTGCCGACTCATTCACCCA	118	429	512.5243	385.5095
miR812-z	24	GACGGACGGTCAAACGTTGGGCAC	103	429	447.3729	385.5095

osa-miR1868	24	TCACGGAAAACGAGGGAGCAGCCA	140	422	608.0796	379.2192
novel-m0026-3p	24	TAACCGGGACTAAAGATCATCTTT	118	407	512.5243	365.7398
novel-m0015-5p	24	AAAACCGGGGCTAAAGATGATCTT	190	402	825.2509	361.2467
novel-m0009-5p	24	AATACCAACCGGGACTAATGATCC	129	400	560.302	359.4494
novel-m0008-3p	24	AATACCAACCGGGACTAATGATCC	128	398	555.9585	357.6522
osa-miR1862a	24	ACGAGGTTGGTTTATTTTGGGACG	108	394	469.09	354.0577
osa-miR1862b	24	ACGAGGTTGGTTTATTTTGGGACG	108	394	469.09	354.0577
osa-miR1862c	24	ACGAGGTTGGTTTATTTTGGGACG	108	394	469.09	354.0577
osa-miR166e-3p	21	TCGAACCAGGCTTCATTCCCC	61	391	264.949	351.3618
novel-m0054-3p	22	TTTACTCGTGATGACGTGGACA	1	390	4.3434	350.4632
osa-miR5814	24	AATCAAGTTAGGAACCATGCAAGT	90	385	390.9083	345.9701
osa-miR1883a	24	ACCTGTGACGGGCCGAGAATGGAA	89	377	386.5649	338.7811
miR319-y	23	CTTGGACTGAAGGGTGCTCCCTT	135	374	586.3625	336.0852
osa-miR1320-5p	21	TGGAACGGAGGAATTTTATAG	93	350	403.9386	314.5183
novel-m0011-3p	24	GTTTAGGGACCTAGATGACATACC	112	347	486.4637	311.8224
novel-m0010-5p	24	ACCGGGATGACATAGTCGAACAAG	104	326	451.7163	292.9513
osa-miR1862e	24	CTAGATTTGTTTATTTTGGGACGG	89	306	386.5649	274.9788
novel-m0017-3p	22	TCCTGCGGCATGTCGAGGGCCT	71	305	308.3832	274.0802
novel-m0027-5p	21	TCGTCCTCTCTTGTTCTTGTT	42	284	182.4239	255.2091
novel-m0019-5p	22	TTACCAACCGGGACTAAAGATC	36	273	156.3633	245.3242
novel-m0020-3p	22	TTACCAACCGGGACTAAAGATC	36	273	156.3633	245.3242
novel-m0031-5p	24	TTTGGGACCTAGATGACACACTAT	100	268	434.3426	240.8311
novel-m0016-5p	24	TGTAATTGTGTCGTGGGCTAAGCC	71	265	308.3832	238.1352
novel-m0047-3p	22	TATCGACTGATACCTGGTACCC	46	260	199.7976	233.6421
miR2120-x	24	TAACACCAACCGGGACTAAAGATC	58	255	251.9187	229.149
miR11336-y	20	TGGGAAATGCTAGAATGACT	62	250	269.2924	224.6559
osa-miR2106	21	CCGAGGTTTTCTGGATACATT	79	237	343.1307	212.9738
osa-miR531a	24	CTCGCCGGGGCTGCGTGCCGCCAT	68	232	295.353	208.4807
osa-miR531c	24	CTCGCCGGGGCTGCGTGCCGCCAT	68	232	295.353	208.4807
osa-miR164a	21	TGGAGAAGCAGGGCACGTGCA	53	229	230.2016	205.7848
osa-miR164b	21	TGGAGAAGCAGGGCACGTGCA	53	229	230.2016	205.7848
osa-miR164f	21	TGGAGAAGCAGGGCACGTGCA	53	229	230.2016	205.7848

osa-miR319a-3p	21	ACTGGATGACGCGGGAGCTAA	37	223	160.7068	200.3931
novel-m0032-5p	24	AATATTCCACGCTTGAGACTTGAG	73	219	317.0701	196.7986
miR156-z	22	CTTGACAGAAGAGAGTGAGCAC	55	218	238.8884	195.8999
osa-miR812h	24	AAGACGGATGATTAAAGTTGGACA	66	217	286.6661	195.0013
osa-miR812i	24	AAGACGGATGATTAAAGTTGGACA	66	217	286.6661	195.0013
osa-miR812j	24	AAGACGGATGATTAAAGTTGGACA	66	217	286.6661	195.0013
novel-m0018-3p	21	TTTGCATGACCAAGGAGCCGA	51	216	221.5147	194.1027
osa-miR444a-3p.2	21	TGCAGTTGCTGCCTCAAGCTT	35	214	152.0199	192.3054
osa-miR444e	21	TGCAGTTGCTGCCTCAAGCTT	35	214	152.0199	192.3054
miR408-y	19	TGCACTGCCTCTTCCCTTT	52	212	225.8582	190.5082
osa-miR5083	20	AGACTACAATTATCTGATCA	33	209	143.3331	187.8123
osa-miR444d.2	21	TGCAGTTGCTGCCTCAAGCTT	35	209	152.0199	187.8123
miR159-y	21	TTTGGATTGAAGGGAGCTTTT	61	206	264.949	185.1165
novel-m0012-3p	24	GGTTTGTTTATTTTGGGACGGAGG	91	200	395.2518	179.7247
novel-m0013-3p	24	GGTTTGTTTATTTTGGGACGGAGG	91	200	395.2518	179.7247
osa-miR1883b	24	ACCTGTGACGGGCCGAGAATGGAA	45	189	195.4542	169.8399
osa-miR531b	20	CTCGCCGGGGCTGCGTGCCG	61	187	264.949	168.0426
miR156-x	21	TGACAGAAGAGAGTGAGCTTT	65	184	282.3227	165.3467
novel-m0044-3p	24	ATTTGTTGTATTAGGGAATGTCTC	41	182	178.0805	163.5495
novel-m0036-3p	24	ACACCAACCAGGACTAAAGATCCC	54	167	234.545	150.0701
novel-m0023-3p	24	ATCAATATGCATGTGGGAAATACT	48	166	208.4844	149.1715
novel-m0025-5p	24	TCCTAGGACAATGTATGTAGACAT	34	166	147.6765	149.1715
novel-m0045-3p	24	ATGTCCAGATTCGTAGTACTAGGA	55	165	238.8884	148.2729
miR8175-z	18	TCCCCGGCAACGGCGCCA	45	164	195.4542	147.3743
novel-m0022-5p	24	AAGACACCCGTAGTGACTTCGTCA	51	161	221.5147	144.6784
osa-miR1863b.2	23	AGAGACTTGGCTGATGCATTACT	65	160	282.3227	143.7798
miR812-y	24	GAACGGTCAAACGTTGGACACGGA	45	160	195.4542	143.7798
osa-miR1428e-3p	21	TAAGATAATGCCATGAATTTG	13	158	56.4645	141.9825
osa-miR2880	24	ACGGTATCCCGTTCGGACAGGATG	38	158	165.0502	141.9825
osa-miR5150-5p	24	AGCTTCTGACAGCTGCAGTTTCTC	37	156	160.7068	140.1853
novel-m0021-3p	24	AGCAAATTTTGGTATGGTAGAGAA	46	154	199.7976	138.388
miR160-x	21	TGCCTGGCTCCCTGTATGCTT	61	148	264.949	132.9963

novel-m0103-3p	24	ATATCCAGATTCGTAGTACTAGGA	54	148	234.545	132.9963
novel-m0029-5p	24	AATAGAGATCAGCACTGTAGATGC	24	148	104.2422	132.9963
osa-miR1863a	24	AGCTCTGATACCATGTTAGATTAG	39	146	169.3936	131.199
osa-miR6250	22	GGGGATAGATCGACGCGTCAAG	50	145	217.1713	130.3004
novel-m0041-5p	24	AGTATCCCGCATGATCTCTGACCA	34	144	147.6765	129.4018
novel-m0043-5p	24	AGTATCCCGCATGATCTCTGACCA	34	144	147.6765	129.4018
miR11339-y	21	AATATGAATGTGGGAAATGCT	13	143	56.4645	128.5032
novel-m0033-5p	24	ACAATGTATCTGGATATGAGACGT	37	142	160.7068	127.6045
miR820-z	23	TCGGCCTCGTGGATGGACCAGGT	46	138	199.7976	124.0101
novel-m0024-3p	23	TGCTCACTGCTCTGTCTGTCATC	59	138	256.2621	124.0101
novel-m0037-5p	24	AGATATCGAGACCACGTATGTACT	22	138	95.5554	124.0101
novel-m0067-5p	24	CCTCGCCGGCGCGCGTGCTCACCC	73	137	317.0701	123.1114
osa-miR444d.3	21	TTGTGGCTTTCTTGCAAGTTG	26	135	112.9291	121.3142
osa-miR156j-3p	22	GCTCGCTCCTCTTTCTGTCAGC	44	133	191.1107	119.5169
osa-miR5149	22	GAGGAGCTGTGACGATTTGGGA	44	133	191.1107	119.5169
osa-miR1879	24	GTGTTTGGTTTAGGGATGAGGTGG	30	131	130.3028	117.7197
osa-miR11339-3p	21	TATGAATGTGGGCAATGCTAG	17	130	73.8382	116.8211
novel-m0028-5p	24	AGCATCCGTCACAGGCTAGAAGTC	39	128	169.3936	115.0238
novel-m0050-3p	24	AATGATCGGAAATGATTTGGTACC	34	128	147.6765	115.0238
novel-m0051-3p	24	AATGATCGGAAATGATTTGGTACC	34	128	147.6765	115.0238
osa-miR2055	21	TTTCCTTGGGAAGGTGGTTTC	24	125	104.2422	112.3279
miR402-x	18	CCTTGGCCTGATGAACCT	0	124	0.01	111.4293
novel-m0040-5p	24	ACGAATCCAGGACTAAAGATACCC	31	124	134.6462	111.4293
novel-m0030-5p	24	ACGGAATGATACCTCTGAAGTTGG	36	123	156.3633	110.5307
novel-m0035-5p	24	AACACCAATCGGGACTAAAGATGG	28	123	121.6159	110.5307
novel-m0034-3p	22	TTGGTCGTGGATGTGGATCACT	23	122	99.8988	109.6321
novel-m0038-3p	24	AGTATCAACCGGGACTAAAGATCC	32	122	138.9896	109.6321
osa-miR160f-5p	21	TGCCTGGCTCCCTGAATGCCA	20	121	86.8685	108.7335
osa-miR2871a-5p	24	GACCGTAGAAACTAGCATAGAAAA	17	116	73.8382	104.2403
novel-m0064-5p	24	AACACCAACCAGGACTAAAGATCA	22	116	95.5554	104.2403
novel-m0066-5p	24	AACACCAATCGGGACTAAAGATCC	29	115	125.9594	103.3417
miR812-x	20	TCCGTGTCCAACGTTTGACT	37	113	160.7068	101.5445

novel-m0042-5p	24	AGTATCCCGCATGATCTCTGACCA	29	110	125.9594	98.8486
novel-m0049-3p	24	AATGATCGGAAATGATTTGGTACC	21	107	91.2119	96.1527
osa-miR397a	21	TCATTGAGTGCAGCGTTGATG	14	102	60.808	91.6596
osa-miR397b	21	TTATTGAGTGCAGCGTTGATG	14	102	60.808	91.6596
novel-m0169-5p	21	TTTGAACCGGGACTAAAGATC	13	102	56.4645	91.6596
osa-miR812n-5p	24	AAGTGCAGCCATGAGTTTCCGTGC	42	102	182.4239	91.6596
osa-miR171b	21	TGATTGAGCCGTGCCAATATC	12	101	52.1211	90.761
osa-miR171c-3p	21	TGATTGAGCCGTGCCAATATC	12	101	52.1211	90.761
osa-miR171f-3p	21	TGATTGAGCCGTGCCAATATC	12	101	52.1211	90.761
novel-m0071-5p	24	AAGAGATCCGTCAATGAAAATAGA	23	100	99.8988	89.8624
novel-m0102-5p	22	TTACCAACCAGGACTAAAGATC	17	97	73.8382	87.1665
miR5059-z	18	TCCTGGGCAGCAACACCA	25	94	108.5857	84.4706
novel-m0048-3p	24	AACACCAACATGGACTAAAGACCA	32	92	138.9896	82.6734
osa-miR171d-3p	21	TGATTGAGCCGTGCCAATATC	10	91	43.4343	81.7747
osa-miR171e-3p	21	TGATTGAGCCGTGCCAATATC	10	91	43.4343	81.7747
osa-miR5082	24	TGCGATGATGGCCGCGCGGGTTCA	16	91	69.4948	81.7747
novel-m0202-3p	24	AGATATCAGACCTTGGTACCTGGG	27	91	117.2725	81.7747
novel-m0282-3p	25	ATGGTTCAGAGACAGTGCGACATCA	16	91	69.4948	81.7747
miR535-z	22	CTGACAACGAGAGAGAGCACGC	27	90	117.2725	80.8761
osa-miR1874-5p	24	TAGGGCTACTACACCATCCATAAG	34	88	147.6765	79.0789
miR5139-z	18	AACCTGGCTCTGATACCA	13	86	56.4645	77.2816
novel-m0117-5p	24	TCTTTAGTCCCGGATTGGTACTCC	25	86	108.5857	77.2816
novel-m0119-3p	24	AACTTACGAAAACCGGGCAAACGA	25	86	108.5857	77.2816
osa-miR5159	24	AACTAGAGTGGGTCAACGGGTACC	20	85	86.8685	76.383
miR6478-z	23	CCGACCTTAACTCAGTTGGTAGA	62	83	269.2924	74.5858
novel-m0039-5p	24	ATCCACGGATTGATTGATGTCACT	34	83	147.6765	74.5858
novel-m0101-5p	22	TTACCAACCAGGACTAAAGATC	17	82	73.8382	73.6871
novel-m0052-5p	24	ACGAGATCATAGTACAACGAATGT	20	81	86.8685	72.7885
novel-m0150-3p	19	TAACCGGGACTAAAGATTT	21	80	91.2119	71.8899
novel-m0059-5p	24	AAATACCATCGTACGTGAGAATGA	12	80	52.1211	71.8899
novel-m0060-5p	24	AAATACCATCGTACGTGAGAATGA	12	80	52.1211	71.8899
novel-m0061-5p	24	AAATACCATCGTACGTGAGAATGA	12	80	52.1211	71.8899

novel-m0062-5p	24	AAATACCATCGTACGTGAGAATGA	12	80	52.1211	71.8899
novel-m0142-5p	24	ATTAAAGTATGTGACTTGGTACCC	15	78	65.1514	70.0926
osa-miR1860-3p	22	ATCTGGAAGCTAGGTTTTCTCT	24	77	104.2422	69.194
novel-m0143-5p	24	GTTTGAGGATGGCATTTTAACGAA	23	77	99.8988	69.194
novel-m0224-3p	24	ATACCAACCAGGACTAAAGATCCC	12	77	52.1211	69.194
novel-m0225-5p	19	GAACACTGTGAATGCACTG	0	76	0.01	68.2954
osa-miR812g	24	AAGACGGATGATTAAAGTTGGACA	28	76	121.6159	68.2954
novel-m0055-3p	24	AAACAGTGTAGGATATGTCCCATC	14	76	60.808	68.2954
novel-m0056-3p	24	ATCCAGTGCGGACCACGTAACACG	18	74	78.1817	66.4981
osa-miR827	21	TTAGATGACCATCAGCAAACA	22	73	95.5554	65.5995
osa-miR394	20	TTGGCATTCTGTCCACCTCC	12	72	52.1211	64.7009
osa-miR166b-5p	21	GGAATGTTGTCTGGCTCGGGG	16	70	69.4948	62.9037
miR529-x	21	AGAAGAGAGAGAGTACAGCCT	14	70	60.808	62.9037
novel-m0075-3p	24	ATATCCAGATTCGTTGTACTAGGA	45	70	195.4542	62.9037
novel-m0120-3p	24	ATGTCCAGATTCGTTGTACTAGGA	19	70	82.5251	62.9037
novel-m0121-3p	24	ATGTCCAGATTCGTTGTACTAGGA	19	70	82.5251	62.9037
novel-m0199-3p	24	ACCAACCGAGACTAAAGATAGAGC	11	70	47.7777	62.9037
osa-miR164d	21	TGGAGAAGCAGGGCACGTGCT	17	69	73.8382	62.005
novel-m0272-3p	24	CTCCCCAAGCAGCTCTAGACATTC	19	69	82.5251	62.005
osa-miR1877	24	AGATGACATGTGAATGATGAGGGG	26	68	112.9291	61.1064
miR1511-y	18	AACCTGGCTCTGATACCA	9	67	39.0908	60.2078
miR8155-z	18	AACCTGGCTCTGATACCA	11	66	47.7777	59.3092
novel-m0082-3p	24	AACACTAACCGGGACTAAAGATCC	24	66	104.2422	59.3092
novel-m0093-5p	24	AAACCGGTACCTGTAGACATCACA	13	66	56.4645	59.3092
osa-miR1850.1	21	TGGAAAGTTGGGAGATTGGGG	28	65	121.6159	58.4105
novel-m0083-3p	24	AACACTAACCGGGACTAAAGATCC	23	65	99.8988	58.4105
osa-miR1866-5p	24	GAGGGATTTTGCGGGAATTTCACG	48	64	208.4844	57.5119
novel-m0046-5p	24	GCTGGCTTTTGAACCCATTTATGG	27	64	117.2725	57.5119
novel-m0113-3p	21	AACACCGGATTCGAATCTTTT	7	62	30.404	55.7147
osa-miR1878	24	ACTTAATCTGGACACTATAAAAGA	16	62	69.4948	55.7147
novel-m0167-5p	24	GTAACACCAACCGAGACTAAAGAT	22	62	95.5554	55.7147
novel-m0168-3p	24	GTAACACCAACCGAGACTAAAGAT	22	62	95.5554	55.7147

novel-m0063-5p	24	AAGAATACCTGACACTGATGTGAG	20	61	86.8685	54.816
novel-m0057-3p	24	GATGGAGACTTGAACCCATGACCT	20	61	86.8685	54.816
novel-m0058-3p	24	GATGGAGACTTGAACCCATGACCT	20	61	86.8685	54.816
novel-m0076-5p	24	ATCACCCGATACGTCGTAGAAATC	15	61	65.1514	54.816
novel-m0545-3p	24	AAATTTTGGCACACGAACTAAACA	10	61	43.4343	54.816
novel-m0153-5p	18	TACCGGATGTGACATATT	7	60	30.404	53.9174
osa-miR408-5p	21	CAGGGATGAGGCAGAGCATGG	11	60	47.7777	53.9174
miR11340-y	24	AATGGTGTAATCGGATTGTAGATC	14	60	60.808	53.9174
osa-miR2876-3p	21	TTCCTATATGAACACTGTTGC	9	59	39.0908	53.0188
novel-m0094-3p	24	AGATCCGTAGCACTCGAATGTGTC	12	59	52.1211	53.0188
osa-miR5801c-5p	21	ATCGTTTCCGATCGTTGGATC	6	58	26.0606	52.1202
osa-miR1863c	24	TAGAAACTTGGCTGATGCATTACT	9	58	39.0908	52.1202
novel-m0073-5p	24	AGTCACTGGTATGTAGGTCTCACC	24	58	104.2422	52.1202
novel-m0074-3p	24	AGTCACTGGTATGTAGGTCTCACC	24	58	104.2422	52.1202
novel-m0072-3p	24	ATATCCAGATCCATTGTACTAAGA	10	58	43.4343	52.1202
osa-miR5504	21	AGTGACGGGAGGACTGCAAGG	5	57	21.7171	51.2215
miR535-x	21	TGACAACGAGAGAGAGCACTA	8	57	34.7474	51.2215
miR1862-z	24	ACGAGATTGGTTTATTTTGGAACG	28	57	121.6159	51.2215
miR5160-z	24	GCGAGATCGACGTTATATTTCTGC	8	57	34.7474	51.2215
novel-m0097-5p	24	AGTACCGGATGTGACATATATAGT	11	57	47.7777	51.2215
miR2916-z	19	TGGGGGCTCGAAGACGATA	14	56	60.808	50.3229
novel-m0161-3p	20	TCAACCGGGACTAAAGATTT	12	56	52.1211	50.3229
osa-miR1846d-5p	22	TCCCACCGAGCAGCCGGATCTC	13	56	56.4645	50.3229
novel-m0077-5p	24	GTTGAAACTGTTTGGCTTGGCTCC	11	56	47.7777	50.3229
osa-miR166d-5p	21	GGAATGTTGTCTGGCTCGAGG	13	55	56.4645	49.4243
miR11339-x	24	AGCATTTCCCACATTCATATTGAT	16	55	69.4948	49.4243
novel-m0081-3p	24	AACACCAACCAGGACTAAAGTCCC	18	55	78.1817	49.4243
novel-m0190-5p	24	TTAGAGATGATGTGACTGAAAAGT	22	55	95.5554	49.4243
novel-m0068-5p	24	AGCTCACCGCTGGCTGTGACTCCT	17	55	73.8382	49.4243
novel-m0122-3p	24	AATAATCGGAAACGACTTGGTACC	12	55	52.1211	49.4243
novel-m0130-5p	24	AACACCAACCAGAACTAAAGATCC	13	55	56.4645	49.4243
miR5168-y	21	TCGGACCAGGCTTCAATCTTT	14	54	60.808	48.5257

novel-m0105-3p	21	TCAGCGCCACATAGGATTCTA	24	53	104.2422	47.627
novel-m0053-3p	24	TTGTACAAGAGATGGGAAAGGACC	30	53	130.3028	47.627
novel-m0421-5p	24	AAAACCGGCACCTATACGTAGTAC	21	53	91.2119	47.627
novel-m0217-3p	24	ATCATCCGGTGGAACTTGGCACCT	17	53	73.8382	47.627
miR5150-y	25	GAAGCTGCAGCTGTCAGAAGCTCCA	14	53	60.808	47.627
miR2120-y	22	TTTAGTCCCGGTTGGTGTTACT	16	52	69.4948	46.7284
miR1423-x	24	GCAACTACACGTTGGGCGCTCGAA	15	52	65.1514	46.7284
novel-m0086-3p	24	ATCGGACCAACCATGATTTATACA	13	51	56.4645	45.8298
novel-m0087-3p	24	ATCGGACCAACCATGATTTATACA	13	51	56.4645	45.8298
novel-m0106-5p	24	GATACCCTGTCGAACAACCACAGT	16	51	69.4948	45.8298
novel-m0107-5p	24	GATACCCTGTCGAACAACCACAGT	16	51	69.4948	45.8298
osa-miR1846e	20	CAACGAGGAGGCCGGGACCA	27	50	117.2725	44.9312
miR6225-x	20	TAGGCTCAAAAGATTCGTCT	13	50	56.4645	44.9312
novel-m0114-3p	24	AATATCCAGTTGCTAGAAGCTCAC	21	50	91.2119	44.9312
novel-m0078-3p	24	ACGCTAGCTGCGTTGGCGCTGAGA	17	50	73.8382	44.9312
osa-miR5827	21	TTTGTTGCAATTTGGACTACC	13	49	56.4645	44.0326
novel-m0288-5p	22	TTGATGTGGCAACAATGATGAT	13	49	56.4645	44.0326
osa-miR812t	24	ACGGAAAATCATGGCTGCACTTAA	15	49	65.1514	44.0326
osa-miR812u	24	ACGGAAAATCATGGCTGCACTTAA	13	49	56.4645	44.0326
miR1874-y	24	TATGGATGGAGGTGTAACCCGAGA	12	49	52.1211	44.0326
novel-m0091-3p	24	AATTTGGCTTAGAACCGGCACCTA	13	49	56.4645	44.0326
novel-m0147-5p	24	TTGAGATCCTCTGCAGTACTGCAT	22	49	95.5554	44.0326
novel-m0100-5p	24	ACCGGCACCTTTCTCTGACACAGG	12	48	52.1211	43.1339
novel-m0098-3p	24	ACTTCCGGGTTGTCTGAACAGACC	19	48	82.5251	43.1339
novel-m0251-3p	24	AGAGATTGGCGCTGAGATATAACA	24	47	104.2422	42.2353
novel-m0126-5p	24	AACACCAACCAGGACTAGAACACC	18	47	78.1817	42.2353
osa-miR812n-3p	24	ACGGAAAATCATGGCTGCACTTAA	14	46	60.808	41.3367
osa-miR166h-5p	21	GGAATGTTGGCTGGCTCGAGG	31	45	134.6462	40.4381
miR444-y	23	TGCAGTTGTTGTCTCAAGCTTTT	12	44	52.1211	39.5394
miR2121-z	24	GAAAACGGAACGGTCTATTAGCGC	13	44	56.4645	39.5394
novel-m0313-3p	24	ACAACCGGGACTAAAGATAGATCT	14	44	60.808	39.5394
novel-m0237-5p	24	AACAACCTAGGATAGGATGAGACC	12	44	52.1211	39.5394

novel-m0128-3p	24	AAGTTCCGGACAGTCTGAGCAGAC	19	43	82.5251	38.6408
novel-m0108-5p	24	TCCTAGGACAATGTATCTGGACAT	18	43	78.1817	38.6408
novel-m0109-5p	24	TCCTAGGACAATGTATCTGGACAT	18	43	78.1817	38.6408
novel-m0065-5p	24	AGTTGTGGGTTAAGAACTGGCACC	23	42	99.8988	37.7422
novel-m0149-3p	24	TAAGTCCGGATTGGTGTTACCAAC	30	41	130.3028	36.8436
novel-m0134-5p	24	GTCTCCACTTTTGTAGAATTACGG	14	41	60.808	36.8436
novel-m0137-3p	24	AGAACGACTTACACTGTGAAACGG	14	41	60.808	36.8436
miR159-x	21	AGCTGCTTGTTCATGGTTCCC	14	39	60.808	35.0463
novel-m0080-3p	24	AACCGACACCTGTAGAATAGATGT	17	37	73.8382	33.2491
novel-m0403-5p	24	AATTACGGCCCGTCACTGATAGGT	14	36	60.808	32.3504
novel-m0404-5p	24	AATTACGGCCCGTCACTGATAGGT	14	36	60.808	32.3504
novel-m0095-3p	24	ATGTCCAGATTCGTCGTATTAGGA	12	36	52.1211	32.3504
novel-m0099-5p	24	AATACTGTGCATGGACTCCGTGTA	20	36	86.8685	32.3504
osa-miR5807	24	AGGAGGTCTGGAGAGTTATGTGGC	17	35	73.8382	31.4518
novel-m0226-3p	24	GAGATACTAGGATGTGTTACCTCC	16	35	69.4948	31.4518
novel-m0450-5p	24	TTAATCCCGGTCAGTAACATCAAC	19	35	82.5251	31.4518
miR1876-z	25	CATAAGTGGGTTTGTGGGCTGGCCC	16	35	69.4948	31.4518
osa-miR166k-5p	21	GGTTTGTTGTCTGGCTCGAGG	12	31	52.1211	27.8573
novel-m0129-3p	21	TTGAGCCGCGCCAATATCTCT	13	30	56.4645	26.9587
novel-m0176-5p	24	TAGTCACCTGTGACGGGCCGAGAA	15	30	65.1514	26.9587
novel-m0135-5p	24	AACACCAACCGGAACTAAAGATTC	14	30	60.808	26.9587
novel-m0136-5p	24	AACACCAACCGGAACTAAAGATTC	14	30	60.808	26.9587
novel-m0505-5p	24	ATCACCCCGATACGTCGTAGAAAT	14	30	60.808	26.9587
novel-m0506-5p	24	ATCACCCCGATACGTCGTAGAAAT	14	30	60.808	26.9587
novel-m0507-5p	24	ATCACCCCGATACGTCGTAGAAAT	14	30	60.808	26.9587
novel-m0508-5p	24	ATCACCCCGATACGTCGTAGAAAT	14	30	60.808	26.9587
novel-m0208-5p	24	AGCCGAGCCATGACGTGAAAACCT	12	30	52.1211	26.9587
osa-miR1848	21	CCTCGCCGGCGCGCGCGTGCA	15	29	65.1514	26.0601
miR1130-y	24	ATCTTATATTATTGGACGAAGGGA	13	29	56.4645	26.0601
novel-m0111-3p	24	AACACCACGTAGGACGAAGATCAT	13	29	56.4645	26.0601
novel-m0172-3p	24	GTTAAAATTAGTCACGTAGGCGCC	12	29	52.1211	26.0601
novel-m0139-5p	24	AGGACCGGACCGGACATCGAACCG	13	28	56.4645	25.1615

novel-m0140-5p	24	AGGACCGGACCGGACATCGAACCG	13	28	56.4645	25.1615
novel-m0084-3p	24	AACCGGCACCTATAGAATAAGTGT	19	27	82.5251	24.2628
novel-m0085-5p	24	AACCGGCACCTATAGAATAAGTGT	19	27	82.5251	24.2628
novel-m0092-5p	24	AGGATCCTCTGACGTGAAGTAAGA	17	27	73.8382	24.2628
novel-m0159-5p	24	ATAATAACTTTTGGCTATGAATCT	13	27	56.4645	24.2628
novel-m0160-5p	24	ATAATAACTTTTGGCTATGAATCT	13	27	56.4645	24.2628
novel-m0209-3p	24	ATATCCCAGATTCGTAGTACTGTC	14	26	60.808	23.3642
novel-m0625-5p	24	ATTTAGCACCCTGTTGAACAACCG	14	26	60.808	23.3642
novel-m0509-5p	24	ATCACCCCGATACGTCGTAGAAAT	12	26	52.1211	23.3642
osa-miR7693-3p	22	GACGTCCATCGATGAAGAGCGA	12	25	52.1211	22.4656
novel-m0144-5p	24	AGTACCGGATATGACACATACGTC	13	25	56.4645	22.4656
novel-m0475-5p	24	AGCAACTTAGAATCGGATTGGACG	17	24	73.8382	21.567
novel-m0252-3p	24	AGAAGCTGTGGACTGTTTGGGACA	13	24	56.4645	21.567
novel-m0164-3p	24	ACGACAGGAGCATGGCTCTGCGCC	17	23	73.8382	20.6683
novel-m0483-3p	24	AGGCACTCGGTACTGTTAAGGTAT	14	23	60.808	20.6683
novel-m0115-5p	22	TTGAGTGCAGCGTTGATGAACA	15	22	65.1514	19.7697
miR168-x	21	TCGCTTGGTGCAGGTCGGGAA	22	19	95.5554	17.0738
novel-m0132-3p	24	AGTCCCGGTTGTTGTAACATCATC	15	19	65.1514	17.0738
novel-m0558-5p	24	ATGCCCGATTGAGATGACTAGTCA	14	19	60.808	17.0738
novel-m0183-5p	24	GTCACGGATTGGTAAGATGGCGCC	12	18	52.1211	16.1752
novel-m0194-5p	24	AGACGCAGGACTGCTGGTCAGACC	13	18	56.4645	16.1752
novel-m0238-3p	24	AATTCTAGGTTGTTTAGTACTCCC	13	18	56.4645	16.1752
novel-m0180-5p	24	ATACATGGCTGAGGCTGCAGACCA	13	17	56.4645	15.2766
novel-m0345-5p	24	ATCGGATCCGGTACTTATAGGTAT	16	16	69.4948	14.378
novel-m0456-3p	24	ACCGGAACTATTGTGAAAATCAGC	12	14	52.1211	12.5807
novel-m0321-5p	24	ATAGAATCTCCAGTTACCAGAAGC	12	13	52.1211	11.6821
novel-m0454-5p	24	AATACCGGATAAGACACTTTATAG	13	12	56.4645	10.7835
novel-m0289-5p	24	AACACCACCAAGGACTAAAGATCC	12	10	52.1211	8.9862
novel-m0096-3p	24	ACCTTCCCTATCTGTGGAGCTCCT	32	9	138.9896	8.0876
novel-m0312-3p	24	AATAGATGTCTCGCTGTTGATGGG	12	8	52.1211	7.189
novel-m0319-5p	24	ATGGCCAAATAACACTGACACCAG	17	8	73.8382	7.189
novel-m0346-3p	24	ATGGCACCCCATGACTCCTTGAGC	13	6	56.4645	5.3917

novel-m0669-5p	24	ATGAGGGAATGATAGGGCTGTCCC	15	6	65.1514	5.3917

Supplementary Table S4. Germination rates of 98 varieties selected from 3,000 Rice Genomes Project.

Cultivar Name	Cultivar ID	Subpopulation	Genotype	Germination rate (%)
TANG_DU_GU	IRIS_313-11967	Indica Intermediate	Reference	0.00
CT45	IRIS_313-11153	Temperate Japonica	Reference	0.33
7507-137	IRIS_313-9438	Temperate Japonica	Reference	0.67
HWANGJO	IRIS_313-9379	Temperate Japonica	Reference	1.67
LU_TAO_2	IRIS_313-10453	Japonica Intermediate	Reference	2.00
SHINCHIKU-IKU_97	IRIS_313-10631	Temperate Japonica	Reference	3.00
BETIS	IRIS_313-10083	Japonica Intermediate	Reference	4.00
None	CX224	Temperate Japonica	Reference	4.33
CHUSEI_HONEN	IRIS_313-10570	Temperate Japonica	Reference	5.33
IR_68333-R-R-B-19	IRIS_313-10373	Temperate Japonica	Reference	5.33
CHINES	IRIS_313-10618	Temperate Japonica	Reference	6.00
JEONBUKGUNGWEONNA	IRIS_313-10067	Tropical Japonica	Reference	7.67
M_203	IRIS_313-8444	Japonica Intermediate	Reference	8.00
None	CX324	Temperate Japonica	Reference	8.33
CHIYODA_WASE	IRIS_313-10071	Tropical Japonica	Reference	8.67
TAICHUNG_65	IRIS_313-9701	Temperate Japonica	Reference	10.00
CI_1600	IRIS_313-9463	Temperate Japonica	Reference	11.33
TEBONNET	IRIS_313-8434	Tropical Japonica	Reference	15.67
BLUE_BELLE	IRIS_313-9491	Tropical Japonica	Reference	17.33
UKON-NISHIKI	IRIS_313-10585	Intermediate	Reference	21.67
RIKUTO_NORIN_MOCHI_20	IRIS_313-8400	Tropical Japonica	Reference	24.67
ALEXANDROS	IRIS_313-8209	Tropical Japonica	Reference	25.33
None	CX494	Temperate Japonica	Reference	29.00
Yungeng_23	CX345	Temperate Japonica	Reference	30.67
IR_73688-57-2	IRIS_313-10379	Temperate Japonica	Reference	31.33
None	CX519	Temperate Japonica	Reference	32.00
S_201	IRIS_313-8856	Temperate Japonica	Reference	35.00
GAO_JIAO_YING_GAN_ZHAN	IRIS_313-11748	Indica Intermediate	Reference	36.67
Wuyugeng_20_	CX350	Temperate Japonica	Reference	39.50
S_102-2	IRIS_313-8134	Temperate Japonica	Reference	40.67
32_UPLA	IRIS_313-8064	Tropical Japonica	Reference	41.67
HOKURIKU_52	IRIS_313-10056	Tropical Japonica	Reference	43.67
KYUUSHUU	IRIS_313-10082	Tropical Japonica	Reference	43.67
BENLLOK	IRIS_313-9782	Temperate Japonica	Reference	44.00

MARANHAO_BRANCO	IRIS_313-11428	Tropical Japonica	Reference	44.00
None	CX325	Indica Intermediate	Reference	45.67
None	CX493	Temperate Japonica	Reference	46.00
Jinyuan_85	CX389	Temperate Japonica	Reference	47.33
None	CX523	Temperate Japonica	Reference	47.33
Yueguang	CX330	Temperate Japonica	Reference	49.33
None	CX550	Indica Intermediate	Reference	51.00
OITA-MII_120	IRIS_313-10568	Temperate Japonica	Reference	51.67
LUO_AI_ZAO_3	IRIS_313-11667	Indica I	Reference	53.00
YOSHINO_MOCHI	IRIS_313-10078	Tropical Japonica	Reference	57.00
CALROSE_76	IRIS_313-8669	Tropical Japonica	Reference	58.00
RIKU_AIKOKU	IRIS_313-10074	Tropical Japonica	Reference	58.33
053A-3	CX351	Temperate Japonica	Reference	59.00
91_UPLA	IRIS_313-8058	Tropical Japonica	Reference	63.00
75_UPLA	IRIS_313-8061	Tropical Japonica	Reference	65.33
YONG_AN_HUK	IRIS_313-9891	Temperate Japonica	Reference	65.50
24869	IRIS_313-8399	Temperate Japonica	Reference	75.67
RR_272-17-829	IRIS_313-10412	Indica Intermediate	Reference	84.00
BAMOA_A75	IRIS_313-9953	Indica Intermediate	Reference	10.67
SML_AWINI	IRIS_313-9841	Indica Intermediate	Reference	10.67
C_662083	IRIS_313-10001	Indica II	Reference	13.00
UPL_RI-7	IRIS_313-7797	Indica Intermediate	Reference	26.33
IR64-IL	CX230	Indica II	Reference	41.33
CT_6510-24-1-2	IRIS_313-7665	Indica Intermediate	Reference	43.67
GUI_HUA_ZAO	IRIS_313-10179	Indica Intermediate	Reference	47.00
IR68	CX278	Indica Intermediate	Reference	50.67
None	CX472	Indica Intermediate	Reference	55.00
IR_80310-12-B-1-3-B	IRIS_313-10394	Indica Intermediate	Reference	61.00
JHODI_BIRUN	IRIS_313-9262	Indica III	Reference	63.67
None	CX502	Temperate Japonica	Reference	78.00
Huanyangnian	CX88	Indica I	Reference	79.67
None	CX178	Indica Intermediate	139bp deletion	13.33
IR_77390-1-6-4-19-1-B	IRIS_313-10392	Indica II	139bp deletion	15.67
IR_57920-AC-25-2-B	IRIS_313-7698	Indica Intermediate	139bp deletion	19.00
DANAU_LAUT_TAWAR	IRIS_313-7668	Indica Intermediate	139bp deletion	28.00
Basmati	CX60	Indica II	139bp deletion	29.33

None	CX505	Indica II	139bp deletion	34.67
None	CX513	Indica II	139bp deletion	34.67
ZALE	CX131	Indica I	139bp deletion	40.33
Zhongjian_100	CX548	Indica Intermediate	139bp deletion	40.67
Yuexiangzhan_	CX17	Indica Intermediate	139bp deletion	42.67
MIN_KE_ZHAN	IRIS_313-10191	Indica Intermediate	139bp deletion	45.33
HURANG_ARISO_LUTA	IRIS_313-10966	Indica Intermediate	139bp deletion	51.67
None	CX514	Indica Intermediate	139bp deletion	59.00
None	CX539	Indica I	139bp deletion	61.33
None	CX176	Indica II	139bp deletion	62.33
RACE_PERUMAL	IRIS_313-9970	Indica III	139bp deletion	62.67
B_6136-3-TB-0-1-5	IRIS_313-10333	Indica II	139bp deletion	63.33
CO_39	IRIS_313-8265	Indica Intermediate	139bp deletion	63.67
None	CX222	Indica Intermediate	139bp deletion	64.33
None	CX510	Indica II	139bp deletion	64.67
None	CX512	Indica II	139bp deletion	66.00
NPT-100	CX385	Indica II	139bp deletion	68.00
None	CX469	Indica II	139bp deletion	69.33
HSIEH-DAU	IRIS_313-10504	Indica I	139bp deletion	73.50
NPT-114	CX386	Indica Intermediate	139bp deletion	76.00
KHAO'_SIM	IRIS_313-10928	Indica III	139bp deletion	77.67
None	CX475	Indica II	139bp deletion	82.67
None	CX509	Indica Intermediate	139bp deletion	83.33
Zhong_413	CX19	Indica Intermediate	139bp deletion	83.67
None	CX467	Indica Intermediate	139bp deletion	85.67
Huhan_15	CX378	Indica II	139bp deletion	89.33
None	CX480	Indica II	139bp deletion	92.67
Linyitangdao	CX54	Indica Intermediate	139bp deletion	94.00

Supplementary Table S5. The primer sequences used for map-based cloning, genotyping, transgene constructs and RT-qPCR assays.

Primers	Forward sequence (5'-3')	Reverse sequence (5’-3’)	Applications
Indel 5	TCCAACTACCAGCTCCGATG	TTAAGCTTCTTGTGCCTGGC	Map-based cloning
Indel 14	TGGAAGTCGTCTCTGAACCT	AGGTATACGGTGGCTTCTCC	Map-based cloning
Indel 15	AAACATTCCGGCCTTGCAAA	ATTGGCTCCGATGTTGGGTA	Map-based cloning
Indel 16	CCTCCAAATGCCAGCGATTA	GGGAGAGATGGTTGATTGGC	Map-based cloning
Indel 19	ATACGTATTGCCGACCTTGC	AAGAGGCAGTTGTGGTGGTA	Map-based cloning
Indel 20	GGTGGAGATCGAACGAGGAA	ATCAATCCACCTCCCACACC	Map-based cloning
Indel 21	TGTCATGTCATCCTGGCGT	CGCTGCTCATGTCTGTTCC	Map-based cloning
Indel 30	GGGACTCCACGTAGAAGTTC	GTTGGGTTGGGGTTTGGTT	Map-based cloning
Indel 31	GCAGTTGAGCATGAGGAGC	AGCCTCAAGTCTCGGTCAC	Map-based cloning
Indel1a8	TTCTTTCGCTGCAATGCTTA	CAAAGCGCAGTGGCTTATCT	Genotyping
Indel1a5	TCCAACTACCAGCTCCGATG	TTAAGCTTCTTGTGCCTGGC	Genotyping
Indel1a10	ACCCGTTGTTGATGTGCATT	ACGTAAAATCAACCCGGAAA	Genotyping
Indel1b5	TACCACTGGAATCTCGAACG	GGCTACGGTATGTTCCTTGA	Genotyping
Indel1b7	GTACCAGCTTAAGAGGGCC	GTGGTCAAACACGAGGTAATTAC	Genotyping
Indel2a4	ACCAGATACCAGTGTCGTGA	GGGATCATGATTGGAAATGCTT	Genotyping
Indel2a11	CAAGGTATTGCGGTTTTCGT	AAGCCAGCTGATGTCCAACT	Genotyping
Indel2a13	CCACGTCACCCCAAATTAAG	AGACTCGAGAGCGAGGTGAC	Genotyping
Indel4a7	ACTAACACGGCCTCAACATC	ATCAATCATTGGAAGGCCCC	Genotyping
Indel4a9	TGAATCCATCGTGAGTGTGC	CGAAACGATGGCCTCCTATC	Genotyping
Indel4a15	GAAGCAGAGCATAGCCCTTA	TGTCGTTTGGAGTACTGCAT	Genotyping
Indel7a1	GACTTTCTGCGACCCCTTTT	CTTTAGCAGCCACACAGCC	Genotyping
Indel7a6	AAGCGTGTTCAGTTCCCATT	CCACCGCAAAATTCTCGTTG	Genotyping
RDR3-indel	TCACATTTTCTCAATATGCTTGCCA	AGAAATGGACCAGCTACCCG	Genotyping
OsRDR3-pDONR	GGGGACAAGTTTGTACAAAAAAGCA<br>GGCTCCATGTACAACCCCATCGGCTC	GGGGACCACTTTGTACAAGAAAGC<br>TGGGTCCTACCTGCGATGATCCTTCT	Transgene constructs
qRDR3	CAGGTAGAGTGTCGTGTT	TGCTCCTAACATTACTGCTA	RT-qPCR
qGRF1	AAAGAGGACGACGATGAGAAAGAG	GCCCAGGAGGAAGCAGTG	RT-qPCR
qGRF2	TACGGACGGCAAGAAGTG	GGCATTTCCACAGGCTTTC	RT-qPCR
qGRF3	CAATGCTGCGTCTTACTC	AATGTGGAGGTCTGAGAAG	RT-qPCR
qGRF4	CATCTGTTGTCGGTTCTG	GCAATAGCAGGGTAAAGAG	RT-qPCR
qGRF5	TTCTTCTCAGGAGCATCAG	GTTCAAGGTGGGAGTAGG	RT-qPCR

qGRF6	CCTCGCTATCAACCATCAG	GCACTTGTTCACTCTCATTATG	RT-qPCR
qGRF7	TTGGATCAGGTGGCTATC	TTGTGTTGGTGTGAATGG	RT-qPCR
qGRF8	GCAAGAGCAAGAGCAAGATG	CCAGCTCCATCCACTGTGTT	RT-qPCR
qGRF9	GCTCATTGCCATCTTCTGTC	GTTCGCCATTGTCCTGTTC	RT-qPCR
qGRF10	TGCTCATCTACCGCTACTTC	CGACGCTCTTCCAGATGG	RT-qPCR
qGRF11	TGCCTACTCATCTCGTCTTC	GTTCTGGTTCTGGGTTCTTTC	RT-qPCR
qGRF12	TCAAGAAAGCCTATGGAAGCCTCTG	TGGCGACGGTGTGGAGTG	RT-qPCR
qβ-OsLCY	TCTTCGACGCCTTCTTCGAC	GATCATCTTGGCGAGAGGGG	RT-qPCR
qZDS	TCCGAAAGGATTGTTCCCGC	ACAGATCAACCTCATGGCCC	RT-qPCR
qISA1	CTGTTCAATCGGACGGGGAA	ACACCATACTCTCCTCGGCT	RT-qPCR
qOsGRX3	CGGGCTCGATCGGCTCATGG	GAGCCACAGGGCTCCTGCCT	RT-qPCR
qABA8ox1	ACCTCGCAACCAAGTACAGG	CACTCCTGCTCGGTGTTCTT	RT-qPCR
qOsPP2C51	TGACGAGTTGGAACGAGTGG	CTCGCCAGGATCAGGAACTC	RT-qPCR
qSD6-F	CTGAGCCTCCATTTCACCTG	GATGTGATGATAGGTGGTGC	RT-qPCR
qRAmy3D	GGCTCGACGCAGAAGCTT	GACGACGGCGACGTATGC	RT-qPCR

Supplemental Materials & Methods

Plant materials and field growth conditions

The population of random crossing-recombinant inbred lines (RC-RILs, RC_4F_6) used for QTL analysis was developed from the cross between JiafuzhanS (JFZS) and Nipponbare (NIP) through random crossing for four generations (beginning from F_1) followed by consecutive selfing for five generations (Supplemental figure 1). The field experiments were conducted in standard paddy conditions with a spacing of 20 cm between plants and rows at Shaxian (Fujian Province) and Sanya (Hainan Province).

Germination assay

Germination assays were performed as previously described with some modifications (Liao et al. 2009). A higher germination rate indicates lower PHS resistance. 10 mature panicles of each lines were harvested at Shaxian (Fujian Province) or Sanya (Hainan Province). The sampled panicles were soaked in water for 10 h and then incubated with wet towels at 28°C. The number of sprouted spikelets was counted after soaking (spikelet sprouting was defined as the emergence of a coleoptile or radicle through the seed coat). For Nipponbare background plants, germination tests statistics were taken after 7-d imbibition and photographs were taken after 5-d imbibition. For WYJ7 background plants, the germination rate was counted daily for a total of 8 days and photographs were taken after 6-d imbibition. For TaifengB and YexiangB background plants, germination tests statistics were taken after 7-d imbibition.

The germination rate was calculated according to the following formula:

Germination rate = Number of sprouted spikelets / Total number of spikelets × 100%

QTL mapping

The BSA-seq-based QTL mapping approach is based on a previously published work (Tang et al. 2018, Huang et al. 2020). Three leaves pools, including Random pool, PHS resistant pool and PHS sensitive pool, were created from 50 random lines, 50 PHS-resistant lines and 50 PHS-sensitive lines of the RC-RIL population derived from JFZS × NIP, respectively. DNA of each pool was extracted using a DNA extraction kit (TIANGEN), qualified by 1% agarose gel electrophoresis and a NanoDrop 2000 (Thermo Scientific) and then sequenced by JointGene Biotechnology company. The following QTL analysis was performed as reported before.

Based on the QTL mapping results, molecular markers were designed flanking the confidence intervals of the candidate QTLs (Supplementary Table 5). These markers were used to genotype 100 lines from both the PHS-resistant and PHS-sensitive pools. The associations of the markers with PHS were examined using *t*-test. The results of this analysis were employed to assess the validity of the candidate QTLs. As a result, of the five candidate QTLs identified, the one on

chromosome 2 was determined to be a false positive (Supplementary Table 1)

Fine mapping

The map-based cloning of *qPHS1a* was based on 2389 progenies from a single RHL23 plant heterozygous for *qPHS1a*. Sequences of primers used for genotyping assays are given in Supplementary Table 5.

Transgene constructs

The coding sequence (CDS) of *OsRDR3*^{NIP} was amplified from NIP. The CDS of *OsRDR3*^{JFZ} was amplified from JFZ. These amplified DNA fragments were then inserted into the CAMBIA2300 (CAMBIA, http://www.cambia.org/) vectors to generate the *p35S::OsRDR3*^{NIP} and *p35S::OsRDR3*^{JFZ} constructs, respectively. The vector the CRISPR-Cas9-generated *OsRDR3* loss-of-function allele (*osrdr3*) in the NIP genetic background was made as described elsewhere (Ma et al. 2015). Transgenic rice was generated by Agrobacterium-mediated transformation as described elsewhere (Huang et al. 2009). Relevant primer sequences are given in Supplementary Table 5.

RNA-seq and miRNA-seq analysis

Fresh seed embryos of wild-type Nipponbare and *rdr3-c1* mutant were collected after soaked in water for 24 h. Three biological replicates were performed for RNA-seq. The samples were then stored on dry ice and shipped to Gene Denovo Biotechnology Company Limited (Guangzhou) for RNA library construction and sequencing. The FPKM values of the differentially expressed genes in the three individual replicates are given in Supplementary Table 2. RNA-seq data were analyzed using a standard pipeline for differential expression analysis. Briefly, raw sequencing reads were first trimmed for adapter sequences and low-quality bases using Trimmomatic (v0.39) (Bolger et al. 2014). The cleaned reads were then aligned to the rice reference genome v7.0 (NCBI taxonomy ID: 4530) using HISAT2 (v2.2.1) (Kim et al. 2015). Gene expression levels were quantified as fragments per kilobase of transcript per million mapped reads (FPKM) using StringTie (v2.1.4). Differentially expressed genes were identified using edgeR (Pertea et al. 2015) with $P < 0.05$ and log2 fold-change cutoff of ± 0.5.

After total RNA was extracted, the RNA molecules in a size range of 18–30 nt were enriched by polyacrylamide gel electrophoresis, followed by sequential ligation of 3′ and 5′ adapters. The ligated products were reverse-transcribed, PCR-amplified, and size-selected (140–160 bp) to generate cDNA libraries and sequencing. Raw reads were processed to remove adapters and low-quality sequences. Clean tags were filtered by alignment against the GenBank (Release 209.0) and Rfam (Release 11.0) databases to eliminate non-miRNA small RNAs (rRNA, scRNA, snoRNA, snRNA, tRNA). Reads mapping to exons, introns, or repetitive regions in the reference genome were discarded. Known miRNAs were annotated using miRBase (Release 22). Novel miRNAs were predicted from unannotated reads using miRDeep2, based

on genome alignment and hairpin structure evaluation. miRNA expression levels, quantified as transcripts per million (TPM), are provided in Supplementary Table 3. miRNAs with zero counts in either the wild type or *rdr3-c1* mutant, or with expression levels below 50 TPM in both wild type and *rdr3-c1*, were excluded.

RT-qPCR

Total RNAs were extracted from fresh seed embryos after soaked in water for 24 h using HiPure Plant RNA Mini Kit B. Full-length cDNAs were then reverse transcribed using a cDNA synthesis kit (Baimeng Biotechnology company). RT-qPCRs were performed according to the manufacturer's instructions (Baimeng Biotechnology company), with three independent RNA preparations as biological replicates. Rice *Actin1* gene transcripts were used as references. The sequences of relevant primers are given in Supplementary Table 5.

ABA content detection

The fresh seeds (about 25 days after pollination) of NIP and *osrdr3-c1* were stored at -80°C. Approximately 200 mg (fresh weight) seeds were homogenized under liquid nitrogen, weighted and extracted for 24 h with methanol and $^{2}H_{6}$-ABA. Endogenous ABA was purified and measured as previously described (Fu et al. 2012; Chu, et al. 2017) with following modifications in detection conditions. LC-MS/MS analysis was performed on a UPLC system (Waters) coupled to the 5500 Qtrap system (AB SCIEX). LC separation used a BEH C18 column (1.7μm, 100 × 2.1 mm; Waters) with mobile phase A, 0.1% (v/v) formic acid in water, and B, acetonitrile. The gradient was as follows: 0-0.5 min, 5% B, 0.5-14 min, 5% B to 25% B; 14-16 min, 25% B to 100% B; 16-16.5 min, 100% B; 16.5-17.5 min, 100% B to 5%B; 17.5-19.0 min, 5% B to 5%B. ABA was detected in multiple reaction monitoring (MRM) mode with transition. The transitions for ABA and [$^{2}H_{6}$]-ABA are 263.0>153.1 and 269.2>159.2. Three biological replicates were analyzed for each sample.

Statistical analysis

Statistical analysis was performed using Duncan's multiple range test for multiple comparisons, or two-tailed independent Student's *t*-tests for two-group comparisons. Differences were considered statistically significant at $P < 0.05$. All statistical computations were conducted with Microsoft Excel 2021.

References

Bolger AM, Lohse M and Usadel B (2014) Trimmomatic: a flexible trimmer for Illumina sequence data. *Bioinformatics* **30** :2114-2120.

Chu, J., Fang, S., Xin, P., Guo, Z., and Chen, Y. (2017). Quantitative analysis of plant hormones based on LC-MS/MS. In: Hormone Metabolism and Signaling in Plants--Li, J., Li, C., and Smith, S.M., eds. London: Elsevier. 471-537.

Fu, J., Chu, J., Sun, X., Wang, J., and Yan, C. (2012) Simple, rapid, and simultaneous assay of multiple carboxyl containing phytohormones in wounded tomatoes by UPLC-MS/MS using single SPE purification and isotope dilution. *Anal Sci* **28**: 1081-1087.

Huang L, Tang W, Bu S and Wu W (2020) BRM: A statistical method for QTL mapping based on bulked segregant analysis by deep sequencing. *Bioinformatics* **36**: 2150-2156.

Huang XZ., Qian Q, Liu ZB, Sun HY, He SY, Luo D, Xia GM, Chu CC, Li JY and Fu XD (2009) Natural variation at the DEP1 locus enhances grain yield in rice. *Nature Genetics* **41**: 494-497

Kim D, Langmead B and Salzberg S (2015) HISAT: a fast spliced aligner with low memory requirements. *Nature Methods* **12**: 357–360.

Liao Y, Zhen C, Gao M, Li D, Xue J, Zhang H, Xu P and Wu X (2009) Studies on testing methods of pre-harvest sprouting of male sterile lines in hybrid rice seed production. *Hybrid Rice* **24**: 22-23

Ma X, Zhang Q, Zhu Q, Liu W, Chen Y, Qiu R, Wang B, Yang Z, Li H, Lin Y, Xie Y, Shen R, Chen S, Wang Z, Chen Y, Guo J, Chen L, Zhao X, Dong Z and Liu YG (2015) A robust CRISPR/Cas9 system for convenient, high-efficiency multiplex genome editing in monocot and dicot plants. *Molecular Plant* **8**: 1274-1284

Pertea M, Pertea G, Antonescu C, Chang T, Mendell J and Salzberg S (2015) StringTie enables improved reconstruction of a transcriptome from RNA-seq reads. *Nature Biotechnology* **33**:290-5

Tang W, Huang L, Bu S, Zhang X and Wu W (2018) Estimation of QTL heritability based on pooled sequencing data. *Bioinformatics* **34**: 978-984.
